# Potential preventive role of low-dose methotrexate against incident recorded psychosis: a retrospective cohort study based on electronic health records

**DOI:** 10.1016/j.eclinm.2026.104111

**Published:** 2026-07-23

**Authors:** Fabiana Corsi-Zuelli, Maxime Taquet, Bill Deakin, Rachel Upthegrove

**Affiliations:** a Department of Psychiatry, University of Oxford, Warneford Hospital, Oxford, UK; b Oxford Health NHS Foundation Trust, Oxford, UK; c Division of Psychology and Mental Health, University of Manchester, Manchester, UK; d Institute for Mental Health, University of Birmingham, Birmingham, UK; e Early Intervention Service, Birmingham and Solihull Mental Health Foundation Trust, UK

**Keywords:** Low-dose methotrexate, Psychosis, Rheumatoid arthritis, Anti-inflammatory drugs, Regulatory T cells

## Abstract

**Background:**

Recent evidence suggests that low-dose methotrexate might have antipsychotic properties. However, it remains unknown whether low-dose methotrexate is associated with a reduced risk of incident recorded psychosis in real-world data, whether these associations extend to other putatively immune-related common psychiatric conditions, or whether similar associations are observed with other disease-modifying anti-rheumatic drugs (DMARDs).

**Methods:**

In this retrospective cohort study using electronic health records (TriNetX US Collaborative Network), we identified adults with rheumatoid arthritis (age ≤45 years at treatment initiation; data extracted from 1 January 2000 to 21 December 2025). The 5-year risk of an incident recorded psychosis (primary outcome) and bipolar, depression, or anxiety disorders (secondary outcomes) were compared between low-dose methotrexate and each of 14 comparator drugs used in rheumatoid arthritis - three primary comparators (non-steroidal anti-inflammatory drugs; NSAIDs, naproxen, diclofenac and celecoxib) and 11 secondary and exploratory DMARDs comparators. Cumulative incidences and ratios of restricted mean time lost (rRMTL) are reported. Results were Bonferroni-corrected for multiple comparison.

**Findings:**

Comparator cohort sizes ranged from 1161 to 21,445 (mean ages 33.7–37.4 years). For the primary outcome of psychosis, initiation of low-dose methotrexate was associated with lower 5-year incidence of newly recorded psychosis than initiation of naproxen (rRMTL 0.69, 95% CI 0.55–0.87) or diclofenac (rRMTL 0.71, 0.56–0.89); no significant difference was observed versus celecoxib or biologic DMARDs. For the secondary outcomes, low-dose methotrexate was similarly associated with lower 5-year incidence of newly recorded bipolar disorder, depression, and anxiety compared with non-selective NSAIDs naproxen and diclofenac, with the mood and anxiety associations extending to the selective cyclo-oxygenase-2 inhibitor celecoxib.

**Interpretation:**

In a real-world rheumatoid arthritis cohort, initiation of low-dose methotrexate was associated with lower 5-year incidence of newly recorded psychosis, bipolar disorder, depression, and anxiety than initiation of non-selective NSAIDs. Given that extensive trials of broad anti-inflammatories have yielded limited psychiatric benefit, the differential profile of low-dose methotrexate is consistent with a mechanism beyond simple inflammation suppression. We hypothesise potentiation of regulatory T cell-mediated control of systemic inflammation and neuro-glial regulation. The active-comparator observational design cannot establish causation; these findings are hypothesis-generating and confirmatory inference will require interventional studies.

**Funding:**

UK Research and Innovation (UKRI) Medical Research Council [grant number UKRI4403] Mental Health Platform. The National Institute for Health and Care Research (NIHR) Oxford Health Biomedical Research.

**Research in context**

**Evidence before this study**

A repurposing study reported that the major disease-modifying anti-rheumatic drug (DMARD) methotrexate, at low-doses, reduced symptoms of psychosis in patients with schizophrenia. In a retrospective cohort study of rheumatoid arthritis, we investigated whether the 5-year incidence of recorded psychosis was lower following initiation of low-dose methotrexate compared with three broad non-steroidal anti-inflammatory drugs (NSAIDs; naproxen, diclofenac, celecoxib) but not in comparison with biologic DMARDs (adalimumab, infliximab, etanercept, tocilizumab). Prior to the study there had been no double-blind randomised placebo-controlled trials (RCTs) of low-dose methotrexate in psychosis, bipolar disorder, depression or anxiety disorders, which we confirmed by searches on PubMed for clinical trials (July 3rd 2025) using the following syntax and search terms, with no language restrictions: “low dose methotrexate AND (psychosis OR schizophrenia OR depression OR bipolar disorder)” and on clinicaltrials.gov, and there have been none since. Employing the same syntax for comparator efficacy, the most recent meta-analysis of adjunctive celecoxib in schizophrenia (7 trials) and in depression (4 trials) reported greater improvement with celecoxib than with placebo but the trials were predominantly small (n < 100) and geographically concentrated. In addition, two large RCTs published after the meta-analyses, found no benefit of celecoxib in depression, and a trial of naproxen in schizophrenia was decisively negative, and there were no trials of diclofenac. Biologic DMARDs have been evaluated only in small add-on studies, insufficient to establish therapeutic efficacy.

**Added value of this study**

This is the first large-scale observational study to suggest that initiating low-dose methotrexate in patients with rheumatoid arthritis is associated with a lower incidence of recorded psychosis and bipolar disorder compared to those taking non-selective NSAIDs (naproxen and diclofenac). Unexpectedly, a substantially lower risk of non-psychotic mood and anxiety disorders was also evident in the low-dose methotrexate group compared with the NSAID groups, including the selective cyclo-oxygenase-2 (COX-2) inhibitor celecoxib. By comparing low-dose methotrexate to both regulatory T-cell (Treg)-neutral treatments (NSAIDs) and Treg-enhancing biological DMARDs, we provide real-world evidence compatible with a role of Treg function in psychiatric protection.

**Implications of all the available evidence**

Our findings suggest that low-dose methotrexate acts as more than a simple anti-inflammatory; its primary psychiatric benefit likely stems from its role as a Treg-immune modulatory drug. In rheumatoid arthritis, low-dose methotrexate is known to promote Treg control of systemic immunity. We propose that this mechanism extends to the central nervous system through the dysregulation of neuro-glial function. Specifically, in psychosis, the reduced association may be driven by central neuro-glial regulation, whereas in mood and anxiety disorders, the effect may stem from a transdiagnostic reduction in systemic inflammation. These results provide clinical reassurance that effectively treating rheumatoid arthritis with low-dose methotrexate is unlikely to increase psychiatric risk and may defer the onset of severe mental illness. The findings are hypothesis generating only and cannot establish causality. Future research should focus on mechanistic studies and confirmatory RCTs to determine if low-dose methotrexate can be repurposed for psychiatric prevention in populations without systemic inflammatory disease.

## Introduction

The influential neuroinflammatory hypothesis of psychosis is based principally on studies that report mildly raised circulating concentrations of inflammatory markers, including C-reactive protein (CRP) and cytokines such as interleukin (IL)-6 and tumour necrosis factor (TNF),^1^ in people with psychosis compared with controls. However, numerous trials of broad anti-inflammatory agents (e.g., celecoxib) have not reported clinically meaningful anti-psychotic benefits in meta-analysis.^2^ Recently, the disease-modifying anti-rheumatic drug (DMARD) methotrexate at low-doses (low-dose methotrexate) was effective in reducing psychotic symptoms in a small controlled clinical trial in people with psychosis.^3^

Low-dose methotrexate is the cornerstone treatment for rheumatoid arthritis and other autoimmune conditions.^4^ The key mechanism of low-dose methotrexate is to restore control of the immune system by specialised CD4+ T cells known as regulatory T cells (Tregs).^5^, ^6^, ^7^ Circulating Tregs have been identified to infiltrate the healthy human brain, where they orchestrate neuro-glial function.^8^ We have previously hypothesised that low-dose methotrexate, despite its negligible brain penetration, might exert an antipsychotic effect by restoring Treg control in brain.^9^ A potential preventive association of low-dose methotrexate with risk of psychosis is one of several possible explanations for the reduced prevalence of schizophrenia in those with rheumatoid arthritis.

In contrast to the inverse association between rheumatoid arthritis and psychosis,^10^, ^11^, ^12^, ^13^ rheumatoid arthritis shows positive association with risk of affective disorders, including major depressive disorder, bipolar disorder, and anxiety disorders.^14^, ^15^, ^16^ However, psychosis and mood disorders are both associated with greater levels of peripheral immune activation.^1^ Whether low-dose methotrexate influences the recorded incidence of psychosis and mood disorders in rheumatoid arthritis in real-world settings is not known. If low-dose methotrexate does not delay mood disorders in rheumatoid arthritis but reduces risk of psychosis, this would suggest distinct immune mechanisms are involved in the disorders.

We used a retrospective cohort study to test our primary prediction that individuals with rheumatoid arthritis prescribed low-dose methotrexate would have a lower recorded incidence of late onset psychosis (defined as non-affective and affective psychosis) at 5 years follow-up compared with those treated with non-steroidal anti-inflammatory drugs (NSAIDs). In a secondary analysis, we predicted that low-dose methotrexate would not reduce late onsets of psychosis in comparison with biological DMARDS that, like low-dose methotrexate, boost Tregs but in different ways via targeting cytokines such as IL-6 (e.g., tocilizumab) and TNF (e.g., infliximab, adalimumab, etanercept). We also explored comparisons with other DMARDS with uncertain effects on Tregs such as hydroxychloroquine and other non-biological DMARDs. Lastly, we explored the effect of low-dose methotrexate on the recorded incidence of other psychiatric outcomes, particularly bipolar disorder, major depressive disorder, and anxiety disorders not expecting a benefit of low-dose methotrexate versus other therapies (NSAIDs, DMARDs).

## Methods

### Study design and data source

This retrospective cohort study used data from the TriNetX US Collaborative Network, a federated health research platform providing access to de-identified electronic health records (EHR), including diagnoses, medications, and procedures, from 70 healthcare organisations across the USA. At the time of analysis, the database included approximately 124 million patients. Data were extracted from 1 January 2000 to 21 December 2025.

### Ethics

No ethical approval was required. The authors were granted unconditional access to the TriNetX US Collaborative Network. The process of data de-identification is attested by a qualified expert as defined in Section 164.514(b) (1) of the Health Insurance Portability and Accountability Act of 1996 Privacy Rule. This formal determination by a qualified expert supersedes the need for TriNetX's previous waiver from the Western Institutional Review Board.

### Cohorts

The primary (exposed) cohort comprised individuals with rheumatoid arthritis treated with low-dose methotrexate who met all of the following inclusion criteria: *a)* recorded diagnosis of rheumatoid arthritis (International Classification of Diseases, 10th Revision, Clinical Modification [ICD-10-CM]: M05–M06), on or before treatment initiation; *b)* initiation of anti-rheumatic treatment at age ≤45 years (after which the risk of psychosis is very low)^17^; and *c)* the index treatment was oral low-dose methotrexate (2–20 mg). We applied the following exclusion criteria: *d)* injectable methotrexate was excluded to avoid contamination by high-dose oncology indications, where parenteral methotrexate is used at doses well above the rheumatoid arthritis range; *e)* a diagnosis of malignancy was excluded to minimise confounding from high-dose methotrexate for cancer treatment^18^; and *f)* a prior diagnosis of psychosis recorded up to the day of rheumatoid arthritis treatment initiation.

The comparator cohorts were constructed using the same criteria *a, b, e* and *f*, with two modifications: the index treatment (criterion *c*) was the comparator drug rather than low-dose methotrexate, and exclusion criterion *d* was replaced by exclusion of any prior low-dose methotrexate exposure. Low-dose methotrexate was compared head-to-head (each drug tested separately) against 14 alternative anti-rheumatic drug treatments approved by the US Food and Drug Administration (FDA; Appendix pp 2–5),^4^ grouped as follows: *i)* the primary comparators were commonly prescribed NSAIDs: naproxen and diclofenac (non-selective inhibitors of cyclo-oxygenase COX-1 and COX-2), and celecoxib (a selective COX-2 inhibitor); *ii)* secondary comparators were biological DMARDs: infliximab, adalimumab, etanercept (TNF inhibitors), tocilizumab (IL-6 receptor inhibitor, IL-6Rs), and the conventional synthetic DMARD hydroxychloroquine; *iii)* exploratory comparators were additional conventional synthetic DMARDs (leflunomide, sulfasalazine, minocycline), a biological DMARD (abatacept), and targeted synthetic DMARDs (tofacitinib and upadacitinib).

Because low-dose methotrexate is typically used as a background first-line DMARD in rheumatoid arthritis, with NSAIDs and other DMARDs added according to symptom control or disease activity,^4^ we did not exclude prior or concurrent NSAID or other DMARD use from the low-dose methotrexate cohort. This approach was adopted to capture a clinically representative population of patients receiving low-dose methotrexate for rheumatoid arthritis, thereby maximising the relevance of the findings to routine clinical practice. However, to assess the influence of baseline co-medication on the results, we run sensitivity analyses (detailed in subgroup and sensitivity analyses section).

For a mechanistic overview of each drug,^19^ see Table 1.

**Table 1:** Summary of mechanism of action for comparator drugs included in the study.

Drug name	Primary mechanism of action (Main target)
Non-steroidal anti-inflammatory drugs	
Naproxen	Inhibits cyclooxygenase enzymes 1 and 2 (COX-1 and COX-2) to reduce the production of prostaglandins and thromboxanes. COX-1 is constitutively expressed in most tissues and is involved in maintaining gastric mucosal integrity, renal function, and platelet aggregation. COX-2 is inducible in response to inflammation and is responsible for increased prostaglandin production involved in pain and inflammation. Naproxen is considered a non-selective COX inhibitor.
Diclofenac	A non-selective COX inhibitor, inhibiting both COX-1 and COX-2. Other potential mechanisms include inhibition of the thromboxane-prostanoid receptor, effects on arachidonic acid release and uptake, inhibition of lipoxygenase enzymes, and activation of the nitric oxide–cGMP antinociceptive pathway.
Celecoxib	A selective COX-2 inhibitor, primarily targeting COX-2 with minimal effect on COX-1. This selective inhibition aims to reduce inflammation and pain with a potentially lower risk of gastrointestinal side effects compared to non-selective NSAIDs.
Biological DMARDs	
Infliximab	A chimeric monoclonal antibody that specifically binds to and neutralises tumour necrosis factor (TNF). TNF is a key pro-inflammatory cytokine in rheumatoid arthritis, driving inflammation, joint damage, and other systemic effects. There is evidence that infliximab increases regulatory T cell (Treg) function.
Adalimumab	A fully human, recombinant monoclonal antibody with high affinity for TNF, functioning as a TNF inhibitor. A potent TNF receptor type R2 agonist, leading to increased Treg function.
Etanercept	A dimeric fusion protein composed of the extracellular portion of the human 75 kDa TNF receptor (p75) linked to the Fc portion of human IgG1. It acts as a decoy receptor, binding to TNF and lymphotoxin (TNF-β), and preventing their interaction with cell surface receptors, thereby inhibiting TNF-mediated inflammation.
Tocilizumab	A humanised anti-human interleukin (IL)-6 receptor antibody that binds to soluble and membrane-bound IL-6 receptor, inhibiting IL-6 signalling. IL-6 is a key cytokine in rheumatoid arthritis pathogenesis, involved in inflammation, joint damage, and systemic symptoms.
Abatacept	A fusion protein of CTLA-4 and IgG1 Fc. It is designed to blocks the co-stimulatory signal (the “second signal”) required for full T-cell activation by binding to CD80 and CD86 on antigen-presenting cells, preventing their interaction with the CD28 receptor on T cells. This leads to inhibited T-cell proliferation and downstream inflammatory responses.
Conventional synthetic DMARDs	
Hydroxychloroquine	Accumulates in acidic cellular compartments like lysosomes, raising pH and interfering with antigen processing and presentation to T cells, reducing immune cell activation. It also affects Toll-like receptor (TLR) 9 signalling.
Leflunomide	Inhibits dihydroorotate dehydrogenase (DHODH), an enzyme critical for the *de novo* synthesis of pyrimidine nucleotides, thus blocking the proliferation of activated lymphocytes.
Sulfasalazine	Its exact mechanism isn't fully understood, but it's believed to have multiple effects. It's converted into 5-aminosalicylic acid (5-ASA) and sulfapyridine. 5-ASA primarily acts locally in the bowel, while sulfapyridine is thought to act systemically. Proposed actions include inhibiting prostaglandin synthesis and free radical scavenging. It may also influence gut microbiota, suppress inflammatory cell function and cytokine/antibody production, inhibit folate-dependent enzymes, and potentially inhibit synovial neovascularization.
Minocycline	While primarily an antibiotic, it exhibits anti-inflammatory and immunomodulatory properties that are independent of its antimicrobial activity. It's thought to reduce inflammation through multiple mechanisms, including inhibition of matrix metalloproteinases (MMPs), phospholipase A-2 (PLA2), and reducing the production of pro-inflammatory cytokines like TNF and IL-1β.
Targeted synthetic DMARDs	
Tofacitinib	Inhibits Janus kinases (JAKs), particularly JAK1 and JAK3, and to a lesser extent JAK2. JAKs are enzymes crucial for intracellular signalling pathways activated by various cytokines involved in inflammation and immune responses. By blocking these enzymes, tofacitinib disrupts the JAK-STAT (signal transducer and activator of transcription) pathway, reducing the production of inflammatory proteins.
Upadacitinib	A selective Janus kinase (JAK) inhibitor, primarily targeting JAK1 over other JAK isoforms like JAK2, JAK3, and TYK2. By inhibiting JAK1-dependent pathways, upadacitinib reduces the signalling of specific cytokines involved in inflammatory diseases, including rheumatoid arthritis.

CD28/CD80/CD86, cluster of differentiation 28/80/86; cGMP, cyclic guanosine monophosphate; COX, cyclo-oxygenase; CTLA-4, cytotoxic.

T-lymphocyte-associated antigen 4; DHODH, dihydroorotate dehydrogenase; DMARDs, disease-modifying anti-rheumatic drugs; Fc, fragment crystallisable region; IgG1, immunoglobulin G1; IL, interleukin; JAK, Janus kinase; JAK-STAT, Janus kinase–signal transducer and activator of transcription; MMPs, matrix metalloproteinases; NSAIDs, non-steroidal anti-inflammatory drugs; PLA2, phospholipase A2; TLR, Toll-like receptor; TNF, tumour necrosis factor; Treg, regulatory T cell; TYK2, tyrosine kinase 2.

### Outcomes

In a time-to-event analysis, we assessed new events of psychiatric outcomes occurring within 5 years of the index event. The index date was the first qualifying prescription of oral low-dose methotrexate or comparator treatment. Psychiatric outcomes were defined as the first recorded diagnosis of the outcome of interest on or after the index date. Follow-up began at the index prescription and continued until the first recorded outcome, death, end of available follow-up, or 5 years after index, whichever occurred first. Individuals without the outcome were censored at the earliest of death, last available record, or the end of the 5-year follow-up period.

The primary outcome was the recorded incidence of psychosis, defined as non-affective psychosis (ICD-10-CM: F20–F29) together with affective psychosis (ICD-10-CM: F30.2, F31.2, F31.5, F32.3, F33.3); these were combined to maximise statistical power, given the low incidence of psychosis after age 35.^17^

We analysed three secondary outcomes: bipolar disorder (ICD-10-CM: F31); depressive episode or recurrent major depressive disorder (ICD-10-CM: F32–F33), without psychosis; and anxiety disorders (any of ICD-10-CM: F41.1, F41, F41.9, F41.8, F40, F41.0, F41.3, F42).

Cases with previous episodes of outcome diagnoses were excluded. See Appendix (p 6) for more information on outcomes.

### Confounding factors

Cohorts were matched for 40 confounding factors including age at index event, sex, ethnicity, socioeconomic indicators, history and familial history of psychiatric disorders, and comorbidities (e.g., metabolic and cardiovascular disease) recorded before or up to the day of the index event. We also adjusted for prior or concurrent corticosteroid prescription, given its frequent use in combination with low-dose methotrexate, particularly in more severe cases,^4^ and its known association with psychosis.^20^ For a full list of confounding factors including diagnostic/medication codes see the Appendix (pp 7–8).

### Statistics

Propensity-score matching was achieved with a greedy nearest-neighbour matching and a calliper of 0.1. Covariate balance was assessed using standardised mean differences (SMDs), with SMD <0.1 indicating adequate balance.

All analyses were performed in R version 4.4.3 unless otherwise stated. Survival analysis with the Kaplan–Meier estimator was used to estimate the cumulative incidence of each outcome. Proportional hazards diagnostics were assessed using the generalised Schoenfeld residual test implemented in cox.zph from the survival package, with Schoenfeld test p < 0.05 indicating evidence of violation of the proportional hazards assumption (Appendix pp 112–113). Given the 5-year follow-up horizon and the possibility that treatment-group differences could vary over time, between-group differences were summarised using restricted mean time lost ratios (rRMTL), which provide a fixed-horizon measure of cumulative outcome burden without requiring the proportional hazards assumption,^21^ an approach applied in previous studies of similar design.^22^^,^^23^ The rRMTL were calculated using the survRM2 package^24^ (version 1.0.4). The rRMTL ratio represents how much more or less time, on average, an individual has lived with the outcome during follow-up in the treatment group relative to the comparator group.^21^ rRMTL values below 1 indicate less time lived with the outcome in the low-dose methotrexate group, whereas values above 1 indicate more time lived with the outcome. CIs for rRMTL were calculated using a parametric approach defined within the survRM2 package. Results were expressed both as ratios and as absolute time gained (converted to mean days without outcome) by dividing the difference in rRMTL by the cumulative incidence in the comparator group. For each cohort, the absolute 5-year cumulative incidence of each outcome (with 95% confidence intervals) was estimated from the Kaplan–Meier survival function (one minus the survival probability at 5 years) and is reported in the text alongside rRMTL measures.

Statistical significance was set at two-sided Bonferroni adjusted critical p values. We prespecified families of tests and applied Bonferroni corrected critical values of p within each family. Primary analyses (psychosis *vs* three NSAIDs) used α = 0.05/3 = 0.0167 and the same threshold was applied individually for the secondary outcomes bipolar disorder, depression, and anxiety. Comparisons between low-dose methotrexate *vs* other DMARDs used α = 0.05/44 = 0.00114 for 11 drugs × 4 outcomes. Uncorrected p values are shown throughout the text; statistical significance attributed when p is less than the Bonferroni adjusted critical value is indicated in bold in the tables and asterisks in the figures. As further sensitivity analysis, we applied two unified Bonferroni thresholds: α/14 = 0.00357, correcting across all 14 comparators within a single outcome, and α/55 = 0.000909, correcting across all 55 informative low-dose methotrexate-*vs*-comparator outcome tests in the study (upadacitinib × psychosis is suppressed for insufficient events). The analyses reported here were not pre-registered; the outcomes and comparators were specified a priori on the basis of a pre-existing mechanistic hypothesis^9^ rather than selected after inspection of the data.

All analyses were repeated stratified by sex (male, female). Sex-stratified analyses (male and female) doubled the number of tests; therefore, Bonferroni correction was applied (primary NSAIDs α = 0.05/6 = 0.0083; secondary DMARDs α = 0.05/88 = 0.00056).

To assess whether baseline co-medication differences could explain the primary findings (outcome: psychosis; comparators: NSAIDs), we performed sensitivity analyses. These cohorts were constructed as follows: *i)* the same eligibility criteria and the same propensity-score matching strategy as the primary psychosis cohort; *ii)* the additional application of symmetric baseline medication exclusions across exposure groups, whereby individuals with prior or concurrent exposure to anti-rheumatic drugs (NSAIDs and/or DMARDs) other than the index treatment were excluded from both the low-dose methotrexate and NSAID comparator cohorts.

We note that our comparator cohorts received active treatments (NSAIDs or DMARDs) rather than no medication, a design chosen to minimise confounding by indication. As a falsification check for residual confounding and surveillance bias, we pre-specified a panel of negative-control outcomes (NCOs). NCOs are not expected to be affected by differences between choices of anti-rheumatic drugs but might be affected by differences in healthcare use and other unmeasured confounders. The NCOs were dog bite (W54.0), ingrowing nail (L60.0), and viral warts (B07), and between-group differences were tested for every drug comparator.

We also conducted analyses with all-cause death (recorded in the patient record on or after the index date) as the endpoint. A previous meta-analysis^25^ of 15 studies found low-dose methotrexate was associated with reduced mortality in rheumatoid arthritis, which would bias against finding fewer predicted recorded incidents of interest but nevertheless we carried out confirmatory analysis of mortality with the other treatments. The same paired propensity-matched cohorts and 5-year follow-up window used for the psychiatric outcomes were applied; rRMTL ratios are reported with 95% bootstrap confidence intervals. Because this is a single safety endpoint outside the multiplicity family of the psychiatric outcomes, a conventional p < 0.05 threshold (uncorrected) was applied.

### Role of the funding source

The funder had no role in the design and conduct of the study; collection, management, analysis, and interpretation of the data; preparation, review, or approval of the manuscript; and decision to submit the manuscript for publication.

## Results

After matching each low-dose methotrexate and comparator cohort, the sample size ranged from 1161 to 21,445 individuals, with a mean age ranging from 33.7 years (SD 8.7) to 37.4 years (6.9) depending on the comparison. Baseline characteristics after matching are summarised in Table 2, Table 3 and the Appendix (pp 9–24, 29–40).

**Table 2:** Baseline characteristics after propensity score matching (low-dose methotrexate *vs* NSAIDs).

Low-dose methotrexate *vs* comparator drug						
Characteristics	Low-dose methotrexate	Naproxen	Low-dose methotrexate	Diclofenac	Low-dose methotrexate	Celecoxib
Cohort size	12,447	12,447	12,916	12,916	8647	8647
Age at index, mean (SD)	34.0 (8.7)	33.8 (9.1)	35.9 (7.5)	35.9 (7.4)	36.3 (7.4)	36.1 (7.4)
Female	9788 (78.6%)	9770 (78.5%)	10,604 (82.1%)	10,582 (81.9%)	7021 (81.2%)	7024 (81.2%)
White	7905 (63.5%)	7090 (63.0%)	8374 (64.8%)	8301 (64.3%)	6304 (72.9%)	6179 (71.5%)
Problems related to education and literacy	12 (0.2%)	25 (0.2%)	24 (0.2%)	25 (0.2%)	18 (0.2%)	22 (0.3%)
Problems related to employment and unemployment	59 (0.5%)	62 (0.5%)	69 (0.5%)	80 (0.6%)	44 (0.5%)	56 (0.6%)
Diseases of the circulatory system	4485 (36.0%)	4392 (35.3%)	4498 (39.4%)	4521 (39.6%)	3678 (42.5%)	3613 (41.8%)
Metabolic disorders	3240 (26.0%)	3167 (25.4%)	3697 (28.6%)	3743 (29.0%)	2569 (29.7%)	2612 (30.2%)
Family history of mental and behavioural disorders	92 (0.7%)	94 (0.8%)	108 (0.8%)	115 (0.9%)	85 (1.0%)	99 (1.1%)
Corticosteroids	5627 (45.2%)	5403 (43.4%)	6543 (50.7%)	6403 (49.6%)	5140 (59.4%)	5058 (58.5%)

Cohort of individuals with rheumatoid arthritis who initiated pharmacological treatment at age ≤45 years (males and females). For each comparison, the left column corresponds to the low-dose methotrexate cohort, and the right column corresponds to the comparator cohort. SD, standard deviation; NSAIDs, non-steroidal anti-inflammatory drugs.

**Table 3:** Baseline characteristics after propensity score matching (low-dose methotrexate *vs* DMARDs).

Low-dose methotrexate *vs* comparator drug						
Characteristics	Low-dose methotrexate	Infliximab	Low-dose methotrexate	Adalimumab	Low-dose methotrexate	Etanercept
Cohort size	1226	1226	8915	8915	6240	6240
Age at index, mean (SD)	33.7 (8.7)	33.8 (8.3)	34.0 (8.5)	34.1 (8.3)	34.7 (8.1)	34.7 (8.1)
Female	901 (73.5%)	899 (73.3%)	6811 (76.4%)	6800 (76.3%)	4918 (78.8%)	4939 (79.2%)
White	880 (71.8%)	857 (69.9%)	6404 (71.8%)	6358 (71.3%)	4538 (72.7%)	4515 (72.4%)
Problems related to education and literacy	10 (0.8%)	10 (0.8%)	10 (0.1%)	16 (0.2%)	10 (0.2%)	10 (0.2%)
Problems related to employment and unemployment	10 (0.8%)	10 (0.8%)	45 (0.5%)	41 (0.5%)	13 (0.2%)	22 (0.4%)
Diseases of the circulatory system	415 (33.8%)	414 (33.8%)	2411 (27.0%)	2445 (27.4%)	1516 (24.3%)	1545 (24.8%)
Metabolic disorders	324 (26.4%)	336 (27.4%)	1651 (18.5%)	1755 (19.7%)	938 (15.0%)	974 (15.6%)
Family history of mental and behavioural disorders	16 (1.3%)	15 (1.2%)	30 (0.3%)	41 (0.5%)	15 (0.2%)	23 (0.4%)
Corticosteroids	552 (45.0%)	548 (44.7%)	3343 (37.5%)	3401 (38.1%)	1949 (31.2%)	1986 (31.8%)

Low-dose methotrexate *vs* comparator drug				
Characteristics	Low-dose methotrexate	Tocilizumab	Low-dose methotrexate	Hydroxychloroquine
Cohort size	1617	1617	21,445	21,445
Age at index, mean (SD)	34.6 (8.5)	34.3 (8.9)	35.1 (7.9)	35.1 (7.4)
Female	1395 (86.3%)	1379 (85.3%)	19,017 (88.7%)	18,993 (88.6%)
White	1103 (68.2%)	1087 (67.2%)	14,324 (66.8%)	14,198 (66.2%)
Problems related to education and literacy	10 (0.6%)	10 (0.6%)	36 (0.2%)	31 (0.1%)
Problems related to employment and unemployment	12 (0.7%)	10 (0.6%)	63 (0.3%)	64 (0.3%)
Diseases of the circulatory system	561 (34.7%)	563 (34.8%)	6692 (31.2%)	6720 (31.3%)
Metabolic disorders	378 (23.4%)	402 (24.9%)	3429 (19.0%)	3491 (19.4%)
Family history of mental and behavioural disorders	10 (0.6%)	10 (0.6%)	4236 (19.8%)	4265 (19.9%)
Corticosteroids	701 (43.4%)	695 (43.0%)	7548 (35.2%)	7507 (35.0%)

Cohort of individuals with rheumatoid arthritis who initiated pharmacological treatment at age ≤45 years (males and females). For each comparison, the left column corresponds to the low-dose methotrexate cohort, and the right column corresponds to the comparator cohort. Values are N (%) unless stated otherwise. Full baseline characteristics are provided in the Appendix. SD, standard deviation; DMARDs, Disease Modifying Anti-Rheumatic Drugs.

**Fig. 1:**
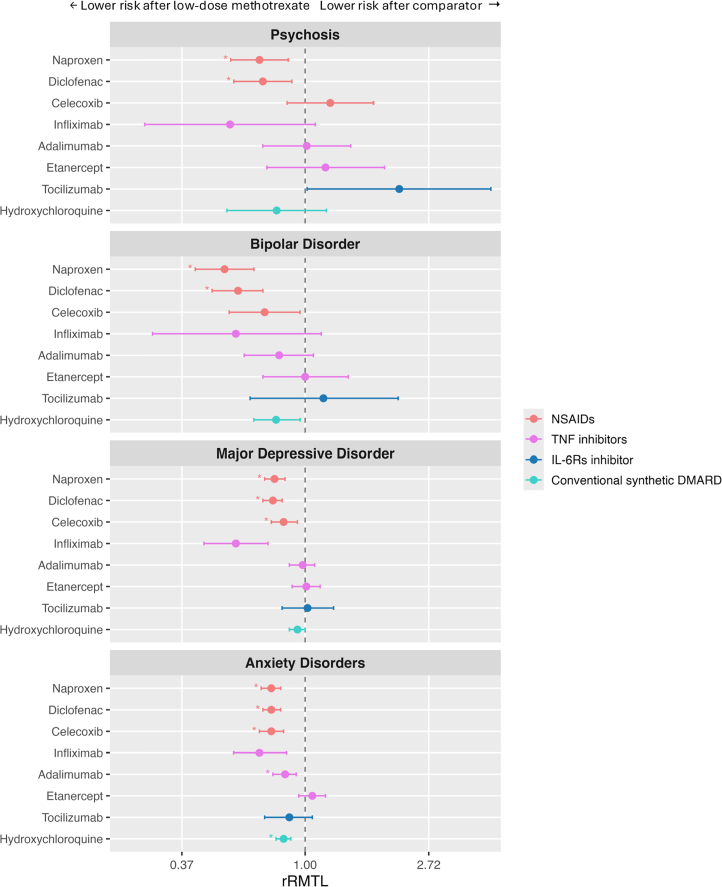
**Restricted mean time lost ratios (rRMTL) at 5 years after initiation of low-dose methotrexate versus comparator drugs (individuals aged ≤45 years).** Values are shown on the log scale with 95% confidence intervals (CIs); axis labels are back-transformed to rRMTL for interpretability. An rRMTL <1 favours low-dose methotrexate by indicating that the outcome was diagnosed more frequently or sooner after the comparator drug than after low-dose methotrexate, and an rRMTL >1 favours the comparator drug by indicating that the outcome was diagnosed more frequently or sooner after low-dose methotrexate than after the comparator drug. The vertical dashed line represents no difference (rRMTL 1). Colours indicate drug classes (non-steroidal anti-inflammatory drugs [NSAIDs]; tumour necrosis factor [TNF] inhibitors; interleukin-6 [IL-6] receptor inhibitor; conventional synthetic disease-modifying anti-rheumatic drug [DMARD]). ∗p value less than the Bonferroni-corrected critical value for multiple comparison: α = 0.05/3 = 0.0167 for the primary analyses (3 non-steroidal anti-inflammatory drugs, NSAIDs) and α = 0.05/44 = 0.0011 for the secondary analyses (4 outcomes × 11 disease-modifying anti-rheumatic drugs, DMARDs).

**Fig. 2:**
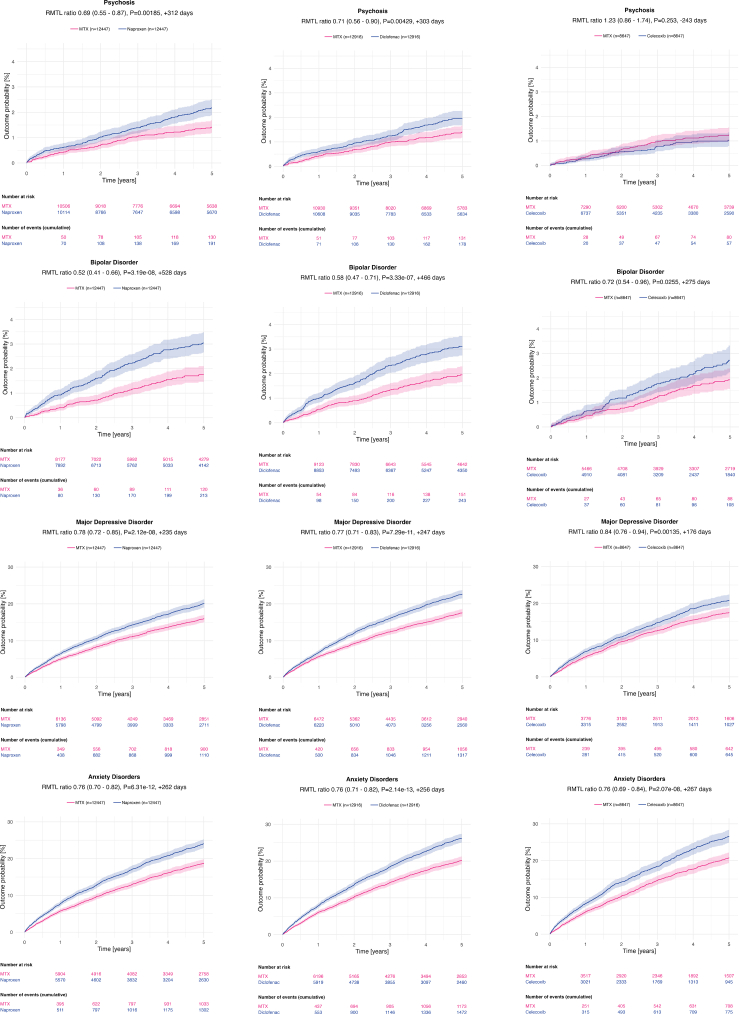
**Kaplan–Meier estimates of the cumulative incidence of psychosis, bipolar disorder, major depressive disorder, and anxiety disorders after initiation of low-dose methotrexate versusprimarycomparator drugs (naproxen, diclofenac, and celecoxib), in the cohort aged ≤45 years (males and females combined).** Curves show the outcome probability (cumulative incidence, %) over 5 years of follow-up. The low-dose methotrexate (pink) and comparator (blue) curves are shown with 95% confidence interval bands; legend values are matched cohort sizes after 1:1 propensity-score matching. Each panel reports the restricted mean time lost (RMTL) ratio (95% CI), the p value, and the estimated number of additional days free of the outcome over 5 years. An rRMTL <1 favours low-dose methotrexate by indicating that the outcome was diagnosed more frequently or sooner after the comparator drug than after low-dose methotrexate, and an rRMTL >1 favours the comparator drug by indicating that the outcome was diagnosed more frequently or sooner after low-dose methotrexate than after the comparator drug. Below each panel, the number of patients at risk and the cumulative number of events in each cohort are tabulated at yearly intervals (years 1–5). To allow valid visual comparison across drugs and outcomes, the y-axis scale is held constant within each outcome group (psychosis and bipolar disorder, 0–4.5%; major depressive disorder and anxiety disorders, 0–30%). The time axis is shown in years.

Results for the primary analyses comparing low-dose methotrexate with NSAIDs (naproxen, diclofenac, celecoxib) and secondary comparators biological DMARDs (infliximab, adalimumab, etanercept, tocilizumab) and a conventional synthetic DMARD (hydroxychloroquine) are shown in Table 4 and Fig. 1. The Kaplan–Meier curves are shown in Fig. 2 and the Appendix (pp 25–28).

**Table 4:** Restricted mean time lost ratio (rRMTL, 95% CI) for the risk of psychiatric disorders at 5 years follow-up after low-dose methotrexate *vs* comparators (age at index ≤45 years).

	Psychosis	Bipolar disorder	Major depressive disorder	Anxiety
Naproxen (*N* = 12,447)				
rRMTL (95% CI)	**0.69 (0.55**–**0.87)**	**0.52 (0.41**–**0.66)**	**0.78 (0.72**–**0.85)**	**0.76 (0.70**–**0.82)**
P value	**0.0019**	**p < 0.0001**	**p < 0.0001**	**p < 0.0001**
Events low-dose methotrexate	**130**	**120**	**900**	**1033**
Events Naproxen	**191**	**213**	**1110**	**1302**
Diclofenac (*N =* 12,916)				
rRMTL (95% CI)	**0.71 (0.56**–**0.90)**	**0.58 (0.47**–**0.71)**	**0.77 (0.71–0.83)**	**0.76 (0.71–0.82)**
P value	**0.0043**	**p < 0.0001**	**p < 0.0001**	**p < 0.0001**
Events low-dose methotrexate	**131**	**151**	**1056**	**1173**
Events Diclofenac	**178**	**243**	**1317**	**1472**
Celecoxib (*N =* 8647)				
rRMTL (95% CI)	1.23 (0.86–1.74)	0.72 (0.54–0.96)	**0.84 (0.76–0.94)**	**0.76 (0.69–0.84)**
P value	0.25	0.026	**0.0014**	**p < 0.0001**
Events low-dose methotrexate	80	88	**642**	**708**
Events Celecoxib	57	108	**645**	**775**
Infliximab (*N =* 1226)				
rRMTL (95% CI)	0.54 (0.27–1.09)	0.57 (0.29–1.14)	0.57 (0.44–0.74)	0.69 (0.56–0.86)
P value	0.085	0.11	0.0022	0.0052
Events low-dose methotrexate	12	14	82	126
Events Infliximab	23	21	133	166
Adalimumab (*N =* 8915)				
rRMTL (95% CI)	1.01 (0.71–1.45)	0.81 (0.61–1.07)	0.98 (0.88–1.08)	**0.85 (0.77–0.93)**
P value	0.94	0.15	0.63	**0.00046**
Events low-dose methotrexate	69	100	763	**834**
Events Adalimumab	63	111	732	**895**
Etanercept (*N =* 6240)				
rRMTL (95% CI)	1.18 (0.73–1.90)	1 (0.71–1.42)	1.01 (0.90–1.13)	1.06 (0.95–1.18)
P value	0.50	0.98	0.89	0.31
Events low-dose methotrexate	40	76	564	654
Events Etanercept	35	68	527	586
Tocilizumab (*N =* 1617)				
rRMTL (95% CI)	2.14 (1.02–4.50)	1.16 (0.64–2.12)	1.02 (0.83–1.26)	0.88 (0.72–1.06)
P value	0.045	0.62	0.84	0.18
Events low-dose methotrexate	24	26	168	177
Events Tocilizumab	10	18	138	170
Hydroxychloroquine (*N =* 21,445)				
rRMTL (95% CI)	0.79 (0.53–1.19)	0.79 (0.66–0.96)	0.94 (0.88–1.0)	**0.84 (0.79–0.89)**
P value	0.26	0.017	0.058	**p < 0.0001**
Events low-dose methotrexate	48	218	1692	**1901**
Events Hydroxychloroquine	56	248	1681	**2093**

Cohort of individuals with rheumatoid arthritis who initiated pharmacological treatment at age ≤45 years, (males and females). An rRMTL >1 indicates that people were diagnosed with the psychiatric outcome (psychosis, bipolar disorder, major depressive disorder, or anxiety disorders) more frequently or sooner after the comparator drug than after low-dose methotrexate, and conversely for rRMTL <1. Cohort sizes are after propensity score matching are indicated next comparator drug name. Bold colour indicates significant results less than Bonferroni corrected critical value for multiple comparison α = 0.05/3 = 0.0167 for the primary analyses (3 non-steroidal anti-inflammatory drugs), and α = 0.05/44 = 0.0011 for secondary analyses (4 outcomes × 11 drugs). rRMTL, restricted mean time lost ratio; CI, confidence interval.

For the primary outcome of psychosis and the primary drug comparators NSAIDs (Bonferroni threshold for multiple comparison α = 0.0167), the results showed that low-dose methotrexate was associated with lower risk of late onset psychosis at 5 years follow-up compared to naproxen (rRMTL 0.69, 95% CI 0.55–0.87; p = 0.0019), and diclofenac (0.71, 0.56–0.90; p = 0.0043), but not when compared with celecoxib (1.23, 0.86–1.74; p = 0.25). In absolute terms, the 5-year cumulative incidence of recorded psychosis was approximately 1.4% (95% CI 1.2–1.7) in the low-dose methotrexate cohorts, compared with 2.2% (1.9–2.5) for naproxen and 2.0% (1.7–2.3) for diclofenac; in the comparison with celecoxib, cumulative incidences were 1.2% (1.0–1.6) versus 1.0% (0.8–1.4).

For the secondary drug comparators (biological or conventional synthetic DMARDs) infliximab, adalimumab, etanercept, tocilizumab, and hydroxychloroquine (Bonferroni threshold for multiple comparison α = 0.0011), the risk of recorded incident psychosis did not differ between low-dose methotrexate and infliximab (rRMTL 0.54, 95% CI 0.27–1.09, p = 0.085), adalimumab (1.01, 0.71–1.45, p = 0.94), etanercept (1.18, 0.73–1.90, p = 0.50), tocilizumab (2.14, 1.02–4.50, p = 0.045) or hydroxychloroquine (0.79, 0.53–1.19, p = 0.26). Analyses were constrained by the low number of psychosis events at follow-up (range 10–191). In these analyses, the 5-year cumulative incidence of recorded psychosis was 0.3–1.6% across the low-dose methotrexate cohorts, compared with 0.4–2.2% for the comparator (biological and conventional synthetic) DMARDs.

Analyses evaluating exploratory drug comparators (leflunomide, sulfasalazine, minocycline, abatacept, tofacitinib and upadacitinib) showed no differences for psychosis events *vs* low-dose methotrexate after correction for multiple comparison (p > 0.0011 threshold for all; leflunomide p = 0.64, sulfasalazine p = 0.31, minocycline p = 0.25, abatacept p = 0.035, tofacitinib p = 0.084; Appendix pp 41–45, 103–104).

Secondary outcomes were bipolar disorder, major depressive disorder, and anxiety disorders. For bipolar disorder, analyses of the primary drug comparators NSAIDs (Bonferroni threshold for multiple comparison NSAIDs α = 0.0167; DMARDs α = 0.0011), showed that low-dose methotrexate was associated with lower risk of bipolar disorder at 5 years follow-up compared with naproxen (rRMTL 0.52, 95% CI 0.41–0.66, p < 0.0001) and diclofenac (0.58, 0.47–0.71, p < 0.0001) but not celecoxib (0.72, 0.54–0.96, p = 0.026). In absolute terms, the 5-year cumulative incidence of bipolar disorder was approximately 1.8–1.9% (95% CI 1.5–2.4) in the low-dose methotrexate cohorts, compared with 3.0% (2.7–3.5) for naproxen, 3.1% (2.7–3.5) for diclofenac, and 2.7% (2.2–3.3) for celecoxib.

For the secondary drug comparators infliximab, adalimumab, etanercept, tocilizumab, and hydroxychloroquine, we observed that, similar to psychosis, there was no difference in the recorded incidence of bipolar disorder when comparing low-dose methotrexate to DMARDs infliximab, adalimumab, etanercept, tocilizumab, or hydroxychloroquine (p > 0.0011 threshold for all; infliximab p = 0.11, adalimumab p = 0.15, etanercept p = 0.98, tocilizumab p = 0.62, or hydroxychloroquine p = 0.017). Similar to psychosis, analyses were constrained by the low number of events at follow-up (range 6–248).

There were no differences for bipolar disorder events when evaluating exploratory DMARDs drug comparators versus low-dose methotrexate after correction for multiple comparison (p > 0.0011 threshold for all; leflunomide p = 0.022, sulfasalazine p = 0.41, minocycline p = 0.73, abatacept p = 0.64, tofacitinib p = 0.77, and upadacitinib p = 0.50; Appendix pp, 41–45, 103–104).

For major depressive disorder, the number of events were substantially greater (range 77–1692) than for psychosis or bipolar disorder. Analyses of the primary drug comparators NSAIDs showed that low-dose methotrexate was associated with lower risk of depression at 5 years follow-up relative to all three NSAIDs: naproxen (rRMTL 0.78, 95% CI 0.72–0.85, p < 0.0001), diclofenac (0.77, 0.71–0.83, p < 0.0001), and celecoxib (0.84, 0.76–0.94, p = 0.0014). In absolute terms, the 5-year cumulative incidence of depression in the low-dose methotrexate cohorts ranged from 16.0% to 17.6%, compared with 20.1% (95% CI 19.1–21.2) for naproxen, 22.6% (21.5–23.8) for diclofenac, and 20.8% (19.3–22.4) for celecoxib.

When compared with secondary DMARDs drug comparators, low-dose methotrexate was not associated with reduced risk of depression (p > 0.0011 threshold for all; infliximab p = 0.0022, adalimumab p = 0.63, etanercept p = 0.89, tocilizumab p = 0.84, and hydroxychloroquine p = 0.058).

There were no differences for depression events when evaluating exploratory DMARDs drug comparators *vs* low-dose methotrexate after correction for multiple comparison (p > 0.0011 threshold for all; leflunomide p = 0.0024, sulfasalazine p = 0.0026, minocycline p = 0.0078, abatacept p = 0.69, tofacitinib p = 0.70, and upadacitinib p = 0.31; Appendix pp, 41–45, 103–104).

For anxiety disorders, the number of events were substantially greater (range 84–2093) than for psychosis or bipolar disorder. Analyses of the primary drug comparators NSAIDs showed that, similar to depression, low-dose methotrexate was associated with lower risk of anxiety disorders at 5 years follow-up compared to all three NSAIDs: naproxen (rRMTL 0.76, 95% CI 0.70–0.82, p < 0.0001), diclofenac (0.76, 0.71–0.82, p < 0.0001), and celecoxib (0.76, 0.69–0.84, p < 0.0001). In absolute terms, the 5-year cumulative incidence of anxiety disorders in the low-dose methotrexate cohorts ranged from 18.8% to 20.8%, compared with 24.0% (95% CI 22.9–25.3) for naproxen, 26.2% (25.0–27.4) for diclofenac, and 26.6% (24.9–28.4) for celecoxib.

When compared with secondary drug comparators infliximab, adalimumab, etanercept, tocilizumab, and hydroxychloroquine, we observed that low-dose methotrexate was associated with reduced risk for anxiety disorders when compared with adalimumab (rRMTL 0.85, 95% CI 0.78–0.93, p = 0.00046; 5-year cumulative incidence 17.8% [95% CI 16.7–19.0] vs 21.4% [20.1–22.7]) and hydroxychloroquine (rRMTL 0.84, 95% CI 0.79–0.89, p < 0.0001; 5-year cumulative incidence 18.5% [95% CI 17.8–19.3] vs 21.3% [20.5–22.2]). There was no difference between low-dose methotrexate and other biological DMARDs (p > 0.0011 threshold for all; infliximab p = 0.0052, etanercept p = 0.31, or tocilizumab p = 0.18).

When compared with exploratory DMARDs drug comparators, we observed that low-dose methotrexate was associated with lower risk versus sulfasalazine only (rRMTL 0.62, 95% CI 0.48–0.80; p = 0.00025). No differences were found between low-dose methotrexate and leflunomide (p = 0.0052), minocycline (p = 0.121), abatacept (p = 0.058), tofacitinib (p = 0.13), and upadacitinib (p = 0.035; p > 0.0011 threshold for all; Appendix pp 41–45, 103–104).

Sensitivity analyses showed that under the unified Bonferroni thresholds (α/14 and α/55), the previous significant associations observed for psychosis (low-dose methotrexate *vs* the NSAIDs naproxen and diclofenac) survived at α/14, but not at the stringent α/55, although the direction and magnitude were overall unchanged. For the secondary outcomes bipolar disorder, depression, and anxiety disorders, the previous significant associations when compared with NSAIDs continued to survive, and most of the associations also survived when compared with DMARDs. These results are reported in the Appendix (pp 105–106). Sensitivity analyses for the primary and secondary outcomes showed no significant associations between treatment groups and the NCOs composite (Appendix pp 111).

To test whether baseline co-medication differences—rather than low-dose methotrexate itself—could account for the lower number of recorded psychosis events versus naproxen and diclofenac, we repeated these comparisons after applying symmetric baseline co-medication exclusions. Findings remained directionally consistent for psychosis (and across all secondary psychiatric outcomes), and negative-control analyses suggested no evidence of residual confounding (Appendix pp 114–117).

All-cause mortality was low across all cohorts and generally lower in the low-dose methotrexate group (rRMTL range 0.32–0.79): the 5-year cumulative incidence of death in the low-dose methotrexate cohorts was 1.0–1.5%, versus 1.7–2.1% for the NSAIDs and 0.9–2.7% for the DMARDs.

Detailed sex-stratified data for all primary and secondary outcomes are provided in the Appendix (pp 46–102, 105–108). Results were broadly replicated in sex-stratified analyses, but particularly in females for some comparators and with varying associations reaching statistical significance after Bonferroni correction. For example, among NSAIDs, low-dose methotrexate generally demonstrated a lower recorded incidence of psychosis, bipolar disorder, depression, and anxiety in females compared with naproxen and diclofenac. Low-dose methotrexate showed no potential protective association when compared with other DMARDs for any of the outcomes except for anxiety disorders. Specifically, low-dose methotrexate was associated with a lower risk of anxiety when compared to hydroxychloroquine (significant in females; trend in males) and adalimumab (significant in males only).

**Fig. 3:**
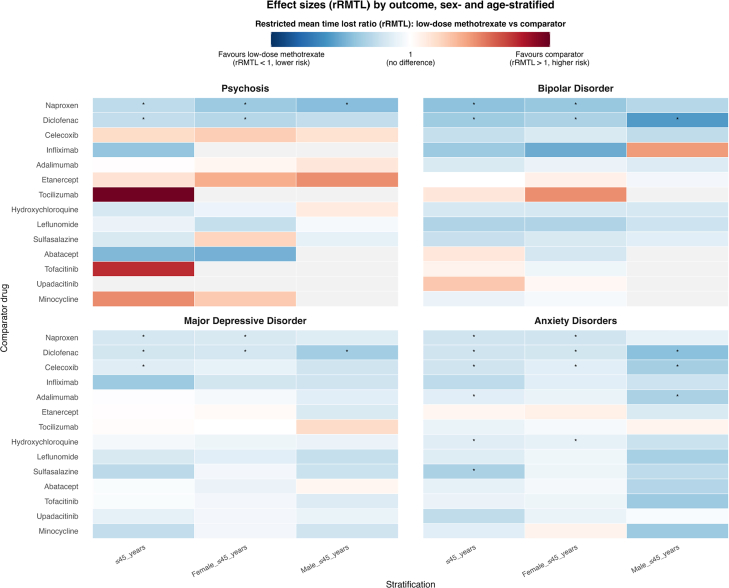
**Heatmap of restricted mean time lost ratios (rRMTL) for psychiatric outcomes at 5 years after initiation of low-dose methotrexate versus comparator drugs, stratified by age and sex.** The rRMTL compares the time lost to each outcome with low-dose methotrexate relative to the comparator: a value < 1 (blue) indicates lower risk with low-dose methotrexate *vs* the comparator drug, a value > 1 (red) indicates higher risk with low-dose methotrexate *vs* the comparator drug, and 1 (white) indicates no difference between groups; colour intensity reflects the magnitude of the deviation from 1. Boxes are light grey where ≤5 events occurred in either treatment group (effect estimates not calculated). Outcomes are displayed in separate panels. Groups: ≤45 years refers to individuals who initiated treatment for rheumatoid arthritis at ≤45 years, with estimates calculated with both sexes combined; Female_≤45_years and Male_≤45_years represent estimates stratified by sex and age at treatment initiation. ∗p value less than the Bonferroni-corrected critical value for multiple comparisons: α = 0.05/3 = 0.0167 for the primary analyses (3 non-steroidal anti-inflammatory drugs, NSAIDs) and α = 0.0544 = 0.0011 for the secondary analyses (4 outcomes × 11 disease-modifying anti-rheumatic drugs, DMARDs); for the sex-stratified analyses, α = 0.05/6 = 0.0083 for the primary analyses (NSAIDs) and α = 0.05/88 = 0.00057 for the secondary analyses (DMARDs).

The heatmap (Fig. 3) shows a summary of all comparisons, both unstratified and sex stratified.

## Discussion

We previously proposed that the reported antipsychotic effect of low-dose methotrexate is potentially mediated by its promotion of Treg control of systemic immunity as in rheumatoid arthritis and of neuro-glial dysregulation in psychosis. In this real-world cohort of individuals with rheumatoid arthritis, we tested the prediction that initiation of low-dose methotrexate therapy would be associated with lower risk of subsequent late onset psychosis compared with initiation of NSAIDs—positive control treatments that do not enhance Treg function. Indeed, we observed a reduced recorded incidence of late onset psychosis after low-dose methotrexate by comparison with the two non-selective COX-1 and COX-2 inhibitors, naproxen and diclofenac but not *vs* celecoxib, a selective COX-2 inhibitor. Consistent with our hypothesis, no significant difference in the recorded incidence of psychosis was detected between those treated with low-dose methotrexate and Treg-enhancing biological DMARDs (IL-6Rs and TNF inhibitors), although these comparisons are event-limited and should be regarded as inconclusive. However, we did not expect potential preventive effects of low-dose methotrexate versus NSAIDS (including versus celecoxib) to be more salient in deferring the onset of non-psychotic disorders (mood and anxiety disorders).

In comparing rRMTL magnitudes across psychiatric endpoints, it is important to bear in mind the influence of the size of the cohorts and the number of events. For older treatments (e.g., methotrexate *vs* naproxen, diclofenac and hydroxychloroquine) events were compared in samples of over 10,000 whereas the methotrexate *vs* infliximab and tocilizumab cohorts numbered less than 1700 with intermediate numbers for methotrexate versus celecoxib, adalimumab and etanercept (6–9000). The number of events varied considerably too, reflecting the far lower lifetime risk of psychosis and bipolar disorder in the community (∼1–2%) compared to depression and anxiety (∼25%).^17^ About 1–2% of each cohort developed psychosis whereas about 20–25% developed an anxiety disorder. The rarity of psychosis and bipolar events is exacerbated by the fact that at 35 years (the approximate age of treatment initiation), almost all the risk of psychosis has passed but about 40% of depression risk remains. Consequently, psychosis events were highly infrequent with approximately 1/8th of the number of events of depression or of anxiety giving rise to large confidence intervals. The null findings for the biological DMARD comparisons, particularly for infliximab and tocilizumab in which event counts were the lowest, should therefore be interpreted as the absence of strong evidence for a difference rather than as evidence of equivalence or non-inferiority, and adequately powered comparisons with biological DMARDs remain a priority for future replication.

The primary prediction of the low-dose methotrexate Treg hypothesis was partially upheld by its clear superiority to non-selective COX-1&2 NSAIDs in reducing events of psychosis. However, two findings prevent definitive conclusions. First, low-dose methotrexate did not outperform the selective COX-2 antagonist celecoxib. One explanation might be that celecoxib has some antipsychotic efficacy as claimed in some small studies.^26^ However, it would be difficult to attribute this to COX-2 inhibition since the non-selective inhibitors, especially diclofenac, are effective COX-2 antagonists. Celecoxib does achieve measurable concentrations in human cerebrospinal fluid, in contrast to several non-selective NSAIDs that show limited central nervous system penetration.^27^ Additionally, celecoxib has COX- actions including activation of peroxisome proliferator-activated receptor-γ^28^ and engagement of additional non-COX-2 targets relevant to glial activation,^29^ any of which could plausibly contribute to a central effect distinct from cyclo-oxygenase inhibition. Second, in the non-psychotic disorder cohorts, low-dose methotrexate was again superior to NSAIDs but not versus the other DMARDS. Therefore, the null findings observed when comparing low-dose methotrexate to celecoxib in psychosis should be interpreted as *“absence of strong evidence for a difference”* rather than as evidence of equivalence. The major difference between DMARDS and NSAIDS in rheumatoid arthritis is that the DMARDS improve the physical aspects of the illness by, for example, restoring Tregs and reducing inflammation. This general restoration of immune control, which does not occur with NSAIDs,^19^ may correct one or more inflammatory pathways to the onset of psychotic and non-psychotic illness.

The benefit of low-dose methotrexate over NSAIDs across disorders has biologically plausibility in light of mounting evidence implicating inflammation broadly in the pathogenesis of many psychiatric disorders.^1^ Low-dose methotrexate is known to increase extracellular adenosine, a potent anti-inflammatory mediator that promotes the expansion of blood Tregs.^5^^,^^6^ Low-dose methotrexate has also been shown to restore deficient Treg function by reversing aberrant epigenetic silencing of the *FOXP3* gene that is essential for Treg survival and activity.^7^ Flow cytometry studies have reported associations between lower blood Treg counts and symptoms that cut-across diagnostic boundaries, particularly negative, depressive, and cognitive symptoms in psychiatric conditions, including psychosis, bipolar disorder, and depression.^30^ Although low-dose methotrexate does not cross the blood brain barrier,^31^ a small population of circulating Tregs infiltrate the human brain where they regulate glial function to promote brain homoeostasis.^32^

In secondary analyses, there was no difference between low-dose methotrexate and biological DMARDs IL-6 (e.g., tocilizumab) and TNF (e.g., infliximab, adalimumab, etanercept) inhibitors for psychosis event, in line with our hypothesis. Like low-dose methotrexate, IL-6 and TNF inhibitors can boost Tregs in blood.^30^ We explored comparisons with other DMARDs but with their uncertain effects on Tregs, we simply record that they were also not less effective than low-dose methotrexate. Among secondary psychiatric outcomes and other DMARDs, significant results were observed for anxiety disorders only; low-dose methotrexate was associated with lower risk of anxiety disorders *vs* adalimumab which was not a tendency noted in the other disorders. In contrast, a significant benefit over hydroxychloroquine and sulfasalazine for anxiety is also nominally statistically significant for the other non-psychotic disorders (Table 2) and therefore not specific to anxiety.

The association between low-dose methotrexate and lower risk of psychiatric outcomes over NSAIDs was overall replicated in sex-stratified analyses, but particularly in females for some comparators, likely reflecting the greater incidence of rheumatoid arthritis in women.^33^

It is important to note that our findings reflect a potential preventive effect—i.e., delaying or averting the first episode of psychosis in individuals without prior psychotic illness—rather than a therapeutic effect on established symptoms. The biological mechanisms required to prevent disease onset may differ from those required to alleviate acute symptoms in psychosis, such as those explored in the prior add-on clinical trial of low-dose methotrexate in early-stage schizophrenia.^3^

As with any pharmacoepidemiological study, this study is prone to indication bias. Because low-dose methotrexate is typically used as a background first-line DMARD in rheumatoid arthritis, with NSAIDs and other DMARDs added according to symptom control or disease activity,^4^ we did not exclude previous or concurrent NSAID or other DMARD use from the low-dose methotrexate cohort in our primary analyses. This approach was adopted to capture a clinically representative population of patients receiving low-dose methotrexate for rheumatoid arthritis, thereby maximising the relevance of the findings to routine clinical practice. The analyses should therefore be interpreted as active-comparator treatment-strategy comparisons rather than strict monotherapy contrasts, and residual confounding by disease severity or co-medication cannot be fully excluded. Reassuringly, sensitivity analyses applying symmetric baseline co-medication exclusions showed that the primary findings remained directionally consistent.

It is unlikely that the potential preventive effect of low-dose methotrexate over NSAIDs for the incident recorded event of psychiatric outcomes reflects differences in rheumatoid arthritis severity. In routine clinical practice, NSAIDs are typically used for early or milder symptoms, whereas escalation to low-dose methotrexate generally signals more persistent inflammation or more active disease.^4^ If milder rheumatoid arthritis disease severity was associated with lower psychiatric risk, this pattern would be expected to bias against our findings. However, we observed higher rates of psychiatric outcomes among individuals treated with NSAIDs who were unexposed to low-dose methotrexate, making confounding by disease severity an improbable explanation.

We further attempted to minimise confounding by disease severity through propensity score matching and by adjusting for baseline corticosteroid use, an approach used in previous studies^34^ which serves as a proxy for more severe inflammatory disease. Although some residual confounding is always possible in observational data, we pre-specified a panel of negative-control outcomes and tested between-group differences for every drug comparator. No significant differences were detected across the negative-control outcome panel, arguing against major residual confounding or generalised differences in healthcare use between cohorts.

The lower rates of recorded incident illness after low-dose methotrexate are not attributable to shorter survival since we observed decreases in all-cause mortality in the low-dose methotrexate group versus comparator treatments, which were consistent with the overall reduction (hazard ratio 0.59) reported in a meta-analysis of 15 studies.^25^ The meta-analysis identified specific protection against raised rates of cardiovascular and interstitial lung disease in rheumatoid arthritis but cause of death was not available in this network.

Our study has important limitations over and above the variation of sample and frequency of events. First, indication bias may affect comparisons with NSAIDs; since NSAIDs are often used for acute flares, the underlying inflammatory surge–rather than the lack of methotrexate–might drive the higher psychosis risk. Second, while EHR mis-recording is possible, it likely represents non-differential “noise” rather than directional bias. To mitigate under-recording of over the counter use, we excluded ibuprofen, though the over the counter availability of other NSAIDs like naproxen remains a factor. Furthermore, we could not assess medication adherence or exact treatment duration. Refined stratification by age and sex was also limited by low event frequencies, requiring replication in larger datasets. Mortality ascertainment in the TriNetX Network is incomplete: month and year of death are recorded but day is obfuscated for de-identification, and deaths occurring outside the network's contributing healthcare organisations are not captured unless linked to external death registries, so some deceased patients are likely misclassified as living.^35^ Nevertheless, it seems unlikely that under-capture of mortality would be influenced by the different treatments. Finally, these results apply to a rheumatoid arthritis population; repurposing low-dose methotrexate for clinical use outside of its current indication would require many further steps, particularly demonstrating the potential size of effect, efficacy, tolerability, and risk benefit; methotrexate even at low-dose has some significant side effects that would need careful consideration even for the potential benefit of a serious mental illness with very significant personal, family and societal costs.

In conclusion, our findings suggest potential psychiatric risk reduction associated with low-dose methotrexate compared to a range of NSAIDs, but not versus biological DMARDs. Given that extensive trials of other anti-inflammatories have yielded limited psychiatric benefit, this superior profile suggests a unique mechanism beyond simple inflammation suppression. We propose this benefit is driven by Treg-mediated systemic control of inflammation and central neuro-glial regulation. Ultimately, these results provide clinical reassurance that low-dose methotrexate is unlikely to increase psychiatric risk and highlights the potential of targeting regulatory immune pathways as a novel strategy for psychiatric prevention.

## Contributors

FC-Z, RUTG, BD, and MT developed the study methodology. FC-Z conducted the study analyses and drafted the manuscript, tables, and figures. BD and FC-Z wrote the Research in Context section. RUTG, BD, and MT supported the interpretation of the findings, and supervised the overall project. All authors critically revised the manuscript and approved the final version. All authors had full access to all the data in the study and accept responsibility to submit for publication. FCZ and MT verified the data, and both had access to the data.

## Data sharing statement

Aggregate data, as presented in this paper, is fully provided in the Appendix. Data presented in this article were acquired from TriNetX. This study had no special privileges. Eligibility criteria specified in the Methods and Supplementary Material would allow other researchers to identify similar cohorts of patients as used for our analyses; however, TriNetX is a live platform with new data being added daily so exact counts will vary. To gain access to the data, a request can be made to TriNetX (join@trinetx. com), but costs might be incurred, and a data sharing agreement would be necessary.

## Declaration of interests

We declare no competing interests.
