# Supplementary material for: Potential preventive role of low-dose methotrexate against incident recorded psychosis: a retrospective cohort study based on electronic health records
Source: eClinicalMedicine. 2026 Jul 23;98:104111. doi: 10.1016/j.eclinm.2026.104111 (PMC13427570; doi:10.1016/j.eclinm.2026.104111)
Supplement: Supplementary Tables and Figures [file mmc1.pdf]

# APPENDIX

# Potential preventive role of low-dose methotrexate against incident recorded psychosis: a retrospective cohort study based on electronic health records

Fabiana Corsi-Zuelli PhD[1*], Maxime Taquet PhD[1,2], Bill Deakin PhD[3],

Rachel Upthegrove PhD[1,4,5]

[1] Department of Psychiatry, University of Oxford, Warneford Hospital, Oxford, UK

[2] Oxford Health NHS Foundation Trust, Oxford, UK

[3] Division of Psychology and Mental Health, University of Manchester, Manchester, UK

[4] Institute for Mental Health, University of Birmingham, Birmingham, UK

[5] Early Intervention Service, Birmingham and Solihull Mental Health Foundation Trust

*** Corresponding author:**

Dr Fabiana Corsi-Zuelli, Department of Psychiatry, University of Oxford, Warneford Hospital, Oxford, OX3 7JX, United Kingdom. email: fabiana.corsi-zuelli@psych.ox.ac.uk

**Keywords:** Psychosis, Low-dose Methotrexate; Rheumatoid Arthritis; Major Depressive Disorder; Bipolar Disorder; Anxiety Disorders

## Supplementary Methods

### Cohorts

The analysis process includes two main steps: 1) Defining the cohorts through query criteria; 2) Setting up and running the analysis. Setting up the analysis requires definitions for the index event, outcomes criteria, and the time frame.

*Cohorts' definition*

This query was run on the network US Collaborative Network with 61 health care organisations (HCOs) queried and 61 HCO(s) responded.

## Supplementary Tables

## Supplementary Table S1. Summary of study cohorts

| | | | | | |
|---|---|---|---|---|---|
| **Group 1*** | | | | | |
| | **Group 1A Low-dose methotrexate** | | | | |
| | must have | | medication | NLM:RXNORM:6851 | methotrexate (at most 45 years old at event**; Route: Oral Product; Strength: 2 MG or 2.5 MG or 5 MG or 7.5 MG or 10 MG or 15 MG or 20 MG) |
| | event relationship | | The first instance of Group 1B occurred on or before the first instance of low-dose methotrexate | | |
| | **Group 1B Rheumatoid arthritis** | | | | |
| | must have | any of | diagnosis | UMLS:ICD10CM:M05 | Rheumatoid arthritis with rheumatoid factor |
| | | | diagnosis | UMLS:ICD10CM:M06 | Other rheumatoid arthritis |
| | cannot have | | diagnosis | UMLS:ICD10CM:F20-F29 | Schizophrenia, schizotypal, delusional, and other non-mood psychotic disorders |
| | | or | diagnosis | UMLS:ICD10CM:C00-C14 | Malignant neoplasms of lip, oral cavity and pharynx |
| | | or | diagnosis | UMLS:ICD10CM:C15-C26 | Malignant neoplasms of digestive organs |
| | | or | diagnosis | UMLS:ICD10CM:C30-C39 | Malignant neoplasms of respiratory and intrathoracic organs |
| | | or | diagnosis | UMLS:ICD10CM:C40-C41 | Malignant neoplasms of bone and articular cartilage |
| | | or | diagnosis | UMLS:ICD10CM:C43-C44 | Melanoma and other malignant neoplasms of skin |
| | | or | diagnosis | UMLS:ICD10CM:C45-C49 | Malignant neoplasms of mesothelial and soft tissue |
| | | or | diagnosis | UMLS:ICD10CM:C50-C50 | Malignant neoplasms of breast (C50) |
| | | or | diagnosis | UMLS:ICD10CM:C51-C58 | Malignant neoplasms of female genital organs |
| | | or | diagnosis | UMLS:ICD10CM:C60-C63 | Malignant neoplasms of male genital organs |
| | | or | diagnosis | UMLS:ICD10CM:C64-C68 | Malignant neoplasms of urinary tract |

| | | | | | |
|---|---|---|---|---|---|
| | | or | diagnosis | UMLS:ICD10CM:C69-C72 | Malignant neoplasms of eye, brain and other parts of central nervous system |
| | | or | diagnosis | UMLS:ICD10CM:C73-C75 | Malignant neoplasms of thyroid and other endocrine glands |
| | | or | diagnosis | UMLS:ICD10CM:C76-C80 | Malignant neoplasms of ill-defined, other secondary and unspecified sites |
| | | or | diagnosis | UMLS:ICD10CM:C7A-C7A | Malignant neuroendocrine tumors (C7A) |
| | | or | diagnosis | UMLS:ICD10CM:C7B-C7B | Secondary neuroendocrine tumors (C7B) |
| | | or | diagnosis | UMLS:ICD10CM:C81-C96 | Malignant neoplasms of lymphoid, hematopoietic and related tissue |

* For the comparator cohorts, low-dose methotrexate was replaced with 14 individual alternative drug treatments approved by the US Food and Drug Administration (FDA) for the treatment of rheumatoid arthritis. In these comparator cohorts, patients with prior exposure to low-dose methotrexate were excluded.

**Additional cohorts stratified by sex (male, female) were also built.

**Supplementary Table S2. Summary of comparator drugs**

| Comparator drug | TriNETx code |
|---|---|
| **Non-steroidal anti-inflammatory drugs** | |
| Naproxen | NLM:RXNORM:7258 |
| Diclofenac | NLM:RXNORM:3355 |
| Celecoxib | NLM:RXNORM:140587 |
| **Biological DMARDs** | |
| Infliximab | NLM:RXNORM:191831 |
| Adalimumab | NLM:RXNORM:327361 |
| Etanercept | NLM:RXNORM:214555 |
| Tocilizumab | NLM:RXNORM:612865 |
| Abatacept | NLM:RXNORM:614391 |
| **Conventional synthetic DMARDs** | |
| Hydroxychloroquine | NLM:RXNORM:5521 |
| Leflunomide | NLM:RXNORM:27169 |
| Sulfasalazine | NLM:RXNORM:9524 |
| Minocycline | NLM:RXNORM:6980 |
| **Targeted synthetic DMARDs** | |
| Tofacitinib | NLM:RXNORM:1357536 |
| Upadacitinib | NLM:RXNORM:2196092 |

DMARD: disease modifying antirheumatic drugs

**Supplementary Table S3. Summary of diagnostic outcomes**

| Diagnosis | TriNETx code | Definition |
|---|---|---|
| **Psychosis**<br>F20-29, F30.2, F31.2, F31.5, F32.3, F33.3 | UMLS:ICD10CM:F20-F29<br>UMLS:ICD10CM:F30.2<br>UMLS:ICD10CM:F31.2<br>UMLS:ICD10CM:F31.5<br>UMLS:ICD10CM:F32.3<br>UMLS:ICD10CM:F33.3 | Schizophrenia, schizotypal, delusional, and other non-mood psychotic disorders<br>Manic episode, severe with psychotic symptoms<br>Bipolar disorder, current episode manic, severe, with psychotic features<br>Bipolar disorder, current episode depressed, severe, with psychotic features<br>Major depressive disorder, single episode, severe, with psychotic features<br>Major depressive disorder, recurrent, severe, with psychotic symptoms |
| **Bipolar Disorder**<br>F31 | UMLS:ICD10CM:F31 | Bipolar disorder |
| F32, F33<br>Major Depressive Disorder | UMLS:ICD10CM:F32<br>UMLS:ICD10CM:F33 | Depressive episode<br>Major depressive disorder, recurrent |
| **Anxiety disorders** | | |
| F41 | UMLS:ICD10CM:F41 | Other anxiety disorders |
| F41.1 | UMLS:ICD10CM:F41.1 | Generalized anxiety disorder |
| F40 | UMLS:ICD10CM:F40 | Phobic anxiety disorders |
| F41.8 | UMLS:ICD10CM:F41.8 | Other specified anxiety disorders |
| F41.0 | UMLS:ICD10CM:F41.0 | Panic disorder [episodic paroxysmal anxiety] |
| F41.3 | UMLS:ICD10CM:F41.3 | Other mixed anxiety disorders |
| F41.9 | UMLS:ICD10CM:F41.9 | Anxiety disorder, unspecified |
| F42 | UMLS:ICD10CM:F42 | Obsessive-compulsive disorder |

**Supplementary Table S4. List of confounding**

| **Demographics** | |
|---|---|
| Code | Characteristic |
| AI | Age at Index |
| F | Female |
| 2054-5 | Black or African American |
| M | Male |
| 2106-3 | White |
| 1002-5 | American Indian or Alaska Native |
| UNK | Unknown Race |
| 2076-8 | Native Hawaiian or Other Pacific Islander |
| UN | Unknown Ethnicity |
| 2186-5 | Not Hispanic or Latino |
| 2135-2 | Hispanic or Latino |
| 2131-1 | Other Race |
| 2028-9 | Asian |
| **Diagnosis*** | |
| Z55 | Problems related to education and literacy |
| Z56 | Problems related to employment and unemployment |
| Z81 | Family history of mental and behavioral disorders |
| E70-E88 | Metabolic disorders |
| F31 | Bipolar disorder |
| F32 | Depressive episode |
| F33 | Major depressive disorder, recurrent |
| X71 | Intentional self-harm by drowning and submersion |
| R45.851 | Suicidal ideations |
| T14.91 | Suicide attempt |
| X71-X83 | Intentional self-harm |
| X71 | Intentional self-harm by drowning and submersion |
| R45.851 | Suicidal ideations |
| T14.91 | Suicide attempt |
| X71-X83 | Intentional self-harm |
| W54.0 | Bitten by dog** |
| L60.0 | Ingrowing nail** |
| B07 | Viral warts** |
| I00-I99 | Diseases of the circulatory system |
| F41 | Other anxiety disorders |

| | |
|---|---|
| F41.1 | Generalized anxiety disorder |
| F40 | Phobic anxiety disorders |
| F41.8 | Other specified anxiety disorders |
| F41.0 | Panic disorder [episodic paroxysmal anxiety] |
| F41.3 | Other mixed anxiety disorders |
| F41.9 | Anxiety disorder, unspecified |
| F42 | Obsessive-compulsive disorder |
| F30.2 | Manic episode, severe with psychotic symptoms |
| F31.2 | Major depressive disorder, single episode, severe with psychotic features |
| F31.5 | Major depressive disorder, recurrent, severe with psychotic symptoms |
| F32.3 | Bipolar disorder, current episode manic severe with psychotic features |
| F33.3 | Bipolar disorder, current episode depressed, severe, with psychotic features |
| **Medication** | |
| R01AD | Corticosteroids |

* Diagnostic exclusions for psychiatric disorders were outcome-specific

** Negative control outcomes

## Supplementary Table S5. Baseline characteristics after propensity score matching low-dose methotrexate *vs* Naproxen

| Cohort 1. Low-dose methotrexate (N = 12,447) and cohort 2. Naproxen (N = 12,447) | | | | | | | |
|---|---|---|---|---|---|---|---|
| **Demographics** | | | | | | | |
| Cohort | | | Mean ± standard deviation, SD | Patients | % of Cohort | P-Value | Std diff. |
| 1<br>2 | AI | Age at Index | 34.0 +/- 8.7<br>33.8 +/- 9.1 | 12,447<br>12,447 | 100%<br>100% | 0.111 | 0.020 |
| 1<br>2 | F | Female | | 9,788<br>9,770 | 78.6%<br>78.5% | 0.781 | 0.004 |
| 1<br>2 | 2054-5 | Black or African American | | 2,320<br>2,247 | 18.6%<br>18.1% | 0.232 | 0.015 |
| 1<br>2 | M | Male | | 2,655<br>2,673 | 21.3%<br>21.5% | 0.781 | 0.004 |
| 1<br>2 | 2106-3 | White | | 7,905<br>7,909 | 63.5%<br>63.5% | 0.958 | 0.001 |
| 1<br>2 | 1002-5 | American Indian or Alaska Native | | 109<br>116 | 0.9%<br>0.9% | 0.639 | 0.006 |
| 1<br>2 | UNK | Unknown Race | | 837<br>887 | 6.7%<br>7.1% | 0.212 | 0.016 |
| 1<br>2 | 2076-8 | Native Hawaiian or Other Pacific Islander | | 98<br>105 | 0.8%<br>0.8% | 0.622 | 0.006 |
| 1<br>2 | UN | Unknown Ethnicity | | 1,808<br>1,831 | 14.5%<br>14.7% | 0.680 | 0.005 |
| 1<br>2 | 2186-5 | Not Hispanic or Latino | | 8,943<br>8,879 | 71.8%<br>71.3% | 0.368 | 0.011 |
| 1<br>2 | 2135-2 | Hispanic or Latino | | 1,696<br>1,737 | 13.6%<br>14.0% | 0.451 | 0.010 |
| 1<br>2 | 2131-1 | Other Race | | 737<br>741 | 5.9%<br>6.0% | 0.915 | 0.001 |
| 1<br>2 | 2028-9 | Asian | | 441<br>442 | 3.5%<br>3.6% | 0.973 | <0.001 |
| **Diagnosis** | | | | | | | |
| Cohort | | | Mean ± SD | Patients | % of Cohort | P-Value | Std diff. |
| 1<br>2 | Z55 | Problems related to education and literacy | | 21<br>25 | 0.2%<br>0.2% | 0.555 | 0.007 |
| 1<br>2 | Z56 | Problems related to employment and unemployment | | 59<br>62 | 0.5%<br>0.5% | 0.785 | 0.003 |
| 1<br>2 | Z81 | Family history of mental and behavioral disorders | | 92<br>94 | 0.7%<br>0.8% | 0.883 | 0.002 |
| 1<br>2 | E70-E88 | Metabolic disorders | | 3,240<br>3,167 | 26.0%<br>25.4% | 0.290 | 0.013 |
| 1<br>2 | F31 | Bipolar disorder | | 563<br>603 | 4.5%<br>4.8% | 0.230 | 0.015 |
| 1<br>2 | F32 | Depressive episode | | 2,797<br>2,848 | 22.5%<br>22.9% | 0.440 | 0.010 |
| 1<br>2 | F33 | Major depressive disorder, recurrent | | 737<br>783 | 5.9%<br>6.3% | 0.223 | 0.015 |

| | | | | | | | |
|---|---|---|---|---|---|---|---|
| 1<br>2 | X71 | Intentional self-harm by drowning and submersion | | 0<br>0 | 0%<br>0% | -- | -- |
| 1<br>2 | R45.851 | Suicidal ideations | | 273<br>302 | 2.2%<br>2.4% | 0.221 | 0.016 |
| 1<br>2 | T14.91 | Suicide attempt | | 16<br>20 | 0.1%<br>0.2% | 0.505 | 0.008 |
| 1<br>2 | X71-X83 | Intentional self-harm | | 25<br>27 | 0.2%<br>0.2% | 0.781 | 0.004 |
| 1<br>2 | W54.0 | Bitten by dog | | 43<br>44 | 0.3%<br>0.4% | 0.914 | 0.001 |
| 1<br>2 | L60.0 | Ingrowing nail | | 134<br>124 | 1.1%<br>1.0% | 0.531 | 0.008 |
| 1<br>2 | B07 | Viral warts | | 231<br>252 | 1.9%<br>2.0% | 0.335 | 0.012 |
| 1<br>2 | I00-I99 | Diseases of the circulatory system | | 4,485<br>4,392 | 36.0%<br>35.3% | 0.218 | 0.016 |
| 1<br>2 | F41 | Other anxiety disorders | | 3,276<br>3,371 | 26.3%<br>27.1% | 0.174 | 0.017 |
| 1<br>2 | F41.1 | Generalized anxiety disorder | | 1,028<br>1,124 | 8.3%<br>9.0% | 0.030 | 0.027 |
| 1<br>2 | F40 | Phobic anxiety disorders | | 135<br>155 | 1.1%<br>1.2% | 0.237 | 0.015 |
| 1<br>2 | F41.8 | Other specified anxiety disorders | | 475<br>530 | 3.8%<br>4.3% | 0.077 | 0.022 |
| 1<br>2 | F41.0 | Panic disorder [episodic paroxysmal anxiety] | | 394<br>443 | 3.2%<br>3.6% | 0.085 | 0.022 |
| 1<br>2 | F41.3 | Other mixed anxiety disorders | | 10<br>10 | 0.1%<br>0.1% | 1 | <0.001 |
| 1<br>2 | F41.9 | Anxiety disorder, unspecified | | 2,750<br>2,778 | 22.1%<br>22.3% | 0.669 | 0.005 |
| 1<br>2 | F42 | Obsessive-compulsive disorder | | 107<br>121 | 0.9%<br>1.0% | 0.352 | 0.012 |
| 1<br>2 | F30.2 | Manic episode, severe with psychotic symptoms | | 0<br>0 | 0%<br>0% | -- | -- |
| 1<br>2 | F31.2 | Bipolar disorder, current episode manic severe with psychotic features | | 10<br>10 | 0.1%<br>0.1% | 1 | <0.001 |
| 1<br>2 | F31.5 | Bipolar disorder, current episode depressed, severe, with psychotic features | | 10<br>10 | 0.1%<br>0.1% | 1 | <0.001 |
| 1<br>2 | F32.3 | Major depressive disorder, single episode, severe with psychotic features | | 12<br>13 | 0.1%<br>0.1% | 0.841 | 0.003 |
| 1<br>2 | F33.3 | Major depressive disorder, recurrent, severe with psychotic symptoms | | 18<br>16 | 0.1%<br>0.1% | 0.731 | 0.004 |
| **Medication** | | | | | | | |
| Cohort | | | Mean ± SD | Patients | % of Cohort | P-Value | Std diff. |
| 1<br>2 | R01AD | Corticosteroids | | 5,627<br>5,403 | 45.2%<br>43.4% | 0.004 | 0.036 |

## Supplementary Table S6. Baseline characteristics after propensity score matching low-dose methotrexate *vs* Diclofenac

**Cohort 1. Low-dose methotrexate (N = 12,916) and cohort 2. Diclofenac (N = 12,916)**

**Demographics**

| Cohort | | | Mean ± SD | Patients | % of Cohort | P-Value | Std diff. |
|---|---|---|---|---|---|---|---|
| 1<br>2 | AI | Age at Index | 35.9 +/- 7.5<br>35.9 +/- 7.4 | 12,916<br>12,916 | 100%<br>100% | 0.785 | 0.003 |
| 1<br>2 | F | Female | | 10,604<br>10,582 | 82.1%<br>81.9% | 0.722 | 0.004 |
| 1<br>2 | 2054-5 | Black or African American | | 2,246<br>2,229 | 17.4%<br>17.3% | 0.780 | 0.003 |
| 1<br>2 | M | Male | | 2,312<br>2,334 | 17.9%<br>18.1% | 0.722 | 0.004 |
| 1<br>2 | 2106-3 | White | | 8,374<br>8,301 | 64.8%<br>64.3% | 0.342 | 0.012 |
| 1<br>2 | 1002-5 | American Indian or Alaska Native | | 135<br>131 | 1.0%<br>1.0% | 0.805 | 0.003 |
| 1<br>2 | UNK | Unknown Race | | 1,037<br>1,087 | 8.0%<br>8.4% | 0.257 | 0.014 |
| 1<br>2 | 2076-8 | Native Hawaiian or Other Pacific Islander | | 62<br>61 | 0.5%<br>0.5% | 0.928 | 0.001 |
| 1<br>2 | UN | Unknown Ethnicity | | 1,878<br>1,917 | 14.5%<br>14.8% | 0.493 | 0.009 |
| 1<br>2 | 2186-5 | Not Hispanic or Latino | | 9,423<br>9,244 | 73.0%<br>71.6% | 0.013 | 0.031 |
| 1<br>2 | 2135-2 | Hispanic or Latino | | 1,615<br>1,755 | 12.5%<br>13.6% | 0.010 | 0.032 |
| 1<br>2 | 2131-1 | Other Race | | 710<br>755 | 5.5%<br>5.8% | 0.226 | 0.015 |
| 1<br>2 | 2028-9 | Asian | | 352<br>352 | 2.7%<br>2.7% | 1 | <0.001 |

**Diagnosis**

| Cohort | | | Mean ± SD | Patients | % of Cohort | P-Value | Std diff. |
|---|---|---|---|---|---|---|---|
| 1<br>2 | Z55 | Problems related to education and literacy | | 24<br>25 | 0.2%<br>0.2% | 0.886 | 0.002 |
| 1<br>2 | Z56 | Problems related to employment and unemployment | | 69<br>80 | 0.5%<br>0.6% | 0.366 | 0.011 |
| 1<br>2 | Z81 | Family history of mental and behavioral disorders | | 108<br>115 | 0.8%<br>0.9% | 0.638 | 0.006 |
| 1<br>2 | E70-E88 | Metabolic disorders | | 3,697<br>3,743 | 28.6%<br>29.0% | 0.527 | 0.008 |
| 1<br>2 | F31 | Bipolar disorder | | 603<br>623 | 4.7%<br>4.8% | 0.558 | 0.007 |
| 1<br>2 | F32 | Depressive episode | | 3,390<br>3,450 | 26.2%<br>26.7% | 0.398 | 0.011 |
| 1<br>2 | F33 | Major depressive disorder, recurrent | | 942<br>1,005 | 7.3%<br>7.8% | 0.138 | 0.018 |
| 1<br>2 | X71 | Intentional self-harm by drowning and submersion | | 0<br>0 | 0%<br>0% | -- | -- |

| | | | | | | | |
|---|---|---|---|---|---|---|---|
| 1<br>2 | R45.851 | Suicidal ideations | | 265<br>290 | 2.1%<br>2.2% | 0.283 | 0.013 |
| 1<br>2 | T14.91 | Suicide attempt | | 21<br>22 | 0.2%<br>0.2% | 0.879 | 0.002 |
| 1<br>2 | X71-X83 | Intentional self-harm | | 30<br>29 | 0.2%<br>0.2% | 0.896 | 0.002 |
| 1<br>2 | W54.0 | Bitten by dog | | 45<br>59 | 0.3%<br>0.5% | 0.169 | 0.017 |
| 1<br>2 | L60.0 | Ingrowing nail | | 148<br>193 | 1.1%<br>1.5% | 0.014 | 0.031 |
| 1<br>2 | B07 | Viral warts | | 253<br>286 | 2.0%<br>2.2% | 0.151 | 0.018 |
| 1<br>2 | I00-I99 | Diseases of the circulatory system | | 5,298<br>5,284 | 41.0%<br>40.9% | 0.859 | 0.002 |
| 1<br>2 | F41 | Other anxiety disorders | | 4,048<br>4,132 | 31.3%<br>32.0% | 0.261 | 0.014 |
| 1<br>2 | F41.1 | Generalized anxiety disorder | | 1,304<br>1,371 | 10.1%<br>10.6% | 0.171 | 0.017 |
| 1<br>2 | F40 | Phobic anxiety disorders | | 171<br>193 | 1.3%<br>1.5% | 0.246 | 0.014 |
| 1<br>2 | F41.8 | Other specified anxiety disorders | | 671<br>700 | 5.2%<br>5.4% | 0.421 | 0.010 |
| 1<br>2 | F41.0 | Panic disorder [episodic paroxysmal anxiety] | | 490<br>513 | 3.8%<br>4.0% | 0.459 | 0.009 |
| 1<br>2 | F41.3 | Other mixed anxiety disorders | | 12<br>16 | 0.1%<br>0.1% | 0.449 | 0.009 |
| 1<br>2 | F41.9 | Anxiety disorder, unspecified | | 3,322<br>3,385 | 25.7%<br>26.2% | 0.371 | 0.011 |
| 1<br>2 | F42 | Obsessive-compulsive disorder | | 135<br>149 | 1.0%<br>1.2% | 0.404 | 0.010 |
| 1<br>2 | F30.2 | Manic episode, severe with psychotic symptoms | | 0<br>0 | 0%<br>0% | -- | -- |
| 1<br>2 | F31.2 | Bipolar disorder, current episode manic severe with psychotic features | | 10<br>10 | 0.1%<br>0.1% | 1 | <0.001 |
| 1<br>2 | F31.5 | Bipolar disorder, current episode depressed, severe, with psychotic features | | 10<br>10 | 0.1%<br>0.1% | 1 | <0.001 |
| 1<br>2 | F32.3 | Major depressive disorder, single episode, severe with psychotic features | | 13<br>15 | 0.1%<br>0.1% | 0.705 | 0.005 |
| 1<br>2 | F33.3 | Major depressive disorder, recurrent, severe with psychotic symptoms | | 21<br>21 | 0.2%<br>0.2% | 1 | <0.001 |
| **Medication** | | | | | | | |
| Cohort | | | Mean ± SD | Patients | % of Cohort | P-Value | Std diff. |
| 1<br>2 | R01AD | Corticosteroids | | 6,543<br>6,403 | 50.7%<br>49.6% | 0.081 | 0.022 |

## Supplementary Table S7. Baseline characteristics after propensity score matching low-dose methotrexate *vs* Celecoxib

| Cohort 1. Low-dose methotrexate (N = 8,647) and cohort 2. Celecoxib (N = 8,647) | | | | | | | |
|---|---|---|---|---|---|---|---|
| **Demographics** | | | | | | | |
| Cohort | | | Mean ± SD | Patients | % of Cohort | P-Value | Std diff. |
| 1<br>2 | AI | Age at Index | 36.3 +/- 7.4<br>36.1 +/- 7.4 | 8,647<br>8,647 | 100%<br>100% | 0.204 | 0.019 |
| 1<br>2 | F | Female | | 7,021<br>7,024 | 81.2%<br>81.2% | 0.953 | 0.001 |
| 1<br>2 | 2054-5 | Black or African American | | 1,073<br>1,082 | 12.4%<br>12.5% | 0.836 | 0.003 |
| 1<br>2 | M | Male | | 1,625<br>1,621 | 18.8%<br>18.7% | 0.938 | 0.001 |
| 1<br>2 | 2106-3 | White | | 6,304<br>6,179 | 72.9%<br>71.5% | 0.034 | 0.032 |
| 1<br>2 | 1002-5 | American Indian or Alaska Native | | 74<br>92 | 0.9%<br>1.1% | 0.160 | 0.021 |
| 1<br>2 | UNK | Unknown Race | | 501<br>536 | 5.8%<br>6.2% | 0.262 | 0.017 |
| 1<br>2 | 2076-8 | Native Hawaiian or Other Pacific Islander | | 42<br>48 | 0.5%<br>0.6% | 0.526 | 0.010 |
| 1<br>2 | UN | Unknown Ethnicity | | 1,327<br>1,334 | 15.3%<br>15.4% | 0.883 | 0.002 |
| 1<br>2 | 2186-5 | Not Hispanic or Latino | | 6,332<br>6,260 | 73.2%<br>72.4% | 0.218 | 0.019 |
| 1<br>2 | 2135-2 | Hispanic or Latino | | 988<br>1,053 | 11.4%<br>12.2% | 0.126 | 0.023 |
| 1<br>2 | 2131-1 | Other Race | | 391<br>442 | 4.5%<br>5.1% | 0.070 | 0.028 |
| 1<br>2 | 2028-9 | Asian | | 262<br>268 | 3.0%<br>3.1% | 0.791 | 0.004 |
| **Diagnosis** | | | | | | | |
| Cohort | | | Mean ± SD | Patients | % of Cohort | P-Value | Std diff. |
| 1<br>2 | Z55 | Problems related to education and literacy | | 18<br>22 | 0.2%<br>0.3% | 0.527 | 0.010 |
| 1<br>2 | Z56 | Problems related to employment and unemployment | | 44<br>56 | 0.5%<br>0.6% | 0.229 | 0.018 |
| 1<br>2 | Z81 | Family history of mental and behavioral disorders | | 85<br>99 | 1.0%<br>1.1% | 0.299 | 0.016 |
| 1<br>2 | E70-E88 | Metabolic disorders | | 2,569<br>2,612 | 29.7%<br>30.2% | 0.475 | 0.011 |
| 1<br>2 | F31 | Bipolar disorder | | 433<br>459 | 5.0%<br>5.3% | 0.371 | 0.014 |
| 1<br>2 | F32 | Depressive episode | | 2,479<br>2,519 | 28.7%<br>29.1% | 0.502 | 0.010 |
| 1<br>2 | F33 | Major depressive disorder, recurrent | | 735<br>783 | 8.5%<br>9.1% | 0.197 | 0.020 |
| 1<br>2 | X71 | Intentional self-harm by drowning and submersion | | 0<br>10 | 0%<br>0.1% | 0.002 | 0.048 |

| | | | | | | | |
|---|---|---|---|---|---|---|---|
| 1<br>2 | R45.851 | Suicidal ideations | | 180<br>196 | 2.1%<br>2.3% | 0.404 | 0.013 |
| 1<br>2 | T14.91 | Suicide attempt | | 14<br>13 | 0.2%<br>0.2% | 0.847 | 0.003 |
| 1<br>2 | X71-X83 | Intentional self-harm | | 20<br>23 | 0.2%<br>0.3% | 0.647 | 0.007 |
| 1<br>2 | W54.0 | Bitten by dog | | 34<br>36 | 0.4%<br>0.4% | 0.811 | 0.004 |
| 1<br>2 | L60.0 | Ingrowing nail | | 120<br>129 | 1.4%<br>1.5% | 0.566 | 0.009 |
| 1<br>2 | B07 | Viral warts | | 201<br>182 | 2.3%<br>2.1% | 0.326 | 0.015 |
| 1<br>2 | I00-I99 | Diseases of the circulatory system | | 3,678<br>3,613 | 42.5%<br>41.8% | 0.317 | 0.015 |
| 1<br>2 | F41 | Other anxiety disorders | | 3,093<br>3,247 | 35.8%<br>37.6% | 0.015 | 0.037 |
| 1<br>2 | F41.1 | Generalized anxiety disorder | | 1,020<br>1,099 | 11.8%<br>12.7% | 0.067 | 0.028 |
| 1<br>2 | F40 | Phobic anxiety disorders | | 124<br>136 | 1.4%<br>1.6% | 0.453 | 0.011 |
| 1<br>2 | F41.8 | Other specified anxiety disorders | | 547<br>606 | 6.3%<br>7.0% | 0.072 | 0.027 |
| 1<br>2 | F41.0 | Panic disorder [episodic paroxysmal anxiety] | | 363<br>420 | 4.2%<br>4.9% | 0.037 | 0.032 |
| 1<br>2 | F41.3 | Other mixed anxiety disorders | | 10<br>11 | 0.1%<br>0.1% | 0.827 | 0.003 |
| 1<br>2 | F41.9 | Anxiety disorder, unspecified | | 2,606<br>2,718 | 30.1%<br>31.4% | 0.065 | 0.028 |
| 1<br>2 | F42 | Obsessive-compulsive disorder | | 92<br>111 | 1.1%<br>1.3% | 0.180 | 0.020 |
| 1<br>2 | F30.2 | Manic episode, severe with psychotic symptoms | | 0<br>10 | 0%<br>0.1% | 0.002 | 0.048 |
| 1<br>2 | F31.2 | Bipolar disorder, current episode manic severe with psychotic features | | 10<br>10 | 0.1%<br>0.1% | 1 | <0.001 |
| 1<br>2 | F31.5 | Bipolar disorder, current episode depressed, severe, with psychotic features | | 10<br>10 | 0.1%<br>0.1% | 1 | <0.001 |
| 1<br>2 | F32.3 | Major depressive disorder, single episode, severe with psychotic features | | 10<br>11 | 0.1%<br>0.1% | 0.827 | 0.003 |
| 1<br>2 | F33.3 | Major depressive disorder, recurrent, severe with psychotic symptoms | | 18<br>18 | 0.2%<br>0.2% | 1 | <0.001 |
| **Medication** | | | | | | | |
| Cohort | | | Mean ± SD | Patients | % of Cohort | P-Value | Std diff. |
| 1<br>2 | R01AD | Corticosteroids | | 5,140<br>5,058 | 59.4%<br>58.5% | 0.205 | 0.019 |

## Supplementary Table S8. Baseline characteristics after propensity score matching low-dose methotrexate *vs* Infliximab

| Cohort 1. Low-dose methotrexate (N = 1,226) and cohort 2. Infliximab (N = 1,226) | | | | | | | |
|---|---|---|---|---|---|---|---|
| **Demographics** | | | | | | | |
| Cohort | | | Mean ± SD | Patients | % of Cohort | P-Value | Std diff. |
| 1<br>2 | AI | Age at Index | 33.7 +/- 8.7<br>33.8 +/- 8.3 | 1,226<br>1,226 | 100%<br>100% | 0.646 | 0.019 |
| 1<br>2 | F | Female | | 901<br>899 | 73.5%<br>73.3% | 0.927 | 0.004 |
| 1<br>2 | 2054-5 | Black or African American | | 170<br>185 | 13.9%<br>15.1% | 0.389 | 0.035 |
| 1<br>2 | M | Male | | 325<br>327 | 26.5%<br>26.7% | 0.927 | 0.004 |
| 1<br>2 | 2106-3 | White | | 880<br>857 | 71.8%<br>69.9% | 0.307 | 0.041 |
| 1<br>2 | 1002-5 | American Indian or Alaska Native | | 10<br>12 | 0.8%<br>1.0% | 0.668 | 0.017 |
| 1<br>2 | UNK | Unknown Race | | 72<br>74 | 5.9%<br>6.0% | 0.864 | 0.007 |
| 1<br>2 | 2076-8 | Native Hawaiian or Other Pacific Islander | | 10<br>10 | 0.8%<br>0.8% | 1 | <0.001 |
| 1<br>2 | UN | Unknown Ethnicity | | 90<br>96 | 7.3%<br>7.8% | 0.647 | 0.018 |
| 1<br>2 | 2186-5 | Not Hispanic or Latino | | 980<br>977 | 79.9%<br>79.7% | 0.880 | 0.006 |
| 1<br>2 | 2135-2 | Hispanic or Latino | | 156<br>153 | 12.7%<br>12.5% | 0.855 | 0.007 |
| 1<br>2 | 2131-1 | Other Race | | 66<br>68 | 5.4%<br>5.5% | 0.859 | 0.007 |
| 1<br>2 | 2028-9 | Asian | | 23<br>24 | 1.9%<br>2.0% | 0.883 | 0.006 |
| **Diagnosis** | | | | | | | |
| Cohort | | | Mean ± SD | Patients | % of Cohort | P-Value | Std diff. |
| 1<br>2 | Z55 | Problems related to education and literacy | | 10<br>10 | 0.8%<br>0.8% | 1 | <0.001 |
| 1<br>2 | Z56 | Problems related to employment and unemployment | | 10<br>10 | 0.8%<br>0.8% | 1 | <0.001 |
| 1<br>2 | Z81 | Family history of mental and behavioral disorders | | 16<br>15 | 1.3%<br>1.2% | 0.857 | 0.007 |
| 1<br>2 | E70-E88 | Metabolic disorders | | 324<br>336 | 26.4%<br>27.4% | 0.585 | 0.022 |
| 1<br>2 | F31 | Bipolar disorder | | 30<br>39 | 2.4%<br>3.2% | 0.272 | 0.044 |
| 1<br>2 | F32 | Depressive episode | | 283<br>305 | 23.1%<br>24.9% | 0.298 | 0.042 |
| 1<br>2 | F33 | Major depressive disorder, recurrent | | 50<br>74 | 4.1%<br>6.0% | 0.027 | 0.089 |
| 1<br>2 | X71 | Intentional self-harm by drowning and submersion | | 0<br>0 | 0%<br>0% | -- | -- |

| | | | | | | | |
|---|---|---|---|---|---|---|---|
| 1<br>2 | R45.851 | Suicidal ideations | | 18<br>27 | 1.5%<br>2.2% | 0.176 | 0.055 |
| 1<br>2 | T14.91 | Suicide attempt | | 0<br>0 | 0%<br>0% | -- | -- |
| 1<br>2 | X71-X83 | Intentional self-harm | | 10<br>10 | 0.8%<br>0.8% | 1 | <0.001 |
| 1<br>2 | W54.0 | Bitten by dog | | 10<br>10 | 0.8%<br>0.8% | 1 | <0.001 |
| 1<br>2 | L60.0 | Ingrowing nail | | 12<br>12 | 1.0%<br>1.0% | 1 | <0.001 |
| 1<br>2 | B07 | Viral warts | | 27<br>25 | 2.2%<br>2.0% | 0.779 | 0.011 |
| 1<br>2 | I00-I99 | Diseases of the circulatory system | | 415<br>414 | 33.8%<br>33.8% | 0.966 | 0.002 |
| 1<br>2 | F41 | Other anxiety disorders | | 324<br>334 | 26.4%<br>27.2% | 0.649 | 0.018 |
| 1<br>2 | F41.1 | Generalized anxiety disorder | | 91<br>110 | 7.4%<br>9.0% | 0.162 | 0.057 |
| 1<br>2 | F40 | Phobic anxiety disorders | | 10<br>12 | 0.8%<br>1.0% | 0.668 | 0.017 |
| 1<br>2 | F41.8 | Other specified anxiety disorders | | 49<br>56 | 4.0%<br>4.6% | 0.485 | 0.028 |
| 1<br>2 | F41.0 | Panic disorder [episodic paroxysmal anxiety] | | 34<br>39 | 2.8%<br>3.2% | 0.552 | 0.024 |
| 1<br>2 | F41.3 | Other mixed anxiety disorders | | 0<br>0 | 0%<br>0% | -- | -- |
| 1<br>2 | F41.9 | Anxiety disorder, unspecified | | 284<br>288 | 23.2%<br>23.5% | 0.849 | 0.008 |
| 1<br>2 | F42 | Obsessive-compulsive disorder | | 11<br>15 | 0.9%<br>1.2% | 0.430 | 0.032 |
| 1<br>2 | F30.2 | Manic episode, severe with psychotic symptoms | | 0<br>0 | 0%<br>0% | -- | -- |
| 1<br>2 | F31.2 | Bipolar disorder, current episode manic severe with psychotic features | | 10<br>10 | 0.8%<br>0.8% | 1 | <0.001 |
| 1<br>2 | F31.5 | Bipolar disorder, current episode depressed, severe, with psychotic features | | 0<br>0 | 0%<br>0% | -- | -- |
| 1<br>2 | F32.3 | Major depressive disorder, single episode, severe with psychotic features | | 0<br>0 | 0%<br>0% | -- | -- |
| 1<br>2 | F33.3 | Major depressive disorder, recurrent, severe with psychotic symptoms | | 0<br>10 | 0%<br>0.8% | 0.002 | 0.128 |
| **Medication** | | | | | | | |
| Cohort | | | Mean ± SD | Patients | % of Cohort | P-Value | Std diff. |
| 1<br>2 | R01AD | Corticosteroids | | 552<br>548 | 45.0%<br>44.7% | 0.871 | 0.007 |

## Supplementary Table S9. Baseline characteristics after propensity score matching low-dose methotrexate *vs* Adalimumab

**Cohort 1. Low-dose methotrexate (N = 8,915) and cohort 2. Adalimumab (N = 8,915)**

**Demographics**

| Cohort | | | Mean ± SD | Patients | % of Cohort | P-Value | Std diff. |
|---|---|---|---|---|---|---|---|
| 1<br>2 | AI | Age at Index | 34.0 +/- 8.5<br>34.1 +/- 8.3 | 8,915<br>8,915 | 100%<br>100% | 0.452 | 0.011 |
| 1<br>2 | F | Female | | 6,811<br>6,800 | 76.4%<br>76.3% | 0.846 | 0.003 |
| 1<br>2 | 2054-5 | Black or African American | | 999<br>994 | 11.2%<br>11.1% | 0.905 | 0.002 |
| 1<br>2 | M | Male | | 2,098<br>2,110 | 23.5%<br>23.7% | 0.832 | 0.003 |
| 1<br>2 | 2106-3 | White | | 6,404<br>6,358 | 71.8%<br>71.3% | 0.445 | 0.011 |
| 1<br>2 | 1002-5 | American Indian or Alaska Native | | 122<br>121 | 1.4%<br>1.4% | 0.948 | 0.001 |
| 1<br>2 | UNK | Unknown Race | | 673<br>719 | 7.5%<br>8.1% | 0.199 | 0.019 |
| 1<br>2 | 2076-8 | Native Hawaiian or Other Pacific Islander | | 22<br>24 | 0.2%<br>0.3% | 0.768 | 0.004 |
| 1<br>2 | UN | Unknown Ethnicity | | 1,508<br>1,568 | 16.9%<br>17.6% | 0.234 | 0.018 |
| 1<br>2 | 2186-5 | Not Hispanic or Latino | | 6,389<br>6,294 | 71.7%<br>70.6% | 0.116 | 0.024 |
| 1<br>2 | 2135-2 | Hispanic or Latino | | 1,018<br>1,053 | 11.4%<br>11.8% | 0.413 | 0.012 |
| 1<br>2 | 2131-1 | Other Race | | 474<br>470 | 5.3%<br>5.3% | 0.894 | 0.002 |
| 1<br>2 | 2028-9 | Asian | | 221<br>229 | 2.5%<br>2.6% | 0.702 | 0.006 |

**Diagnosis**

| Cohort | | | Mean ± SD | Patients | % of Cohort | P-Value | Std diff. |
|---|---|---|---|---|---|---|---|
| 1<br>2 | Z55 | Problems related to education and literacy | | 10<br>16 | 0.1%<br>0.2% | 0.239 | 0.018 |
| 1<br>2 | Z56 | Problems related to employment and unemployment | | 45<br>41 | 0.5%<br>0.5% | 0.665 | 0.006 |
| 1<br>2 | Z81 | Family history of mental and behavioral disorders | | 30<br>41 | 0.3%<br>0.5% | 0.191 | 0.020 |
| 1<br>2 | E70-E88 | Metabolic disorders | | 1,651<br>1,755 | 18.5%<br>19.7% | 0.048 | 0.030 |
| 1<br>2 | F31 | Bipolar disorder | | 194<br>232 | 2.2%<br>2.6% | 0.062 | 0.028 |
| 1<br>2 | F32 | Depressive episode | | 1,396<br>1,496 | 15.7%<br>16.8% | 0.042 | 0.030 |
| 1<br>2 | F33 | Major depressive disorder, recurrent | | 375<br>431 | 4.2%<br>4.8% | 0.044 | 0.030 |
| 1<br>2 | X71 | Intentional self-harm by drowning and submersion | | 0<br>0 | 0%<br>0% | -- | -- |

| | | | | | | | |
|---|---|---|---|---|---|---|---|
| 1<br>2 | R45.851 | Suicidal ideations | | 112<br>123 | 1.3%<br>1.4% | 0.470 | 0.011 |
| 1<br>2 | T14.91 | Suicide attempt | | 10<br>12 | 0.1%<br>0.1% | 0.670 | 0.006 |
| 1<br>2 | X71-X83 | Intentional self-harm | | 12<br>14 | 0.1%<br>0.2% | 0.695 | 0.006 |
| 1<br>2 | W54.0 | Bitten by dog | | 24<br>21 | 0.3%<br>0.2% | 0.654 | 0.007 |
| 1<br>2 | L60.0 | Ingrowing nail | | 71<br>91 | 0.8%<br>1.0% | 0.114 | 0.024 |
| 1<br>2 | B07 | Viral warts | | 148<br>162 | 1.7%<br>1.8% | 0.422 | 0.012 |
| 1<br>2 | I00-I99 | Diseases of the circulatory system | | 2,411<br>2,445 | 27.0%<br>27.4% | 0.567 | 0.009 |
| 1<br>2 | F41 | Other anxiety disorders | | 1,724<br>1,954 | 19.3%<br>21.9% | <0.001 | 0.064 |
| 1<br>2 | F41.1 | Generalized anxiety disorder | | 526<br>623 | 5.9%<br>7.0% | 0.003 | 0.044 |
| 1<br>2 | F40 | Phobic anxiety disorders | | 81<br>86 | 0.9%<br>1.0% | 0.697 | 0.006 |
| 1<br>2 | F41.8 | Other specified anxiety disorders | | 248<br>312 | 2.8%<br>3.5% | 0.006 | 0.041 |
| 1<br>2 | F41.0 | Panic disorder [episodic paroxysmal anxiety] | | 199<br>224 | 2.2%<br>2.5% | 0.219 | 0.018 |
| 1<br>2 | F41.3 | Other mixed anxiety disorders | | 10<br>10 | 0.1%<br>0.1% | 1 | <0.001 |
| 1<br>2 | F41.9 | Anxiety disorder, unspecified | | 1,417<br>1,586 | 15.9%<br>17.8% | 0.001 | 0.051 |
| 1<br>2 | F42 | Obsessive-compulsive disorder | | 75<br>82 | 0.8%<br>0.9% | 0.575 | 0.008 |
| 1<br>2 | F30.2 | Manic episode, severe with psychotic symptoms | | 0<br>0 | 0%<br>0% | -- | -- |
| 1<br>2 | F31.2 | Bipolar disorder, current episode manic severe with psychotic features | | 10<br>10 | 0.1%<br>0.1% | 1 | <0.001 |
| 1<br>2 | F31.5 | Bipolar disorder, current episode depressed, severe, with psychotic features | | 10<br>10 | 0.1%<br>0.1% | 1 | <0.001 |
| 1<br>2 | F32.3 | Major depressive disorder, single episode, severe with psychotic features | | 10<br>10 | 0.1%<br>0.1% | 1 | <0.001 |
| 1<br>2 | F33.3 | Major depressive disorder, recurrent, severe with psychotic symptoms | | 10<br>10 | 0.1%<br>0.1% | 1 | <0.001 |
| **Medication** | | | | | | | |
| Cohort | | | Mean ± SD | Patients | % of Cohort | P-Value | Std diff. |
| 1<br>2 | R01AD | Corticosteroids | | 3,343<br>3,401 | 37.5%<br>38.1% | 0.370 | 0.013 |

## Supplementary Table S10. Baseline characteristics after propensity score matching low-dose methotrexate *vs* Etanercept

| Cohort 1. Low-dose methotrexate (N = 6,240) and cohort 2. Etanercept (N = 6,240) | | | | | | | |
|---|---|---|---|---|---|---|---|
| **Demographics** | | | | | | | |
| Cohort | | | Mean ± SD | Patients | % of Cohort | P-Value | Std diff. |
| 1<br>2 | AI | Age at Index | 34.7 +/- 8.1<br>34.7 +/- 8.1 | 6,240<br>6,240 | 100%<br>100% | 0.850 | 0.003 |
| 1<br>2 | F | Female | | 4,918<br>4,939 | 78.8%<br>79.2% | 0.645 | 0.008 |
| 1<br>2 | 2054-5 | Black or African American | | 548<br>535 | 8.8%<br>8.6% | 0.679 | 0.007 |
| 1<br>2 | M | Male | | 1,319<br>1,298 | 21.1%<br>20.8% | 0.644 | 0.008 |
| 1<br>2 | 2106-3 | White | | 4,538<br>4,515 | 72.7%<br>72.4% | 0.645 | 0.008 |
| 1<br>2 | 1002-5 | American Indian or Alaska Native | | 89<br>99 | 1.4%<br>1.6% | 0.462 | 0.013 |
| 1<br>2 | UNK | Unknown Race | | 495<br>507 | 7.9%<br>8.1% | 0.693 | 0.007 |
| 1<br>2 | 2076-8 | Native Hawaiian or Other Pacific Islander | | 24<br>28 | 0.4%<br>0.4% | 0.578 | 0.010 |
| 1<br>2 | UN | Unknown Ethnicity | | 1,154<br>1,189 | 18.5%<br>19.1% | 0.422 | 0.014 |
| 1<br>2 | 2186-5 | Not Hispanic or Latino | | 4,326<br>4,264 | 69.3%<br>68.3% | 0.231 | 0.021 |
| 1<br>2 | 2135-2 | Hispanic or Latino | | 760<br>787 | 12.2%<br>12.6% | 0.463 | 0.013 |
| 1<br>2 | 2131-1 | Other Race | | 331<br>356 | 5.3%<br>5.7% | 0.326 | 0.018 |
| 1<br>2 | 2028-9 | Asian | | 215<br>200 | 3.4%<br>3.2% | 0.454 | 0.013 |
| **Diagnosis** | | | | | | | |
| Cohort | | | Mean ± SD | Patients | % of Cohort | P-Value | Std diff. |
| 1<br>2 | Z55 | Problems related to education and literacy | | 10<br>10 | 0.2%<br>0.2% | 1 | <0.001 |
| 1<br>2 | Z56 | Problems related to employment and unemployment | | 13<br>22 | 0.2%<br>0.4% | 0.128 | 0.027 |
| 1<br>2 | Z81 | Family history of mental and behavioral disorders | | 15<br>23 | 0.2%<br>0.4% | 0.194 | 0.023 |
| 1<br>2 | E70-E88 | Metabolic disorders | | 937<br>974 | 15.0%<br>15.6% | 0.358 | 0.016 |
| 1<br>2 | F31 | Bipolar disorder | | 103<br>151 | 1.7%<br>2.4% | 0.002 | 0.054 |
| 1<br>2 | F32 | Depressive episode | | 823<br>908 | 13.2%<br>14.6% | 0.028 | 0.039 |
| 1<br>2 | F33 | Major depressive disorder, recurrent | | 233<br>296 | 3.7%<br>4.7% | 0.005 | 0.050 |
| 1<br>2 | X71 | Intentional self-harm by drowning and submersion | | 0<br>0 | 0%<br>0% | -- | -- |

| | | | | | | | |
|---|---|---|---|---|---|---|---|
| 1<br>2 | R45.851 | Suicidal ideations | | 66<br>79 | 1.1%<br>1.3% | 0.278 | 0.019 |
| 1<br>2 | T14.91 | Suicide attempt | | 10<br>10 | 0.2%<br>0.2% | 1 | <0.001 |
| 1<br>2 | X71-X83 | Intentional self-harm | | 10<br>10 | 0.2%<br>0.2% | 1 | <0.001 |
| 1<br>2 | W54.0 | Bitten by dog | | 18<br>11 | 0.3%<br>0.2% | 0.193 | 0.023 |
| 1<br>2 | L60.0 | Ingrowing nail | | 44<br>52 | 0.7%<br>0.8% | 0.412 | 0.015 |
| 1<br>2 | B07 | Viral warts | | 84<br>83 | 1.3%<br>1.3% | 0.938 | 0.001 |
| 1<br>2 | I00-I99 | Diseases of the circulatory system | | 1,516<br>1,545 | 24.3%<br>24.8% | 0.546 | 0.011 |
| 1<br>2 | F41 | Other anxiety disorders | | 1,057<br>1,215 | 16.9%<br>19.5% | <0.001 | 0.066 |
| 1<br>2 | F41.1 | Generalized anxiety disorder | | 290<br>382 | 4.6%<br>6.1% | <0.001 | 0.065 |
| 1<br>2 | F40 | Phobic anxiety disorders | | 45<br>54 | 0.7%<br>0.9% | 0.364 | 0.016 |
| 1<br>2 | F41.8 | Other specified anxiety disorders | | 142<br>192 | 2.3%<br>3.1% | 0.006 | 0.050 |
| 1<br>2 | F41.0 | Panic disorder [episodic paroxysmal anxiety] | | 90<br>132 | 1.4%<br>2.1% | 0.004 | 0.051 |
| 1<br>2 | F41.3 | Other mixed anxiety disorders | | 0<br>10 | 0%<br>0.2% | 0.002 | 0.057 |
| 1<br>2 | F41.9 | Anxiety disorder, unspecified | | 871<br>977 | 14.0%<br>15.7% | 0.008 | 0.048 |
| 1<br>2 | F42 | Obsessive-compulsive disorder | | 38<br>47 | 0.6%<br>0.8% | 0.327 | 0.018 |
| 1<br>2 | F30.2 | Manic episode, severe with psychotic symptoms | | 0<br>0 | 0%<br>0% | -- | -- |
| 1<br>2 | F31.2 | Bipolar disorder, current episode manic severe with psychotic features | | 10<br>10 | 0.2%<br>0.2% | 1 | <0.001 |
| 1<br>2 | F31.5 | Bipolar disorder, current episode depressed, severe, with psychotic features | | 0<br>10 | 0%<br>0.2% | 0.002 | 0.057 |
| 1<br>2 | F32.3 | Major depressive disorder, single episode, severe with psychotic features | | 10<br>10 | 0.2%<br>0.2% | 1 | <0.001 |
| 1<br>2 | F33.3 | Major depressive disorder, recurrent, severe with psychotic symptoms | | 11<br>10 | 0.2%<br>0.2% | 0.827 | 0.004 |
| **Medication** | | | | | | | |
| Cohort | | | Mean ± SD | Patients | % of Cohort | P-Value | Std diff. |
| 1<br>2 | R01AD | Corticosteroids | | 1,949<br>1,986 | 31.2%<br>31.8% | 0.476 | 0.013 |

## Supplementary Table S11. Baseline characteristics after propensity score matching low-dose methotrexate *vs* Tocilizumab

| Cohort 1. Low-dose methotrexate (N = 1,617) and cohort 2. Tocilizumab (N = 1,617) | | | | | | | |
|---|---|---|---|---|---|---|---|
| **Demographics** | | | | | | | |
| Cohort | | | Mean ± SD | Patients | % of Cohort | P-Value | Std diff. |
| 1<br>2 | AI | Age at Index | 34.6 +/- 8.5<br>34.3 +/- 8.9 | 1,617<br>1,617 | 100%<br>100% | 0.445 | 0.027 |
| 1<br>2 | F | Female | | 1,395<br>1,379 | 86.3%<br>85.3% | 0.421 | 0.028 |
| 1<br>2 | 2054-5 | Black or African American | | 202<br>205 | 12.5%<br>12.7% | 0.874 | 0.006 |
| 1<br>2 | M | Male | | 222<br>238 | 13.7%<br>14.7% | 0.421 | 0.028 |
| 1<br>2 | 2106-3 | White | | 1,103<br>1,087 | 68.2%<br>67.2% | 0.547 | 0.021 |
| 1<br>2 | 1002-5 | American Indian or Alaska Native | | 21<br>23 | 1.3%<br>1.4% | 0.761 | 0.011 |
| 1<br>2 | UNK | Unknown Race | | 143<br>137 | 8.8%<br>8.5% | 0.708 | 0.013 |
| 1<br>2 | 2076-8 | Native Hawaiian or Other Pacific Islander | | 10<br>10 | 0.6%<br>0.6% | 1 | <0.001 |
| 1<br>2 | UN | Unknown Ethnicity | | 285<br>284 | 17.6%<br>17.6% | 0.963 | 0.002 |
| 1<br>2 | 2186-5 | Not Hispanic or Latino | | 1,155<br>1,133 | 71.4%<br>70.1% | 0.395 | 0.030 |
| 1<br>2 | 2135-2 | Hispanic or Latino | | 177<br>200 | 10.9%<br>12.4% | 0.208 | 0.044 |
| 1<br>2 | 2131-1 | Other Race | | 94<br>104 | 5.8%<br>6.4% | 0.463 | 0.026 |
| 1<br>2 | 2028-9 | Asian | | 46<br>54 | 2.8%<br>3.3% | 0.416 | 0.029 |
| **Diagnosis** | | | | | | | |
| Cohort | | | Mean ± SD | Patients | % of Cohort | P-Value | Std diff. |
| 1<br>2 | Z55 | Problems related to education and literacy | | 10<br>10 | 0.6%<br>0.6% | 1 | <0.001 |
| 1<br>2 | Z56 | Problems related to employment and unemployment | | 12<br>10 | 0.7%<br>0.6% | 0.669 | 0.015 |
| 1<br>2 | Z81 | Family history of mental and behavioral disorders | | 10<br>10 | 0.6%<br>0.6% | 1 | <0.001 |
| 1<br>2 | E70-E88 | Metabolic disorders | | 378<br>402 | 23.4%<br>24.9% | 0.324 | 0.035 |
| 1<br>2 | F31 | Bipolar disorder | | 36<br>45 | 2.2%<br>2.8% | 0.311 | 0.036 |
| 1<br>2 | F32 | Depressive episode | | 264<br>285 | 16.3%<br>17.6% | 0.325 | 0.035 |
| 1<br>2 | F33 | Major depressive disorder, recurrent | | 68<br>87 | 4.2%<br>5.4% | 0.118 | 0.055 |
| 1<br>2 | X71 | Intentional self-harm by drowning and submersion | | 0<br>0 | 0%<br>0% | -- | -- |

| | | | | | | | |
|---|---|---|---|---|---|---|---|
| 1<br>2 | R45.851 | Suicidal ideations | | 13<br>18 | 0.8%<br>1.1% | 0.367 | 0.032 |
| 1<br>2 | T14.91 | Suicide attempt | | 0<br>10 | 0%<br>0.6% | 0.002 | 0.112 |
| 1<br>2 | X71-X83 | Intentional self-harm | | 10<br>10 | 0.6%<br>0.6% | 1 | <0.001 |
| 1<br>2 | W54.0 | Bitten by dog | | 10<br>10 | 0.6%<br>0.6% | 1 | <0.001 |
| 1<br>2 | L60.0 | Ingrowing nail | | 19<br>19 | 1.2%<br>1.2% | 1 | <0.001 |
| 1<br>2 | B07 | Viral warts | | 18<br>38 | 1.1%<br>2.4% | 0.007 | 0.095 |
| 1<br>2 | I00-I99 | Diseases of the circulatory system | | 561<br>563 | 34.7%<br>34.8% | 0.941 | 0.003 |
| 1<br>2 | F41 | Other anxiety disorders | | 325<br>362 | 20.1%<br>22.4% | 0.112 | 0.056 |
| 1<br>2 | F41.1 | Generalized anxiety disorder | | 90<br>92 | 5.6%<br>5.7% | 0.879 | 0.005 |
| 1<br>2 | F40 | Phobic anxiety disorders | | 10<br>18 | 0.6%<br>1.1% | 0.129 | 0.053 |
| 1<br>2 | F41.8 | Other specified anxiety disorders | | 35<br>47 | 2.2%<br>2.9% | 0.179 | 0.047 |
| 1<br>2 | F41.0 | Panic disorder [episodic paroxysmal anxiety] | | 28<br>40 | 1.7%<br>2.5% | 0.141 | 0.052 |
| 1<br>2 | F41.3 | Other mixed anxiety disorders | | 0<br>0 | 0%<br>0% | -- | -- |
| 1<br>2 | F41.9 | Anxiety disorder, unspecified | | 286<br>309 | 17.7%<br>19.1% | 0.297 | 0.037 |
| 1<br>2 | F42 | Obsessive-compulsive disorder | | 10<br>10 | 0.6%<br>0.6% | 1 | <0.001 |
| 1<br>2 | F30.2 | Manic episode, severe with psychotic symptoms | | 0<br>0 | 0%<br>0% | -- | -- |
| 1<br>2 | F31.2 | Bipolar disorder, current episode manic severe with psychotic features | | 0<br>0 | 0%<br>0% | -- | -- |
| 1<br>2 | F31.5 | Bipolar disorder, current episode depressed, severe, with psychotic features | | 0<br>0 | 0%<br>0% | -- | -- |
| 1<br>2 | F32.3 | Major depressive disorder, single episode, severe with psychotic features | | 10<br>0 | 0.6%<br>0% | 0.002 | 0.112 |
| 1<br>2 | F33.3 | Major depressive disorder, recurrent, severe with psychotic symptoms | | 10<br>10 | 0.6%<br>0.6% | 1 | <0.001 |
| **Medication** | | | | | | | |
| Cohort | | | Mean ± SD | Patients | % of Cohort | P-Value | Std diff. |
| 1<br>2 | R01AD | Corticosteroids | | 701<br>695 | 43.4%<br>43.0% | 0.831 | 0.007 |

## Supplementary Table S12. Baseline characteristics after propensity score matching low-dose methotrexate *vs* Hydroxychloroquine

**Cohort 1. Low-dose methotrexate (N = 21,445) and cohort 2. Hydroxychloroquine (N = 21,445)**

**Demographics**

| Cohort | | | Mean ± SD | Patients | % of Cohort | P-Value | Std diff. |
|---|---|---|---|---|---|---|---|
| 1<br>2 | AI | Age at Index | 35.1 +/- 7.9<br>35.1 +/- 7.4 | 21,445<br>21,445 | 100%<br>100% | 0.363 | 0.009 |
| 1<br>2 | F | Female | | 19,017<br>18,993 | 88.7%<br>88.6% | 0.715 | 0.004 |
| 1<br>2 | 2054-5 | Black or African American | | 2,891<br>2,920 | 13.5%<br>13.6% | 0.682 | 0.004 |
| 1<br>2 | M | Male | | 2,421<br>2,448 | 11.3%<br>11.4% | 0.681 | 0.004 |
| 1<br>2 | 2106-3 | White | | 14,324<br>14,198 | 66.8%<br>66.2% | 0.197 | 0.012 |
| 1<br>2 | 1002-5 | American Indian or Alaska Native | | 255<br>260 | 1.2%<br>1.2% | 0.825 | 0.002 |
| 1<br>2 | UNK | Unknown Race | | 1,893<br>1,926 | 8.8%<br>9.0% | 0.576 | 0.005 |
| 1<br>2 | 2076-8 | Native Hawaiian or Other Pacific Islander | | 118<br>107 | 0.6%<br>0.5% | 0.462 | 0.007 |
| 1<br>2 | UN | Unknown Ethnicity | | 4,046<br>4,034 | 18.9%<br>18.8% | 0.882 | 0.001 |
| 1<br>2 | 2186-5 | Not Hispanic or Latino | | 14,519<br>14,484 | 67.7%<br>67.5% | 0.718 | 0.003 |
| 1<br>2 | 2135-2 | Hispanic or Latino | | 2,880<br>2,927 | 13.4%<br>13.6% | 0.507 | 0.006 |
| 1<br>2 | 2131-1 | Other Race | | 1,192<br>1,215 | 5.6%<br>5.7% | 0.629 | 0.005 |
| 1<br>2 | 2028-9 | Asian | | 772<br>819 | 3.6%<br>3.8% | 0.230 | 0.012 |

**Diagnosis**

| Cohort | | | Mean ± SD | Patients | % of Cohort | P-Value | Std diff. |
|---|---|---|---|---|---|---|---|
| 1<br>2 | Z55 | Problems related to education and literacy | | 36<br>31 | 0.2%<br>0.1% | 0.541 | 0.006 |
| 1<br>2 | Z56 | Problems related to employment and unemployment | | 63<br>64 | 0.3%<br>0.3% | 0.929 | 0.001 |
| 1<br>2 | Z81 | Family history of mental and behavioral disorders | | 107<br>117 | 0.5%<br>0.5% | 0.503 | 0.006 |
| 1<br>2 | E70-E88 | Metabolic disorders | | 4,236<br>4,265 | 19.8%<br>19.9% | 0.725 | 0.003 |
| 1<br>2 | F31 | Bipolar disorder | | 615<br>659 | 2.9%<br>3.1% | 0.211 | 0.012 |
| 1<br>2 | F32 | Depressive episode | | 3,785<br>3,813 | 17.6%<br>17.8% | 0.723 | 0.003 |
| 1<br>2 | F33 | Major depressive disorder, recurrent | | 994<br>1,031 | 4.6%<br>4.8% | 0.400 | 0.008 |
| 1<br>2 | X71 | Intentional self-harm by drowning and submersion | | 0<br>10 | 0%<br>0.0% | 0.002 | 0.031 |

| | | | | | | | |
|---|---|---|---|---|---|---|---|
| 1<br>2 | R45.851 | Suicidal ideations | | 268<br>282 | 1.2%<br>1.3% | 0.548 | 0.006 |
| 1<br>2 | T14.91 | Suicide attempt | | 20<br>22 | 0.1%<br>0.1% | 0.758 | 0.003 |
| 1<br>2 | X71-X83 | Intentional self-harm | | 30<br>35 | 0.1%<br>0.2% | 0.535 | 0.006 |
| 1<br>2 | W54.0 | Bitten by dog | | 61<br>57 | 0.3%<br>0.3% | 0.712 | 0.004 |
| 1<br>2 | L60.0 | Ingrowing nail | | 179<br>170 | 0.8%<br>0.8% | 0.629 | 0.005 |
| 1<br>2 | B07 | Viral warts | | 336<br>301 | 1.6%<br>1.4% | 0.162 | 0.013 |
| 1<br>2 | I00-I99 | Diseases of the circulatory system | | 6,692<br>6,720 | 31.2%<br>31.3% | 0.771 | 0.003 |
| 1<br>2 | F41 | Other anxiety disorders | | 4,624<br>4,721 | 21.6%<br>22.0% | 0.257 | 0.011 |
| 1<br>2 | F41.1 | Generalized anxiety disorder | | 1,456<br>1,502 | 6.8%<br>7.0% | 0.381 | 0.008 |
| 1<br>2 | F40 | Phobic anxiety disorders | | 198<br>206 | 0.9%<br>1.0% | 0.689 | 0.004 |
| 1<br>2 | F41.8 | Other specified anxiety disorders | | 736<br>749 | 3.4%<br>3.5% | 0.731 | 0.003 |
| 1<br>2 | F41.0 | Panic disorder [episodic paroxysmal anxiety] | | 521<br>533 | 2.4%<br>2.5% | 0.708 | 0.004 |
| 1<br>2 | F41.3 | Other mixed anxiety disorders | | 14<br>15 | 0.1%<br>0.1% | 0.853 | 0.002 |
| 1<br>2 | F41.9 | Anxiety disorder, unspecified | | 3,717<br>3,789 | 17.3%<br>17.7% | 0.360 | 0.009 |
| 1<br>2 | F42 | Obsessive-compulsive disorder | | 152<br>161 | 0.7%<br>0.8% | 0.610 | 0.005 |
| 1<br>2 | F30.2 | Manic episode, severe with psychotic symptoms | | 0<br>10 | 0%<br>0.0% | 0.002 | 0.031 |
| 1<br>2 | F31.2 | Bipolar disorder, current episode manic severe with psychotic features | | 10<br>10 | 0.0%<br>0.0% | 1 | <0.001 |
| 1<br>2 | F31.5 | Bipolar disorder, current episode depressed, severe, with psychotic features | | 10<br>10 | 0.0%<br>0.0% | 1 | <0.001 |
| 1<br>2 | F32.3 | Major depressive disorder, single episode, severe with psychotic features | | 13<br>17 | 0.1%<br>0.1% | 0.465 | 0.007 |
| 1<br>2 | F33.3 | Major depressive disorder, recurrent, severe with psychotic symptoms | | 20<br>19 | 0.1%<br>0.1% | 0.873 | 0.002 |
| **Medication** | | | | | | | |
| Cohort | | | Mean ± SD | Patients | % of Cohort | P-Value | Std diff. |
| 1<br>2 | R01AD | Corticosteroids | | 7,548<br>7,507 | 35.2%<br>35.0% | 0.678 | 0.004 |

# Supplementary Figure 1A

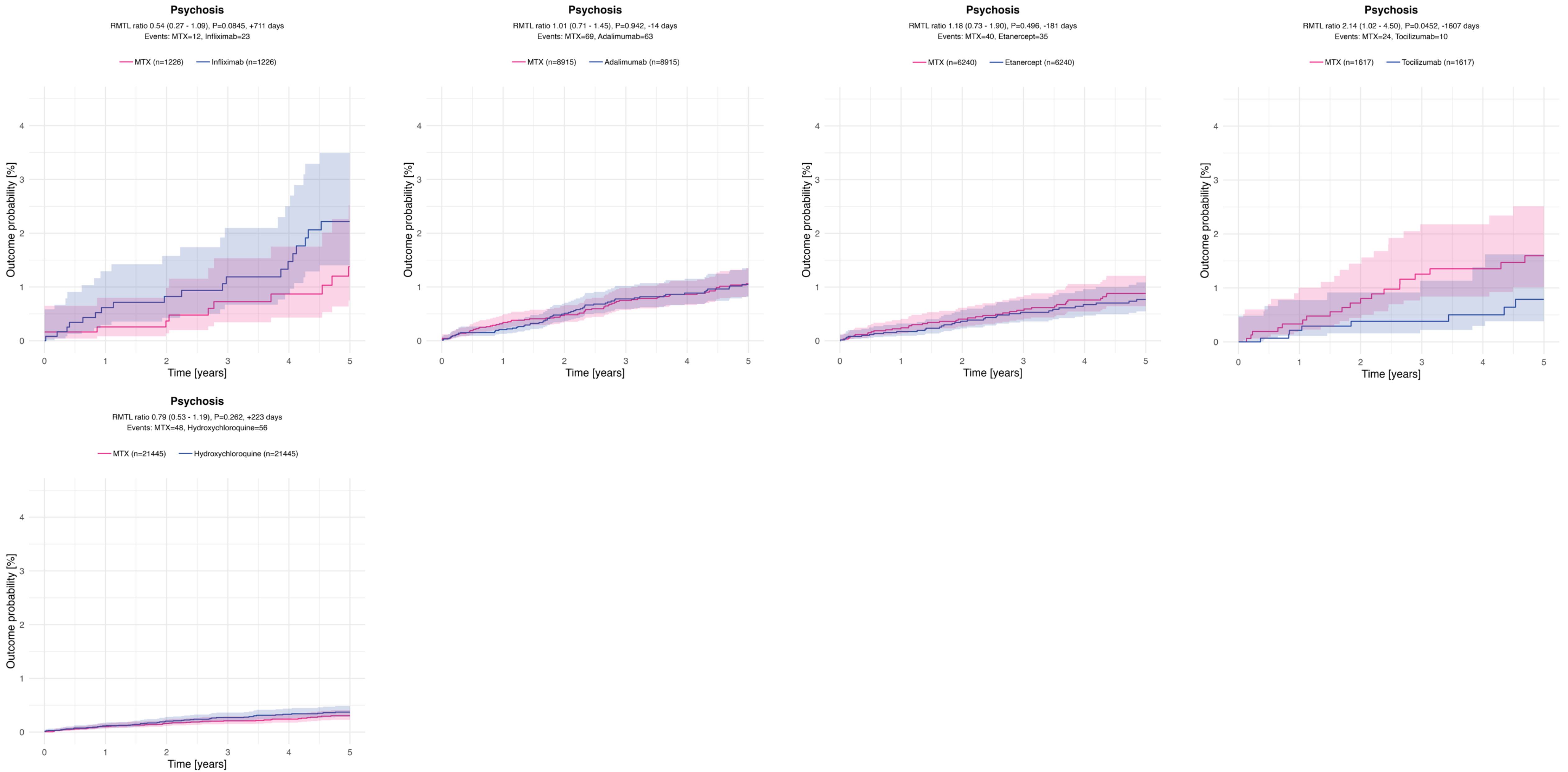


**Supplementary Figure 1A.** Curves representing the Kaplan–Meier estimates of the cumulative incidence of psychosis after initiation of low-dose methotrexate *vs* comparator drugs. Cohort of individuals with rheumatoid arthritis who initiated pharmacological treatment at age ≤45 years. rRMTL <1 indicate that the risk for psychosis is lower after low-dose methotrexate than after the comparator drug, and conversely for rRMTL >1. P value less than Bonferroni corrected critical value for multiple comparison: $\alpha = 0.05/44 = 0.0011$. Cohort size after propensity score matching: Infliximab (N=1,226), Adalimumab (N=8,915), Etanercept (N=6,240), Tocilizumab (N=1,617), Hydroxychloroquine (N=21,445). rRMTL, restricted mean time lost ratio.

## Supplementary Figure 1B

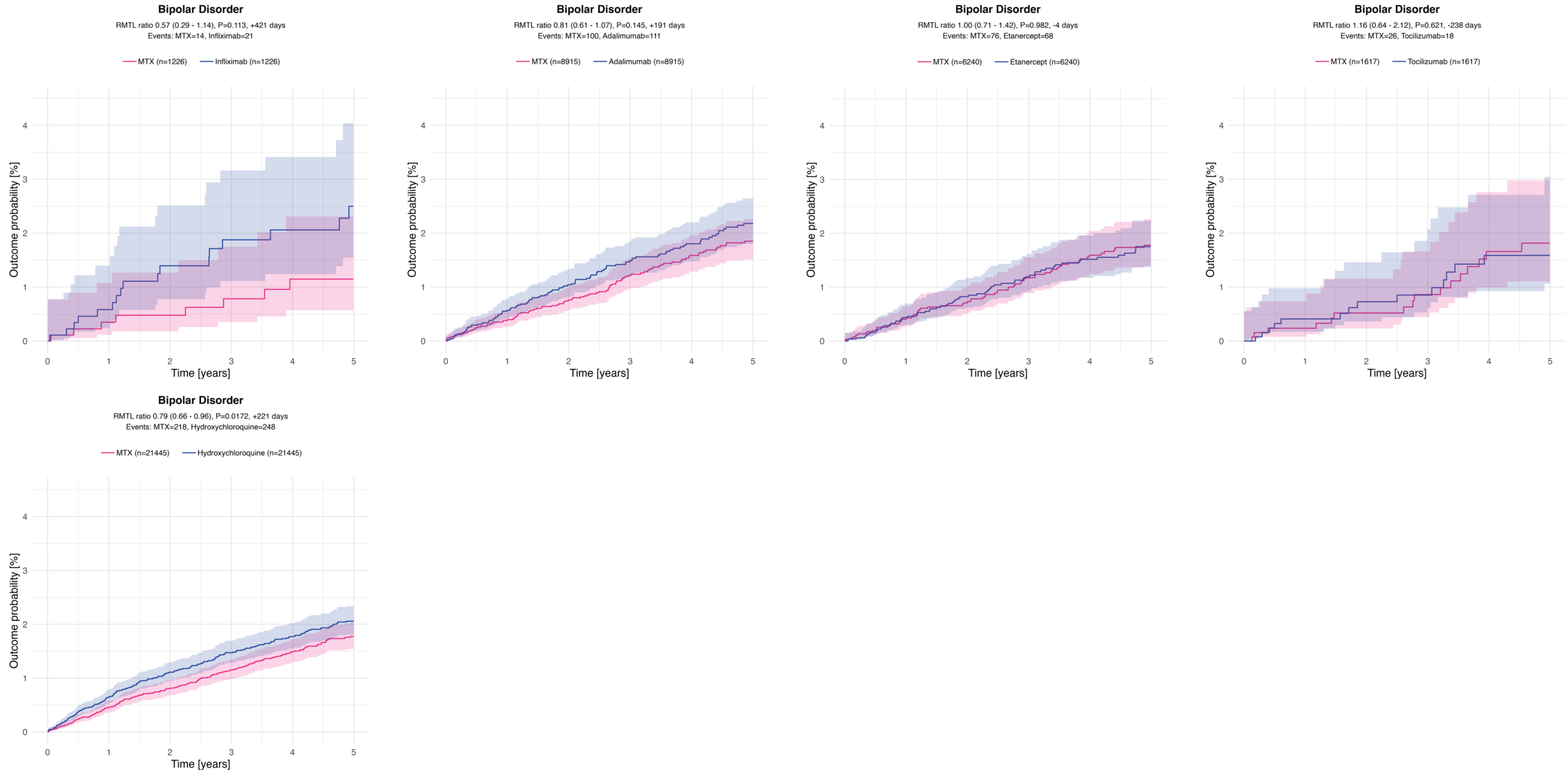


**Supplementary Figure 1B.** Curves representing the Kaplan–Meier estimates of the cumulative incidence of bipolar disorder after initiation of low-dose methotrexate *vs* comparator drugs. Cohort of individuals with rheumatoid arthritis who initiated pharmacological treatment at age ≤45 years. rRMTL <1 indicate that the risk for bipolar disorder is lower after low-dose methotrexate than after the comparator drug, and conversely for rRMTL >1. P value less than Bonferroni corrected critical value for multiple comparison: $\alpha = 0.05/44 = 0.0011$. Cohort size after propensity score matching: Infliximab (N=1,226), Adalimumab (N=8,915), Etanercept (N=6,240), Tocilizumab (N=1,617), Hydroxychloroquine (N=21,445). rRMTL, restricted mean time lost ratio.

## Supplementary Figure 1C

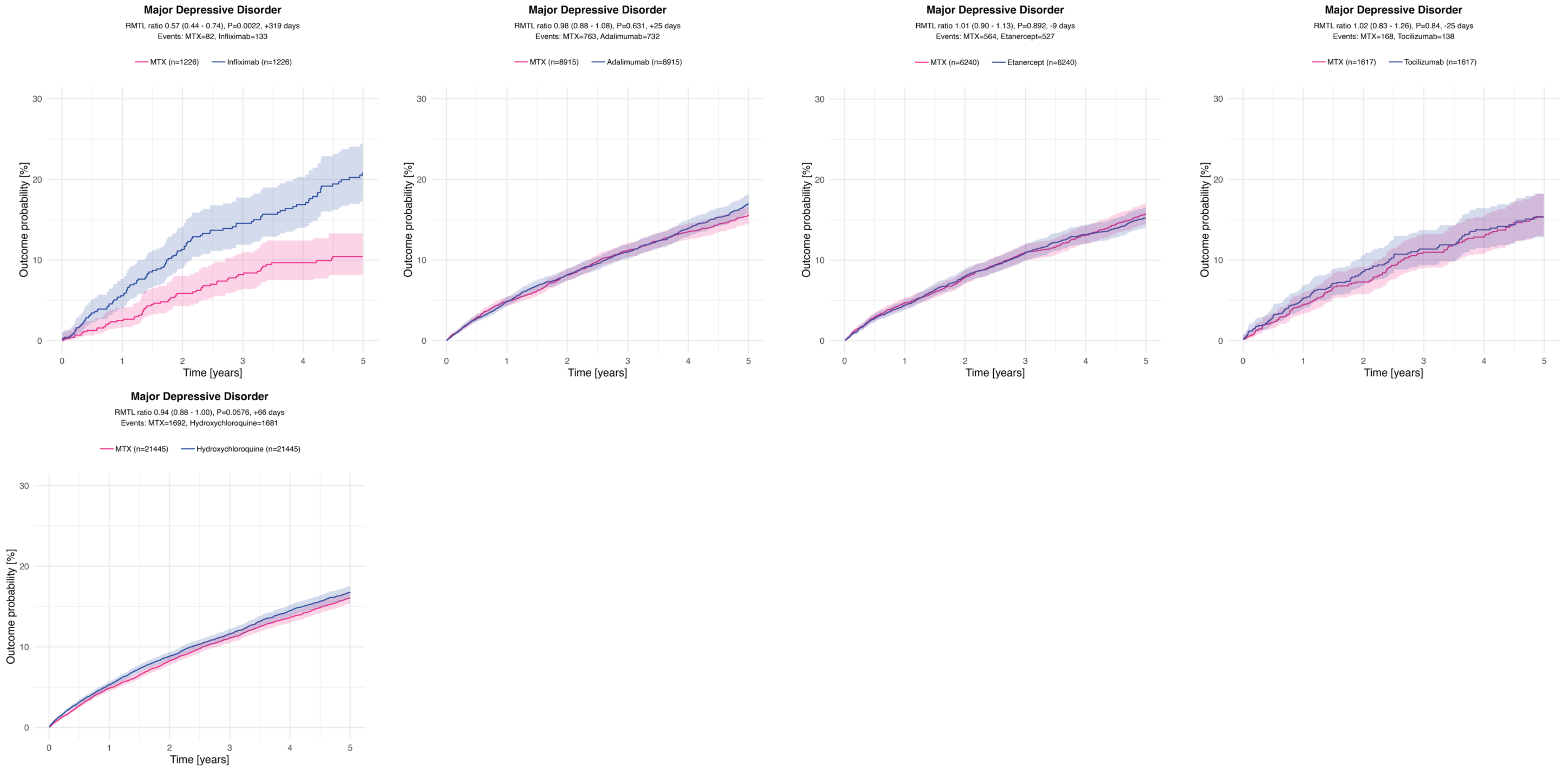


**Supplementary Figure 1C.** Curves representing the Kaplan–Meier estimates of the cumulative incidence of major depressive disorder after initiation of low-dose methotrexate *vs* comparator drugs. Cohort of individuals with rheumatoid arthritis who initiated pharmacological treatment at age ≤45 years. rRMTL <1 indicate that the risk for depression is lower after low-dose methotrexate than after the comparator drug, and conversely for rRMTL >1. P value less than Bonferroni corrected critical value for multiple comparison: $\alpha = 0.05/44 = 0.0011$. Cohort size after propensity score matching: Infliximab (N=1,226), Adalimumab (N=8,915), Etanercept (N=6,240), Tocilizumab (N=1,617), Hydroxychloroquine (N=21,445). rRMTL, restricted mean time lost ratio.

**Supplementary Figure 1D**

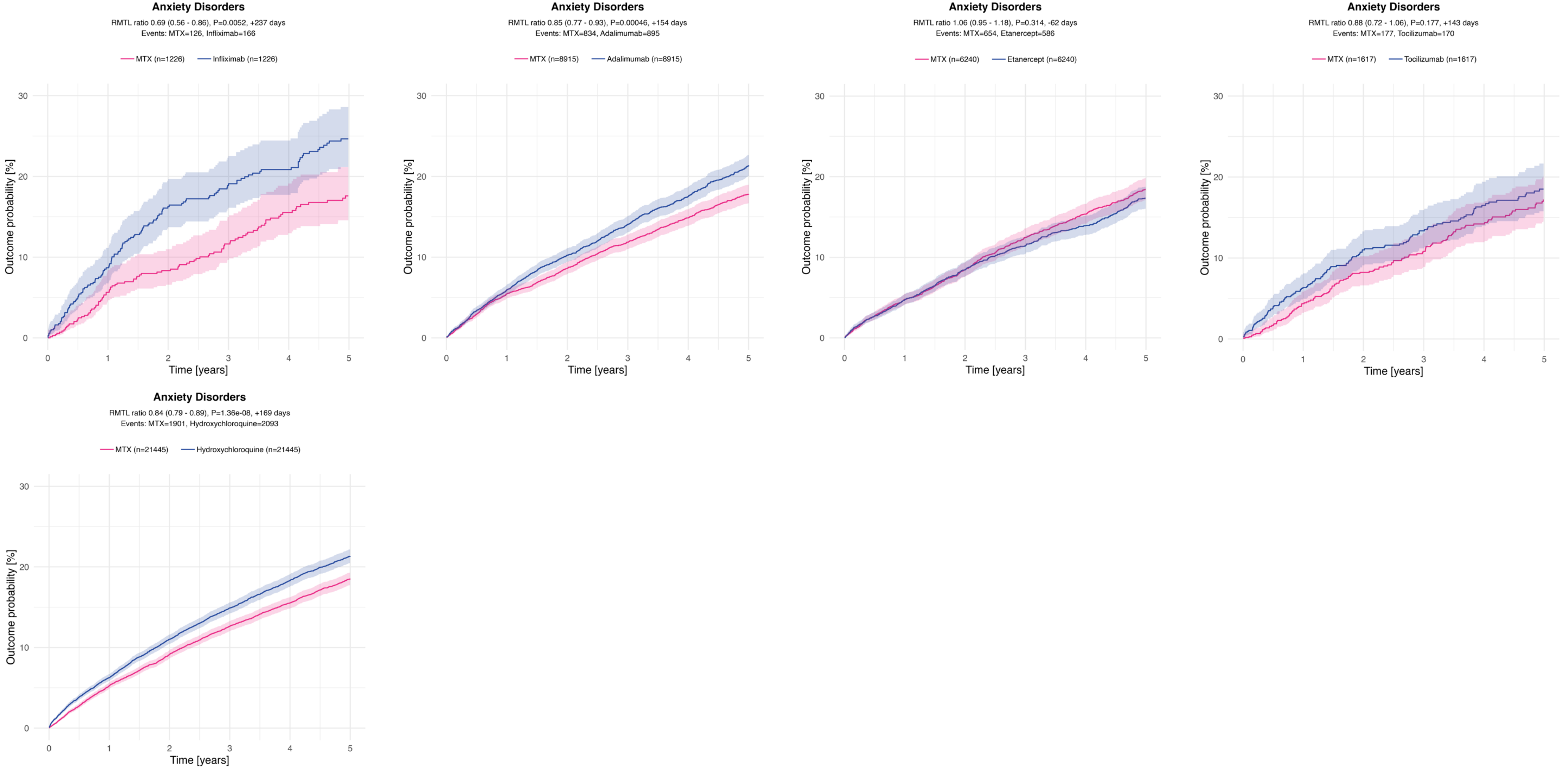


**Supplementary Figure 1D.** Curves representing the Kaplan–Meier estimates of the cumulative incidence of anxiety disorders after initiation of low-dose methotrexate *vs* comparator drugs. Cohort of individuals with rheumatoid arthritis who initiated pharmacological treatment at age ≤45 years. rRMTL <1 indicate that the risk for anxiety disorders is lower after low-dose methotrexate than after the comparator drug, and conversely for rRMTL >1. P value less than Bonferroni corrected critical value for multiple comparison: α = 0.05/44 = 0.0011. Cohort size after propensity score matching: Infliximab (N=1,226), Adalimumab (N=8,915), Etanercept (N=6,240), Tocilizumab (N=1,617), Hydroxychloroquine (N=21,445). rRMTL, restricted mean time lost ratio.

## Supplementary Table S13. Baseline characteristics after propensity score matching low-dose methotrexate *vs* Leflunomide

**Cohort 1. Low-dose methotrexate (N = 3,966) and cohort 2. Leflunomide (N = 3,966)**

**Demographics**

| Cohort | | | Mean ± SD | Patients | % of Cohort | P-Value | Std diff. |
|---|---|---|---|---|---|---|---|
| 1<br>2 | AI | Age at Index | 37.4 +/- 6.9<br>37.3 +/- 7.0 | 3,966<br>3,966 | 100%<br>100% | 0.420 | 0.018 |
| 1<br>2 | F | Female | | 3,312<br>3,297 | 83.5%<br>83.1% | 0.651 | 0.010 |
| 1<br>2 | 2054-5 | Black or African American | | 493<br>489 | 12.4%<br>12.3% | 0.892 | 0.003 |
| 1<br>2 | M | Male | | 654<br>669 | 16.5%<br>16.9% | 0.651 | 0.010 |
| 1<br>2 | 2106-3 | White | | 2,784<br>2,770 | 70.2%<br>69.8% | 0.732 | 0.008 |
| 1<br>2 | 1002-5 | American Indian or Alaska Native | | 37<br>49 | 0.9%<br>1.2% | 0.193 | 0.029 |
| 1<br>2 | UNK | Unknown Race | | 314<br>299 | 7.9%<br>7.5% | 0.528 | 0.014 |
| 1<br>2 | 2076-8 | Native Hawaiian or Other Pacific Islander | | 15<br>16 | 0.4%<br>0.4% | 0.857 | 0.004 |
| 1<br>2 | UN | Unknown Ethnicity | | 725<br>740 | 18.3%<br>18.7% | 0.664 | 0.010 |
| 1<br>2 | 2186-5 | Not Hispanic or Latino | | 2,671<br>2,660 | 67.3%<br>67.1% | 0.792 | 0.006 |
| 1<br>2 | 2135-2 | Hispanic or Latino | | 570<br>566 | 14.4%<br>14.3% | 0.898 | 0.003 |
| 1<br>2 | 2131-1 | Other Race | | 227<br>250 | 5.7%<br>6.3% | 0.277 | 0.024 |
| 1<br>2 | 2028-9 | Asian | | 96<br>93 | 2.4%<br>2.3% | 0.825 | 0.005 |

**Diagnosis**

| Cohort | | | Mean ± SD | Patients | % of Cohort | P-Value | Std diff. |
|---|---|---|---|---|---|---|---|
| 1<br>2 | Z55 | Problems related to education and literacy | | 10<br>10 | 0.3%<br>0.3% | 1 | <0.001 |
| 1<br>2 | Z56 | Problems related to employment and unemployment | | 17<br>18 | 0.4%<br>0.5% | 0.865 | 0.004 |
| 1<br>2 | Z81 | Family history of mental and behavioral disorders | | 20<br>27 | 0.5%<br>0.7% | 0.306 | 0.023 |
| 1<br>2 | E70-E88 | Metabolic disorders | | 883<br>917 | 22.3%<br>23.1% | 0.362 | 0.020 |
| 1<br>2 | F31 | Bipolar disorder | | 109<br>133 | 2.7%<br>3.4% | 0.117 | 0.035 |
| 1<br>2 | F32 | Depressive episode | | 790<br>833 | 19.9%<br>21.0% | 0.231 | 0.027 |
| 1<br>2 | F33 | Major depressive disorder, recurrent | | 171<br>230 | 4.3%<br>5.8% | 0.002 | 0.068 |
| 1<br>2 | X71 | Intentional self-harm by drowning and submersion | | 0<br>0 | 0%<br>0% | -- | -- |

| Cohort | Code | Description | Mean ± SD | Patients | % of Cohort | P-Value | Std diff. |
|---|---|---|---|---|---|---|---|
| 1<br>2 | R45.851 | Suicidal ideations | | 39<br>55 | 1.0%<br>1.4% | 0.097 | 0.037 |
| 1<br>2 | T14.91 | Suicide attempt | | 10<br>10 | 0.3%<br>0.3% | 1 | <0.001 |
| 1<br>2 | X71-X83 | Intentional self-harm | | 10<br>10 | 0.3%<br>0.3% | 1 | <0.001 |
| 1<br>2 | W54.0 | Bitten by dog | | 13<br>10 | 0.3%<br>0.3% | 0.531 | 0.014 |
| 1<br>2 | L60.0 | Ingrowing nail | | 42<br>34 | 1.1%<br>0.9% | 0.356 | 0.021 |
| 1<br>2 | B07 | Viral warts | | 70<br>58 | 1.8%<br>1.5% | 0.285 | 0.024 |
| 1<br>2 | I00-I99 | Diseases of the circulatory system | | 1,414<br>1,394 | 35.7%<br>35.1% | 0.639 | 0.011 |
| 1<br>2 | F41 | Other anxiety disorders | | 879<br>959 | 22.2%<br>24.2% | 0.033 | 0.048 |
| 1<br>2 | F41.1 | Generalized anxiety disorder | | 280<br>327 | 7.1%<br>8.2% | 0.047 | 0.045 |
| 1<br>2 | F40 | Phobic anxiety disorders | | 62<br>62 | 1.6%<br>1.6% | 1 | <0.001 |
| 1<br>2 | F41.8 | Other specified anxiety disorders | | 131<br>159 | 3.3%<br>4.0% | 0.094 | 0.038 |
| 1<br>2 | F41.0 | Panic disorder [episodic paroxysmal anxiety] | | 102<br>118 | 2.6%<br>3.0% | 0.274 | 0.025 |
| 1<br>2 | F41.3 | Other mixed anxiety disorders | | 10<br>10 | 0.3%<br>0.3% | 1 | <0.001 |
| 1<br>2 | F41.9 | Anxiety disorder, unspecified | | 720<br>762 | 18.2%<br>19.2% | 0.226 | 0.027 |
| 1<br>2 | F42 | Obsessive-compulsive disorder | | 20<br>34 | 0.5%<br>0.9% | 0.056 | 0.043 |
| 1<br>2 | F30.2 | Manic episode, severe with psychotic symptoms | | 0<br>0 | 0%<br>0% | -- | -- |
| 1<br>2 | F31.2 | Bipolar disorder, current episode manic severe with psychotic features | | 0<br>10 | 0%<br>0.3% | 0.002 | 0.071 |
| 1<br>2 | F31.5 | Bipolar disorder, current episode depressed, severe, with psychotic features | | 10<br>10 | 0.3%<br>0.3% | 1 | <0.001 |
| 1<br>2 | F32.3 | Major depressive disorder, single episode, severe with psychotic features | | 10<br>10 | 0.3%<br>0.3% | 1 | <0.001 |
| 1<br>2 | F33.3 | Major depressive disorder, recurrent, severe with psychotic symptoms | | 10<br>10 | 0.3%<br>0.3% | 1 | <0.001 |
| **Medication** | | | | | | | |
| Cohort | | | Mean ± SD | Patients | % of Cohort | P-Value | Std diff. |
| 1<br>2 | R01AD | Corticosteroids | | 1,523<br>1,521 | 38.4%<br>38.4% | 0.963 | 0.001 |

## Supplementary Table S14. Baseline characteristics after propensity score matching low-dose methotrexate *vs* Sulfasalazine

**Cohort 1 Low-dose methotrexate (N = 6,358) and cohort 2 (N = 6,358) characteristics after propensity score matching**

**Demographics**

| Cohort | | | Mean ± SD | Patients | % of Cohort | P-Value | Std diff. |
|---|---|---|---|---|---|---|---|
| 1<br>2 | AI | Age at Index | 34.6 +/- 7.9<br>34.9 +/- 7.4 | 6,358<br>6,358 | 100%<br>100% | 0.078 | 0.031 |
| 1<br>2 | F | Female | | 5,169<br>5,155 | 81.3%<br>81.1% | 0.751 | 0.006 |
| 1<br>2 | 2054-5 | Black or African American | | 606<br>631 | 9.5%<br>9.9% | 0.454 | 0.013 |
| 1<br>2 | M | Male | | 1,186<br>1,199 | 18.7%<br>18.9% | 0.768 | 0.005 |
| 1<br>2 | 2106-3 | White | | 4,684<br>4,616 | 73.7%<br>72.6% | 0.174 | 0.024 |
| 1<br>2 | 1002-5 | American Indian or Alaska Native | | 72<br>70 | 1.1%<br>1.1% | 0.866 | 0.003 |
| 1<br>2 | UNK | Unknown Race | | 425<br>441 | 6.7%<br>6.9% | 0.573 | 0.010 |
| 1<br>2 | 2076-8 | Native Hawaiian or Other Pacific Islander | | 45<br>53 | 0.7%<br>0.8% | 0.417 | 0.014 |
| 1<br>2 | UN | Unknown Ethnicity | | 1,395<br>1,365 | 21.9%<br>21.5% | 0.519 | 0.011 |
| 1<br>2 | 2186-5 | Not Hispanic or Latino | | 4,062<br>4,041 | 63.9%<br>63.6% | 0.699 | 0.007 |
| 1<br>2 | 2135-2 | Hispanic or Latino | | 901<br>952 | 14.2%<br>15.0% | 0.200 | 0.023 |
| 1<br>2 | 2131-1 | Other Race | | 289<br>304 | 4.5%<br>4.8% | 0.528 | 0.011 |
| 1<br>2 | 2028-9 | Asian | | 237<br>243 | 3.7%<br>3.8% | 0.780 | 0.005 |

**Diagnosis**

| Cohort | | | Mean ± SD | Patients | % of Cohort | P-Value | Std diff. |
|---|---|---|---|---|---|---|---|
| 1<br>2 | Z55 | Problems related to education and literacy | | 10<br>10 | 0.2%<br>0.2% | 1 | <0.001 |
| 1<br>2 | Z56 | Problems related to employment and unemployment | | 18<br>22 | 0.3%<br>0.3% | 0.526 | 0.011 |
| 1<br>2 | Z81 | Family history of mental and behavioral disorders | | 27<br>35 | 0.4%<br>0.6% | 0.308 | 0.018 |
| 1<br>2 | E70-E88 | Metabolic disorders | | 1,223<br>1,285 | 19.2%<br>20.2% | 0.167 | 0.025 |
| 1<br>2 | F31 | Bipolar disorder | | 212<br>226 | 3.3%<br>3.6% | 0.496 | 0.012 |
| 1<br>2 | F32 | Depressive episode | | 1,127<br>1,202 | 17.7%<br>18.9% | 0.086 | 0.031 |
| 1<br>2 | F33 | Major depressive disorder, recurrent | | 295<br>345 | 4.6%<br>5.4% | 0.043 | 0.036 |
| 1<br>2 | X71 | Intentional self-harm by drowning and submersion | | 0<br>0 | 0%<br>0% | -- | -- |

| Cohort | Code | Description | Mean ± SD | Patients | % of Cohort | P-Value | Std diff. |
|---|---|---|---|---|---|---|---|
| 1<br>2 | R45.851 | Suicidal ideations | | 85<br>98 | 1.3%<br>1.5% | 0.333 | 0.017 |
| 1<br>2 | T14.91 | Suicide attempt | | 10<br>10 | 0.2%<br>0.2% | 1 | <0.001 |
| 1<br>2 | X71-X83 | Intentional self-harm | | 10<br>11 | 0.2%<br>0.2% | 0.827 | 0.004 |
| 1<br>2 | W54.0 | Bitten by dog | | 27<br>16 | 0.4%<br>0.3% | 0.093 | 0.030 |
| 1<br>2 | L60.0 | Ingrowing nail | | 69<br>67 | 1.1%<br>1.1% | 0.863 | 0.003 |
| 1<br>2 | B07 | Viral warts | | 101<br>112 | 1.6%<br>1.8% | 0.447 | 0.013 |
| 1<br>2 | I00-I99 | Diseases of the circulatory system | | 1,736<br>1,798 | 27.3%<br>28.3% | 0.220 | 0.022 |
| 1<br>2 | F41 | Other anxiety disorders | | 1,376<br>1,478 | 21.6%<br>23.2% | 0.030 | 0.038 |
| 1<br>2 | F41.1 | Generalized anxiety disorder | | 405<br>477 | 6.4%<br>7.5% | 0.012 | 0.045 |
| 1<br>2 | F40 | Phobic anxiety disorders | | 54<br>71 | 0.8%<br>1.1% | 0.126 | 0.027 |
| 1<br>2 | F41.8 | Other specified anxiety disorders | | 193<br>236 | 3.0%<br>3.7% | 0.035 | 0.037 |
| 1<br>2 | F41.0 | Panic disorder [episodic paroxysmal anxiety] | | 144<br>172 | 2.3%<br>2.7% | 0.111 | 0.028 |
| 1<br>2 | F41.3 | Other mixed anxiety disorders | | 10<br>10 | 0.2%<br>0.2% | 1 | <0.001 |
| 1<br>2 | F41.9 | Anxiety disorder, unspecified | | 1,152<br>1,198 | 18.1%<br>18.8% | 0.293 | 0.019 |
| 1<br>2 | F42 | Obsessive-compulsive disorder | | 62<br>63 | 1.0%<br>1.0% | 0.928 | 0.002 |
| 1<br>2 | F30.2 | Manic episode, severe with psychotic symptoms | | 0<br>0 | 0%<br>0% | -- | -- |
| 1<br>2 | F31.2 | Bipolar disorder, current episode manic severe with psychotic features | | 0<br>0 | 0%<br>0% | -- | -- |
| 1<br>2 | F31.5 | Bipolar disorder, current episode depressed, severe, with psychotic features | | 10<br>10 | 0.2%<br>0.2% | 1 | <0.001 |
| 1<br>2 | F32.3 | Major depressive disorder, single episode, severe with psychotic features | | 10<br>10 | 0.2%<br>0.2% | 1 | <0.001 |
| 1<br>2 | F33.3 | Major depressive disorder, recurrent, severe with psychotic symptoms | | 10<br>10 | 0.2%<br>0.2% | 1 | <0.001 |
| **Medication** | | | | | | | |
| Cohort | | | Mean ± SD | Patients | % of Cohort | P-Value | Std diff. |
| 1<br>2 | R01AD | Corticosteroids | | 2,232<br>2,305 | 35.1%<br>36.3% | 0.177 | 0.024 |

## Supplementary Table S15. Baseline characteristics after propensity score matching low-dose methotrexate *vs* Minocycline

**Cohort 1. Low-dose methotrexate (N = 1,161) and cohort 2. Minocycline (N = 1,161)**

**Demographics**

| Cohort | | | Mean ± SD | Patients | % of Cohort | P-Value | Std diff. |
|---|---|---|---|---|---|---|---|
| 1<br>2 | AI | Age at Index | 33.9 +/- 8.7<br>34.3 +/- 8.4 | 1,161<br>1,161 | 100%<br>100% | 0.281 | 0.045 |
| 1<br>2 | F | Female | | 954<br>940 | 82.2%<br>81.0% | 0.454 | 0.031 |
| 1<br>2 | 2054-5 | Black or African American | | 155<br>149 | 13.4%<br>12.8% | 0.712 | 0.015 |
| 1<br>2 | M | Male | | 206<br>220 | 17.7%<br>18.9% | 0.453 | 0.031 |
| 1<br>2 | 2106-3 | White | | 863<br>836 | 74.3%<br>72.0% | 0.206 | 0.053 |
| 1<br>2 | 1002-5 | American Indian or Alaska Native | | 10<br>14 | 0.9%<br>1.2% | 0.412 | 0.034 |
| 1<br>2 | UNK | Unknown Race | | 49<br>65 | 4.2%<br>5.6% | 0.124 | 0.064 |
| 1<br>2 | 2076-8 | Native Hawaiian or Other Pacific Islander | | 10<br>10 | 0.9%<br>0.9% | 1 | <0.001 |
| 1<br>2 | UN | Unknown Ethnicity | | 231<br>218 | 19.9%<br>18.8% | 0.495 | 0.028 |
| 1<br>2 | 2186-5 | Not Hispanic or Latino | | 858<br>845 | 73.9%<br>72.8% | 0.542 | 0.025 |
| 1<br>2 | 2135-2 | Hispanic or Latino | | 72<br>98 | 6.2%<br>8.4% | 0.038 | 0.086 |
| 1<br>2 | 2131-1 | Other Race | | 52<br>59 | 4.5%<br>5.1% | 0.496 | 0.028 |
| 1<br>2 | 2028-9 | Asian | | 30<br>34 | 2.6%<br>2.9% | 0.612 | 0.021 |

**Diagnosis**

| Cohort | | | Mean ± SD | Patients | % of Cohort | P-Value | Std diff. |
|---|---|---|---|---|---|---|---|
| 1<br>2 | Z55 | Problems related to education and literacy | | 0<br>10 | 0%<br>0.9% | 0.002 | 0.132 |
| 1<br>2 | Z56 | Problems related to employment and unemployment | | 13<br>10 | 1.1%<br>0.9% | 0.530 | 0.026 |
| 1<br>2 | Z81 | Family history of mental and behavioral disorders | | 14<br>13 | 1.2%<br>1.1% | 0.847 | 0.008 |
| 1<br>2 | E70-E88 | Metabolic disorders | | 333<br>362 | 28.7%<br>31.2% | 0.189 | 0.055 |
| 1<br>2 | F31 | Bipolar disorder | | 50<br>65 | 4.3%<br>5.6% | 0.151 | 0.060 |
| 1<br>2 | F32 | Depressive episode | | 312<br>339 | 26.9%<br>29.2% | 0.212 | 0.052 |
| 1<br>2 | F33 | Major depressive disorder, recurrent | | 85<br>100 | 7.3%<br>8.6% | 0.250 | 0.048 |

| | | | | | | | |
|---|---|---|---|---|---|---|---|
| 1<br>2 | X71 | Intentional self-harm by drowning and submersion | | 0<br>0 | 0%<br>0% | -- | -- |
| 1<br>2 | R45.851 | Suicidal ideations | | 33<br>25 | 2.8%<br>2.2% | 0.287 | 0.044 |
| 1<br>2 | T14.91 | Suicide attempt | | 10<br>10 | 0.9%<br>0.9% | 1 | <0.001 |
| 1<br>2 | X71-X83 | Intentional self-harm | | 10<br>10 | 0.9%<br>0.9% | 1 | <0.001 |
| 1<br>2 | W54.0 | Bitten by dog | | 10<br>10 | 0.9%<br>0.9% | 1 | <0.001 |
| 1<br>2 | L60.0 | Ingrowing nail | | 20<br>19 | 1.7%<br>1.6% | 0.872 | 0.007 |
| 1<br>2 | B07 | Viral warts | | 25<br>30 | 2.2%<br>2.6% | 0.495 | 0.028 |
| 1<br>2 | I00-I99 | Diseases of the circulatory system | | 472<br>480 | 40.7%<br>41.3% | 0.736 | 0.014 |
| 1<br>2 | F41 | Other anxiety disorders | | 354<br>374 | 30.5%<br>32.2% | 0.371 | 0.037 |
| 1<br>2 | F41.1 | Generalized anxiety disorder | | 123<br>142 | 10.6%<br>12.2% | 0.215 | 0.051 |
| 1<br>2 | F40 | Phobic anxiety disorders | | 20<br>22 | 1.7%<br>1.9% | 0.755 | 0.013 |
| 1<br>2 | F41.8 | Other specified anxiety disorders | | 68<br>75 | 5.9%<br>6.5% | 0.546 | 0.025 |
| 1<br>2 | F41.0 | Panic disorder [episodic paroxysmal anxiety] | | 49<br>62 | 4.2%<br>5.3% | 0.206 | 0.053 |
| 1<br>2 | F41.3 | Other mixed anxiety disorders | | 10<br>10 | 0.9%<br>0.9% | 1 | <0.001 |
| 1<br>2 | F41.9 | Anxiety disorder, unspecified | | 315<br>310 | 27.1%<br>26.7% | 0.815 | 0.010 |
| 1<br>2 | F42 | Obsessive-compulsive disorder | | 12<br>15 | 1.0%<br>1.3% | 0.561 | 0.024 |
| 1<br>2 | F30.2 | Manic episode, severe with psychotic symptoms | | 0<br>0 | 0%<br>0% | -- | -- |
| 1<br>2 | F31.2 | Bipolar disorder, current episode manic severe with psychotic features | | 10<br>10 | 0.9%<br>0.9% | 1 | <0.001 |
| 1<br>2 | F31.5 | Bipolar disorder, current episode depressed, severe, with psychotic features | | 10<br>0 | 0.9%<br>0% | 0.002 | 0.132 |
| 1<br>2 | F32.3 | Major depressive disorder, single episode, severe with psychotic features | | 0<br>0 | 0%<br>0% | -- | -- |
| 1<br>2 | F33.3 | Major depressive disorder, recurrent, severe with psychotic symptoms | | 10<br>10 | 0.9%<br>0.9% | 1 | <0.001 |
| **Medication** | | | | | | | |
| Cohort | | | Mean ± SD | Patients | % of Cohort | P-Value | Std diff. |
| 1<br>2 | R01AD | Corticosteroids | | 605<br>605 | 52.1%<br>52.1% | 1 | <0.001 |

## Supplementary Table S16. Baseline characteristics after propensity score matching low-dose methotrexate *vs* Abatacept

| Cohort 1. Low-dose methotrexate (N = 2,219) and cohort 2. Abatacept (N = 2,219) | | | | | | | |
|---|---|---|---|---|---|---|---|
| **Demographics** | | | | | | | |
| Cohort | | | Mean ± SD | Patients | % of Cohort | P-Value | Std diff. |
| 1<br>2 | AI | Age at Index | 35.6 +/- 7.8<br>35.8 +/- 7.7 | 2,219<br>2,219 | 100%<br>100% | 0.558 | 0.018 |
| 1<br>2 | F | Female | | 1,978<br>1,961 | 89.1%<br>88.4% | 0.419 | 0.024 |
| 1<br>2 | 2054-5 | Black or African American | | 253<br>262 | 11.4%<br>11.8% | 0.673 | 0.013 |
| 1<br>2 | M | Male | | 241<br>258 | 10.9%<br>11.6% | 0.419 | 0.024 |
| 1<br>2 | 2106-3 | White | | 1,615<br>1,599 | 72.8%<br>72.1% | 0.591 | 0.016 |
| 1<br>2 | 1002-5 | American Indian or Alaska Native | | 23<br>27 | 1.0%<br>1.2% | 0.569 | 0.017 |
| 1<br>2 | UNK | Unknown Race | | 170<br>167 | 7.7%<br>7.5% | 0.865 | 0.005 |
| 1<br>2 | 2076-8 | Native Hawaiian or Other Pacific Islander | | 10<br>10 | 0.5%<br>0.5% | 1 | <0.001 |
| 1<br>2 | UN | Unknown Ethnicity | | 391<br>394 | 17.6%<br>17.8% | 0.906 | 0.004 |
| 1<br>2 | 2186-5 | Not Hispanic or Latino | | 1,558<br>1,554 | 70.2%<br>70.0% | 0.896 | 0.004 |
| 1<br>2 | 2135-2 | Hispanic or Latino | | 270<br>271 | 12.2%<br>12.2% | 0.963 | 0.001 |
| 1<br>2 | 2131-1 | Other Race | | 108<br>113 | 4.9%<br>5.1% | 0.730 | 0.010 |
| 1<br>2 | 2028-9 | Asian | | 41<br>43 | 1.8%<br>1.9% | 0.826 | 0.007 |
| **Diagnosis** | | | | | | | |
| Cohort | | | Mean ± SD | Patients | % of Cohort | P-Value | Std diff. |
| 1<br>2 | Z55 | Problems related to education and literacy | | 10<br>10 | 0.5%<br>0.5% | 1 | <0.001 |
| 1<br>2 | Z56 | Problems related to employment and unemployment | | 10<br>11 | 0.5%<br>0.5% | 0.827 | 0.007 |
| 1<br>2 | Z81 | Family history of mental and behavioral disorders | | 10<br>10 | 0.5%<br>0.5% | 1 | <0.001 |
| 1<br>2 | E70-E88 | Metabolic disorders | | 446<br>481 | 20.1%<br>21.7% | 0.196 | 0.039 |
| 1<br>2 | F31 | Bipolar disorder | | 47<br>75 | 2.1%<br>3.4% | 0.010 | 0.077 |
| 1<br>2 | F32 | Depressive episode | | 402<br>439 | 18.1%<br>19.8% | 0.156 | 0.043 |
| 1<br>2 | F33 | Major depressive disorder, recurrent | | 83<br>123 | 3.7%<br>5.5% | 0.004 | 0.086 |

| | | | | | | |
|---|---|---|---|---|---|---|
| 1<br>2 | X71 | Intentional self-harm by drowning and submersion | 0<br>0 | 0%<br>0% | -- | -- |
| 1<br>2 | R45.851 | Suicidal ideations | 16<br>21 | 0.7%<br>0.9% | 0.409 | 0.025 |
| 1<br>2 | T14.91 | Suicide attempt | 10<br>10 | 0.5%<br>0.5% | 1 | <0.001 |
| 1<br>2 | X71-X83 | Intentional self-harm | 10<br>10 | 0.5%<br>0.5% | 1 | <0.001 |
| 1<br>2 | W54.0 | Bitten by dog | 10<br>10 | 0.5%<br>0.5% | 1 | <0.001 |
| 1<br>2 | L60.0 | Ingrowing nail | 18<br>29 | 0.8%<br>1.3% | 0.107 | 0.048 |
| 1<br>2 | B07 | Viral warts | 38<br>31 | 1.7%<br>1.4% | 0.396 | 0.026 |
| 1<br>2 | I00-I99 | Diseases of the circulatory system | 727<br>754 | 32.8%<br>34.0% | 0.390 | 0.026 |
| 1<br>2 | F41 | Other anxiety disorders | 473<br>528 | 21.3%<br>23.8% | 0.048 | 0.059 |
| 1<br>2 | F41.1 | Generalized anxiety disorder | 119<br>163 | 5.4%<br>7.3% | 0.007 | 0.081 |
| 1<br>2 | F40 | Phobic anxiety disorders | 17<br>24 | 0.8%<br>1.1% | 0.272 | 0.033 |
| 1<br>2 | F41.8 | Other specified anxiety disorders | 81<br>91 | 3.7%<br>4.1% | 0.437 | 0.023 |
| 1<br>2 | F41.0 | Panic disorder [episodic paroxysmal anxiety] | 56<br>63 | 2.5%<br>2.8% | 0.515 | 0.020 |
| 1<br>2 | F41.3 | Other mixed anxiety disorders | 10<br>10 | 0.5%<br>0.5% | 1 | <0.001 |
| 1<br>2 | F41.9 | Anxiety disorder, unspecified | 396<br>441 | 17.8%<br>19.9% | 0.084 | 0.052 |
| 1<br>2 | F42 | Obsessive-compulsive disorder | 12<br>19 | 0.5%<br>0.9% | 0.207 | 0.038 |
| 1<br>2 | F30.2 | Manic episode, severe with psychotic symptoms | 0<br>0 | 0%<br>0% | -- | -- |
| 1<br>2 | F31.2 | Bipolar disorder, current episode manic severe with psychotic features | 10<br>10 | 0.5%<br>0.5% | 1 | <0.001 |
| 1<br>2 | F31.5 | Bipolar disorder, current episode depressed, severe, with psychotic features | 10<br>10 | 0.5%<br>0.5% | 1 | <0.001 |
| 1<br>2 | F32.3 | Major depressive disorder, single episode, severe with psychotic features | 0<br>10 | 0%<br>0.5% | 0.002 | 0.095 |
| 1<br>2 | F33.3 | Major depressive disorder, recurrent, severe with psychotic symptoms | 10<br>10 | 0.5%<br>0.5% | 1 | <0.001 |

**Medication**

| Cohort | | | Mean ± SD | Patients | % of Cohort | P-Value | Std diff. |
|---|---|---|---|---|---|---|---|
| 1<br>2 | R01AD | Corticosteroids | | 929<br>918 | 41.9%<br>41.4% | 0.738 | 0.010 |

## Supplementary Table S17. Baseline characteristics after propensity score matching low-dose methotrexate *vs* Tofacitinib

| Cohort 1. Low-dose methotrexate (N = 2,269) and cohort 2. Tofacitinib (N = 2,269) | | | | | | | |
|---|---|---|---|---|---|---|---|
| **Demographics** | | | | | | | |
| Cohort | | | Mean ± SD | Patients | % of Cohort | P-Value | Std diff. |
| 1<br>2 | AI | Age at Index | 35.7 +/- 7.8<br>35.5 +/- 7.9 | 2,269<br>2,269 | 100%<br>100% | 0.543 | 0.018 |
| 1<br>2 | F | Female | | 1,898<br>1,906 | 83.6%<br>84.0% | 0.747 | 0.010 |
| 1<br>2 | 2054-5 | Black or African American | | 214<br>221 | 9.4%<br>9.7% | 0.724 | 0.010 |
| 1<br>2 | M | Male | | 371<br>361 | 16.4%<br>15.9% | 0.687 | 0.012 |
| 1<br>2 | 2106-3 | White | | 1,663<br>1,622 | 73.3%<br>71.5% | 0.173 | 0.040 |
| 1<br>2 | 1002-5 | American Indian or Alaska Native | | 22<br>26 | 1.0%<br>1.1% | 0.562 | 0.017 |
| 1<br>2 | UNK | Unknown Race | | 176<br>181 | 7.8%<br>8.0% | 0.783 | 0.008 |
| 1<br>2 | 2076-8 | Native Hawaiian or Other Pacific Islander | | 10<br>10 | 0.4%<br>0.4% | 1 | <0.001 |
| 1<br>2 | UN | Unknown Ethnicity | | 369<br>358 | 16.3%<br>15.8% | 0.656 | 0.013 |
| 1<br>2 | 2186-5 | Not Hispanic or Latino | | 1,649<br>1,647 | 72.7%<br>72.6% | 0.947 | 0.002 |
| 1<br>2 | 2135-2 | Hispanic or Latino | | 251<br>264 | 11.1%<br>11.6% | 0.543 | 0.018 |
| 1<br>2 | 2131-1 | Other Race | | 138<br>153 | 6.1%<br>6.7% | 0.363 | 0.027 |
| 1<br>2 | 2028-9 | Asian | | 51<br>61 | 2.2%<br>2.7% | 0.339 | 0.028 |
| **Diagnosis** | | | | | | | |
| Cohort | | | Mean ± SD | Patients | % of Cohort | P-Value | Std diff. |
| 1<br>2 | Z55 | Problems related to education and literacy | | 10<br>10 | 0.4%<br>0.4% | 1 | <0.001 |
| 1<br>2 | Z56 | Problems related to employment and unemployment | | 10<br>10 | 0.4%<br>0.4% | 1 | <0.001 |
| 1<br>2 | Z81 | Family history of mental and behavioral disorders | | 14<br>15 | 0.6%<br>0.7% | 0.852 | 0.006 |
| 1<br>2 | E70-E88 | Metabolic disorders | | 486<br>492 | 21.4%<br>21.7% | 0.829 | 0.006 |
| 1<br>2 | F31 | Bipolar disorder | | 71<br>79 | 3.1%<br>3.5% | 0.507 | 0.020 |
| 1<br>2 | F32 | Depressive episode | | 411<br>462 | 18.1%<br>20.4% | 0.055 | 0.057 |
| 1<br>2 | F33 | Major depressive disorder, recurrent | | 109<br>130 | 4.8%<br>5.7% | 0.163 | 0.041 |

| | | | | | | | |
|---|---|---|---|---|---|---|---|
| 1<br>2 | X71 | Intentional self-harm by drowning and submersion | | 0<br>0 | 0%<br>0% | -- | -- |
| 1<br>2 | R45.851 | Suicidal ideations | | 34<br>33 | 1.5%<br>1.5% | 0.902 | 0.004 |
| 1<br>2 | T14.91 | Suicide attempt | | 0<br>10 | 0%<br>0.4% | 0.002 | 0.094 |
| 1<br>2 | X71-X83 | Intentional self-harm | | 10<br>10 | 0.4%<br>0.4% | 1 | <0.001 |
| 1<br>2 | W54.0 | Bitten by dog | | 10<br>10 | 0.4%<br>0.4% | 1 | <0.001 |
| 1<br>2 | L60.0 | Ingrowing nail | | 18<br>24 | 0.8%<br>1.1% | 0.352 | 0.028 |
| 1<br>2 | B07 | Viral warts | | 38<br>42 | 1.7%<br>1.9% | 0.652 | 0.013 |
| 1<br>2 | I00-I99 | Diseases of the circulatory system | | 805<br>775 | 35.5%<br>34.2% | 0.350 | 0.028 |
| 1<br>2 | F41 | Other anxiety disorders | | 506<br>576 | 22.3%<br>25.4% | 0.015 | 0.072 |
| 1<br>2 | F41.1 | Generalized anxiety disorder | | 120<br>162 | 5.3%<br>7.1% | 0.010 | 0.077 |
| 1<br>2 | F40 | Phobic anxiety disorders | | 28<br>32 | 1.2%<br>1.4% | 0.603 | 0.015 |
| 1<br>2 | F41.8 | Other specified anxiety disorders | | 82<br>98 | 3.6%<br>4.3% | 0.224 | 0.036 |
| 1<br>2 | F41.0 | Panic disorder [episodic paroxysmal anxiety] | | 43<br>54 | 1.9%<br>2.4% | 0.259 | 0.034 |
| 1<br>2 | F41.3 | Other mixed anxiety disorders | | 10<br>10 | 0.4%<br>0.4% | 1 | <0.001 |
| 1<br>2 | F41.9 | Anxiety disorder, unspecified | | 425<br>480 | 18.7%<br>21.2% | 0.041 | 0.061 |
| 1<br>2 | F42 | Obsessive-compulsive disorder | | 20<br>14 | 0.9%<br>0.6% | 0.302 | 0.031 |
| 1<br>2 | F30.2 | Manic episode, severe with psychotic symptoms | | 0<br>0 | 0%<br>0% | -- | -- |
| 1<br>2 | F31.2 | Bipolar disorder, current episode manic severe with psychotic features | | 0<br>10 | 0%<br>0.4% | 0.002 | 0.094 |
| 1<br>2 | F31.5 | Bipolar disorder, current episode depressed, severe, with psychotic features | | 10<br>0 | 0.4%<br>0% | 0.002 | 0.094 |
| 1<br>2 | F32.3 | Major depressive disorder, single episode, severe with psychotic features | | 0<br>10 | 0%<br>0.4% | 0.002 | 0.094 |
| 1<br>2 | F33.3 | Major depressive disorder, recurrent, severe with psychotic symptoms | | 0<br>10 | 0%<br>0.4% | 0.002 | 0.094 |

**Medication**

| Cohort | | | Mean ± SD | Patients | % of Cohort | P-Value | Std diff. |
|---|---|---|---|---|---|---|---|
| 1<br>2 | R01AD | Corticosteroids | | 992<br>990 | 43.7%<br>43.6% | 0.952 | 0.002 |

## Supplementary Table S18. Baseline characteristics after propensity score matching low-dose methotrexate *vs* Upadacitinib

| Cohort 1. Low-dose methotrexate (N = 1,403) and cohort 2. Upadacitinib (N = 1,403) | | | | | | | |
|---|---|---|---|---|---|---|---|
| **Demographics** | | | | | | | |
| Cohort | | | Mean ± SD | Patients | % of Cohort | P-Value | Std diff. |
| 1<br>2 | AI | Age at Index | 35.7 +/- 7.6<br>35.7 +/- 7.4 | 1,403<br>1,403 | 100%<br>100% | 0.814 | 0.009 |
| 1<br>2 | F | Female | | 1,176<br>1,166 | 83.8%<br>83.1% | 0.611 | 0.019 |
| 1<br>2 | 2054-5 | Black or African American | | 140<br>136 | 10.0%<br>9.7% | 0.800 | 0.010 |
| 1<br>2 | M | Male | | 227<br>236 | 16.2%<br>16.8% | 0.647 | 0.017 |
| 1<br>2 | 2106-3 | White | | 1,050<br>1,036 | 74.8%<br>73.8% | 0.545 | 0.023 |
| 1<br>2 | 1002-5 | American Indian or Alaska Native | | 23<br>22 | 1.6%<br>1.6% | 0.881 | 0.006 |
| 1<br>2 | UNK | Unknown Race | | 97<br>102 | 6.9%<br>7.3% | 0.713 | 0.014 |
| 1<br>2 | 2076-8 | Native Hawaiian or Other Pacific Islander | | 10<br>10 | 0.7%<br>0.7% | 1 | <0.001 |
| 1<br>2 | UN | Unknown Ethnicity | | 298<br>286 | 21.2%<br>20.4% | 0.577 | 0.021 |
| 1<br>2 | 2186-5 | Not Hispanic or Latino | | 976<br>968 | 69.6%<br>69.0% | 0.743 | 0.012 |
| 1<br>2 | 2135-2 | Hispanic or Latino | | 129<br>149 | 9.2%<br>10.6% | 0.206 | 0.048 |
| 1<br>2 | 2131-1 | Other Race | | 37<br>49 | 2.6%<br>3.5% | 0.189 | 0.050 |
| 1<br>2 | 2028-9 | Asian | | 52<br>53 | 3.7%<br>3.8% | 0.921 | 0.004 |
| **Diagnosis** | | | | | | | |
| Cohort | | | Mean ± SD | Patients | % of Cohort | P-Value | Std diff. |
| 1<br>2 | Z55 | Problems related to education and literacy | | 10<br>10 | 0.7%<br>0.7% | 1 | <0.001 |
| 1<br>2 | Z56 | Problems related to employment and unemployment | | 10<br>10 | 0.7%<br>0.7% | 1 | <0.001 |
| 1<br>2 | Z81 | Family history of mental and behavioral disorders | | 14<br>13 | 1.0%<br>0.9% | 0.847 | 0.007 |
| 1<br>2 | E70-E88 | Metabolic disorders | | 325<br>353 | 23.2%<br>25.2% | 0.217 | 0.047 |
| 1<br>2 | F31 | Bipolar disorder | | 57<br>64 | 4.1%<br>4.6% | 0.515 | 0.025 |
| 1<br>2 | F32 | Depressive episode | | 306<br>321 | 21.8%<br>22.9% | 0.497 | 0.026 |
| 1<br>2 | F33 | Major depressive disorder, recurrent | | 95<br>118 | 6.8%<br>8.4% | 0.101 | 0.062 |

| | | | | | | | |
|---|---|---|---|---|---|---|---|
| 1<br>2 | X71 | Intentional self-harm by drowning and submersion | | 0<br>0 | 0%<br>0% | -- | -- |
| 1<br>2 | R45.851 | Suicidal ideations | | 26<br>27 | 1.9%<br>1.9% | 0.890 | 0.005 |
| 1<br>2 | T14.91 | Suicide attempt | | 10<br>10 | 0.7%<br>0.7% | 1 | <0.001 |
| 1<br>2 | X71-X83 | Intentional self-harm | | 0<br>0 | 0%<br>0% | -- | -- |
| 1<br>2 | W54.0 | Bitten by dog | | 10<br>10 | 0.7%<br>0.7% | 1 | <0.001 |
| 1<br>2 | L60.0 | Ingrowing nail | | 16<br>13 | 1.1%<br>0.9% | 0.575 | 0.021 |
| 1<br>2 | B07 | Viral warts | | 23<br>34 | 1.6%<br>2.4% | 0.141 | 0.056 |
| 1<br>2 | I00-I99 | Diseases of the circulatory system | | 471<br>473 | 33.6%<br>33.7% | 0.936 | 0.003 |
| 1<br>2 | F41 | Other anxiety disorders | | 408<br>433 | 29.1%<br>30.9% | 0.303 | 0.039 |
| 1<br>2 | F41.1 | Generalized anxiety disorder | | 115<br>139 | 8.2%<br>9.9% | 0.114 | 0.060 |
| 1<br>2 | F40 | Phobic anxiety disorders | | 21<br>26 | 1.5%<br>1.9% | 0.462 | 0.028 |
| 1<br>2 | F41.8 | Other specified anxiety disorders | | 83<br>88 | 5.9%<br>6.3% | 0.693 | 0.015 |
| 1<br>2 | F41.0 | Panic disorder [episodic paroxysmal anxiety] | | 53<br>47 | 3.8%<br>3.3% | 0.541 | 0.023 |
| 1<br>2 | F41.3 | Other mixed anxiety disorders | | 0<br>10 | 0%<br>0.7% | 0.002 | 0.120 |
| 1<br>2 | F41.9 | Anxiety disorder, unspecified | | 344<br>355 | 24.5%<br>25.3% | 0.631 | 0.018 |
| 1<br>2 | F42 | Obsessive-compulsive disorder | | 16<br>19 | 1.1%<br>1.4% | 0.610 | 0.019 |
| 1<br>2 | F30.2 | Manic episode, severe with psychotic symptoms | | 0<br>0 | 0%<br>0% | -- | -- |
| 1<br>2 | F31.2 | Bipolar disorder, current episode manic severe with psychotic features | | 0<br>0 | 0%<br>0% | -- | -- |
| 1<br>2 | F31.5 | Bipolar disorder, current episode depressed, severe, with psychotic features | | 10<br>10 | 0.7%<br>0.7% | 1 | <0.001 |
| 1<br>2 | F32.3 | Major depressive disorder, single episode, severe with psychotic features | | 10<br>10 | 0.7%<br>0.7% | 1 | <0.001 |
| 1<br>2 | F33.3 | Major depressive disorder, recurrent, severe with psychotic symptoms | | 10<br>10 | 0.7%<br>0.7% | 1 | <0.001 |
| **Medication** | | | | | | | |
| Cohort | | | Mean ± SD | Patients | % of Cohort | P-Value | Std diff. |
| 1<br>2 | R01AD | Corticosteroids | | 711<br>688 | 50.7%<br>49.0% | 0.385 | 0.033 |

# Supplementary Figure 2A

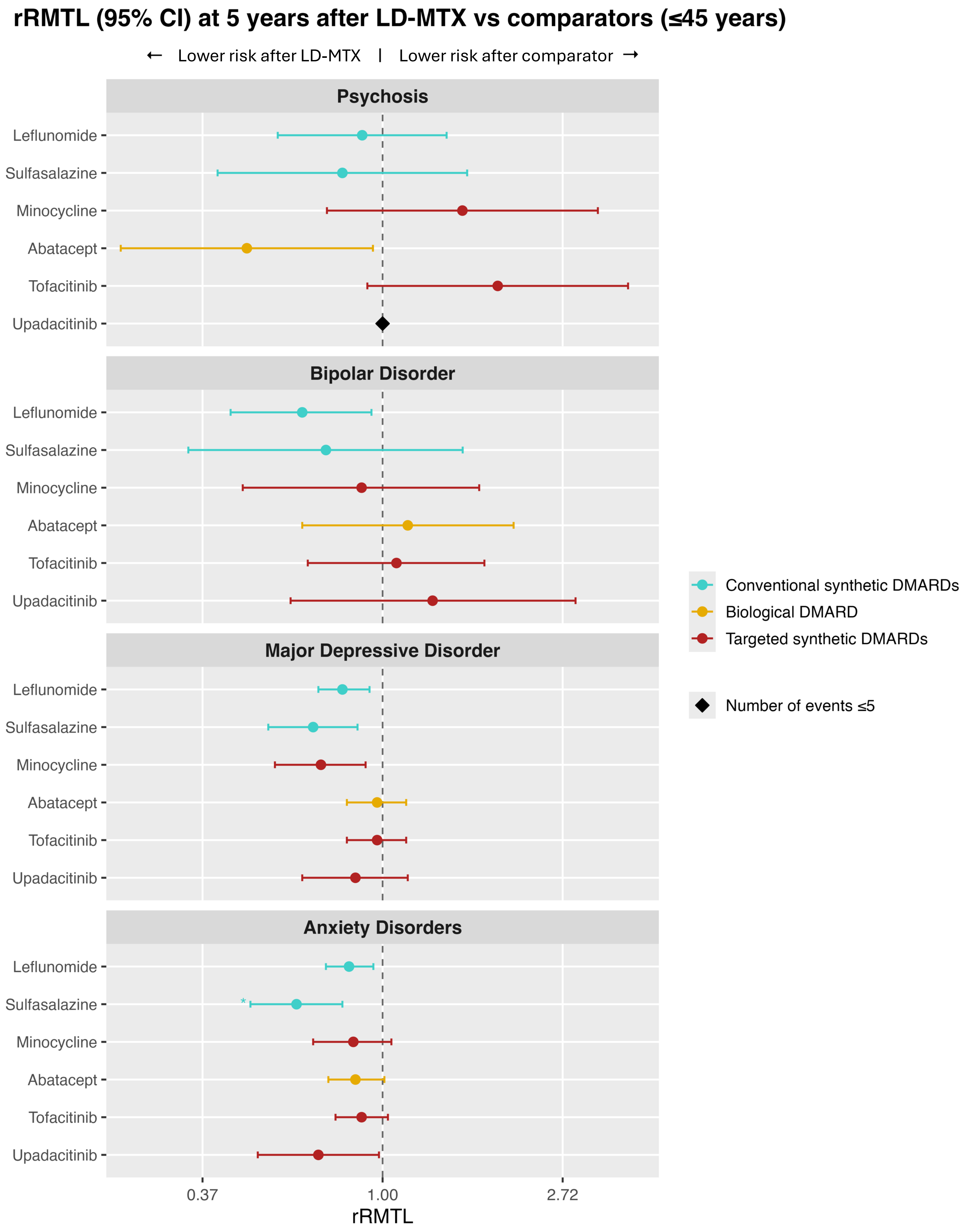


**Supplementary Figure 2A. Restricted mean time lost ratios (rRMTL) at 5 years after initiation of low-dose methotrexate (LD-methotrexate) *vs* comparator drugs.** Values are shown on the log scale with 95% confidence intervals (CIs); axis labels are back-transformed to rRMTL for interpretability. An rRMTL >1 indicates that people were diagnosed with psychosis more frequently or sooner after the comparator drug than after LD-MTX, and conversely for rRMTL <1. The vertical dashed line represents no difference (rRMTL = 1). Colours indicate drug classes; * = P less than Bonferroni corrected critical value for multiple comparison: α = 0.05/44 = 0.0011 for secondary analyses (4 outcomes × 11 drugs). Cohort size after propensity score matching: Leflunomide (N=3,966), Sulfasalazine (N=6,358), Minocycline (N=1,161), Abatacept (N=2,219), Tofacitinib (N=2,269), Upadacitinib (N=1,403). LD-MTX, low-dose methotrexate; DMARDs, disease modifying antirheumatic drugs.

**Supplementary Figure 2B**

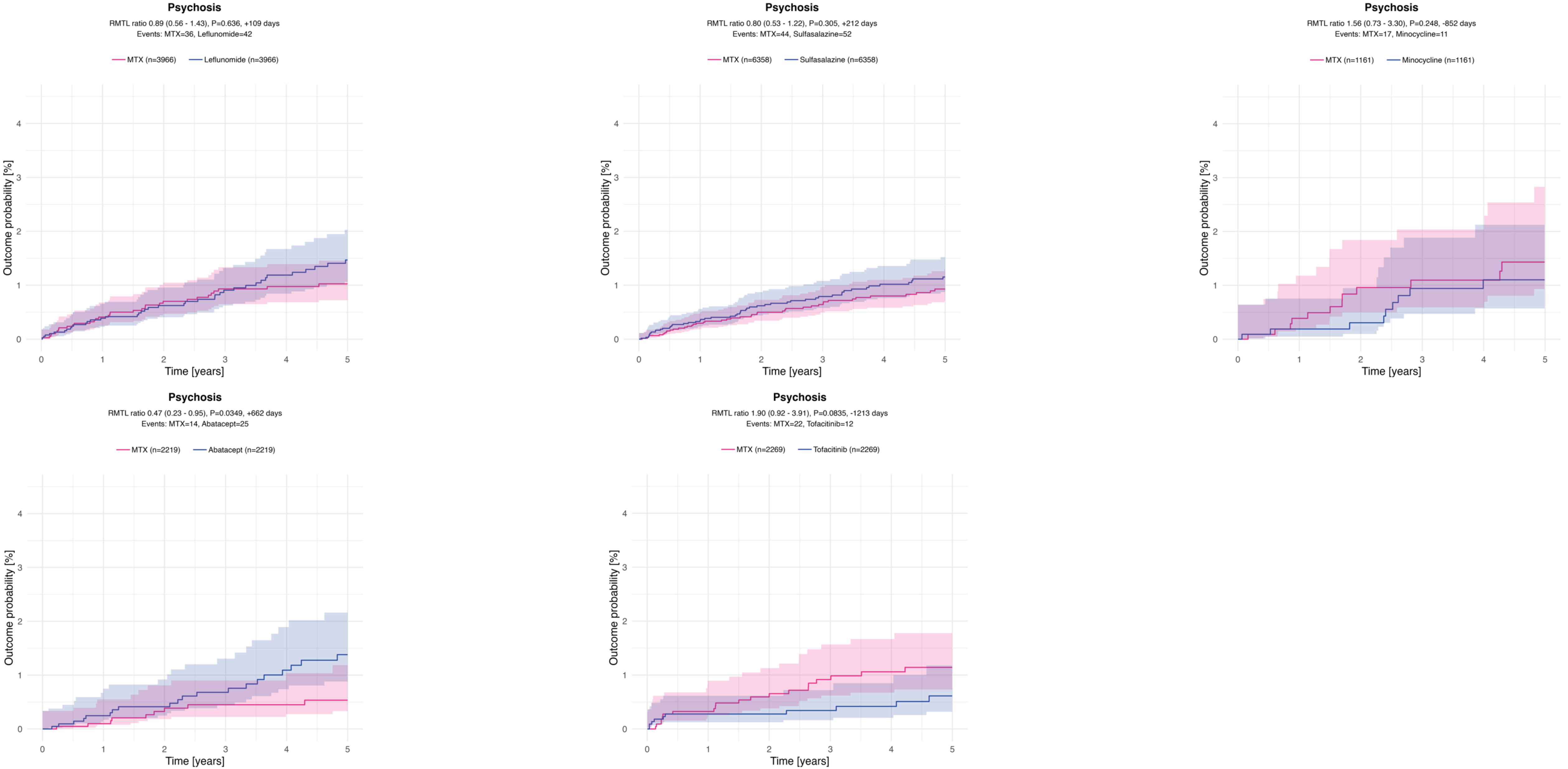


**Supplementary Figure 2B.** Curves representing the Kaplan–Meier estimates of the cumulative incidence of psychosis after initiation of low-dose methotrexate *vs* comparator drugs. Cohort of individuals with rheumatoid arthritis who initiated pharmacological treatment at age ≤45 years. rRMTL <1 indicate that the risk for psychosis is lower after low-dose methotrexate than after the comparator drug, and conversely for rRMTL >1. P value less than Bonferroni corrected critical value for multiple comparison: α = 0.05/44 = 0.0011. Cohort size after propensity score matching: Leflunomide (N=3,966), Sulfasalazine (N=6,358), Minocycline (N=1,161), Abatacept (N=2,219), Tofacitinib (N=2,269) (Upadacitinib panel omitted due to insufficient psychosis events). rRMTL, restricted mean time lost ratio.

**Supplementary Figure 2C**

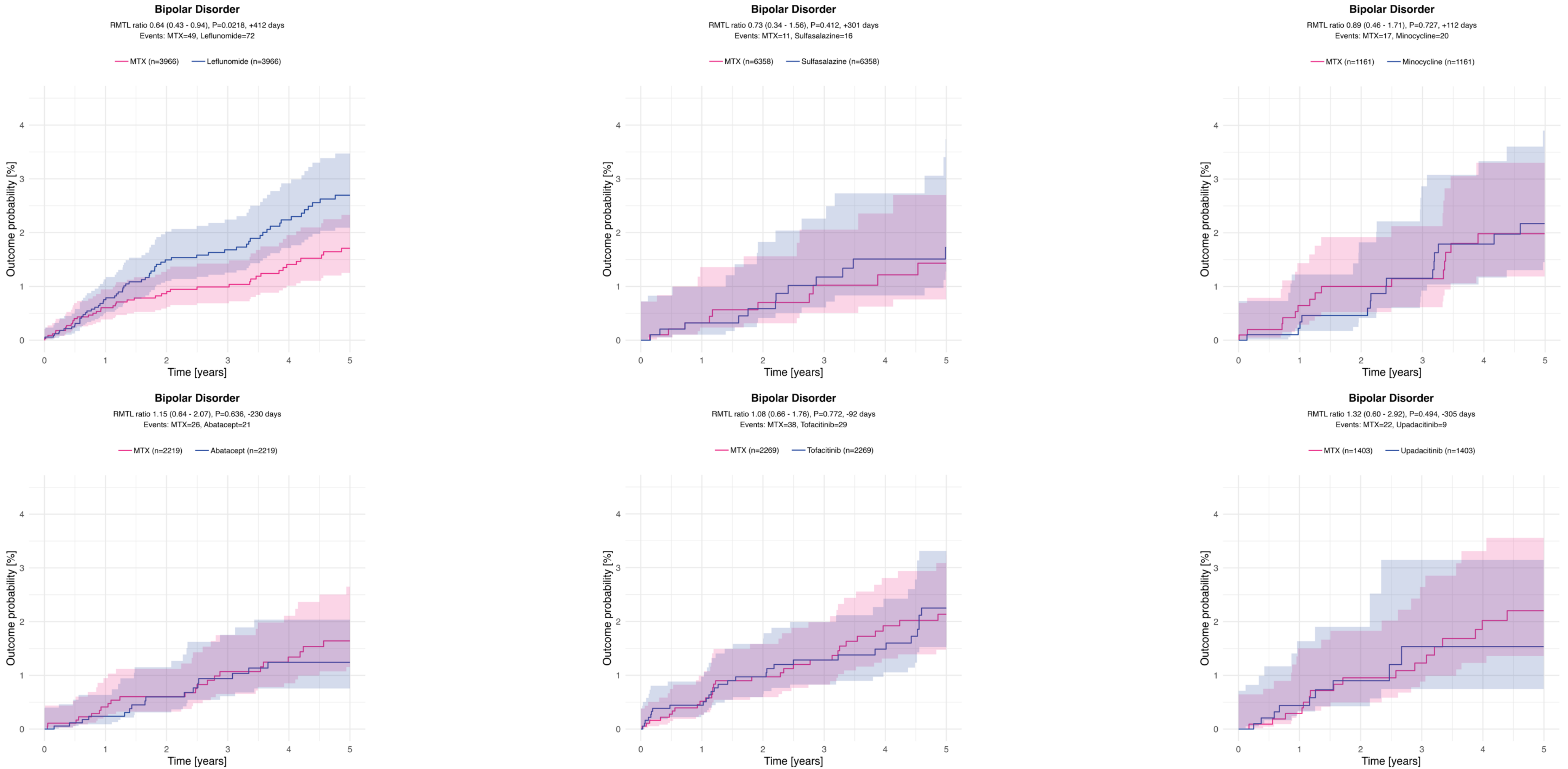


**Supplementary Figure 2C.** Curves representing the Kaplan–Meier estimates of the cumulative incidence of bipolar disorder after initiation of low-dose methotrexate *vs* comparator drugs. Cohort of individuals with rheumatoid arthritis who initiated pharmacological treatment at age ≤45 years. rRMTL <1 indicate that the risk for bipolar disorder is lower after low-dose methotrexate than after the comparator drug, and conversely for rRMTL >1. P value less than Bonferroni corrected critical value for multiple comparison: $\alpha = 0.05/44 = 0.0011$. Cohort size after propensity score matching: Leflunomide (N=3,966), Sulfasalazine (N=6,358), Minocycline (N=1,161), Abatacept (N=2,219), Tofacitinib (N=2,269), Upadacitinib (N=1,403). rRMTL, restricted mean time lost ratio.

**Supplementary Figure 2D**

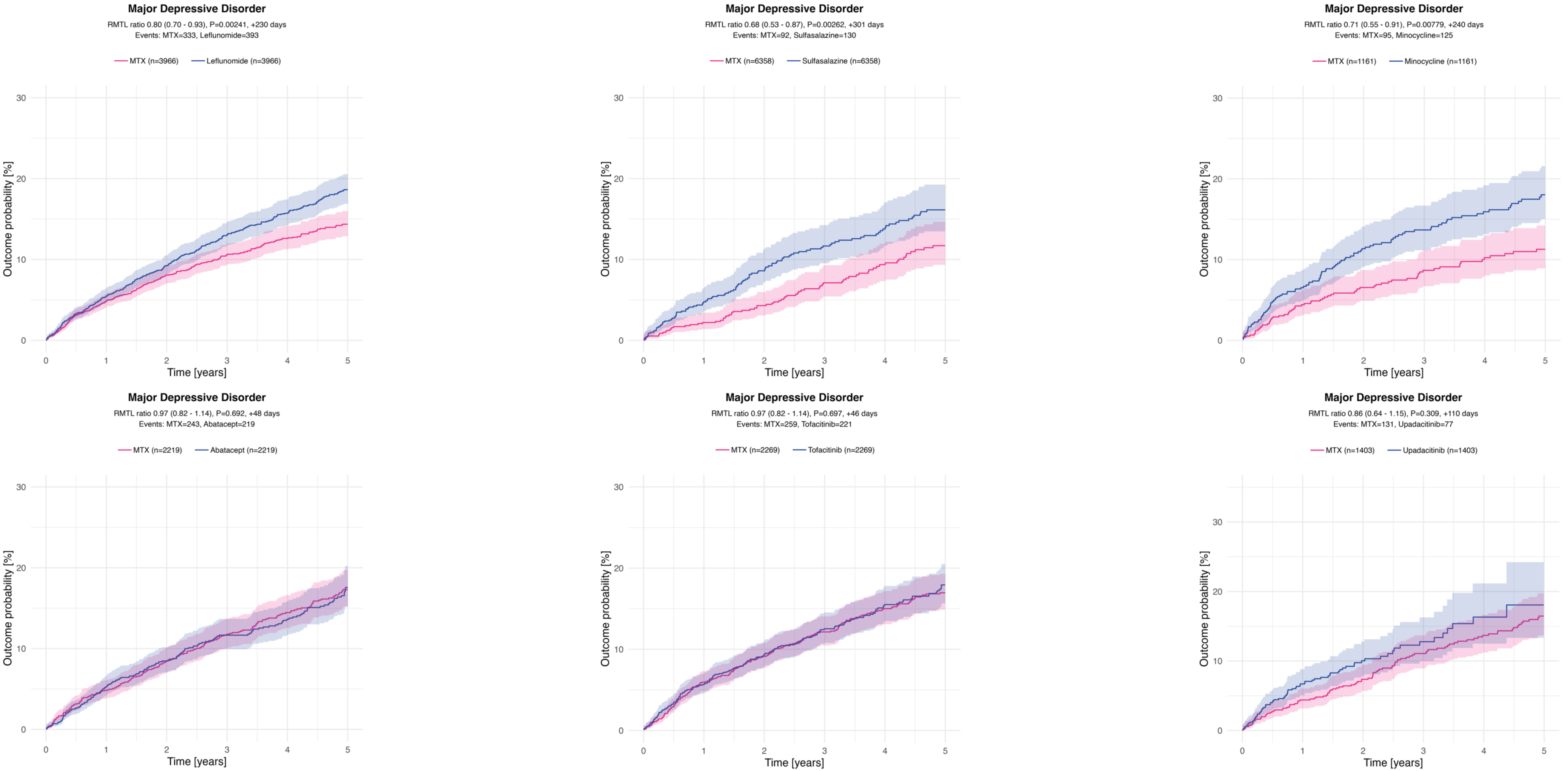


**Supplementary Figure 2D.** Curves representing the Kaplan–Meier estimates of the cumulative incidence of major depressive disorder after initiation of low-dose methotrexate *vs* comparator drugs. Cohort of individuals with rheumatoid arthritis who initiated pharmacological treatment at age ≤45 years. rRMTL <1 indicate that the risk for depression is lower after low-dose methotrexate than after the comparator drug, and conversely for rRMTL >1. P value less than Bonferroni corrected critical value for multiple comparison: α = 0.05/44 = 0.0011. Cohort size after propensity score matching: Leflunomide (N=3,966), Sulfasalazine (N=6,358), Minocycline (N=1,161), Abatacept (N=2,219), Tofacitinib (N=2,269), Upadacitinib (N=1,403). rRMTL, restricted mean time lost ratio.

**Supplementary Figure 2E**

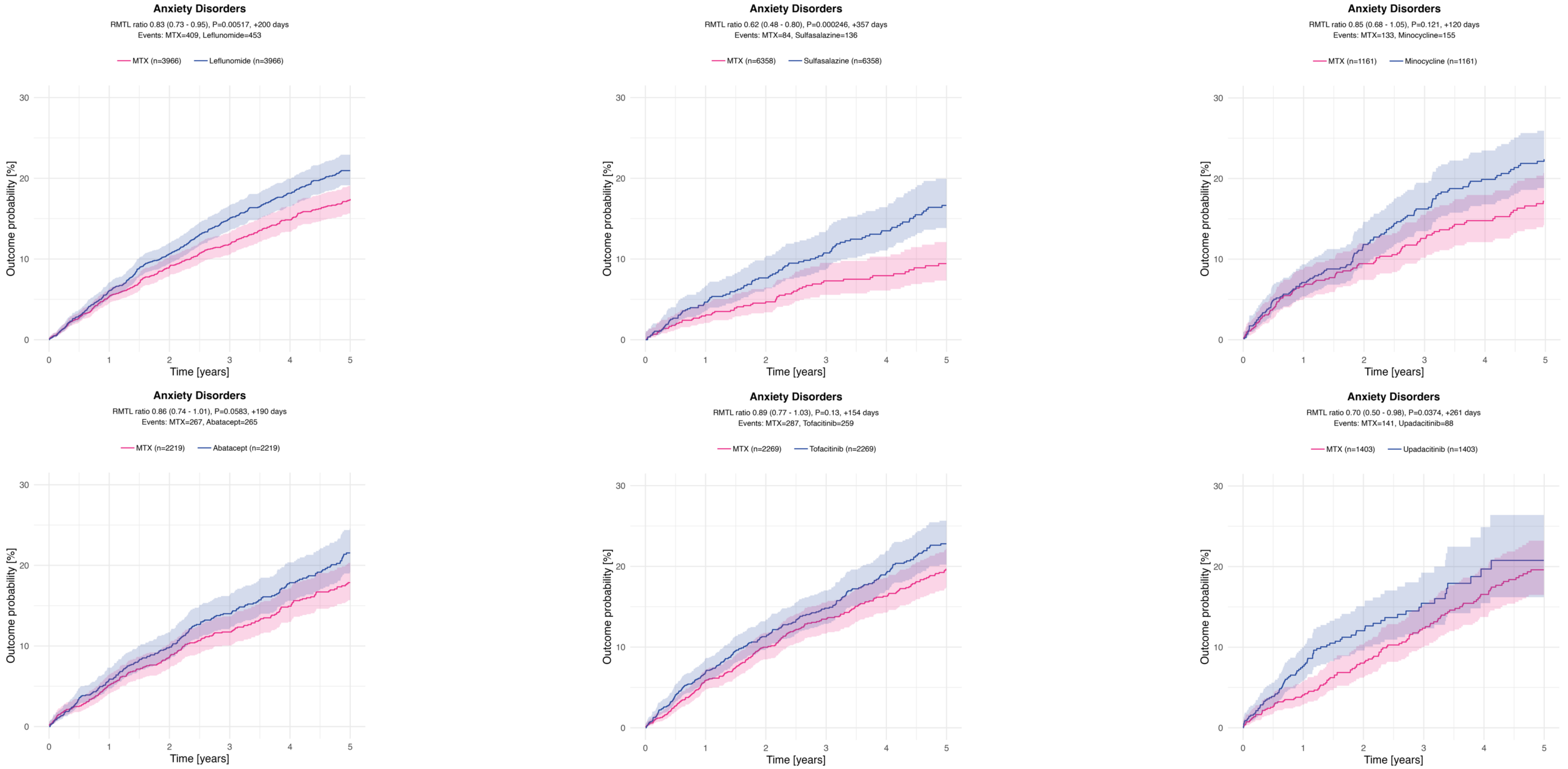


**Supplementary Figure 2E.** Curves representing the Kaplan–Meier estimates of the cumulative incidence of anxiety disorders after initiation of low-dose methotrexate *vs* comparator drugs. Cohort of individuals with rheumatoid arthritis who initiated pharmacological treatment at age ≤45 years. rRMTL <1 indicate that the risk for anxiety disorders is lower after low-dose methotrexate than after the comparator drug, and conversely for rRMTL >1. P value less than Bonferroni corrected critical value for multiple comparison: α = 0.05/44 = 0.0011. Cohort size after propensity score matching: Leflunomide (N=3,966), Sulfasalazine (N=6,358), Minocycline (N=1,161), Abatacept (N=2,219), Tofacitinib (N=2,269), Upadacitinib (N=1,403). rRMTL, restricted mean time lost ratio.

## Supplementary Table S19. Baseline characteristics after propensity score matching low-dose methotrexate *vs* Naproxen (females)

| Cohort 1. Low-dose methotrexate (N = 9,719) and cohort 2. Naproxen (N = 9,719) | | | | | | | |
|---|---|---|---|---|---|---|---|
| **Demographics** | | | | | | | |
| Cohort | | | Mean ± SD | Patients | % of Cohort | P-Value | Std diff. |
| 1<br>2 | AI | Age at Index | 34.2 +/- 8.4<br>34.1 +/- 8.8 | 9,719<br>9,719 | 100%<br>100% | 0.130 | 0.022 |
| 1<br>2 | F | Female | | 9,719<br>9,719 | 100%<br>100% | -- | -- |
| 1<br>2 | 2054-5 | Black or African American | | 1,878<br>1,823 | 19.3%<br>18.8% | 0.315 | 0.014 |
| 1<br>2 | M | Male | | 0<br>0 | 0%<br>0% | -- | -- |
| 1<br>2 | 2106-3 | White | | 6,081<br>6,127 | 62.6%<br>63.0% | 0.495 | 0.010 |
| 1<br>2 | 1002-5 | American Indian or Alaska Native | | 97<br>101 | 1.0%<br>1.0% | 0.775 | 0.004 |
| 1<br>2 | UNK | Unknown Race | | 672<br>683 | 6.9%<br>7.0% | 0.757 | 0.004 |
| 1<br>2 | 2076-8 | Native Hawaiian or Other Pacific Islander | | 67<br>72 | 0.7%<br>0.7% | 0.670 | 0.006 |
| 1<br>2 | UN | Unknown Ethnicity | | 1,464<br>1,472 | 15.1%<br>15.1% | 0.873 | 0.002 |
| 1<br>2 | 2186-5 | Not Hispanic or Latino | | 6,875<br>6,851 | 70.7%<br>70.5% | 0.706 | 0.005 |
| 1<br>2 | 2135-2 | Hispanic or Latino | | 1,380<br>1,396 | 14.2%<br>14.4% | 0.743 | 0.005 |
| 1<br>2 | 2131-1 | Other Race | | 584<br>574 | 6.0%<br>5.9% | 0.762 | 0.004 |
| 1<br>2 | 2028-9 | Asian | | 340<br>339 | 3.5%<br>3.5% | 0.969 | 0.001 |
| **Diagnosis** | | | | | | | |
| Cohort | | | Mean ± SD | Patients | % of Cohort | P-Value | Std diff. |
| 1<br>2 | Z55 | Problems related to education and literacy | | 14<br>19 | 0.1%<br>0.2% | 0.384 | 0.012 |
| 1<br>2 | Z56 | Problems related to employment and unemployment | | 46<br>49 | 0.5%<br>0.5% | 0.758 | 0.004 |
| 1<br>2 | Z81 | Family history of mental and behavioral disorders | | 74<br>81 | 0.8%<br>0.8% | 0.572 | 0.008 |
| 1<br>2 | E70-E88 | Metabolic disorders | | 2,479<br>2,403 | 25.5%<br>24.7% | 0.209 | 0.018 |
| 1<br>2 | F31 | Bipolar disorder | | 493<br>518 | 5.1%<br>5.3% | 0.419 | 0.012 |
| 1<br>2 | F32 | Depressive episode | | 2,426<br>2,455 | 25.0%<br>25.3% | 0.631 | 0.007 |
| 1<br>2 | F33 | Major depressive disorder, recurrent | | 666<br>683 | 6.9%<br>7.0% | 0.631 | 0.007 |
| 1<br>2 | X71 | Intentional self-harm by drowning and submersion | | 0<br>0 | 0%<br>0% | -- | -- |

| | | | | | | | |
|---|---|---|---|---|---|---|---|
| 1<br>2 | R45.851 | Suicidal ideations | | 211<br>241 | 2.2%<br>2.5% | 0.153 | 0.020 |
| 1<br>2 | T14.91 | Suicide attempt | | 14<br>15 | 0.1%<br>0.2% | 0.853 | 0.003 |
| 1<br>2 | X71-X83 | Intentional self-harm | | 23<br>21 | 0.2%<br>0.2% | 0.763 | 0.004 |
| 1<br>2 | W54.0 | Bitten by dog | | 33<br>37 | 0.3%<br>0.4% | 0.632 | 0.007 |
| 1<br>2 | L60.0 | Ingrowing nail | | 102<br>104 | 1.0%<br>1.1% | 0.889 | 0.002 |
| 1<br>2 | B07 | Viral warts | | 195<br>185 | 2.0%<br>1.9% | 0.604 | 0.007 |
| 1<br>2 | I00-I99 | Diseases of the circulatory system | | 3,522<br>3,416 | 36.2%<br>35.1% | 0.113 | 0.023 |
| 1<br>2 | F41 | Other anxiety disorders | | 2,807<br>2,864 | 28.9%<br>29.5% | 0.368 | 0.013 |
| 1<br>2 | F41.1 | Generalized anxiety disorder | | 912<br>950 | 9.4%<br>9.8% | 0.354 | 0.013 |
| 1<br>2 | F40 | Phobic anxiety disorders | | 116<br>130 | 1.2%<br>1.3% | 0.369 | 0.013 |
| 1<br>2 | F41.8 | Other specified anxiety disorders | | 447<br>463 | 4.6%<br>4.8% | 0.587 | 0.008 |
| 1<br>2 | F41.0 | Panic disorder [episodic paroxysmal anxiety] | | 351<br>392 | 3.6%<br>4.0% | 0.125 | 0.022 |
| 1<br>2 | F41.3 | Other mixed anxiety disorders | | 10<br>10 | 0.1%<br>0.1% | 1 | <0.001 |
| 1<br>2 | F41.9 | Anxiety disorder, unspecified | | 2,332<br>2,373 | 24.0%<br>24.4% | 0.492 | 0.010 |
| 1<br>2 | F42 | Obsessive-compulsive disorder | | 97<br>105 | 1.0%<br>1.1% | 0.572 | 0.008 |
| 1<br>2 | F31.2 | Bipolar disorder, current episode manic severe with psychotic features | | 10<br>10 | 0.1%<br>0.1% | 1 | <0.001 |
| 1<br>2 | F31.5 | Bipolar disorder, current episode depressed, severe, with psychotic features | | 10<br>10 | 0.1%<br>0.1% | 1 | <0.001 |
| 1<br>2 | F32.3 | Major depressive disorder, single episode, severe with psychotic features | | 11<br>11 | 0.1%<br>0.1% | 1 | <0.001 |
| 1<br>2 | F33.3 | Major depressive disorder, recurrent, severe with psychotic symptoms | | 16<br>17 | 0.2%<br>0.2% | 0.862 | 0.002 |
| 1<br>2 | F30.2 | Manic episode, severe with psychotic symptoms | | 0<br>0 | 0%<br>0% | -- | -- |
| **Medication** | | | | | | | |
| Cohort | | | Mean ± SD | Patients | % of Cohort | P-Value | Std diff. |
| 1<br>2 | R01AD | Corticosteroids | | 4,563<br>4,401 | 46.9%<br>45.3% | 0.020 | 0.033 |

## Supplementary Table S20. Baseline characteristics after propensity score matching low-dose methotrexate *vs* Diclofenac (females)

**Cohort 1. Low-dose methotrexate (N = 10,596) and cohort 2. Diclofenac (N = 10,596)**

**Demographics**

| Cohort | | | Mean ± SD | Patients | % of Cohort | P-Value | Std diff. |
|---|---|---|---|---|---|---|---|
| 1<br>2 | AI | Age at Index | 35.9 +/- 7.4<br>35.9 +/- 7.3 | 10,596<br>10,596 | 100%<br>100% | 0.989 | <0.001 |
| 1<br>2 | F | Female | | 10,596<br>10,596 | 100%<br>100% | -- | -- |
| 1<br>2 | 2054-5 | Black or African American | | 1,865<br>1,892 | 17.6%<br>17.9% | 0.627 | 0.007 |
| 1<br>2 | M | Male | | 0<br>0 | 0%<br>0% | -- | -- |
| 1<br>2 | 2106-3 | White | | 6,798<br>6,714 | 64.2%<br>63.4% | 0.230 | 0.016 |
| 1<br>2 | 1002-5 | American Indian or Alaska Native | | 104<br>120 | 1.0%<br>1.1% | 0.282 | 0.015 |
| 1<br>2 | UNK | Unknown Race | | 873<br>907 | 8.2%<br>8.6% | 0.400 | 0.012 |
| 1<br>2 | 2076-8 | Native Hawaiian or Other Pacific Islander | | 52<br>49 | 0.5%<br>0.5% | 0.765 | 0.004 |
| 1<br>2 | UN | Unknown Ethnicity | | 1,599<br>1,590 | 15.1%<br>15.0% | 0.863 | 0.002 |
| 1<br>2 | 2186-5 | Not Hispanic or Latino | | 7,600<br>7,536 | 71.7%<br>71.1% | 0.330 | 0.013 |
| 1<br>2 | 2135-2 | Hispanic or Latino | | 1,397<br>1,470 | 13.2%<br>13.9% | 0.143 | 0.020 |
| 1<br>2 | 2131-1 | Other Race | | 621<br>628 | 5.9%<br>5.9% | 0.838 | 0.003 |
| 1<br>2 | 2028-9 | Asian | | 283<br>286 | 2.7%<br>2.7% | 0.899 | 0.002 |

**Diagnosis**

| Cohort | | | Mean ± SD | Patients | % of Cohort | P-Value | Std diff. |
|---|---|---|---|---|---|---|---|
| 1<br>2 | Z55 | Problems related to education and literacy | | 21<br>20 | 0.2%<br>0.2% | 0.876 | 0.002 |
| 1<br>2 | Z56 | Problems related to employment and unemployment | | 50<br>63 | 0.5%<br>0.6% | 0.220 | 0.017 |
| 1<br>2 | Z81 | Family history of mental and behavioral disorders | | 91<br>99 | 0.9%<br>0.9% | 0.560 | 0.008 |
| 1<br>2 | E70-E88 | Metabolic disorders | | 3,007<br>3,003 | 28.4%<br>28.3% | 0.951 | 0.001 |
| 1<br>2 | F31 | Bipolar disorder | | 552<br>570 | 5.2%<br>5.4% | 0.581 | 0.008 |
| 1<br>2 | F32 | Depressive episode | | 3,014<br>3,002 | 28.4%<br>28.3% | 0.855 | 0.003 |
| 1<br>2 | F33 | Major depressive disorder, recurrent | | 858<br>855 | 8.1%<br>8.1% | 0.940 | 0.001 |
| 1<br>2 | X71 | Intentional self-harm by drowning and submersion | | 0<br>0 | 0%<br>0% | -- | -- |

| | | | | | | | |
|---|---|---|---|---|---|---|---|
| 1<br>2 | R45.851 | Suicidal ideations | | 223<br>226 | 2.1%<br>2.1% | 0.886 | 0.002 |
| 1<br>2 | T14.91 | Suicide attempt | | 18<br>17 | 0.2%<br>0.2% | 0.866 | 0.002 |
| 1<br>2 | X71-X83 | Intentional self-harm | | 23<br>27 | 0.2%<br>0.3% | 0.571 | 0.008 |
| 1<br>2 | W54.0 | Bitten by dog | | 41<br>51 | 0.4%<br>0.5% | 0.296 | 0.014 |
| 1<br>2 | L60.0 | Ingrowing nail | | 117<br>153 | 1.1%<br>1.4% | 0.027 | 0.030 |
| 1<br>2 | B07 | Viral warts | | 215<br>225 | 2.0%<br>2.1% | 0.630 | 0.007 |
| 1<br>2 | I00-I99 | Diseases of the circulatory system | | 4,310<br>4,340 | 40.7%<br>41.0% | 0.675 | 0.006 |
| 1<br>2 | F41 | Other anxiety disorders | | 3,602<br>3,576 | 34.0%<br>33.7% | 0.706 | 0.005 |
| 1<br>2 | F41.1 | Generalized anxiety disorder | | 1,188<br>1,181 | 11.2%<br>11.1% | 0.879 | 0.002 |
| 1<br>2 | F40 | Phobic anxiety disorders | | 163<br>159 | 1.5%<br>1.5% | 0.822 | 0.003 |
| 1<br>2 | F41.8 | Other specified anxiety disorders | | 594<br>605 | 5.6%<br>5.7% | 0.744 | 0.004 |
| 1<br>2 | F41.0 | Panic disorder [episodic paroxysmal anxiety] | | 441<br>463 | 4.2%<br>4.4% | 0.455 | 0.010 |
| 1<br>2 | F41.3 | Other mixed anxiety disorders | | 13<br>15 | 0.1%<br>0.1% | 0.705 | 0.005 |
| 1<br>2 | F41.9 | Anxiety disorder, unspecified | | 2,904<br>2,919 | 27.4%<br>27.5% | 0.817 | 0.003 |
| 1<br>2 | F42 | Obsessive-compulsive disorder | | 121<br>116 | 1.1%<br>1.1% | 0.744 | 0.004 |
| 1<br>2 | F31.2 | Bipolar disorder, current episode manic severe with psychotic features | | 10<br>10 | 0.1%<br>0.1% | 1 | <0.001 |
| 1<br>2 | F31.5 | Bipolar disorder, current episode depressed, severe, with psychotic features | | 10<br>10 | 0.1%<br>0.1% | 1 | <0.001 |
| 1<br>2 | F32.3 | Major depressive disorder, single episode, severe with psychotic features | | 11<br>11 | 0.1%<br>0.1% | 1 | <0.001 |
| 1<br>2 | F33.3 | Major depressive disorder, recurrent, severe with psychotic symptoms | | 16<br>18 | 0.2%<br>0.2% | 0.731 | 0.005 |
| 1<br>2 | F30.2 | Manic episode, severe with psychotic symptoms | | 0<br>0 | 0%<br>0% | -- | -- |
| **Medication** | | | | | | | |
| Cohort | | | Mean ± SD | Patients | % of Cohort | P-Value | Std diff. |
| 1<br>2 | R01AD | Corticosteroids | | 5,455<br>5,413 | 51.5%<br>51.1% | 0.564 | 0.008 |

## Supplementary Table S21. Baseline characteristics after propensity score matching low-dose methotrexate *vs* Celecoxib (females)

| Cohort 1. Low-dose methotrexate (N = 7,013) and cohort 2. Celecoxib (N = 7,013) | | | | | | | |
|---|---|---|---|---|---|---|---|
| **Demographics** | | | | | | | |
| Cohort | | | Mean ± SD | Patients | % of Cohort | P-Value | Std diff. |
| 1<br>2 | AI | Age at Index | 36.1 +/- 7.4<br>36.1 +/- 7.3 | 7,013<br>7,013 | 100%<br>100% | 0.867 | 0.003 |
| 1<br>2 | F | Female | | 7,013<br>7,013 | 100%<br>100% | -- | -- |
| 1<br>2 | 2054-5 | Black or African American | | 912<br>917 | 13.0%<br>13.1% | 0.900 | 0.002 |
| 1<br>2 | M | Male | | 0<br>0 | 0%<br>0% | -- | -- |
| 1<br>2 | 2106-3 | White | | 5,080<br>4,969 | 72.4%<br>70.9% | 0.038 | 0.035 |
| 1<br>2 | 1002-5 | American Indian or Alaska Native | | 67<br>72 | 1.0%<br>1.0% | 0.670 | 0.007 |
| 1<br>2 | UNK | Unknown Race | | 417<br>447 | 5.9%<br>6.4% | 0.292 | 0.018 |
| 1<br>2 | 2076-8 | Native Hawaiian or Other Pacific Islander | | 36<br>37 | 0.5%<br>0.5% | 0.907 | 0.002 |
| 1<br>2 | UN | Unknown Ethnicity | | 1,073<br>1,096 | 15.3%<br>15.6% | 0.591 | 0.009 |
| 1<br>2 | 2186-5 | Not Hispanic or Latino | | 5,145<br>5,049 | 73.4%<br>72.0% | 0.069 | 0.031 |
| 1<br>2 | 2135-2 | Hispanic or Latino | | 795<br>868 | 11.3%<br>12.4% | 0.057 | 0.032 |
| 1<br>2 | 2131-1 | Other Race | | 293<br>358 | 4.2%<br>5.1% | 0.009 | 0.044 |
| 1<br>2 | 2028-9 | Asian | | 208<br>213 | 3.0%<br>3.0% | 0.805 | 0.004 |
| **Diagnosis** | | | | | | | |
| Cohort | | | Mean ± SD | Patients | % of Cohort | P-Value | Std diff. |
| 1<br>2 | Z55 | Problems related to education and literacy | | 18<br>16 | 0.3%<br>0.2% | 0.731 | 0.006 |
| 1<br>2 | Z56 | Problems related to employment and unemployment | | 31<br>39 | 0.4%<br>0.6% | 0.338 | 0.016 |
| 1<br>2 | Z81 | Family history of mental and behavioral disorders | | 74<br>83 | 1.1%<br>1.2% | 0.470 | 0.012 |
| 1<br>2 | E70-E88 | Metabolic disorders | | 2,030<br>2,060 | 28.9%<br>29.4% | 0.577 | 0.009 |
| 1<br>2 | F31 | Bipolar disorder | | 379<br>412 | 5.4%<br>5.9% | 0.227 | 0.020 |
| 1<br>2 | F32 | Depressive episode | | 2,210<br>2,190 | 31.5%<br>31.2% | 0.716 | 0.006 |
| 1<br>2 | F33 | Major depressive disorder, recurrent | | 653<br>676 | 9.3%<br>9.6% | 0.507 | 0.011 |
| 1<br>2 | X71 | Intentional self-harm by drowning and submersion | | 0<br>10 | 0%<br>0.1% | 0.002 | 0.053 |

| | | | | | | | |
|---|---|---|---|---|---|---|---|
| 1<br>2 | R45.851 | Suicidal ideations | | 145<br>152 | 2.1%<br>2.2% | 0.681 | 0.007 |
| 1<br>2 | T14.91 | Suicide attempt | | 12<br>12 | 0.2%<br>0.2% | 1 | <0.001 |
| 1<br>2 | X71-X83 | Intentional self-harm | | 21<br>19 | 0.3%<br>0.3% | 0.751 | 0.005 |
| 1<br>2 | W54.0 | Bitten by dog | | 30<br>32 | 0.4%<br>0.5% | 0.799 | 0.004 |
| 1<br>2 | L60.0 | Ingrowing nail | | 93<br>102 | 1.3%<br>1.5% | 0.516 | 0.011 |
| 1<br>2 | B07 | Viral warts | | 167<br>145 | 2.4%<br>2.1% | 0.208 | 0.021 |
| 1<br>2 | I00-I99 | Diseases of the circulatory system | | 2,911<br>2,895 | 41.5%<br>41.3% | 0.784 | 0.005 |
| 1<br>2 | F41 | Other anxiety disorders | | 2,690<br>2,806 | 38.4%<br>40.0% | 0.045 | 0.034 |
| 1<br>2 | F41.1 | Generalized anxiety disorder | | 877<br>946 | 12.5%<br>13.5% | 0.083 | 0.029 |
| 1<br>2 | F40 | Phobic anxiety disorders | | 114<br>119 | 1.6%<br>1.7% | 0.741 | 0.006 |
| 1<br>2 | F41.8 | Other specified anxiety disorders | | 480<br>527 | 6.8%<br>7.5% | 0.124 | 0.026 |
| 1<br>2 | F41.0 | Panic disorder [episodic paroxysmal anxiety] | | 333<br>357 | 4.7%<br>5.1% | 0.349 | 0.016 |
| 1<br>2 | F41.3 | Other mixed anxiety disorders | | 10<br>12 | 0.1%<br>0.2% | 0.670 | 0.007 |
| 1<br>2 | F41.9 | Anxiety disorder, unspecified | | 2,293<br>2,364 | 32.7%<br>33.7% | 0.203 | 0.021 |
| 1<br>2 | F42 | Obsessive-compulsive disorder | | 84<br>83 | 1.2%<br>1.2% | 0.938 | 0.001 |
| 1<br>2 | F31.2 | Bipolar disorder, current episode manic severe with psychotic features | | 10<br>10 | 0.1%<br>0.1% | 1 | <0.001 |
| 1<br>2 | F31.5 | Bipolar disorder, current episode depressed, severe, with psychotic features | | 10<br>10 | 0.1%<br>0.1% | 1 | <0.001 |
| 1<br>2 | F32.3 | Major depressive disorder, single episode, severe with psychotic features | | 10<br>10 | 0.1%<br>0.1% | 1 | <0.001 |
| 1<br>2 | F33.3 | Major depressive disorder, recurrent, severe with psychotic symptoms | | 14<br>12 | 0.2%<br>0.2% | 0.695 | 0.007 |
| 1<br>2 | F30.2 | Manic episode, severe with psychotic symptoms | | 0<br>0 | 0%<br>0% | -- | -- |
| **Medication** | | | | | | | |
| Cohort | | | Mean ± SD | Patients | % of Cohort | P-Value | Std diff. |
| 1<br>2 | R01AD | Corticosteroids | | 4,267<br>4,214 | 60.8%<br>60.1% | 0.360 | 0.015 |

## Supplementary Table S22. Baseline characteristics after propensity score matching low-dose methotrexate *vs* Infliximab (females)

**Cohort 1. Low-dose methotrexate (N = 829) and cohort 2. Infliximab (N = 829)**

**Demographics**

| Cohort | | | Mean ± SD | Patients | % of Cohort | P-Value | Std diff. |
|---|---|---|---|---|---|---|---|
| 1<br>2 | AI | Age at Index | 33.9 +/- 8.4<br>34.0 +/- 8.3 | 829<br>829 | 100%<br>100% | 0.787 | 0.013 |
| 1<br>2 | F | Female | | 829<br>829 | 100%<br>100% | -- | -- |
| 1<br>2 | 2054-5 | Black or African American | | 123<br>124 | 14.8%<br>15.0% | 0.945 | 0.003 |
| 1<br>2 | M | Male | | 0<br>0 | 0%<br>0% | -- | -- |
| 1<br>2 | 2106-3 | White | | 592<br>581 | 71.4%<br>70.1% | 0.553 | 0.029 |
| 1<br>2 | 1002-5 | American Indian or Alaska Native | | 10<br>10 | 1.2%<br>1.2% | 1 | <0.001 |
| 1<br>2 | UNK | Unknown Race | | 38<br>47 | 4.6%<br>5.7% | 0.316 | 0.049 |
| 1<br>2 | 2076-8 | Native Hawaiian or Other Pacific Islander | | 10<br>10 | 1.2%<br>1.2% | 1 | <0.001 |
| 1<br>2 | UN | Unknown Ethnicity | | 73<br>70 | 8.8%<br>8.4% | 0.793 | 0.013 |
| 1<br>2 | 2186-5 | Not Hispanic or Latino | | 656<br>648 | 79.1%<br>78.2% | 0.632 | 0.024 |
| 1<br>2 | 2135-2 | Hispanic or Latino | | 100<br>111 | 12.1%<br>13.4% | 0.418 | 0.040 |
| 1<br>2 | 2131-1 | Other Race | | 49<br>48 | 5.9%<br>5.8% | 0.917 | 0.005 |
| 1<br>2 | 2028-9 | Asian | | 13<br>16 | 1.6%<br>1.9% | 0.574 | 0.028 |

**Diagnosis**

| Cohort | | | Mean ± SD | Patients | % of Cohort | P-Value | Std diff. |
|---|---|---|---|---|---|---|---|
| 1<br>2 | Z55 | Problems related to education and literacy | | 10<br>10 | 1.2%<br>1.2% | 1 | <0.001 |
| 1<br>2 | Z56 | Problems related to employment and unemployment | | 10<br>10 | 1.2%<br>1.2% | 1 | <0.001 |
| 1<br>2 | Z81 | Family history of mental and behavioral disorders | | 10<br>10 | 1.2%<br>1.2% | 1 | <0.001 |
| 1<br>2 | E70-E88 | Metabolic disorders | | 200<br>203 | 24.1%<br>24.5% | 0.864 | 0.008 |
| 1<br>2 | F31 | Bipolar disorder | | 21<br>28 | 2.5%<br>3.4% | 0.310 | 0.050 |
| 1<br>2 | F32 | Depressive episode | | 206<br>216 | 24.8%<br>26.1% | 0.573 | 0.028 |
| 1<br>2 | F33 | Major depressive disorder, recurrent | | 59<br>59 | 7.1%<br>7.1% | 1 | <0.001 |
| 1<br>2 | X71 | Intentional self-harm by drowning and submersion | | 0<br>0 | 0%<br>0% | -- | -- |

| | | | | | | |
|---|---|---|---|---|---|---|
| 1<br>2 | R45.851 | Suicidal ideations | 11<br>18 | 1.3%<br>2.2% | 0.190 | 0.064 |
| 1<br>2 | T14.91 | Suicide attempt | 0<br>0 | 0%<br>0% | -- | -- |
| 1<br>2 | X71-X83 | Intentional self-harm | 10<br>10 | 1.2%<br>1.2% | 1 | <0.001 |
| 1<br>2 | W54.0 | Bitten by dog | 10<br>10 | 1.2%<br>1.2% | 1 | <0.001 |
| 1<br>2 | L60.0 | Ingrowing nail | 11<br>10 | 1.3%<br>1.2% | 0.826 | 0.011 |
| 1<br>2 | B07 | Viral warts | 14<br>16 | 1.7%<br>1.9% | 0.713 | 0.018 |
| 1<br>2 | I00-I99 | Diseases of the circulatory system | 277<br>269 | 33.4%<br>32.4% | 0.676 | 0.021 |
| 1<br>2 | F41 | Other anxiety disorders | 232<br>243 | 28.0%<br>29.3% | 0.550 | 0.029 |
| 1<br>2 | F41.1 | Generalized anxiety disorder | 63<br>73 | 7.6%<br>8.8% | 0.371 | 0.044 |
| 1<br>2 | F40 | Phobic anxiety disorders | 10<br>11 | 1.2%<br>1.3% | 0.826 | 0.011 |
| 1<br>2 | F41.8 | Other specified anxiety disorders | 41<br>43 | 4.9%<br>5.2% | 0.823 | 0.011 |
| 1<br>2 | F41.0 | Panic disorder [episodic paroxysmal anxiety] | 24<br>30 | 2.9%<br>3.6% | 0.406 | 0.041 |
| 1<br>2 | F41.3 | Other mixed anxiety disorders | 0<br>0 | 0%<br>0% | -- | -- |
| 1<br>2 | F41.9 | Anxiety disorder, unspecified | 211<br>213 | 25.5%<br>25.7% | 0.910 | 0.006 |
| 1<br>2 | F42 | Obsessive-compulsive disorder | 10<br>11 | 1.2%<br>1.3% | 0.826 | 0.011 |
| 1<br>2 | F31.2 | Bipolar disorder, current episode manic severe with psychotic features | 10<br>10 | 1.2%<br>1.2% | 1 | <0.001 |
| 1<br>2 | F31.5 | Bipolar disorder, current episode depressed, severe, with psychotic features | 0<br>0 | 0%<br>0% | -- | -- |
| 1<br>2 | F32.3 | Major depressive disorder, single episode, severe with psychotic features | 0<br>0 | 0%<br>0% | -- | -- |
| 1<br>2 | F33.3 | Major depressive disorder, recurrent, severe with psychotic symptoms | 10<br>10 | 1.2%<br>1.2% | 1 | <0.001 |
| 1<br>2 | F30.2 | Manic episode, severe with psychotic symptoms | 0<br>0 | 0%<br>0% | -- | -- |
| **Medication** | | | | | | |
| Cohort | | Mean ± SD | Patients | % of Cohort | P-Value | Std diff. |
| 1<br>2 | R01AD | Corticosteroids | 374<br>377 | 45.1%<br>45.5% | 0.882 | 0.007 |

## Supplementary Table S23. Baseline characteristics after propensity score matching low-dose methotrexate *vs* Adalimumab (females)

| Cohort 1. Low-dose methotrexate (N = 5,230) and cohort 2. Adalimumab (N = 5,230) | | | | | | | |
|---|---|---|---|---|---|---|---|
| **Demographics** | | | | | | | |
| Cohort | | | Mean ± SD | Patients | % of Cohort | P-Value | Std diff. |
| 1<br>2 | AI | Age at Index | 34.2 +/- 8.2<br>34.3 +/- 7.9 | 5,230<br>5,230 | 100%<br>100% | 0.596 | 0.010 |
| 1<br>2 | F | Female | | 5,230<br>5,230 | 100%<br>100% | -- | -- |
| 1<br>2 | 2054-5 | Black or African American | | 548<br>566 | 10.5%<br>10.8% | 0.568 | 0.011 |
| 1<br>2 | M | Male | | 0<br>0 | 0%<br>0% | -- | -- |
| 1<br>2 | 2106-3 | White | | 3,780<br>3,742 | 72.3%<br>71.5% | 0.408 | 0.016 |
| 1<br>2 | 1002-5 | American Indian or Alaska Native | | 80<br>77 | 1.5%<br>1.5% | 0.809 | 0.005 |
| 1<br>2 | UNK | Unknown Race | | 379<br>400 | 7.2%<br>7.6% | 0.434 | 0.015 |
| 1<br>2 | 2076-8 | Native Hawaiian or Other Pacific Islander | | 17<br>18 | 0.3%<br>0.3% | 0.866 | 0.003 |
| 1<br>2 | UN | Unknown Ethnicity | | 734<br>741 | 14.0%<br>14.2% | 0.844 | 0.004 |
| 1<br>2 | 2186-5 | Not Hispanic or Latino | | 3,804<br>3,783 | 72.7%<br>72.3% | 0.645 | 0.009 |
| 1<br>2 | 2135-2 | Hispanic or Latino | | 692<br>706 | 13.2%<br>13.5% | 0.687 | 0.008 |
| 1<br>2 | 2131-1 | Other Race | | 293<br>284 | 5.6%<br>5.4% | 0.700 | 0.008 |
| 1<br>2 | 2028-9 | Asian | | 133<br>143 | 2.5%<br>2.7% | 0.542 | 0.012 |
| **Diagnosis** | | | | | | | |
| Cohort | | | Mean ± SD | Patients | % of Cohort | P-Value | Std diff. |
| 1<br>2 | Z55 | Problems related to education and literacy | | 10<br>10 | 0.2%<br>0.2% | 1 | <0.001 |
| 1<br>2 | Z56 | Problems related to employment and unemployment | | 16<br>20 | 0.3%<br>0.4% | 0.504 | 0.013 |
| 1<br>2 | Z81 | Family history of mental and behavioral disorders | | 16<br>26 | 0.3%<br>0.5% | 0.122 | 0.030 |
| 1<br>2 | E70-E88 | Metabolic disorders | | 927<br>1,005 | 17.7%<br>19.2% | 0.049 | 0.038 |
| 1<br>2 | F31 | Bipolar disorder | | 143<br>162 | 2.7%<br>3.1% | 0.270 | 0.022 |
| 1<br>2 | F32 | Depressive episode | | 944<br>1,013 | 18.0%<br>19.4% | 0.084 | 0.034 |
| 1<br>2 | F33 | Major depressive disorder, recurrent | | 251<br>305 | 4.8%<br>5.8% | 0.019 | 0.046 |
| 1<br>2 | X71 | Intentional self-harm by drowning and submersion | | 0<br>0 | 0%<br>0% | -- | -- |

| Cohort | | | Mean ± SD | Patients | % of Cohort | P-Value | Std diff. |
|---|---|---|---|---|---|---|---|
| 1<br>2 | R45.851 | Suicidal ideations | | 69<br>83 | 1.3%<br>1.6% | 0.253 | 0.022 |
| 1<br>2 | T14.91 | Suicide attempt | | 10<br>10 | 0.2%<br>0.2% | 1 | <0.001 |
| 1<br>2 | X71-X83 | Intentional self-harm | | 10<br>10 | 0.2%<br>0.2% | 1 | <0.001 |
| 1<br>2 | W54.0 | Bitten by dog | | 18<br>13 | 0.3%<br>0.2% | 0.368 | 0.018 |
| 1<br>2 | L60.0 | Ingrowing nail | | 40<br>59 | 0.8%<br>1.1% | 0.055 | 0.038 |
| 1<br>2 | B07 | Viral warts | | 79<br>103 | 1.5%<br>2.0% | 0.073 | 0.035 |
| 1<br>2 | I00-I99 | Diseases of the circulatory system | | 1,436<br>1,468 | 27.5%<br>28.1% | 0.485 | 0.014 |
| 1<br>2 | F41 | Other anxiety disorders | | 1,197<br>1,289 | 22.9%<br>24.6% | 0.035 | 0.041 |
| 1<br>2 | F41.1 | Generalized anxiety disorder | | 353<br>428 | 6.7%<br>8.2% | 0.005 | 0.055 |
| 1<br>2 | F40 | Phobic anxiety disorders | | 48<br>59 | 0.9%<br>1.1% | 0.285 | 0.021 |
| 1<br>2 | F41.8 | Other specified anxiety disorders | | 203<br>233 | 3.9%<br>4.5% | 0.142 | 0.029 |
| 1<br>2 | F41.0 | Panic disorder [episodic paroxysmal anxiety] | | 121<br>154 | 2.3%<br>2.9% | 0.044 | 0.039 |
| 1<br>2 | F41.3 | Other mixed anxiety disorders | | 10<br>10 | 0.2%<br>0.2% | 1 | <0.001 |
| 1<br>2 | F41.9 | Anxiety disorder, unspecified | | 1,001<br>1,058 | 19.1%<br>20.2% | 0.161 | 0.027 |
| 1<br>2 | F42 | Obsessive-compulsive disorder | | 41<br>52 | 0.8%<br>1.0% | 0.252 | 0.022 |
| 1<br>2 | F31.2 | Bipolar disorder, current episode manic severe with psychotic features | | 10<br>10 | 0.2%<br>0.2% | 1 | <0.001 |
| 1<br>2 | F31.5 | Bipolar disorder, current episode depressed, severe, with psychotic features | | 0<br>10 | 0%<br>0.2% | 0.002 | 0.062 |
| 1<br>2 | F32.3 | Major depressive disorder, single episode, severe with psychotic features | | 10<br>10 | 0.2%<br>0.2% | 1 | <0.001 |
| 1<br>2 | F33.3 | Major depressive disorder, recurrent, severe with psychotic symptoms | | 10<br>10 | 0.2%<br>0.2% | 1 | <0.001 |
| 1<br>2 | F30.2 | Manic episode, severe with psychotic symptoms | | 0<br>0 | 0%<br>0% | -- | -- |
| **Medication** | | | | | | | |
| Cohort | | | Mean ± SD | Patients | % of Cohort | P-Value | Std diff. |
| 1<br>2 | R01AD | Corticosteroids | | 2,050<br>2,065 | 39.2%<br>39.5% | 0.764 | 0.006 |

## Supplementary Table S24. Baseline characteristics after propensity score matching low-dose methotrexate *vs* Etanercept (females)

| Cohort 1. Low-dose methotrexate (N = 4,931) and cohort 2. Etanercept (N = 4,931) | | | | | | | |
|---|---|---|---|---|---|---|---|
| **Demographics** | | | | | | | |
| Cohort | | | Mean ± SD | Patients | % of Cohort | P-Value | Std diff. |
| 1<br>2 | AI | Age at Index | 34.7 +/- 7.9<br>34.6 +/- 7.9 | 4,931<br>4,931 | 100%<br>100% | 0.877 | 0.003 |
| 1<br>2 | F | Female | | 4,931<br>4,931 | 100%<br>100% | -- | -- |
| 1<br>2 | 2054-5 | Black or African American | | 420<br>428 | 8.5%<br>8.7% | 0.774 | 0.006 |
| 1<br>2 | M | Male | | 0<br>0 | 0%<br>0% | -- | -- |
| 1<br>2 | 2106-3 | White | | 3,569<br>3,538 | 72.4%<br>71.8% | 0.487 | 0.014 |
| 1<br>2 | 1002-5 | American Indian or Alaska Native | | 68<br>82 | 1.4%<br>1.7% | 0.249 | 0.023 |
| 1<br>2 | UNK | Unknown Race | | 410<br>411 | 8.3%<br>8.3% | 0.971 | 0.001 |
| 1<br>2 | 2076-8 | Native Hawaiian or Other Pacific Islander | | 28<br>25 | 0.6%<br>0.5% | 0.679 | 0.008 |
| 1<br>2 | UN | Unknown Ethnicity | | 931<br>963 | 18.9%<br>19.5% | 0.413 | 0.016 |
| 1<br>2 | 2186-5 | Not Hispanic or Latino | | 3,342<br>3,322 | 67.8%<br>67.4% | 0.667 | 0.009 |
| 1<br>2 | 2135-2 | Hispanic or Latino | | 658<br>646 | 13.3%<br>13.1% | 0.721 | 0.007 |
| 1<br>2 | 2131-1 | Other Race | | 269<br>280 | 5.5%<br>5.7% | 0.629 | 0.010 |
| 1<br>2 | 2028-9 | Asian | | 167<br>167 | 3.4%<br>3.4% | 1 | <0.001 |
| **Diagnosis** | | | | | | | |
| Cohort | | | Mean ± SD | Patients | % of Cohort | P-Value | Std diff. |
| 1<br>2 | Z55 | Problems related to education and literacy | | 10<br>10 | 0.2%<br>0.2% | 1 | <0.001 |
| 1<br>2 | Z56 | Problems related to employment and unemployment | | 19<br>17 | 0.4%<br>0.3% | 0.738 | 0.007 |
| 1<br>2 | Z81 | Family history of mental and behavioral disorders | | 15<br>19 | 0.3%<br>0.4% | 0.492 | 0.014 |
| 1<br>2 | E70-E88 | Metabolic disorders | | 718<br>744 | 14.6%<br>15.1% | 0.461 | 0.015 |
| 1<br>2 | F31 | Bipolar disorder | | 103<br>123 | 2.1%<br>2.5% | 0.178 | 0.027 |
| 1<br>2 | F32 | Depressive episode | | 694<br>775 | 14.1%<br>15.7% | 0.022 | 0.046 |
| 1<br>2 | F33 | Major depressive disorder, recurrent | | 201<br>255 | 4.1%<br>5.2% | 0.010 | 0.052 |
| 1<br>2 | X71 | Intentional self-harm by drowning and submersion | | 0<br>0 | 0%<br>0% | -- | -- |

| Cohort | Code | Description | Mean ± SD | Patients | % of Cohort | P-Value | Std diff. |
|---|---|---|---|---|---|---|---|
| 1<br>2 | R45.851 | Suicidal ideations | | 60<br>69 | 1.2%<br>1.4% | 0.425 | 0.016 |
| 1<br>2 | T14.91 | Suicide attempt | | 10<br>10 | 0.2%<br>0.2% | 1 | <0.001 |
| 1<br>2 | X71-X83 | Intentional self-harm | | 10<br>10 | 0.2%<br>0.2% | 1 | <0.001 |
| 1<br>2 | W54.0 | Bitten by dog | | 15<br>10 | 0.3%<br>0.2% | 0.317 | 0.020 |
| 1<br>2 | L60.0 | Ingrowing nail | | 35<br>39 | 0.7%<br>0.8% | 0.641 | 0.009 |
| 1<br>2 | B07 | Viral warts | | 79<br>56 | 1.6%<br>1.1% | 0.046 | 0.040 |
| 1<br>2 | I00-I99 | Diseases of the circulatory system | | 1,174<br>1,196 | 23.8%<br>24.3% | 0.604 | 0.010 |
| 1<br>2 | F41 | Other anxiety disorders | | 933<br>1,056 | 18.9%<br>21.4% | 0.002 | 0.062 |
| 1<br>2 | F41.1 | Generalized anxiety disorder | | 270<br>328 | 5.5%<br>6.7% | 0.014 | 0.049 |
| 1<br>2 | F40 | Phobic anxiety disorders | | 32<br>48 | 0.6%<br>1.0% | 0.072 | 0.036 |
| 1<br>2 | F41.8 | Other specified anxiety disorders | | 134<br>179 | 2.7%<br>3.6% | 0.010 | 0.052 |
| 1<br>2 | F41.0 | Panic disorder [episodic paroxysmal anxiety] | | 90<br>117 | 1.8%<br>2.4% | 0.058 | 0.038 |
| 1<br>2 | F41.3 | Other mixed anxiety disorders | | 10<br>10 | 0.2%<br>0.2% | 1 | <0.001 |
| 1<br>2 | F41.9 | Anxiety disorder, unspecified | | 787<br>853 | 16.0%<br>17.3% | 0.074 | 0.036 |
| 1<br>2 | F42 | Obsessive-compulsive disorder | | 37<br>41 | 0.8%<br>0.8% | 0.649 | 0.009 |
| 1<br>2 | F30.2 | Manic episode, severe with psychotic symptoms | | 0<br>0 | 0%<br>0% | -- | -- |
| 1<br>2 | F31.2 | Bipolar disorder, current episode manic severe with psychotic features | | 0<br>10 | 0%<br>0.2% | 0.002 | 0.064 |
| 1<br>2 | F31.5 | Bipolar disorder, current episode depressed, severe, with psychotic features | | 0<br>10 | 0%<br>0.2% | 0.002 | 0.064 |
| 1<br>2 | F32.3 | Major depressive disorder, single episode, severe with psychotic features | | 10<br>10 | 0.2%<br>0.2% | 1 | <0.001 |
| 1<br>2 | F33.3 | Major depressive disorder, recurrent, severe with psychotic symptoms | | 10<br>10 | 0.2%<br>0.2% | 1 | <0.001 |
| **Medication** | | | | | | | |
| Cohort | | | Mean ± SD | Patients | % of Cohort | P-Value | Std diff. |
| 1<br>2 | R01AD | Corticosteroids | | 1,597<br>1,625 | 32.4%<br>33.0% | 0.548 | 0.012 |

## Supplementary Table S25. Baseline characteristics after propensity score matching low-dose methotrexate *vs* Tocilizumab (females)

| Cohort 1. Low-dose methotrexate (N = 1,316) and cohort 2. Tocilizumab (N = 1,316) | | | | | | | |
|---|---|---|---|---|---|---|---|
| **Demographics** | | | | | | | |
| Cohort | | | Mean ± SD | Patients | % of Cohort | P-Value | Std diff. |
| 1<br>2 | AI | Age at Index | 34.9 +/- 8.1<br>34.8 +/- 8.4 | 1,316<br>1,316 | 100%<br>100% | 0.620 | 0.019 |
| 1<br>2 | F | Female | | 1,316<br>1,316 | 100%<br>100% | -- | -- |
| 1<br>2 | 2054-5 | Black or African American | | 167<br>166 | 12.7%<br>12.6% | 0.953 | 0.002 |
| 1<br>2 | M | Male | | 0<br>0 | 0%<br>0% | -- | -- |
| 1<br>2 | 2106-3 | White | | 917<br>890 | 69.7%<br>67.6% | 0.257 | 0.044 |
| 1<br>2 | 1002-5 | American Indian or Alaska Native | | 19<br>22 | 1.4%<br>1.7% | 0.637 | 0.018 |
| 1<br>2 | UNK | Unknown Race | | 91<br>98 | 6.9%<br>7.4% | 0.597 | 0.021 |
| 1<br>2 | 2076-8 | Native Hawaiian or Other Pacific Islander | | 10<br>10 | 0.8%<br>0.8% | 1 | <0.001 |
| 1<br>2 | UN | Unknown Ethnicity | | 252<br>242 | 19.1%<br>18.4% | 0.618 | 0.019 |
| 1<br>2 | 2186-5 | Not Hispanic or Latino | | 925<br>917 | 70.3%<br>69.7% | 0.734 | 0.013 |
| 1<br>2 | 2135-2 | Hispanic or Latino | | 139<br>157 | 10.6%<br>11.9% | 0.267 | 0.043 |
| 1<br>2 | 2131-1 | Other Race | | 78<br>86 | 5.9%<br>6.5% | 0.519 | 0.025 |
| 1<br>2 | 2028-9 | Asian | | 40<br>47 | 3.0%<br>3.6% | 0.445 | 0.030 |
| **Diagnosis** | | | | | | | |
| Cohort | | | Mean ± SD | Patients | % of Cohort | P-Value | Std diff. |
| 1<br>2 | Z55 | Problems related to education and literacy | | 10<br>10 | 0.8%<br>0.8% | 1 | <0.001 |
| 1<br>2 | Z56 | Problems related to employment and unemployment | | 10<br>10 | 0.8%<br>0.8% | 1 | <0.001 |
| 1<br>2 | Z81 | Family history of mental and behavioral disorders | | 10<br>10 | 0.8%<br>0.8% | 1 | <0.001 |
| 1<br>2 | E70-E88 | Metabolic disorders | | 296<br>301 | 22.5%<br>22.9% | 0.816 | 0.009 |
| 1<br>2 | F31 | Bipolar disorder | | 26<br>39 | 2.0%<br>3.0% | 0.103 | 0.064 |
| 1<br>2 | F32 | Depressive episode | | 239<br>251 | 18.2%<br>19.1% | 0.548 | 0.023 |
| 1<br>2 | F33 | Major depressive disorder, recurrent | | 71<br>83 | 5.4%<br>6.3% | 0.319 | 0.039 |
| 1<br>2 | X71 | Intentional self-harm by drowning and submersion | | 0<br>0 | 0%<br>0% | -- | -- |

| | | | | | | | |
|---|---|---|---|---|---|---|---|
| 1<br>2 | R45.851 | Suicidal ideations | | 10<br>15 | 0.8%<br>1.1% | 0.315 | 0.039 |
| 1<br>2 | T14.91 | Suicide attempt | | 0<br>10 | 0%<br>0.8% | 0.002 | 0.124 |
| 1<br>2 | X71-X83 | Intentional self-harm | | 10<br>10 | 0.8%<br>0.8% | 1 | <0.001 |
| 1<br>2 | W54.0 | Bitten by dog | | 10<br>10 | 0.8%<br>0.8% | 1 | <0.001 |
| 1<br>2 | L60.0 | Ingrowing nail | | 10<br>14 | 0.8%<br>1.1% | 0.412 | 0.032 |
| 1<br>2 | B07 | Viral warts | | 21<br>33 | 1.6%<br>2.5% | 0.099 | 0.064 |
| 1<br>2 | I00-I99 | Diseases of the circulatory system | | 442<br>451 | 33.6%<br>34.3% | 0.711 | 0.014 |
| 1<br>2 | F41 | Other anxiety disorders | | 303<br>317 | 23.0%<br>24.1% | 0.520 | 0.025 |
| 1<br>2 | F41.1 | Generalized anxiety disorder | | 70<br>79 | 5.3%<br>6.0% | 0.448 | 0.030 |
| 1<br>2 | F40 | Phobic anxiety disorders | | 13<br>15 | 1.0%<br>1.1% | 0.704 | 0.015 |
| 1<br>2 | F41.8 | Other specified anxiety disorders | | 37<br>41 | 2.8%<br>3.1% | 0.646 | 0.018 |
| 1<br>2 | F41.0 | Panic disorder [episodic paroxysmal anxiety] | | 33<br>37 | 2.5%<br>2.8% | 0.628 | 0.019 |
| 1<br>2 | F41.3 | Other mixed anxiety disorders | | 0<br>0 | 0%<br>0% | -- | -- |
| 1<br>2 | F41.9 | Anxiety disorder, unspecified | | 257<br>268 | 19.5%<br>20.4% | 0.592 | 0.021 |
| 1<br>2 | F42 | Obsessive-compulsive disorder | | 10<br>10 | 0.8%<br>0.8% | 1 | <0.001 |
| 1<br>2 | F31.2 | Bipolar disorder, current episode manic severe with psychotic features | | 0<br>0 | 0%<br>0% | -- | -- |
| 1<br>2 | F31.5 | Bipolar disorder, current episode depressed, severe, with psychotic features | | 0<br>0 | 0%<br>0% | -- | -- |
| 1<br>2 | F32.3 | Major depressive disorder, single episode, severe with psychotic features | | 0<br>0 | 0%<br>0% | -- | -- |
| 1<br>2 | F33.3 | Major depressive disorder, recurrent, severe with psychotic symptoms | | 10<br>10 | 0.8%<br>0.8% | 1 | <0.001 |
| 1<br>2 | F30.2 | Manic episode, severe with psychotic symptoms | | 0<br>0 | 0%<br>0% | -- | -- |
| **Medication** | | | | | | | |
| Cohort | | | Mean ± SD | Patients | % of Cohort | P-Value | Std diff. |
| 1<br>2 | R01AD | Corticosteroids | | 557<br>561 | 42.3%<br>42.6% | 0.875 | 0.006 |

## Supplementary Table S26. Baseline characteristics after propensity score matching low-dose methotrexate *vs* Hydroxychloroquine (females)

| Cohort 1. Low-dose methotrexate (N = 17,318) and cohort 2. Hydroxychloroquine (N = 17,318) | | | | | | | |
|---|---|---|---|---|---|---|---|
| **Demographics** | | | | | | | |
| Cohort | | | Mean ± SD | Patients | % of Cohort | P-Value | Std diff. |
| 1<br>2 | AI | Age at Index | 35.0 +/- 8.0<br>35.1 +/- 7.3 | 17,318<br>17,318 | 100%<br>100% | 0.129 | 0.016 |
| 1<br>2 | F | Female | | 17,318<br>17,318 | 100%<br>100% | -- | -- |
| 1<br>2 | 2054-5 | Black or African American | | 2,510<br>2,564 | 14.5%<br>14.8% | 0.412 | 0.009 |
| 1<br>2 | M | Male | | 0<br>0 | 0%<br>0% | -- | -- |
| 1<br>2 | 2106-3 | White | | 11,423<br>11,279 | 66.0%<br>65.1% | 0.103 | 0.017 |
| 1<br>2 | 1002-5 | American Indian or Alaska Native | | 211<br>221 | 1.2%<br>1.3% | 0.628 | 0.005 |
| 1<br>2 | UNK | Unknown Race | | 1,597<br>1,631 | 9.2%<br>9.4% | 0.530 | 0.007 |
| 1<br>2 | 2076-8 | Native Hawaiian or Other Pacific Islander | | 84<br>89 | 0.5%<br>0.5% | 0.703 | 0.004 |
| 1<br>2 | UN | Unknown Ethnicity | | 3,495<br>3,514 | 20.2%<br>20.3% | 0.799 | 0.003 |
| 1<br>2 | 2186-5 | Not Hispanic or Latino | | 11,584<br>11,488 | 66.9%<br>66.3% | 0.274 | 0.012 |
| 1<br>2 | 2135-2 | Hispanic or Latino | | 2,239<br>2,316 | 12.9%<br>13.4% | 0.221 | 0.013 |
| 1<br>2 | 2131-1 | Other Race | | 791<br>820 | 4.6%<br>4.7% | 0.459 | 0.008 |
| 1<br>2 | 2028-9 | Asian | | 702<br>714 | 4.1%<br>4.1% | 0.745 | 0.003 |
| **Diagnosis** | | | | | | | |
| Cohort | | | Mean ± SD | Patients | % of Cohort | P-Value | Std diff. |
| 1<br>2 | Z55 | Problems related to education and literacy | | 24<br>26 | 0.1%<br>0.2% | 0.777 | 0.003 |
| 1<br>2 | Z56 | Problems related to employment and unemployment | | 54<br>61 | 0.3%<br>0.4% | 0.513 | 0.007 |
| 1<br>2 | Z81 | Family history of mental and behavioral disorders | | 102<br>104 | 0.6%<br>0.6% | 0.889 | 0.002 |
| 1<br>2 | E70-E88 | Metabolic disorders | | 3,233<br>3,287 | 18.7%<br>19.0% | 0.458 | 0.008 |
| 1<br>2 | F31 | Bipolar disorder | | 510<br>534 | 2.9%<br>3.1% | 0.451 | 0.008 |
| 1<br>2 | F32 | Depressive episode | | 3,116<br>3,174 | 18.0%<br>18.3% | 0.419 | 0.009 |
| 1<br>2 | F33 | Major depressive disorder, recurrent | | 784<br>828 | 4.5%<br>4.8% | 0.262 | 0.012 |
| 1<br>2 | X71 | Intentional self-harm by drowning and submersion | | 0<br>0 | 0%<br>0% | -- | -- |

| | | | | | | | |
|---|---|---|---|---|---|---|---|
| 1<br>2 | R45.851 | Suicidal ideations | | 207<br>218 | 1.2%<br>1.3% | 0.591 | 0.006 |
| 1<br>2 | T14.91 | Suicide attempt | | 19<br>21 | 0.1%<br>0.1% | 0.752 | 0.003 |
| 1<br>2 | X71-X83 | Intentional self-harm | | 24<br>28 | 0.1%<br>0.2% | 0.579 | 0.006 |
| 1<br>2 | W54.0 | Bitten by dog | | 57<br>54 | 0.3%<br>0.3% | 0.775 | 0.003 |
| 1<br>2 | L60.0 | Ingrowing nail | | 137<br>128 | 0.8%<br>0.7% | 0.579 | 0.006 |
| 1<br>2 | B07 | Viral warts | | 263<br>235 | 1.5%<br>1.4% | 0.206 | 0.014 |
| 1<br>2 | I00-I99 | Diseases of the circulatory system | | 5,325<br>5,397 | 30.7%<br>31.2% | 0.403 | 0.009 |
| 1<br>2 | F41 | Other anxiety disorders | | 3,760<br>3,863 | 21.7%<br>22.3% | 0.182 | 0.014 |
| 1<br>2 | F41.1 | Generalized anxiety disorder | | 1,210<br>1,253 | 7.0%<br>7.2% | 0.369 | 0.010 |
| 1<br>2 | F40 | Phobic anxiety disorders | | 158<br>163 | 0.9%<br>0.9% | 0.779 | 0.003 |
| 1<br>2 | F41.8 | Other specified anxiety disorders | | 584<br>597 | 3.4%<br>3.4% | 0.700 | 0.004 |
| 1<br>2 | F41.0 | Panic disorder [episodic paroxysmal anxiety] | | 397<br>415 | 2.3%<br>2.4% | 0.523 | 0.007 |
| 1<br>2 | F41.3 | Other mixed anxiety disorders | | 10<br>10 | 0.1%<br>0.1% | 1 | <0.001 |
| 1<br>2 | F41.9 | Anxiety disorder, unspecified | | 3,022<br>3,101 | 17.5%<br>17.9% | 0.266 | 0.012 |
| 1<br>2 | F42 | Obsessive-compulsive disorder | | 120<br>123 | 0.7%<br>0.7% | 0.847 | 0.002 |
| 1<br>2 | F31.2 | Bipolar disorder, current episode manic severe with psychotic features | | 10<br>10 | 0.1%<br>0.1% | 1 | <0.001 |
| 1<br>2 | F31.5 | Bipolar disorder, current episode depressed, severe, with psychotic features | | 10<br>10 | 0.1%<br>0.1% | 1 | <0.001 |
| 1<br>2 | F32.3 | Major depressive disorder, single episode, severe with psychotic features | | 11<br>12 | 0.1%<br>0.1% | 0.835 | 0.002 |
| 1<br>2 | F33.3 | Major depressive disorder, recurrent, severe with psychotic symptoms | | 15<br>18 | 0.1%<br>0.1% | 0.601 | 0.006 |
| 1<br>2 | F30.2 | Manic episode, severe with psychotic symptoms | | 0<br>0 | 0%<br>0% | -- | -- |
| **Medication** | | | | | | | |
| Cohort | | | Mean ± SD | Patients | % of Cohort | P-Value | Std diff. |
| 1<br>2 | R01AD | Corticosteroids | | 6,105<br>6,116 | 35.3%<br>35.3% | 0.902 | 0.001 |

## Supplementary Table S27. Baseline characteristics after propensity score matching low-dose methotrexate *vs* Leflunomide (females)

| Cohort 1. Low-dose methotrexate (N = 3,298) and cohort 2. Leflunomide (N = 3,298) | | | | | | | |
|---|---|---|---|---|---|---|---|
| **Demographics** | | | | | | | |
| Cohort | | | Mean ± SD | Patients | % of Cohort | P-Value | Std diff. |
| 1<br>2 | AI | Age at Index | 37.5 +/- 6.8<br>37.4 +/- 6.9 | 3,298<br>3,298 | 100%<br>100% | 0.610 | 0.013 |
| 1<br>2 | F | Female | | 3,298<br>3,298 | 100%<br>100% | -- | -- |
| 1<br>2 | 2054-5 | Black or African American | | 419<br>428 | 12.7%<br>13.0% | 0.740 | 0.008 |
| 1<br>2 | M | Male | | 0<br>0 | 0%<br>0% | -- | -- |
| 1<br>2 | 2106-3 | White | | 2,326<br>2,287 | 70.5%<br>69.3% | 0.295 | 0.026 |
| 1<br>2 | 1002-5 | American Indian or Alaska Native | | 33<br>43 | 1.0%<br>1.3% | 0.249 | 0.028 |
| 1<br>2 | UNK | Unknown Race | | 232<br>244 | 7.0%<br>7.4% | 0.568 | 0.014 |
| 1<br>2 | 2076-8 | Native Hawaiian or Other Pacific Islander | | 15<br>15 | 0.5%<br>0.5% | 1 | <0.001 |
| 1<br>2 | UN | Unknown Ethnicity | | 606<br>630 | 18.4%<br>19.1% | 0.449 | 0.019 |
| 1<br>2 | 2186-5 | Not Hispanic or Latino | | 2,217<br>2,197 | 67.2%<br>66.6% | 0.601 | 0.013 |
| 1<br>2 | 2135-2 | Hispanic or Latino | | 475<br>471 | 14.4%<br>14.3% | 0.888 | 0.003 |
| 1<br>2 | 2131-1 | Other Race | | 197<br>201 | 6.0%<br>6.1% | 0.836 | 0.005 |
| 1<br>2 | 2028-9 | Asian | | 76<br>80 | 2.3%<br>2.4% | 0.746 | 0.008 |
| **Diagnosis** | | | | | | | |
| Cohort | | | Mean ± SD | Patients | % of Cohort | P-Value | Std diff. |
| 1<br>2 | Z55 | Problems related to education and literacy | | 0<br>10 | 0%<br>0.3% | 0.002 | 0.078 |
| 1<br>2 | Z56 | Problems related to employment and unemployment | | 10<br>13 | 0.3%<br>0.4% | 0.531 | 0.015 |
| 1<br>2 | Z81 | Family history of mental and behavioral disorders | | 25<br>22 | 0.8%<br>0.7% | 0.661 | 0.011 |
| 1<br>2 | E70-E88 | Metabolic disorders | | 763<br>754 | 23.1%<br>22.9% | 0.792 | 0.006 |
| 1<br>2 | F31 | Bipolar disorder | | 100<br>124 | 3.0%<br>3.8% | 0.103 | 0.040 |
| 1<br>2 | F32 | Depressive episode | | 730<br>759 | 22.1%<br>23.0% | 0.393 | 0.021 |
| 1<br>2 | F33 | Major depressive disorder, recurrent | | 177<br>206 | 5.4%<br>6.2% | 0.127 | 0.038 |
| 1<br>2 | X71 | Intentional self-harm by drowning and submersion | | 0<br>0 | 0%<br>0% | -- | -- |

| | | | | | | | |
|---|---|---|---|---|---|---|---|
| 1<br>2 | R45.851 | Suicidal ideations | | 33<br>46 | 1.0%<br>1.4% | 0.141 | 0.036 |
| 1<br>2 | T14.91 | Suicide attempt | | 10<br>10 | 0.3%<br>0.3% | 1 | <0.001 |
| 1<br>2 | X71-X83 | Intentional self-harm | | 10<br>10 | 0.3%<br>0.3% | 1 | <0.001 |
| 1<br>2 | W54.0 | Bitten by dog | | 10<br>10 | 0.3%<br>0.3% | 1 | <0.001 |
| 1<br>2 | L60.0 | Ingrowing nail | | 23<br>27 | 0.7%<br>0.8% | 0.570 | 0.014 |
| 1<br>2 | B07 | Viral warts | | 52<br>53 | 1.6%<br>1.6% | 0.922 | 0.002 |
| 1<br>2 | I00-I99 | Diseases of the circulatory system | | 1,135<br>1,152 | 34.4%<br>34.9% | 0.660 | 0.011 |
| 1<br>2 | F41 | Other anxiety disorders | | 827<br>866 | 25.1%<br>26.3% | 0.272 | 0.027 |
| 1<br>2 | F41.1 | Generalized anxiety disorder | | 279<br>302 | 8.5%<br>9.2% | 0.318 | 0.025 |
| 1<br>2 | F40 | Phobic anxiety disorders | | 48<br>50 | 1.5%<br>1.5% | 0.839 | 0.005 |
| 1<br>2 | F41.8 | Other specified anxiety disorders | | 135<br>150 | 4.1%<br>4.5% | 0.364 | 0.022 |
| 1<br>2 | F41.0 | Panic disorder [episodic paroxysmal anxiety] | | 80<br>107 | 2.4%<br>3.2% | 0.045 | 0.049 |
| 1<br>2 | F41.3 | Other mixed anxiety disorders | | 10<br>10 | 0.3%<br>0.3% | 1 | <0.001 |
| 1<br>2 | F41.9 | Anxiety disorder, unspecified | | 674<br>693 | 20.4%<br>21.0% | 0.564 | 0.014 |
| 1<br>2 | F42 | Obsessive-compulsive disorder | | 35<br>27 | 1.1%<br>0.8% | 0.307 | 0.025 |
| 1<br>2 | F31.2 | Bipolar disorder, current episode manic severe with psychotic features | | 10<br>10 | 0.3%<br>0.3% | 1 | <0.001 |
| 1<br>2 | F31.5 | Bipolar disorder, current episode depressed, severe, with psychotic features | | 10<br>10 | 0.3%<br>0.3% | 1 | <0.001 |
| 1<br>2 | F32.3 | Major depressive disorder, single episode, severe with psychotic features | | 10<br>10 | 0.3%<br>0.3% | 1 | <0.001 |
| 1<br>2 | F33.3 | Major depressive disorder, recurrent, severe with psychotic symptoms | | 10<br>10 | 0.3%<br>0.3% | 1 | <0.001 |
| 1<br>2 | F30.2 | Manic episode, severe with psychotic symptoms | | 0<br>10 | 0%<br>0.3% | 0.002 | 0.078 |
| **Medication** | | | | | | | |
| Cohort | | | Mean ± SD | Patients | % of Cohort | P-Value | Std diff. |
| 1<br>2 | R01AD | Corticosteroids | | 1,300<br>1,318 | 39.4%<br>40.0% | 0.651 | 0.011 |

## Supplementary Table S28. Baseline characteristics after propensity score matching low-dose methotrexate *vs* Sulfasalazine (females)

| Cohort 1. Low-dose methotrexate (N = 5,439) and cohort 2. Sulfasalazine (N = 5,439) | | | | | | | |
|---|---|---|---|---|---|---|---|
| **Demographics** | | | | | | | |
| Cohort | | | Mean ± SD | Patients | % of Cohort | P-Value | Std diff. |
| 1<br>2 | AI | Age at Index | 34.5 +/- 7.7<br>34.7 +/- 7.3 | 5,439<br>5,439 | 100%<br>100% | 0.342 | 0.018 |
| 1<br>2 | F | Female | | 5,439<br>5,439 | 100%<br>100% | -- | -- |
| 1<br>2 | 2054-5 | Black or African American | | 562<br>569 | 10.3%<br>10.5% | 0.826 | 0.004 |
| 1<br>2 | M | Male | | 0<br>0 | 0%<br>0% | -- | -- |
| 1<br>2 | 2106-3 | White | | 3,915<br>3,858 | 72.0%<br>70.9% | 0.226 | 0.023 |
| 1<br>2 | 1002-5 | American Indian or Alaska Native | | 60<br>60 | 1.1%<br>1.1% | 1 | <0.001 |
| 1<br>2 | UNK | Unknown Race | | 394<br>408 | 7.2%<br>7.5% | 0.607 | 0.010 |
| 1<br>2 | 2076-8 | Native Hawaiian or Other Pacific Islander | | 38<br>47 | 0.7%<br>0.9% | 0.327 | 0.019 |
| 1<br>2 | UN | Unknown Ethnicity | | 1,101<br>1,079 | 20.2%<br>19.8% | 0.598 | 0.010 |
| 1<br>2 | 2186-5 | Not Hispanic or Latino | | 3,520<br>3,525 | 64.7%<br>64.8% | 0.920 | 0.002 |
| 1<br>2 | 2135-2 | Hispanic or Latino | | 818<br>835 | 15.0%<br>15.4% | 0.650 | 0.009 |
| 1<br>2 | 2131-1 | Other Race | | 250<br>277 | 4.6%<br>5.1% | 0.228 | 0.023 |
| 1<br>2 | 2028-9 | Asian | | 220<br>220 | 4.0%<br>4.0% | 1 | <0.001 |
| **Diagnosis** | | | | | | | |
| Cohort | | | Mean ± SD | Patients | % of Cohort | P-Value | Std diff. |
| 1<br>2 | Z55 | Problems related to education and literacy | | 10<br>10 | 0.2%<br>0.2% | 1 | <0.001 |
| 1<br>2 | Z56 | Problems related to employment and unemployment | | 20<br>16 | 0.4%<br>0.3% | 0.504 | 0.013 |
| 1<br>2 | Z81 | Family history of mental and behavioral disorders | | 32<br>36 | 0.6%<br>0.7% | 0.627 | 0.009 |
| 1<br>2 | E70-E88 | Metabolic disorders | | 1,031<br>1,060 | 19.0%<br>19.5% | 0.480 | 0.014 |
| 1<br>2 | F31 | Bipolar disorder | | 198<br>212 | 3.6%<br>3.9% | 0.481 | 0.014 |
| 1<br>2 | F32 | Depressive episode | | 1,090<br>1,128 | 20.0%<br>20.7% | 0.366 | 0.017 |
| 1<br>2 | F33 | Major depressive disorder, recurrent | | 286<br>338 | 5.3%<br>6.2% | 0.032 | 0.041 |
| 1<br>2 | X71 | Intentional self-harm by drowning and submersion | | 0<br>0 | 0%<br>0% | -- | -- |

| | | | | | | | |
|---|---|---|---|---|---|---|---|
| 1<br>2 | R45.851 | Suicidal ideations | | 77<br>97 | 1.4%<br>1.8% | 0.126 | 0.029 |
| 1<br>2 | T14.91 | Suicide attempt | | 11<br>11 | 0.2%<br>0.2% | 1 | <0.001 |
| 1<br>2 | X71-X83 | Intentional self-harm | | 10<br>10 | 0.2%<br>0.2% | 1 | <0.001 |
| 1<br>2 | W54.0 | Bitten by dog | | 15<br>14 | 0.3%<br>0.3% | 0.852 | 0.004 |
| 1<br>2 | L60.0 | Ingrowing nail | | 41<br>57 | 0.8%<br>1.0% | 0.104 | 0.031 |
| 1<br>2 | B07 | Viral warts | | 81<br>92 | 1.5%<br>1.7% | 0.399 | 0.016 |
| 1<br>2 | I00-I99 | Diseases of the circulatory system | | 1,526<br>1,536 | 28.1%<br>28.2% | 0.831 | 0.004 |
| 1<br>2 | F41 | Other anxiety disorders | | 1,267<br>1,363 | 23.3%<br>25.1% | 0.032 | 0.041 |
| 1<br>2 | F41.1 | Generalized anxiety disorder | | 395<br>456 | 7.3%<br>8.4% | 0.029 | 0.042 |
| 1<br>2 | F40 | Phobic anxiety disorders | | 52<br>69 | 1.0%<br>1.3% | 0.120 | 0.030 |
| 1<br>2 | F41.8 | Other specified anxiety disorders | | 184<br>224 | 3.4%<br>4.1% | 0.044 | 0.039 |
| 1<br>2 | F41.0 | Panic disorder [episodic paroxysmal anxiety] | | 138<br>170 | 2.5%<br>3.1% | 0.064 | 0.035 |
| 1<br>2 | F41.3 | Other mixed anxiety disorders | | 10<br>10 | 0.2%<br>0.2% | 1 | <0.001 |
| 1<br>2 | F41.9 | Anxiety disorder, unspecified | | 1,072<br>1,111 | 19.7%<br>20.4% | 0.350 | 0.018 |
| 1<br>2 | F42 | Obsessive-compulsive disorder | | 59<br>65 | 1.1%<br>1.2% | 0.588 | 0.010 |
| 1<br>2 | F31.2 | Bipolar disorder, current episode manic severe with psychotic features | | 0<br>0 | 0%<br>0% | -- | -- |
| 1<br>2 | F31.5 | Bipolar disorder, current episode depressed, severe, with psychotic features | | 10<br>10 | 0.2%<br>0.2% | 1 | <0.001 |
| 1<br>2 | F32.3 | Major depressive disorder, single episode, severe with psychotic features | | 0<br>10 | 0%<br>0.2% | 0.002 | 0.061 |
| 1<br>2 | F33.3 | Major depressive disorder, recurrent, severe with psychotic symptoms | | 10<br>10 | 0.2%<br>0.2% | 1 | <0.001 |
| 1<br>2 | F30.2 | Manic episode, severe with psychotic symptoms | | 0<br>0 | 0%<br>0% | -- | -- |
| **Medication** | | | | | | | |
| Cohort | | | Mean ± SD | Patients | % of Cohort | P-Value | Std diff. |
| 1<br>2 | R01AD | Corticosteroids | | 2,043<br>2,060 | 37.6%<br>37.9% | 0.737 | 0.006 |

## Supplementary Table S29. Baseline characteristics after propensity score matching low-dose methotrexate *vs* Minocycline (females)

| Cohort 1. Low-dose methotrexate (N = 880) and cohort 2. Minocycline (N = 880) | | | | | | | |
|---|---|---|---|---|---|---|---|
| **Demographics** | | | | | | | |
| Cohort | | | Mean ± SD | Patients | % of Cohort | P-Value | Std diff. |
| 1<br>2 | AI | Age at Index | 34.0 +/- 8.3<br>34.5 +/- 8.1 | 880<br>880 | 100%<br>100% | 0.168 | 0.066 |
| 1<br>2 | F | Female | | 880<br>880 | 100%<br>100% | -- | -- |
| 1<br>2 | 2054-5 | Black or African American | | 103<br>109 | 11.7%<br>12.4% | 0.660 | 0.021 |
| 1<br>2 | M | Male | | 0<br>0 | 0%<br>0% | -- | -- |
| 1<br>2 | 2106-3 | White | | 674<br>656 | 76.6%<br>74.5% | 0.318 | 0.048 |
| 1<br>2 | 1002-5 | American Indian or Alaska Native | | 10<br>10 | 1.1%<br>1.1% | 1 | <0.001 |
| 1<br>2 | UNK | Unknown Race | | 43<br>44 | 4.9%<br>5% | 0.912 | 0.005 |
| 1<br>2 | 2076-8 | Native Hawaiian or Other Pacific Islander | | 10<br>10 | 1.1%<br>1.1% | 1 | <0.001 |
| 1<br>2 | UN | Unknown Ethnicity | | 196<br>187 | 22.3%<br>21.3% | 0.603 | 0.025 |
| 1<br>2 | 2186-5 | Not Hispanic or Latino | | 638<br>626 | 72.5%<br>71.1% | 0.525 | 0.030 |
| 1<br>2 | 2135-2 | Hispanic or Latino | | 46<br>67 | 5.2%<br>7.6% | 0.041 | 0.097 |
| 1<br>2 | 2131-1 | Other Race | | 33<br>42 | 3.8%<br>4.8% | 0.288 | 0.051 |
| 1<br>2 | 2028-9 | Asian | | 16<br>17 | 1.8%<br>1.9% | 0.861 | 0.008 |
| **Diagnosis** | | | | | | | |
| Cohort | | | Mean ± SD | Patients | % of Cohort | P-Value | Std diff. |
| 1<br>2 | Z55 | Problems related to education and literacy | | 0<br>0 | 0%<br>0% | -- | -- |
| 1<br>2 | Z56 | Problems related to employment and unemployment | | 10<br>10 | 1.1%<br>1.1% | 1 | <0.001 |
| 1<br>2 | Z81 | Family history of mental and behavioral disorders | | 14<br>10 | 1.6%<br>1.1% | 0.411 | 0.039 |
| 1<br>2 | E70-E88 | Metabolic disorders | | 238<br>262 | 27.0%<br>29.8% | 0.205 | 0.061 |
| 1<br>2 | F31 | Bipolar disorder | | 48<br>57 | 5.5%<br>6.5% | 0.365 | 0.043 |
| 1<br>2 | F32 | Depressive episode | | 257<br>281 | 29.2%<br>31.9% | 0.214 | 0.059 |
| 1<br>2 | F33 | Major depressive disorder, recurrent | | 75<br>89 | 8.5%<br>10.1% | 0.251 | 0.055 |
| 1<br>2 | X71 | Intentional self-harm by drowning and submersion | | 0<br>0 | 0%<br>0% | -- | -- |

| | | | | | | | |
|---|---|---|---|---|---|---|---|
| 1<br>2 | R45.851 | Suicidal ideations | | 16<br>18 | 1.8%<br>2.0% | 0.729 | 0.017 |
| 1<br>2 | T14.91 | Suicide attempt | | 10<br>10 | 1.1%<br>1.1% | 1 | <0.001 |
| 1<br>2 | X71-X83 | Intentional self-harm | | 0<br>10 | 0%<br>1.1% | 0.002 | 0.152 |
| 1<br>2 | W54.0 | Bitten by dog | | 10<br>10 | 1.1%<br>1.1% | 1 | <0.001 |
| 1<br>2 | L60.0 | Ingrowing nail | | 16<br>12 | 1.8%<br>1.4% | 0.446 | 0.036 |
| 1<br>2 | B07 | Viral warts | | 24<br>20 | 2.7%<br>2.3% | 0.541 | 0.029 |
| 1<br>2 | I00-I99 | Diseases of the circulatory system | | 324<br>341 | 36.8%<br>38.8% | 0.403 | 0.040 |
| 1<br>2 | F41 | Other anxiety disorders | | 299<br>315 | 34.0%<br>35.8% | 0.424 | 0.038 |
| 1<br>2 | F41.1 | Generalized anxiety disorder | | 101<br>119 | 11.5%<br>13.5% | 0.195 | 0.062 |
| 1<br>2 | F40 | Phobic anxiety disorders | | 12<br>18 | 1.4%<br>2.0% | 0.269 | 0.053 |
| 1<br>2 | F41.8 | Other specified anxiety disorders | | 55<br>63 | 6.3%<br>7.2% | 0.446 | 0.036 |
| 1<br>2 | F41.0 | Panic disorder [episodic paroxysmal anxiety] | | 47<br>54 | 5.3%<br>6.1% | 0.473 | 0.034 |
| 1<br>2 | F41.3 | Other mixed anxiety disorders | | 0<br>10 | 0%<br>1.1% | 0.002 | 0.152 |
| 1<br>2 | F41.9 | Anxiety disorder, unspecified | | 259<br>262 | 29.4%<br>29.8% | 0.876 | 0.007 |
| 1<br>2 | F42 | Obsessive-compulsive disorder | | 10<br>10 | 1.1%<br>1.1% | 1 | <0.001 |
| 1<br>2 | F31.2 | Bipolar disorder, current episode manic severe with psychotic features | | 0<br>10 | 0%<br>1.1% | 0.002 | 0.152 |
| 1<br>2 | F31.5 | Bipolar disorder, current episode depressed, severe, with psychotic features | | 0<br>0 | 0%<br>0% | -- | -- |
| 1<br>2 | F32.3 | Major depressive disorder, single episode, severe with psychotic features | | 0<br>0 | 0%<br>0% | -- | -- |
| 1<br>2 | F33.3 | Major depressive disorder, recurrent, severe with psychotic symptoms | | 10<br>10 | 1.1%<br>1.1% | 1 | <0.001 |
| 1<br>2 | F30.2 | Manic episode, severe with psychotic symptoms | | 0<br>0 | 0%<br>0% | -- | -- |
| **Medication** | | | | | | | |
| Cohort | | | Mean ± SD | Patients | % of Cohort | P-Value | Std diff. |
| 1<br>2 | R01AD | Corticosteroids | | 456<br>460 | 51.8%<br>52.3% | 0.849 | 0.009 |

## Supplementary Table S30. Baseline characteristics after propensity score matching low-dose methotrexate *vs* Abatacept (females)

| Cohort 1. Low-dose methotrexate (N = 1,960) and cohort 2. Abatacept (N = 1,960) | | | | | | | |
|---|---|---|---|---|---|---|---|
| **Demographics** | | | | | | | |
| Cohort | | | Mean ± SD | Patients | % of Cohort | P-Value | Std diff. |
| 1<br>2 | AI | Age at Index | 35.8 +/- 7.6<br>35.7 +/- 7.6 | 1,960<br>1,960 | 100%<br>100% | 0.955 | 0.002 |
| 1<br>2 | F | Female | | 1,960<br>1,960 | 100%<br>100% | -- | -- |
| 1<br>2 | 2054-5 | Black or African American | | 234<br>238 | 11.9%<br>12.1% | 0.844 | 0.006 |
| 1<br>2 | M | Male | | 0<br>0 | 0%<br>0% | -- | -- |
| 1<br>2 | 2106-3 | White | | 1,398<br>1,391 | 71.3%<br>71.0% | 0.805 | 0.008 |
| 1<br>2 | 1002-5 | American Indian or Alaska Native | | 15<br>24 | 0.8%<br>1.2% | 0.148 | 0.046 |
| 1<br>2 | UNK | Unknown Race | | 162<br>151 | 8.3%<br>7.7% | 0.517 | 0.021 |
| 1<br>2 | 2076-8 | Native Hawaiian or Other Pacific Islander | | 10<br>10 | 0.5%<br>0.5% | 1 | <0.001 |
| 1<br>2 | UN | Unknown Ethnicity | | 342<br>349 | 17.4%<br>17.8% | 0.769 | 0.009 |
| 1<br>2 | 2186-5 | Not Hispanic or Latino | | 1,397<br>1,371 | 71.3%<br>69.9% | 0.362 | 0.029 |
| 1<br>2 | 2135-2 | Hispanic or Latino | | 221<br>240 | 11.3%<br>12.2% | 0.346 | 0.030 |
| 1<br>2 | 2131-1 | Other Race | | 97<br>106 | 4.9%<br>5.4% | 0.517 | 0.021 |
| 1<br>2 | 2028-9 | Asian | | 44<br>42 | 2.2%<br>2.1% | 0.827 | 0.007 |
| **Diagnosis** | | | | | | | |
| Cohort | | | Mean ± SD | Patients | % of Cohort | P-Value | Std diff. |
| 1<br>2 | Z55 | Problems related to education and literacy | | 10<br>10 | 0.5%<br>0.5% | 1 | <0.001 |
| 1<br>2 | Z56 | Problems related to employment and unemployment | | 10<br>10 | 0.5%<br>0.5% | 1 | <0.001 |
| 1<br>2 | Z81 | Family history of mental and behavioral disorders | | 10<br>10 | 0.5%<br>0.5% | 1 | <0.001 |
| 1<br>2 | E70-E88 | Metabolic disorders | | 381<br>405 | 19.4%<br>20.7% | 0.338 | 0.031 |
| 1<br>2 | F31 | Bipolar disorder | | 36<br>59 | 1.8%<br>3.0% | 0.017 | 0.076 |
| 1<br>2 | F32 | Depressive episode | | 366<br>409 | 18.7%<br>20.9% | 0.085 | 0.055 |
| 1<br>2 | F33 | Major depressive disorder, recurrent | | 88<br>112 | 4.5%<br>5.7% | 0.081 | 0.056 |
| 1<br>2 | X71 | Intentional self-harm by drowning and submersion | | 0<br>0 | 0%<br>0% | -- | -- |

| | | | | | | | |
|---|---|---|---|---|---|---|---|
| 1<br>2 | R45.851 | Suicidal ideations | | 10<br>19 | 0.5%<br>1.0% | 0.093 | 0.054 |
| 1<br>2 | T14.91 | Suicide attempt | | 0<br>0 | 0%<br>0% | -- | -- |
| 1<br>2 | X71-X83 | Intentional self-harm | | 0<br>10 | 0%<br>0.5% | 0.002 | 0.101 |
| 1<br>2 | W54.0 | Bitten by dog | | 10<br>10 | 0.5%<br>0.5% | 1 | <0.001 |
| 1<br>2 | L60.0 | Ingrowing nail | | 13<br>29 | 0.7%<br>1.5% | 0.013 | 0.079 |
| 1<br>2 | B07 | Viral warts | | 27<br>28 | 1.4%<br>1.4% | 0.892 | 0.004 |
| 1<br>2 | I00-I99 | Diseases of the circulatory system | | 636<br>648 | 32.4%<br>33.1% | 0.683 | 0.013 |
| 1<br>2 | F41 | Other anxiety disorders | | 436<br>477 | 22.2%<br>24.3% | 0.121 | 0.050 |
| 1<br>2 | F41.1 | Generalized anxiety disorder | | 112<br>146 | 5.7%<br>7.4% | 0.029 | 0.070 |
| 1<br>2 | F40 | Phobic anxiety disorders | | 15<br>20 | 0.8%<br>1.0% | 0.396 | 0.027 |
| 1<br>2 | F41.8 | Other specified anxiety disorders | | 77<br>82 | 3.9%<br>4.2% | 0.686 | 0.013 |
| 1<br>2 | F41.0 | Panic disorder [episodic paroxysmal anxiety] | | 53<br>58 | 2.7%<br>3.0% | 0.630 | 0.015 |
| 1<br>2 | F41.3 | Other mixed anxiety disorders | | 10<br>10 | 0.5%<br>0.5% | 1 | <0.001 |
| 1<br>2 | F41.9 | Anxiety disorder, unspecified | | 363<br>402 | 18.5%<br>20.5% | 0.116 | 0.050 |
| 1<br>2 | F42 | Obsessive-compulsive disorder | | 22<br>16 | 1.1%<br>0.8% | 0.328 | 0.031 |
| 1<br>2 | F31.2 | Bipolar disorder, current episode manic severe with psychotic features | | 0<br>10 | 0%<br>0.5% | 0.002 | 0.101 |
| 1<br>2 | F31.5 | Bipolar disorder, current episode depressed, severe, with psychotic features | | 0<br>0 | 0%<br>0% | -- | -- |
| 1<br>2 | F32.3 | Major depressive disorder, single episode, severe with psychotic features | | 0<br>10 | 0%<br>0.5% | 0.002 | 0.101 |
| 1<br>2 | F33.3 | Major depressive disorder, recurrent, severe with psychotic symptoms | | 10<br>10 | 0.5%<br>0.5% | 1 | <0.001 |
| 1<br>2 | F30.2 | Manic episode, severe with psychotic symptoms | | 0<br>0 | 0%<br>0% | -- | -- |
| **Medication** | | | | | | | |
| Cohort | | | Mean ± SD | Patients | % of Cohort | P-Value | Std diff. |
| 1<br>2 | R01AD | Corticosteroids | | 826<br>832 | 42.1%<br>42.4% | 0.846 | 0.006 |

## Supplementary Table S31. Baseline characteristics after propensity score matching low-dose methotrexate *vs* Tofacitinib (females)

| Cohort 1. Low-dose methotrexate (N = 1,797) and cohort 2. Tofacitinib (N = 1,797) | | | | | | | |
|---|---|---|---|---|---|---|---|
| **Demographics** | | | | | | | |
| Cohort | | | Mean ± SD | Patients | % of Cohort | P-Value | Std diff. |
| 1<br>2 | AI | Age at Index | 36.0 +/- 7.5<br>35.7 +/- 7.7 | 1,797<br>1,797 | 100%<br>100% | 0.211 | 0.042 |
| 1<br>2 | F | Female | | 1,797<br>1,797 | 100%<br>100% | -- | -- |
| 1<br>2 | 2054-5 | Black or African American | | 193<br>186 | 10.7%<br>10.4% | 0.704 | 0.013 |
| 1<br>2 | M | Male | | 0<br>0 | 0%<br>0% | -- | -- |
| 1<br>2 | 2106-3 | White | | 1,283<br>1,271 | 71.4%<br>70.7% | 0.659 | 0.015 |
| 1<br>2 | 1002-5 | American Indian or Alaska Native | | 23<br>24 | 1.3%<br>1.3% | 0.883 | 0.005 |
| 1<br>2 | UNK | Unknown Race | | 133<br>133 | 7.4%<br>7.4% | 1 | <0.001 |
| 1<br>2 | 2076-8 | Native Hawaiian or Other Pacific Islander | | 10<br>10 | 0.6%<br>0.6% | 1 | <0.001 |
| 1<br>2 | UN | Unknown Ethnicity | | 306<br>305 | 17.0%<br>17.0% | 0.965 | 0.001 |
| 1<br>2 | 2186-5 | Not Hispanic or Latino | | 1,287<br>1,278 | 71.6%<br>71.1% | 0.740 | 0.011 |
| 1<br>2 | 2135-2 | Hispanic or Latino | | 204<br>214 | 11.4%<br>11.9% | 0.603 | 0.017 |
| 1<br>2 | 2131-1 | Other Race | | 121<br>131 | 6.7%<br>7.3% | 0.514 | 0.022 |
| 1<br>2 | 2028-9 | Asian | | 40<br>47 | 2.2%<br>2.6% | 0.447 | 0.025 |
| **Diagnosis** | | | | | | | |
| Cohort | | | Mean ± SD | Patients | % of Cohort | P-Value | Std diff. |
| 1<br>2 | Z55 | Problems related to education and literacy | | 10<br>10 | 0.6%<br>0.6% | 1 | <0.001 |
| 1<br>2 | Z56 | Problems related to employment and unemployment | | 10<br>10 | 0.6%<br>0.6% | 1 | <0.001 |
| 1<br>2 | Z81 | Family history of mental and behavioral disorders | | 16<br>13 | 0.9%<br>0.7% | 0.576 | 0.019 |
| 1<br>2 | E70-E88 | Metabolic disorders | | 379<br>375 | 21.1%<br>20.9% | 0.870 | 0.005 |
| 1<br>2 | F31 | Bipolar disorder | | 47<br>61 | 2.6%<br>3.4% | 0.171 | 0.046 |
| 1<br>2 | F32 | Depressive episode | | 357<br>385 | 19.9%<br>21.4% | 0.249 | 0.039 |
| 1<br>2 | F33 | Major depressive disorder, recurrent | | 92<br>114 | 5.1%<br>6.3% | 0.114 | 0.053 |
| 1<br>2 | X71 | Intentional self-harm by drowning and submersion | | 0<br>0 | 0%<br>0% | -- | -- |

| | | | | | | | |
|---|---|---|---|---|---|---|---|
| 1<br>2 | R45.851 | Suicidal ideations | | 20<br>24 | 1.1%<br>1.3% | 0.544 | 0.020 |
| 1<br>2 | T14.91 | Suicide attempt | | 0<br>0 | 0%<br>0% | -- | -- |
| 1<br>2 | X71-X83 | Intentional self-harm | | 0<br>10 | 0%<br>0.6% | 0.002 | 0.106 |
| 1<br>2 | W54.0 | Bitten by dog | | 10<br>10 | 0.6%<br>0.6% | 1 | <0.001 |
| 1<br>2 | L60.0 | Ingrowing nail | | 17<br>16 | 0.9%<br>0.9% | 0.861 | 0.006 |
| 1<br>2 | B07 | Viral warts | | 35<br>30 | 1.9%<br>1.7% | 0.531 | 0.021 |
| 1<br>2 | I00-I99 | Diseases of the circulatory system | | 611<br>599 | 34.0%<br>33.3% | 0.672 | 0.014 |
| 1<br>2 | F41 | Other anxiety disorders | | 428<br>475 | 23.8%<br>26.4% | 0.071 | 0.060 |
| 1<br>2 | F41.1 | Generalized anxiety disorder | | 113<br>129 | 6.3%<br>7.2% | 0.287 | 0.036 |
| 1<br>2 | F40 | Phobic anxiety disorders | | 33<br>30 | 1.8%<br>1.7% | 0.703 | 0.013 |
| 1<br>2 | F41.8 | Other specified anxiety disorders | | 72<br>83 | 4.0%<br>4.6% | 0.366 | 0.030 |
| 1<br>2 | F41.0 | Panic disorder [episodic paroxysmal anxiety] | | 49<br>47 | 2.7%<br>2.6% | 0.836 | 0.007 |
| 1<br>2 | F41.3 | Other mixed anxiety disorders | | 10<br>10 | 0.6%<br>0.6% | 1 | <0.001 |
| 1<br>2 | F41.9 | Anxiety disorder, unspecified | | 356<br>392 | 19.8%<br>21.8% | 0.139 | 0.049 |
| 1<br>2 | F42 | Obsessive-compulsive disorder | | 10<br>13 | 0.6%<br>0.7% | 0.530 | 0.021 |
| 1<br>2 | F31.2 | Bipolar disorder, current episode manic severe with psychotic features | | 0<br>0 | 0%<br>0% | -- | -- |
| 1<br>2 | F31.5 | Bipolar disorder, current episode depressed, severe, with psychotic features | | 10<br>0 | 0.6%<br>0% | 0.002 | 0.106 |
| 1<br>2 | F32.3 | Major depressive disorder, single episode, severe with psychotic features | | 0<br>10 | 0%<br>0.6% | 0.002 | 0.106 |
| 1<br>2 | F33.3 | Major depressive disorder, recurrent, severe with psychotic symptoms | | 10<br>10 | 0.6%<br>0.6% | 1 | <0.001 |
| 1<br>2 | F30.2 | Manic episode, severe with psychotic symptoms | | 0<br>0 | 0%<br>0% | -- | -- |
| **Medication** | | | | | | | |
| Cohort | | | Mean ± SD | Patients | % of Cohort | P-Value | Std diff. |
| 1<br>2 | R01AD | Corticosteroids | | 789<br>787 | 43.9%<br>43.8% | 0.946 | 0.002 |

## Supplementary Table S32. Baseline characteristics after propensity score matching low-dose methotrexate *vs* Upadacitinib (females)

| Cohort 1. Low-dose methotrexate (N = 1,169) and cohort 2. Upadacitinib (N = 1,169) | | | | | | | |
|---|---|---|---|---|---|---|---|
| **Demographics** | | | | | | | |
| Cohort | | | Mean ± SD | Patients | % of Cohort | P-Value | Std diff. |
| 1<br>2 | AI | Age at Index | 35.9 +/- 7.4<br>35.7 +/- 7.5 | 1,169<br>1,169 | 100%<br>100% | 0.483 | 0.029 |
| 1<br>2 | F | Female | | 1,169<br>1,169 | 100%<br>100% | -- | -- |
| 1<br>2 | 2054-5 | Black or African American | | 133<br>124 | 11.4%<br>10.6% | 0.552 | 0.025 |
| 1<br>2 | M | Male | | 0<br>0 | 0%<br>0% | -- | -- |
| 1<br>2 | 2106-3 | White | | 848<br>848 | 72.5%<br>72.5% | 1 | <0.001 |
| 1<br>2 | 1002-5 | American Indian or Alaska Native | | 27<br>19 | 2.3%<br>1.6% | 0.234 | 0.049 |
| 1<br>2 | UNK | Unknown Race | | 78<br>88 | 6.7%<br>7.5% | 0.421 | 0.033 |
| 1<br>2 | 2076-8 | Native Hawaiian or Other Pacific Islander | | 10<br>10 | 0.9%<br>0.9% | 1 | <0.001 |
| 1<br>2 | UN | Unknown Ethnicity | | 242<br>243 | 20.7%<br>20.8% | 0.959 | 0.002 |
| 1<br>2 | 2186-5 | Not Hispanic or Latino | | 814<br>801 | 69.6%<br>68.5% | 0.561 | 0.024 |
| 1<br>2 | 2135-2 | Hispanic or Latino | | 113<br>125 | 9.7%<br>10.7% | 0.412 | 0.034 |
| 1<br>2 | 2131-1 | Other Race | | 35<br>41 | 3.0%<br>3.5% | 0.484 | 0.029 |
| 1<br>2 | 2028-9 | Asian | | 44<br>45 | 3.8%<br>3.8% | 0.914 | 0.004 |
| **Diagnosis** | | | | | | | |
| Cohort | | | Mean ± SD | Patients | % of Cohort | P-Value | Std diff. |
| 1<br>2 | Z55 | Problems related to education and literacy | | 10<br>10 | 0.9%<br>0.9% | 1 | <0.001 |
| 1<br>2 | Z56 | Problems related to employment and unemployment | | 10<br>10 | 0.9%<br>0.9% | 1 | <0.001 |
| 1<br>2 | Z81 | Family history of mental and behavioral disorders | | 10<br>10 | 0.9%<br>0.9% | 1 | <0.001 |
| 1<br>2 | E70-E88 | Metabolic disorders | | 270<br>273 | 23.1%<br>23.4% | 0.883 | 0.006 |
| 1<br>2 | F31 | Bipolar disorder | | 48<br>54 | 4.1%<br>4.6% | 0.544 | 0.025 |
| 1<br>2 | F32 | Depressive episode | | 271<br>274 | 23.2%<br>23.4% | 0.883 | 0.006 |
| 1<br>2 | F33 | Major depressive disorder, recurrent | | 98<br>101 | 8.4%<br>8.6% | 0.824 | 0.009 |
| 1<br>2 | X71 | Intentional self-harm by drowning and submersion | | 0<br>0 | 0%<br>0% | -- | -- |

| | | | | | | | |
|---|---|---|---|---|---|---|---|
| 1<br>2 | R45.851 | Suicidal ideations | | 19<br>23 | 1.6%<br>2.0% | 0.533 | 0.026 |
| 1<br>2 | T14.91 | Suicide attempt | | 10<br>10 | 0.9%<br>0.9% | 1 | <0.001 |
| 1<br>2 | X71-X83 | Intentional self-harm | | 0<br>0 | 0%<br>0% | -- | -- |
| 1<br>2 | W54.0 | Bitten by dog | | 10<br>10 | 0.9%<br>0.9% | 1 | <0.001 |
| 1<br>2 | L60.0 | Ingrowing nail | | 12<br>12 | 1.0%<br>1.0% | 1 | <0.001 |
| 1<br>2 | B07 | Viral warts | | 21<br>28 | 1.8%<br>2.4% | 0.312 | 0.042 |
| 1<br>2 | I00-I99 | Diseases of the circulatory system | | 408<br>389 | 34.9%<br>33.3% | 0.407 | 0.034 |
| 1<br>2 | F41 | Other anxiety disorders | | 363<br>380 | 31.1%<br>32.5% | 0.450 | 0.031 |
| 1<br>2 | F41.1 | Generalized anxiety disorder | | 105<br>122 | 9.0%<br>10.4% | 0.235 | 0.049 |
| 1<br>2 | F40 | Phobic anxiety disorders | | 20<br>26 | 1.7%<br>2.2% | 0.372 | 0.037 |
| 1<br>2 | F41.8 | Other specified anxiety disorders | | 64<br>80 | 5.5%<br>6.8% | 0.169 | 0.057 |
| 1<br>2 | F41.0 | Panic disorder [episodic paroxysmal anxiety] | | 37<br>43 | 3.2%<br>3.7% | 0.495 | 0.028 |
| 1<br>2 | F41.3 | Other mixed anxiety disorders | | 10<br>10 | 0.9%<br>0.9% | 1 | <0.001 |
| 1<br>2 | F41.9 | Anxiety disorder, unspecified | | 300<br>312 | 25.7%<br>26.7% | 0.572 | 0.023 |
| 1<br>2 | F42 | Obsessive-compulsive disorder | | 15<br>17 | 1.3%<br>1.5% | 0.722 | 0.015 |
| **Medication** | | | | | | | |
| Cohort | | | Mean ± SD | Patients | % of Cohort | P-Value | Std diff. |
| 1<br>2 | R01AD | Corticosteroids | | 615<br>586 | 52.6%<br>50.1% | 0.230 | 0.050 |

**Supplementary Table S33. Baseline characteristics after propensity score matching low-dose methotrexate *vs* Naproxen (males)**

| Cohort 1. Low-dose methotrexate (N = 2,604) and cohort 2. Naproxen (N = 2,604) | | | | | | | |
|---|---|---|---|---|---|---|---|
| **Demographics** | | | | | | | |
| Cohort | | | Mean ± SD | Patients | % of Cohort | P-Value | Std diff. |
| 1<br>2 | AI | Age at Index | 33.8 +/- 9.2<br>33.7 +/- 9.7 | 2,604<br>2,604 | 100%<br>100% | 0.522 | 0.018 |
| 1<br>2 | F | Female | | 0<br>0 | 0%<br>0% | -- | -- |
| 1<br>2 | 2054-5 | Black or African American | | 436<br>400 | 16.7%<br>15.4% | 0.174 | 0.038 |
| 1<br>2 | M | Male | | 2,604<br>2,604 | 100%<br>100% | -- | -- |
| 1<br>2 | 2106-3 | White | | 1,726<br>1,735 | 66.3%<br>66.6% | 0.792 | 0.007 |
| 1<br>2 | 1002-5 | American Indian or Alaska Native | | 16<br>15 | 0.6%<br>0.6% | 0.857 | 0.005 |
| 1<br>2 | UNK | Unknown Race | | 161<br>181 | 6.2%<br>7.0% | 0.263 | 0.031 |
| 1<br>2 | 2076-8 | Native Hawaiian or Other Pacific Islander | | 30<br>27 | 1.2%<br>1.0% | 0.689 | 0.011 |
| 1<br>2 | UN | Unknown Ethnicity | | 349<br>349 | 13.4%<br>13.4% | 1 | <0.001 |
| 1<br>2 | 2186-5 | Not Hispanic or Latino | | 1,972<br>1,948 | 75.7%<br>74.8% | 0.441 | 0.021 |
| 1<br>2 | 2135-2 | Hispanic or Latino | | 283<br>307 | 10.9%<br>11.8% | 0.294 | 0.029 |
| 1<br>2 | 2131-1 | Other Race | | 139<br>143 | 5.3%<br>5.5% | 0.807 | 0.007 |
| 1<br>2 | 2028-9 | Asian | | 96<br>103 | 3.7%<br>4.0% | 0.613 | 0.014 |
| **Diagnosis** | | | | | | | |
| Cohort | | | Mean ± SD | Patients | % of Cohort | P-Value | Std diff. |
| 1<br>2 | Z55 | Problems related to education and literacy | | 10<br>10 | 0.4%<br>0.4% | 1 | <0.001 |
| 1<br>2 | Z56 | Problems related to employment and unemployment | | 10<br>10 | 0.4%<br>0.4% | 1 | <0.001 |
| 1<br>2 | Z81 | Family history of mental and behavioral disorders | | 10<br>12 | 0.4%<br>0.5% | 0.669 | 0.012 |
| 1<br>2 | E70-E88 | Metabolic disorders | | 718<br>726 | 27.6%<br>27.9% | 0.804 | 0.007 |
| 1<br>2 | F31 | Bipolar disorder | | 56<br>67 | 2.2%<br>2.6% | 0.315 | 0.028 |
| 1<br>2 | F32 | Depressive episode | | 346<br>364 | 13.3%<br>14.0% | 0.467 | 0.020 |
| 1<br>2 | F33 | Major depressive disorder, recurrent | | 99<br>96 | 3.8%<br>3.7% | 0.827 | 0.006 |
| 1<br>2 | X71 | Intentional self-harm by drowning and submersion | | 0<br>0 | 0%<br>0% | -- | -- |

| | | | | | | | |
|---|---|---|---|---|---|---|---|
| 1<br>2 | R45.851 | Suicidal ideations | | 41<br>43 | 1.6%<br>1.7% | 0.826 | 0.006 |
| 1<br>2 | T14.91 | Suicide attempt | | 10<br>10 | 0.4%<br>0.4% | 1 | <0.001 |
| 1<br>2 | X71-X83 | Intentional self-harm | | 10<br>10 | 0.4%<br>0.4% | 1 | <0.001 |
| 1<br>2 | W54.0 | Bitten by dog | | 10<br>10 | 0.4%<br>0.4% | 1 | <0.001 |
| 1<br>2 | L60.0 | Ingrowing nail | | 31<br>19 | 1.2%<br>0.7% | 0.088 | 0.047 |
| 1<br>2 | B07 | Viral warts | | 56<br>60 | 2.2%<br>2.3% | 0.707 | 0.010 |
| 1<br>2 | I00-I99 | Diseases of the circulatory system | | 950<br>925 | 36.5%<br>35.5% | 0.470 | 0.020 |
| 1<br>2 | F41 | Other anxiety disorders | | 435<br>461 | 16.7%<br>17.7% | 0.340 | 0.026 |
| 1<br>2 | F41.1 | Generalized anxiety disorder | | 136<br>150 | 5.2%<br>5.8% | 0.394 | 0.024 |
| 1<br>2 | F40 | Phobic anxiety disorders | | 18<br>23 | 0.7%<br>0.9% | 0.433 | 0.022 |
| 1<br>2 | F41.8 | Other specified anxiety disorders | | 60<br>60 | 2.3%<br>2.3% | 1 | <0.001 |
| 1<br>2 | F41.0 | Panic disorder [episodic paroxysmal anxiety] | | 39<br>45 | 1.5%<br>1.7% | 0.509 | 0.018 |
| 1<br>2 | F41.3 | Other mixed anxiety disorders | | 0<br>0 | 0%<br>0% | -- | -- |
| 1<br>2 | F41.9 | Anxiety disorder, unspecified | | 345<br>370 | 13.2%<br>14.2% | 0.314 | 0.028 |
| 1<br>2 | F42 | Obsessive-compulsive disorder | | 16<br>17 | 0.6%<br>0.7% | 0.861 | 0.005 |
| 1<br>2 | F31.2 | Bipolar disorder, current episode manic severe with psychotic features | | 0<br>0 | 0%<br>0% | -- | -- |
| 1<br>2 | F31.5 | Bipolar disorder, current episode depressed, severe, with psychotic features | | 0<br>0 | 0%<br>0% | -- | -- |
| 1<br>2 | F32.3 | Major depressive disorder, single episode, severe with psychotic features | | 0<br>10 | 0%<br>0.4% | 0.002 | 0.088 |
| 1<br>2 | F33.3 | Major depressive disorder, recurrent, severe with psychotic symptoms | | 10<br>10 | 0.4%<br>0.4% | 1 | <0.001 |
| 1<br>2 | F30.2 | Manic episode, severe with psychotic symptoms | | 0<br>0 | 0%<br>0% | -- | -- |
| **Medication** | | | | | | | |
| Cohort | | | Mean ± SD | Patients | % of Cohort | P-Value | Std diff. |
| 1<br>2 | R01AD | Corticosteroids | | 987<br>950 | 37.9%<br>36.5% | 0.289 | 0.029 |

## Supplementary Table S34. Baseline characteristics after propensity score matching low-dose methotrexate *vs* Diclofenac (males)

| Cohort 1. Low-dose methotrexate (N = 1,013) and cohort 2. Diclofenac (N = 1,013) | | | | | | | |
|---|---|---|---|---|---|---|---|
| **Demographics** | | | | | | | |
| Cohort | | | Mean ± SD | Patients | % of Cohort | P-Value | Std diff. |
| 1<br>2 | AI | Age at Index | 35.0 +/- 8.3<br>35.4 +/- 7.9 | 1,013<br>1,013 | 100%<br>100% | 0.366 | 0.040 |
| 1<br>2 | F | Female | | 0<br>0 | 0%<br>0% | -- | -- |
| 1<br>2 | 2054-5 | Black or African American | | 135<br>129 | 13.3%<br>12.7% | 0.692 | 0.018 |
| 1<br>2 | M | Male | | 1,013<br>1,013 | 100%<br>100% | -- | -- |
| 1<br>2 | 2106-3 | White | | 749<br>740 | 73.9%<br>73.1% | 0.651 | 0.020 |
| 1<br>2 | 1002-5 | American Indian or Alaska Native | | 10<br>10 | 1.0%<br>1.0% | 1 | <0.001 |
| 1<br>2 | UNK | Unknown Race | | 69<br>72 | 6.8%<br>7.1% | 0.793 | 0.012 |
| 1<br>2 | 2076-8 | Native Hawaiian or Other Pacific Islander | | 10<br>10 | 1.0%<br>1.0% | 1 | <0.001 |
| 1<br>2 | UN | Unknown Ethnicity | | 144<br>158 | 14.2%<br>15.6% | 0.382 | 0.039 |
| 1<br>2 | 2186-5 | Not Hispanic or Latino | | 782<br>755 | 77.2%<br>74.5% | 0.161 | 0.062 |
| 1<br>2 | 2135-2 | Hispanic or Latino | | 87<br>100 | 8.6%<br>9.9% | 0.318 | 0.044 |
| 1<br>2 | 2131-1 | Other Race | | 31<br>34 | 3.1%<br>3.4% | 0.705 | 0.017 |
| 1<br>2 | 2028-9 | Asian | | 24<br>28 | 2.4%<br>2.8% | 0.574 | 0.025 |
| **Diagnosis** | | | | | | | |
| Cohort | | | Mean ± SD | Patients | % of Cohort | P-Value | Std diff. |
| 1<br>2 | Z55 | Problems related to education and literacy | | 10<br>10 | 1.0%<br>1.0% | 1 | <0.001 |
| 1<br>2 | Z56 | Problems related to employment and unemployment | | 10<br>10 | 1.0%<br>1.0% | 1 | <0.001 |
| 1<br>2 | Z81 | Family history of mental and behavioral disorders | | 10<br>10 | 1.0%<br>1.0% | 1 | <0.001 |
| 1<br>2 | E70-E88 | Metabolic disorders | | 259<br>269 | 25.6%<br>26.6% | 0.613 | 0.022 |
| 1<br>2 | F31 | Bipolar disorder | | 28<br>30 | 2.8%<br>3.0% | 0.790 | 0.012 |
| 1<br>2 | F32 | Depressive episode | | 169<br>177 | 16.7%<br>17.5% | 0.637 | 0.021 |
| 1<br>2 | F33 | Major depressive disorder, recurrent | | 36<br>45 | 3.6%<br>4.4% | 0.307 | 0.045 |
| 1<br>2 | X71 | Intentional self-harm by drowning and submersion | | 0<br>0 | 0%<br>0% | -- | -- |

| | | | | | | | |
|---|---|---|---|---|---|---|---|
| 1<br>2 | R45.851 | Suicidal ideations | | 18<br>22 | 1.8%<br>2.2% | 0.523 | 0.028 |
| 1<br>2 | T14.91 | Suicide attempt | | 0<br>0 | 0%<br>0% | -- | -- |
| 1<br>2 | X71-X83 | Intentional self-harm | | 10<br>10 | 1.0%<br>1.0% | 1 | <0.001 |
| 1<br>2 | W54.0 | Bitten by dog | | 10<br>10 | 1.0%<br>1.0% | 1 | <0.001 |
| 1<br>2 | L60.0 | Ingrowing nail | | 11<br>18 | 1.1%<br>1.8% | 0.190 | 0.058 |
| 1<br>2 | B07 | Viral warts | | 28<br>28 | 2.8%<br>2.8% | 1 | <0.001 |
| 1<br>2 | I00-I99 | Diseases of the circulatory system | | 351<br>353 | 34.6%<br>34.8% | 0.926 | 0.004 |
| 1<br>2 | F41 | Other anxiety disorders | | 206<br>227 | 20.3%<br>22.4% | 0.255 | 0.051 |
| 1<br>2 | F41.1 | Generalized anxiety disorder | | 72<br>90 | 7.1%<br>8.9% | 0.140 | 0.066 |
| 1<br>2 | F40 | Phobic anxiety disorders | | 10<br>10 | 1.0%<br>1.0% | 1 | <0.001 |
| 1<br>2 | F41.8 | Other specified anxiety disorders | | 37<br>31 | 3.7%<br>3.1% | 0.459 | 0.033 |
| 1<br>2 | F41.0 | Panic disorder [episodic paroxysmal anxiety] | | 17<br>27 | 1.7%<br>2.7% | 0.127 | 0.068 |
| 1<br>2 | F41.3 | Other mixed anxiety disorders | | 0<br>0 | 0%<br>0% | -- | -- |
| 1<br>2 | F41.9 | Anxiety disorder, unspecified | | 175<br>177 | 17.3%<br>17.5% | 0.907 | 0.005 |
| 1<br>2 | F42 | Obsessive-compulsive disorder | | 10<br>10 | 1.0%<br>1.0% | 1 | <0.001 |
| 1<br>2 | F31.2 | Bipolar disorder, current episode manic severe with psychotic features | | 10<br>10 | 1.0%<br>1.0% | 1 | <0.001 |
| 1<br>2 | F31.5 | Bipolar disorder, current episode depressed, severe, with psychotic features | | 0<br>0 | 0%<br>0% | -- | -- |
| 1<br>2 | F32.3 | Major depressive disorder, single episode, severe with psychotic features | | 10<br>10 | 1.0%<br>1.0% | 1 | <0.001 |
| 1<br>2 | F33.3 | Major depressive disorder, recurrent, severe with psychotic symptoms | | 10<br>10 | 1.0%<br>1.0% | 1 | <0.001 |
| 1<br>2 | F30.2 | Manic episode, severe with psychotic symptoms | | 0<br>0 | 0%<br>0% | -- | -- |
| **Medication** | | | | | | | |
| Cohort | | | Mean ± SD | Patients | % of Cohort | P-Value | Std diff. |
| 1<br>2 | R01AD | Corticosteroids | | 419<br>403 | 41.4%<br>39.8% | 0.469 | 0.032 |

## Supplementary Table S35. Baseline characteristics after propensity score matching low-dose methotrexate *vs* Celecoxib (males)

| Cohort 1. Low-dose methotrexate (N = 1,601) and cohort 2. Celecoxib (N = 1,601) | | | | | | | |
|---|---|---|---|---|---|---|---|
| **Demographics** | | | | | | | |
| Cohort | | | Mean ± SD | Patients | % of Cohort | P-Value | Std diff. |
| 1<br>2 | AI | Age at Index | 36.2 +/- 8.0<br>36.3 +/- 7.7 | 1,601<br>1,601 | 100%<br>100% | 0.770 | 0.010 |
| 1<br>2 | F | Female | | 0<br>0 | 0%<br>0% | -- | -- |
| 1<br>2 | 2054-5 | Black or African American | | 174<br>164 | 10.9%<br>10.2% | 0.565 | 0.020 |
| 1<br>2 | M | Male | | 1,601<br>1,601 | 100%<br>100% | -- | -- |
| 1<br>2 | 2106-3 | White | | 1,184<br>1,178 | 74.0%<br>73.6% | 0.810 | 0.009 |
| 1<br>2 | 1002-5 | American Indian or Alaska Native | | 16<br>21 | 1.0%<br>1.3% | 0.408 | 0.029 |
| 1<br>2 | UNK | Unknown Race | | 80<br>91 | 5.0%<br>5.7% | 0.387 | 0.031 |
| 1<br>2 | 2076-8 | Native Hawaiian or Other Pacific Islander | | 10<br>10 | 0.6%<br>0.6% | 1 | <0.001 |
| 1<br>2 | UN | Unknown Ethnicity | | 250<br>239 | 15.6%<br>14.9% | 0.589 | 0.019 |
| 1<br>2 | 2186-5 | Not Hispanic or Latino | | 1,192<br>1,181 | 74.5%<br>73.8% | 0.657 | 0.016 |
| 1<br>2 | 2135-2 | Hispanic or Latino | | 159<br>181 | 9.9%<br>11.3% | 0.207 | 0.045 |
| 1<br>2 | 2131-1 | Other Race | | 76<br>82 | 4.7%<br>5.1% | 0.624 | 0.017 |
| 1<br>2 | 2028-9 | Asian | | 63<br>55 | 3.9%<br>3.4% | 0.453 | 0.027 |
| **Diagnosis** | | | | | | | |
| Cohort | | | Mean ± SD | Patients | % of Cohort | P-Value | Std diff. |
| 1<br>2 | Z55 | Problems related to education and literacy | | 10<br>10 | 0.6%<br>0.6% | 1 | <0.001 |
| 1<br>2 | Z56 | Problems related to employment and unemployment | | 10<br>11 | 0.6%<br>0.7% | 0.827 | 0.008 |
| 1<br>2 | Z81 | Family history of mental and behavioral disorders | | 14<br>14 | 0.9%<br>0.9% | 1 | <0.001 |
| 1<br>2 | E70-E88 | Metabolic disorders | | 525<br>524 | 32.8%<br>32.7% | 0.970 | 0.001 |
| 1<br>2 | F31 | Bipolar disorder | | 45<br>52 | 2.8%<br>3.2% | 0.470 | 0.026 |
| 1<br>2 | F32 | Depressive episode | | 265<br>300 | 16.6%<br>18.7% | 0.105 | 0.057 |
| 1<br>2 | F33 | Major depressive disorder, recurrent | | 91<br>98 | 5.7%<br>6.1% | 0.600 | 0.019 |
| 1<br>2 | X71 | Intentional self-harm by drowning and submersion | | 0<br>0 | 0%<br>0% | -- | -- |

| | | | | | | | |
|---|---|---|---|---|---|---|---|
| 1<br>2 | R45.851 | Suicidal ideations | | 36<br>36 | 2.2%<br>2.2% | 1 | <0.001 |
| 1<br>2 | T14.91 | Suicide attempt | | 10<br>0 | 0.6%<br>0% | 0.002 | 0.112 |
| 1<br>2 | X71-X83 | Intentional self-harm | | 10<br>10 | 0.6%<br>0.6% | 1 | <0.001 |
| 1<br>2 | W54.0 | Bitten by dog | | 10<br>10 | 0.6%<br>0.6% | 1 | <0.001 |
| 1<br>2 | L60.0 | Ingrowing nail | | 21<br>28 | 1.3%<br>1.7% | 0.314 | 0.036 |
| 1<br>2 | B07 | Viral warts | | 36<br>36 | 2.2%<br>2.2% | 1 | <0.001 |
| 1<br>2 | I00-I99 | Diseases of the circulatory system | | 700<br>691 | 43.7%<br>43.2% | 0.748 | 0.011 |
| 1<br>2 | F41 | Other anxiety disorders | | 380<br>408 | 23.7%<br>25.5% | 0.251 | 0.041 |
| 1<br>2 | F41.1 | Generalized anxiety disorder | | 118<br>138 | 7.4%<br>8.6% | 0.193 | 0.046 |
| 1<br>2 | F40 | Phobic anxiety disorders | | 16<br>14 | 1.0%<br>0.9% | 0.714 | 0.013 |
| 1<br>2 | F41.8 | Other specified anxiety disorders | | 52<br>60 | 3.2%<br>3.7% | 0.442 | 0.027 |
| 1<br>2 | F41.0 | Panic disorder [episodic paroxysmal anxiety] | | 47<br>49 | 2.9%<br>3.1% | 0.836 | 0.007 |
| 1<br>2 | F41.3 | Other mixed anxiety disorders | | 0<br>0 | 0%<br>0% | -- | -- |
| 1<br>2 | F41.9 | Anxiety disorder, unspecified | | 313<br>327 | 19.6%<br>20.4% | 0.536 | 0.022 |
| 1<br>2 | F42 | Obsessive-compulsive disorder | | 14<br>17 | 0.9%<br>1.1% | 0.588 | 0.019 |
| 1<br>2 | F31.2 | Bipolar disorder, current episode manic severe with psychotic features | | 0<br>0 | 0%<br>0% | -- | -- |
| 1<br>2 | F31.5 | Bipolar disorder, current episode depressed, severe, with psychotic features | | 0<br>0 | 0%<br>0% | -- | -- |
| 1<br>2 | F32.3 | Major depressive disorder, single episode, severe with psychotic features | | 10<br>10 | 0.6%<br>0.6% | 1 | <0.001 |
| 1<br>2 | F33.3 | Major depressive disorder, recurrent, severe with psychotic symptoms | | 10<br>10 | 0.6%<br>0.6% | 1 | <0.001 |
| 1<br>2 | F30.2 | Manic episode, severe with psychotic symptoms | | 0<br>0 | 0%<br>0% | -- | -- |
| **Medication** | | | | | | | |
| Cohort | | | Mean ± SD | Patients | % of Cohort | P-Value | Std diff. |
| 1<br>2 | R01AD | Corticosteroids | | 836<br>812 | 52.2%<br>50.7% | 0.396 | 0.030 |

**Supplementary Table S36. Baseline characteristics after propensity score matching low-dose methotrexate *vs* Infliximab (males)**

| Cohort 1. Low-dose methotrexate (N = 301) and cohort 2. Infliximab (N = 301) | | | | | | | |
|---|---|---|---|---|---|---|---|
| **Demographics** | | | | | | | |
| Cohort | | | Mean ± SD | Patients | % of Cohort | P-Value | Std diff. |
| 1<br>2 | AI | Age at Index | 33.4 +/- 8.7<br>33.2 +/- 8.3 | 301<br>301 | 100%<br>100% | 0.833 | 0.017 |
| 1<br>2 | F | Female | | 0<br>0 | 0%<br>0% | -- | -- |
| 1<br>2 | 2054-5 | Black or African American | | 49<br>51 | 16.3%<br>16.9% | 0.827 | 0.018 |
| 1<br>2 | M | Male | | 301<br>301 | 100%<br>100% | -- | -- |
| 1<br>2 | 2106-3 | White | | 214<br>213 | 71.1%<br>70.8% | 0.928 | 0.007 |
| 1<br>2 | 1002-5 | American Indian or Alaska Native | | 10<br>10 | 3.3%<br>3.3% | 1 | <0.001 |
| 1<br>2 | UNK | Unknown Race | | 10<br>10 | 3.3%<br>3.3% | 1 | <0.001 |
| 1<br>2 | 2076-8 | Native Hawaiian or Other Pacific Islander | | 10<br>10 | 3.3%<br>3.3% | 1 | <0.001 |
| 1<br>2 | UN | Unknown Ethnicity | | 18<br>24 | 6.0%<br>8.0% | 0.337 | 0.078 |
| 1<br>2 | 2186-5 | Not Hispanic or Latino | | 257<br>246 | 85.4%<br>81.7% | 0.226 | 0.099 |
| 1<br>2 | 2135-2 | Hispanic or Latino | | 26<br>31 | 8.6%<br>10.3% | 0.486 | 0.057 |
| 1<br>2 | 2131-1 | Other Race | | 18<br>17 | 6.0%<br>5.6% | 0.862 | 0.014 |
| 1<br>2 | 2028-9 | Asian | | 10<br>10 | 3.3%<br>3.3% | 1 | <0.001 |
| **Diagnosis** | | | | | | | |
| Cohort | | | Mean ± SD | Patients | % of Cohort | P-Value | Std diff. |
| 1<br>2 | Z55 | Problems related to education and literacy | | 0<br>10 | 0%<br>3.3% | 0.001 | 0.262 |
| 1<br>2 | Z56 | Problems related to employment and unemployment | | 10<br>10 | 3.3%<br>3.3% | 1 | <0.001 |
| 1<br>2 | Z81 | Family history of mental and behavioral disorders | | 10<br>10 | 3.3%<br>3.3% | 1 | <0.001 |
| 1<br>2 | E70-E88 | Metabolic disorders | | 84<br>96 | 27.9%<br>31.9% | 0.285 | 0.087 |
| 1<br>2 | F31 | Bipolar disorder | | 10<br>10 | 3.3%<br>3.3% | 1 | <0.001 |
| 1<br>2 | F32 | Depressive episode | | 56<br>51 | 18.6%<br>16.9% | 0.594 | 0.043 |
| 1<br>2 | F33 | Major depressive disorder, recurrent | | 10<br>12 | 3.3%<br>4.0% | 0.664 | 0.035 |
| 1<br>2 | X71 | Intentional self-harm by drowning and submersion | | 0<br>0 | 0%<br>0% | -- | -- |

| | | | | | | | |
|---|---|---|---|---|---|---|---|
| 1<br>2 | R45.851 | Suicidal ideations | | 11<br>10 | 3.7%<br>3.3% | 0.824 | 0.018 |
| 1<br>2 | T14.91 | Suicide attempt | | 0<br>0 | 0%<br>0% | -- | -- |
| 1<br>2 | X71-X83 | Intentional self-harm | | 0<br>0 | 0%<br>0% | -- | -- |
| 1<br>2 | W54.0 | Bitten by dog | | 10<br>0 | 3.3%<br>0% | 0.001 | 0.262 |
| 1<br>2 | L60.0 | Ingrowing nail | | 10<br>10 | 3.3%<br>3.3% | 1 | <0.001 |
| 1<br>2 | B07 | Viral warts | | 10<br>10 | 3.3%<br>3.3% | 1 | <0.001 |
| 1<br>2 | I00-I99 | Diseases of the circulatory system | | 99<br>100 | 32.9%<br>33.2% | 0.931 | 0.007 |
| 1<br>2 | F41 | Other anxiety disorders | | 45<br>54 | 15.0%<br>17.9% | 0.322 | 0.081 |
| 1<br>2 | F41.1 | Generalized anxiety disorder | | 19<br>20 | 6.3%<br>6.6% | 0.868 | 0.013 |
| 1<br>2 | F40 | Phobic anxiety disorders | | 10<br>10 | 3.3%<br>3.3% | 1 | <0.001 |
| 1<br>2 | F41.8 | Other specified anxiety disorders | | 10<br>10 | 3.3%<br>3.3% | 1 | <0.001 |
| 1<br>2 | F41.0 | Panic disorder [episodic paroxysmal anxiety] | | 10<br>10 | 3.3%<br>3.3% | 1 | <0.001 |
| 1<br>2 | F41.3 | Other mixed anxiety disorders | | 0<br>0 | 0%<br>0% | -- | -- |
| 1<br>2 | F41.9 | Anxiety disorder, unspecified | | 37<br>45 | 12.3%<br>15.0% | 0.342 | 0.078 |
| 1<br>2 | F42 | Obsessive-compulsive disorder | | 10<br>10 | 3.3%<br>3.3% | 1 | <0.001 |
| 1<br>2 | F31.2 | Bipolar disorder, current episode manic severe with psychotic features | | 0<br>0 | 0%<br>0% | -- | -- |
| 1<br>2 | F31.5 | Bipolar disorder, current episode depressed, severe, with psychotic features | | 0<br>0 | 0%<br>0% | -- | -- |
| 1<br>2 | F32.3 | Major depressive disorder, single episode, severe with psychotic features | | 0<br>0 | 0%<br>0% | -- | -- |
| 1<br>2 | F33.3 | Major depressive disorder, recurrent, severe with psychotic symptoms | | 0<br>0 | 0%<br>0% | -- | -- |
| 1<br>2 | F30.2 | Manic episode, severe with psychotic symptoms | | 0<br>0 | 0%<br>0% | -- | -- |
| **Medication** | | | | | | | |
| Cohort | | | Mean ± SD | Patients | % of Cohort | P-Value | Std diff. |
| 1<br>2 | R01AD | Corticosteroids | | 127<br>120 | 42.2%<br>39.9% | 0.562 | 0.047 |

## Supplementary Table S37. Baseline characteristics after propensity score matching low-dose methotrexate *vs* Adalimumab (males)

| Cohort 1. Low-dose methotrexate (N = 1,606) and cohort 2. Adalimumab (N = 1,606) | | | | | | | |
|---|---|---|---|---|---|---|---|
| **Demographics** | | | | | | | |
| Cohort | | | Mean ± SD | Patients | % of Cohort | P-Value | Std diff. |
| 1<br>2 | AI | Age at Index | 33.8 +/- 8.9<br>34.1 +/- 8.6 | 1,606<br>1,606 | 100%<br>100% | 0.379 | 0.031 |
| 1<br>2 | F | Female | | 0<br>0 | 0%<br>0% | -- | -- |
| 1<br>2 | 2054-5 | Black or African American | | 165<br>161 | 10.3%<br>10.0% | 0.815 | 0.008 |
| 1<br>2 | M | Male | | 1,606<br>1,606 | 100%<br>100% | -- | -- |
| 1<br>2 | 2106-3 | White | | 1,207<br>1,198 | 75.2%<br>74.6% | 0.714 | 0.013 |
| 1<br>2 | 1002-5 | American Indian or Alaska Native | | 12<br>21 | 0.7%<br>1.3% | 0.115 | 0.056 |
| 1<br>2 | UNK | Unknown Race | | 108<br>109 | 6.7%<br>6.8% | 0.944 | 0.002 |
| 1<br>2 | 2076-8 | Native Hawaiian or Other Pacific Islander | | 10<br>10 | 0.6%<br>0.6% | 1 | <0.001 |
| 1<br>2 | UN | Unknown Ethnicity | | 203<br>207 | 12.6%<br>12.9% | 0.832 | 0.007 |
| 1<br>2 | 2186-5 | Not Hispanic or Latino | | 1,250<br>1,227 | 77.8%<br>76.4% | 0.334 | 0.034 |
| 1<br>2 | 2135-2 | Hispanic or Latino | | 153<br>172 | 9.5%<br>10.7% | 0.266 | 0.039 |
| 1<br>2 | 2131-1 | Other Race | | 75<br>74 | 4.7%<br>4.6% | 0.933 | 0.003 |
| 1<br>2 | 2028-9 | Asian | | 36<br>40 | 2.2%<br>2.5% | 0.642 | 0.016 |
| **Diagnosis** | | | | | | | |
| Cohort | | | Mean ± SD | Patients | % of Cohort | P-Value | Std diff. |
| 1<br>2 | Z55 | Problems related to education and literacy | | 10<br>10 | 0.6%<br>0.6% | 1 | <0.001 |
| 1<br>2 | Z56 | Problems related to employment and unemployment | | 10<br>10 | 0.6%<br>0.6% | 1 | <0.001 |
| 1<br>2 | Z81 | Family history of mental and behavioral disorders | | 10<br>10 | 0.6%<br>0.6% | 1 | <0.001 |
| 1<br>2 | E70-E88 | Metabolic disorders | | 322<br>350 | 20.0%<br>21.8% | 0.225 | 0.043 |
| 1<br>2 | F31 | Bipolar disorder | | 21<br>29 | 1.3%<br>1.8% | 0.254 | 0.040 |
| 1<br>2 | F32 | Depressive episode | | 158<br>176 | 9.8%<br>11.0% | 0.298 | 0.037 |
| 1<br>2 | F33 | Major depressive disorder, recurrent | | 44<br>53 | 2.7%<br>3.3% | 0.353 | 0.033 |
| 1<br>2 | X71 | Intentional self-harm by drowning and submersion | | 0<br>0 | 0%<br>0% | -- | -- |

| | | | | | | |
|---|---|---|---|---|---|---|
| 1<br>2 | R45.851 | Suicidal ideations | 14<br>19 | 0.9%<br>1.2% | 0.382 | 0.031 |
| 1<br>2 | T14.91 | Suicide attempt | 0<br>0 | 0%<br>0% | -- | -- |
| 1<br>2 | X71-X83 | Intentional self-harm | 0<br>10 | 0%<br>0.6% | 0.002 | 0.112 |
| 1<br>2 | W54.0 | Bitten by dog | 10<br>10 | 0.6%<br>0.6% | 1 | <0.001 |
| 1<br>2 | L60.0 | Ingrowing nail | 17<br>12 | 1.1%<br>0.7% | 0.351 | 0.033 |
| 1<br>2 | B07 | Viral warts | 35<br>30 | 2.2%<br>1.9% | 0.531 | 0.022 |
| 1<br>2 | I00-I99 | Diseases of the circulatory system | 422<br>440 | 26.3%<br>27.4% | 0.474 | 0.025 |
| 1<br>2 | F41 | Other anxiety disorders | 224<br>263 | 13.9%<br>16.4% | 0.055 | 0.068 |
| 1<br>2 | F41.1 | Generalized anxiety disorder | 57<br>79 | 3.5%<br>4.9% | 0.054 | 0.068 |
| 1<br>2 | F40 | Phobic anxiety disorders | 13<br>14 | 0.8%<br>0.9% | 0.847 | 0.007 |
| 1<br>2 | F41.8 | Other specified anxiety disorders | 38<br>35 | 2.4%<br>2.2% | 0.722 | 0.013 |
| 1<br>2 | F41.0 | Panic disorder [episodic paroxysmal anxiety] | 21<br>25 | 1.3%<br>1.6% | 0.552 | 0.021 |
| 1<br>2 | F41.3 | Other mixed anxiety disorders | 0<br>0 | 0%<br>0% | -- | -- |
| 1<br>2 | F41.9 | Anxiety disorder, unspecified | 175<br>207 | 10.9%<br>12.9% | 0.081 | 0.062 |
| 1<br>2 | F42 | Obsessive-compulsive disorder | 10<br>15 | 0.6%<br>0.9% | 0.315 | 0.035 |
| 1<br>2 | F31.2 | Bipolar disorder, current episode manic severe with psychotic features | 0<br>0 | 0%<br>0% | -- | -- |
| 1<br>2 | F31.5 | Bipolar disorder, current episode depressed, severe, with psychotic features | 0<br>0 | 0%<br>0% | -- | -- |
| 1<br>2 | F32.3 | Major depressive disorder, single episode, severe with psychotic features | 0<br>0 | 0%<br>0% | -- | -- |
| 1<br>2 | F33.3 | Major depressive disorder, recurrent, severe with psychotic symptoms | 0<br>10 | 0%<br>0.6% | 0.002 | 0.112 |
| 1<br>2 | F30.2 | Manic episode, severe with psychotic symptoms | 0<br>0 | 0%<br>0% | -- | -- |
| **Medication** | | | | | | |
| Cohort | | Mean ± SD | Patients | % of Cohort | P-Value | Std diff. |
| 1<br>2 | R01AD | Corticosteroids | 559<br>560 | 34.8%<br>34.9% | 0.970 | 0.001 |

## Supplementary Table S38. Baseline characteristics after propensity score matching low-dose methotrexate *vs* Etanercept (males)

| Cohort 1. Low-dose methotrexate (N = 1,188) and cohort 2. Etanercept (N = 1,188) | | | | | | | |
|---|---|---|---|---|---|---|---|
| **Demographics** | | | | | | | |
| Cohort | | | Mean ± SD | Patients | % of Cohort | P-Value | Std diff. |
| 1<br>2 | AI | Age at Index | 35.5 +/- 8.0<br>35.4 +/- 8.2 | 1,188<br>1,188 | 100%<br>100% | 0.723 | 0.015 |
| 1<br>2 | F | Female | | 0<br>0 | 0%<br>0% | -- | -- |
| 1<br>2 | 2054-5 | Black or African American | | 110<br>96 | 9.3%<br>8.1% | 0.307 | 0.042 |
| 1<br>2 | M | Male | | 1,188<br>1,188 | 100%<br>100% | -- | -- |
| 1<br>2 | 2106-3 | White | | 890<br>893 | 74.9%<br>75.2% | 0.887 | 0.006 |
| 1<br>2 | 1002-5 | American Indian or Alaska Native | | 10<br>16 | 0.8%<br>1.3% | 0.237 | 0.049 |
| 1<br>2 | UNK | Unknown Race | | 82<br>83 | 6.9%<br>7.0% | 0.936 | 0.003 |
| 1<br>2 | 2076-8 | Native Hawaiian or Other Pacific Islander | | 10<br>10 | 0.8%<br>0.8% | 1 | <0.001 |
| 1<br>2 | UN | Unknown Ethnicity | | 210<br>218 | 17.7%<br>18.4% | 0.669 | 0.018 |
| 1<br>2 | 2186-5 | Not Hispanic or Latino | | 855<br>840 | 72.0%<br>70.7% | 0.496 | 0.028 |
| 1<br>2 | 2135-2 | Hispanic or Latino | | 123<br>130 | 10.4%<br>10.9% | 0.642 | 0.019 |
| 1<br>2 | 2131-1 | Other Race | | 63<br>68 | 5.3%<br>5.7% | 0.653 | 0.018 |
| 1<br>2 | 2028-9 | Asian | | 30<br>29 | 2.5%<br>2.4% | 0.895 | 0.005 |
| **Diagnosis** | | | | | | | |
| Cohort | | | Mean ± SD | Patients | % of Cohort | P-Value | Std diff. |
| 1<br>2 | Z55 | Problems related to education and literacy | | 10<br>10 | 0.8%<br>0.8% | 1 | <0.001 |
| 1<br>2 | Z56 | Problems related to employment and unemployment | | 10<br>10 | 0.8%<br>0.8% | 1 | <0.001 |
| 1<br>2 | Z81 | Family history of mental and behavioral disorders | | 10<br>10 | 0.8%<br>0.8% | 1 | <0.001 |
| 1<br>2 | E70-E88 | Metabolic disorders | | 192<br>211 | 16.2%<br>17.8% | 0.299 | 0.043 |
| 1<br>2 | F31 | Bipolar disorder | | 18<br>22 | 1.5%<br>1.9% | 0.524 | 0.026 |
| 1<br>2 | F32 | Depressive episode | | 95<br>115 | 8.0%<br>9.7% | 0.148 | 0.059 |
| 1<br>2 | F33 | Major depressive disorder, recurrent | | 22<br>38 | 1.9%<br>3.2% | 0.036 | 0.086 |
| 1<br>2 | X71 | Intentional self-harm by drowning and submersion | | 0<br>0 | 0%<br>0% | -- | -- |

| | | | | | | |
|---|---|---|---|---|---|---|
| 1<br>2 | R45.851 | Suicidal ideations | 10<br>10 | 0.8%<br>0.8% | 1 | <0.001 |
| 1<br>2 | T14.91 | Suicide attempt | 0<br>0 | 0%<br>0% | -- | -- |
| 1<br>2 | X71-X83 | Intentional self-harm | 0<br>10 | 0%<br>0.8% | 0.002 | 0.130 |
| 1<br>2 | W54.0 | Bitten by dog | 10<br>10 | 0.8%<br>0.8% | 1 | <0.001 |
| 1<br>2 | L60.0 | Ingrowing nail | 10<br>10 | 0.8%<br>0.8% | 1 | <0.001 |
| 1<br>2 | B07 | Viral warts | 19<br>24 | 1.6%<br>2.0% | 0.442 | 0.032 |
| 1<br>2 | I00-I99 | Diseases of the circulatory system | 325<br>320 | 27.4%<br>26.9% | 0.818 | 0.009 |
| 1<br>2 | F41 | Other anxiety disorders | 101<br>147 | 8.5%<br>12.4% | 0.002 | 0.127 |
| 1<br>2 | F41.1 | Generalized anxiety disorder | 34<br>51 | 2.9%<br>4.3% | 0.060 | 0.077 |
| 1<br>2 | F40 | Phobic anxiety disorders | 10<br>10 | 0.8%<br>0.8% | 1 | <0.001 |
| 1<br>2 | F41.8 | Other specified anxiety disorders | 10<br>12 | 0.8%<br>1.0% | 0.668 | 0.018 |
| 1<br>2 | F41.0 | Panic disorder [episodic paroxysmal anxiety] | 10<br>15 | 0.8%<br>1.3% | 0.315 | 0.041 |
| 1<br>2 | F41.3 | Other mixed anxiety disorders | 0<br>0 | 0%<br>0% | -- | -- |
| 1<br>2 | F41.9 | Anxiety disorder, unspecified | 85<br>115 | 7.2%<br>9.7% | 0.027 | 0.091 |
| 1<br>2 | F42 | Obsessive-compulsive disorder | 10<br>10 | 0.8%<br>0.8% | 1 | <0.001 |
| 1<br>2 | F31.2 | Bipolar disorder, current episode manic severe with psychotic features | 0<br>0 | 0%<br>0% | -- | -- |
| 1<br>2 | F31.5 | Bipolar disorder, current episode depressed, severe, with psychotic features | 0<br>0 | 0%<br>0% | -- | -- |
| 1<br>2 | F32.3 | Major depressive disorder, single episode, severe with psychotic features | 0<br>0 | 0%<br>0% | -- | -- |
| 1<br>2 | F33.3 | Major depressive disorder, recurrent, severe with psychotic symptoms | 10<br>10 | 0.8%<br>0.8% | 1 | <0.001 |
| 1<br>2 | F30.2 | Manic episode, severe with psychotic symptoms | 0<br>0 | 0%<br>0% | -- | -- |
| **Medication** | | | | | | |
| Cohort | | Mean ± SD | Patients | % of Cohort | P-Value | Std diff. |
| 1<br>2 | R01AD | Corticosteroids | 285<br>316 | 24.0%<br>26.6% | 0.143 | 0.060 |

## Supplementary Table S39. Baseline characteristics after propensity score matching low-dose methotrexate *vs* Tocilizumab (males)

| Cohort 1. Low-dose methotrexate (N = 227) and cohort 2. Tocilizumab (N = 227) | | | | | | | |
|---|---|---|---|---|---|---|---|
| **Demographics** | | | | | | | |
| Cohort | | | Mean ± SD | Patients | % of Cohort | P-Value | Std diff. |
| 1<br>2 | AI | Age at Index | 32.3 +/- 10.0<br>32.9 +/- 9.9 | 227<br>227 | 100%<br>100% | 0.541 | 0.057 |
| 1<br>2 | F | Female | | 0<br>0 | 0%<br>0% | -- | -- |
| 1<br>2 | 2054-5 | Black or African American | | 36<br>32 | 15.9%<br>14.1% | 0.599 | 0.049 |
| 1<br>2 | M | Male | | 227<br>227 | 100%<br>100% | -- | -- |
| 1<br>2 | 2106-3 | White | | 158<br>157 | 69.6%<br>69.2% | 0.919 | 0.010 |
| 1<br>2 | 1002-5 | American Indian or Alaska Native | | 0<br>0 | 0%<br>0% | -- | -- |
| 1<br>2 | UNK | Unknown Race | | 15<br>19 | 6.6%<br>8.4% | 0.476 | 0.067 |
| 1<br>2 | 2076-8 | Native Hawaiian or Other Pacific Islander | | 0<br>0 | 0%<br>0% | -- | -- |
| 1<br>2 | UN | Unknown Ethnicity | | 44<br>43 | 19.4%<br>18.9% | 0.905 | 0.011 |
| 1<br>2 | 2186-5 | Not Hispanic or Latino | | 160<br>159 | 70.5%<br>70.0% | 0.918 | 0.010 |
| 1<br>2 | 2135-2 | Hispanic or Latino | | 23<br>25 | 10.1%<br>11.0% | 0.760 | 0.029 |
| 1<br>2 | 2131-1 | Other Race | | 15<br>15 | 6.6%<br>6.6% | 1 | <0.001 |
| 1<br>2 | 2028-9 | Asian | | 10<br>10 | 4.4%<br>4.4% | 1 | <0.001 |
| **Diagnosis** | | | | | | | |
| Cohort | | | Mean ± SD | Patients | % of Cohort | P-Value | Std diff. |
| 1<br>2 | Z55 | Problems related to education and literacy | | 0<br>10 | 0%<br>4.4% | 0.001 | 0.304 |
| 1<br>2 | Z56 | Problems related to employment and unemployment | | 10<br>10 | 4.4%<br>4.4% | 1 | <0.001 |
| 1<br>2 | Z81 | Family history of mental and behavioral disorders | | 0<br>0 | 0%<br>0% | -- | -- |
| 1<br>2 | E70-E88 | Metabolic disorders | | 62<br>70 | 27.3%<br>30.8% | 0.408 | 0.078 |
| 1<br>2 | F31 | Bipolar disorder | | 10<br>10 | 4.4%<br>4.4% | 1 | <0.001 |
| 1<br>2 | F32 | Depressive episode | | 17<br>27 | 7.5%<br>11.9% | 0.113 | 0.149 |
| 1<br>2 | F33 | Major depressive disorder, recurrent | | 0<br>10 | 0%<br>4.4% | 0.001 | 0.304 |
| 1<br>2 | X71 | Intentional self-harm by drowning and submersion | | 0<br>0 | 0%<br>0% | -- | -- |

| | | | | | | | |
|---|---|---|---|---|---|---|---|
| 1<br>2 | R45.851 | Suicidal ideations | | 10<br>10 | 4.4%<br>4.4% | 1 | <0.001 |
| 1<br>2 | T14.91 | Suicide attempt | | 0<br>0 | 0%<br>0% | -- | -- |
| 1<br>2 | X71-X83 | Intentional self-harm | | 0<br>0 | 0%<br>0% | -- | -- |
| 1<br>2 | W54.0 | Bitten by dog | | 0<br>0 | 0%<br>0% | -- | -- |
| 1<br>2 | L60.0 | Ingrowing nail | | 10<br>10 | 4.4%<br>4.4% | 1 | <0.001 |
| 1<br>2 | B07 | Viral warts | | 10<br>10 | 4.4%<br>4.4% | 1 | <0.001 |
| 1<br>2 | I00-I99 | Diseases of the circulatory system | | 81<br>84 | 35.7%<br>37.0% | 0.770 | 0.027 |
| 1<br>2 | F41 | Other anxiety disorders | | 22<br>35 | 9.7%<br>15.4% | 0.066 | 0.173 |
| 1<br>2 | F41.1 | Generalized anxiety disorder | | 10<br>10 | 4.4%<br>4.4% | 1 | <0.001 |
| 1<br>2 | F40 | Phobic anxiety disorders | | 0<br>10 | 0%<br>4.4% | 0.001 | 0.304 |
| 1<br>2 | F41.8 | Other specified anxiety disorders | | 10<br>10 | 4.4%<br>4.4% | 1 | <0.001 |
| 1<br>2 | F41.0 | Panic disorder [episodic paroxysmal anxiety] | | 10<br>10 | 4.4%<br>4.4% | 1 | <0.001 |
| 1<br>2 | F41.3 | Other mixed anxiety disorders | | 0<br>0 | 0%<br>0% | -- | -- |
| 1<br>2 | F41.9 | Anxiety disorder, unspecified | | 21<br>31 | 9.3%<br>13.7% | 0.141 | 0.139 |
| 1<br>2 | F42 | Obsessive-compulsive disorder | | 10<br>10 | 4.4%<br>4.4% | 1 | <0.001 |
| 1<br>2 | F31.2 | Bipolar disorder, current episode manic severe with psychotic features | | 0<br>0 | 0%<br>0% | -- | -- |
| 1<br>2 | F31.5 | Bipolar disorder, current episode depressed, severe, with psychotic features | | 0<br>0 | 0%<br>0% | -- | -- |
| 1<br>2 | F32.3 | Major depressive disorder, single episode, severe with psychotic features | | 0<br>0 | 0%<br>0% | -- | -- |
| 1<br>2 | F33.3 | Major depressive disorder, recurrent, severe with psychotic symptoms | | 0<br>0 | 0%<br>0% | -- | -- |
| 1<br>2 | F30.2 | Manic episode, severe with psychotic symptoms | | 0<br>0 | 0%<br>0% | -- | -- |

**Medication**

| Cohort | | | Mean ± SD | Patients | % of Cohort | P-Value | Std diff. |
|---|---|---|---|---|---|---|---|
| 1<br>2 | R01AD | Corticosteroids | | 88<br>90 | 38.8%<br>39.6% | 0.848 | 0.018 |

## Supplementary Table S40. Baseline characteristics after propensity score matching low-dose methotrexate *vs* Hydroxychloroquine (males)

| Cohort 1. Low-dose methotrexate (N = 2,437) and cohort 2. Hydroxychloroquine (N = 2,437) | | | | | | | |
|---|---|---|---|---|---|---|---|
| **Demographics** | | | | | | | |
| Cohort | | | Mean ± SD | Patients | % of Cohort | P-Value | Std diff. |
| 1<br>2 | AI | Age at Index | 36.5 +/- 7.6<br>36.0 +/- 7.6 | 2,437<br>2,437 | 100%<br>100% | 0.027 | 0.063 |
| 1<br>2 | F | Female | | 0<br>0 | 0%<br>0% | -- | -- |
| 1<br>2 | 2054-5 | Black or African American | | 290<br>298 | 11.9%<br>12.2% | 0.725 | 0.010 |
| 1<br>2 | M | Male | | 2,437<br>2,437 | 100%<br>100% | -- | -- |
| 1<br>2 | 2106-3 | White | | 1,731<br>1,687 | 71.0%<br>69.2% | 0.169 | 0.039 |
| 1<br>2 | 1002-5 | American Indian or Alaska Native | | 18<br>22 | 0.7%<br>0.9% | 0.525 | 0.018 |
| 1<br>2 | UNK | Unknown Race | | 181<br>203 | 7.4%<br>8.3% | 0.242 | 0.034 |
| 1<br>2 | 2076-8 | Native Hawaiian or Other Pacific Islander | | 10<br>11 | 0.4%<br>0.5% | 0.827 | 0.006 |
| 1<br>2 | UN | Unknown Ethnicity | | 470<br>470 | 19.3%<br>19.3% | 1 | <0.001 |
| 1<br>2 | 2186-5 | Not Hispanic or Latino | | 1,717<br>1,690 | 70.5%<br>69.3% | 0.399 | 0.024 |
| 1<br>2 | 2135-2 | Hispanic or Latino | | 250<br>277 | 10.3%<br>11.4% | 0.213 | 0.036 |
| 1<br>2 | 2131-1 | Other Race | | 128<br>133 | 5.3%<br>5.5% | 0.750 | 0.009 |
| 1<br>2 | 2028-9 | Asian | | 80<br>83 | 3.3%<br>3.4% | 0.811 | 0.007 |
| **Diagnosis** | | | | | | | |
| Cohort | | | Mean ± SD | Patients | % of Cohort | P-Value | Std diff. |
| 1<br>2 | Z55 | Problems related to education and literacy | | 10<br>10 | 0.4%<br>0.4% | 1 | <0.001 |
| 1<br>2 | Z56 | Problems related to employment and unemployment | | 10<br>12 | 0.4%<br>0.5% | 0.669 | 0.012 |
| 1<br>2 | Z81 | Family history of mental and behavioral disorders | | 10<br>10 | 0.4%<br>0.4% | 1 | <0.001 |
| 1<br>2 | E70-E88 | Metabolic disorders | | 573<br>602 | 23.5%<br>24.7% | 0.331 | 0.028 |
| 1<br>2 | F31 | Bipolar disorder | | 45<br>53 | 1.8%<br>2.2% | 0.414 | 0.023 |
| 1<br>2 | F32 | Depressive episode | | 269<br>286 | 11.0%<br>11.7% | 0.443 | 0.022 |
| 1<br>2 | F33 | Major depressive disorder, recurrent | | 66<br>78 | 2.7%<br>3.2% | 0.310 | 0.029 |
| 1<br>2 | X71 | Intentional self-harm by drowning and submersion | | 0<br>0 | 0%<br>0% | -- | -- |

| | | | | | | | |
|---|---|---|---|---|---|---|---|
| 1<br>2 | R45.851 | Suicidal ideations | | 31<br>34 | 1.3%<br>1.4% | 0.708 | 0.011 |
| 1<br>2 | T14.91 | Suicide attempt | | 10<br>0 | 0.4%<br>0% | 0.002 | 0.091 |
| 1<br>2 | X71-X83 | Intentional self-harm | | 10<br>10 | 0.4%<br>0.4% | 1 | <0.001 |
| 1<br>2 | W54.0 | Bitten by dog | | 10<br>10 | 0.4%<br>0.4% | 1 | <0.001 |
| 1<br>2 | L60.0 | Ingrowing nail | | 23<br>17 | 0.9%<br>0.7% | 0.341 | 0.027 |
| 1<br>2 | B07 | Viral warts | | 36<br>41 | 1.5%<br>1.7% | 0.566 | 0.016 |
| 1<br>2 | I00-I99 | Diseases of the circulatory system | | 882<br>879 | 36.2%<br>36.1% | 0.929 | 0.003 |
| 1<br>2 | F41 | Other anxiety disorders | | 335<br>368 | 13.7%<br>15.1% | 0.178 | 0.039 |
| 1<br>2 | F41.1 | Generalized anxiety disorder | | 98<br>113 | 4.0%<br>4.6% | 0.291 | 0.030 |
| 1<br>2 | F40 | Phobic anxiety disorders | | 17<br>21 | 0.7%<br>0.9% | 0.515 | 0.019 |
| 1<br>2 | F41.8 | Other specified anxiety disorders | | 38<br>38 | 1.6%<br>1.6% | 1 | <0.001 |
| 1<br>2 | F41.0 | Panic disorder [episodic paroxysmal anxiety] | | 18<br>30 | 0.7%<br>1.2% | 0.082 | 0.050 |
| 1<br>2 | F41.3 | Other mixed anxiety disorders | | 0<br>0 | 0%<br>0% | -- | -- |
| 1<br>2 | F41.9 | Anxiety disorder, unspecified | | 276<br>299 | 11.3%<br>12.3% | 0.307 | 0.029 |
| 1<br>2 | F42 | Obsessive-compulsive disorder | | 14<br>16 | 0.6%<br>0.7% | 0.714 | 0.010 |
| 1<br>2 | F31.2 | Bipolar disorder, current episode manic severe with psychotic features | | 0<br>0 | 0%<br>0% | -- | -- |
| 1<br>2 | F31.5 | Bipolar disorder, current episode depressed, severe, with psychotic features | | 0<br>0 | 0%<br>0% | -- | -- |
| 1<br>2 | F32.3 | Major depressive disorder, single episode, severe with psychotic features | | 0<br>10 | 0%<br>0.4% | 0.002 | 0.091 |
| 1<br>2 | F33.3 | Major depressive disorder, recurrent, severe with psychotic symptoms | | 10<br>10 | 0.4%<br>0.4% | 1 | <0.001 |
| 1<br>2 | F30.2 | Manic episode, severe with psychotic symptoms | | 0<br>0 | 0%<br>0% | -- | -- |
| **Medication** | | | | | | | |
| Cohort | | | Mean ± SD | Patients | % of Cohort | P-Value | Std diff. |
| 1<br>2 | R01AD | Corticosteroids | | 722<br>738 | 29.6%<br>30.3% | 0.617 | 0.014 |

## Supplementary Table S41. Baseline characteristics after propensity score matching low-dose methotrexate *vs* Leflunomide (males)

| Cohort 1. Low-dose methotrexate (N = 622) and cohort 2. Leflunomide (N = 622) | | | | | | | |
|---|---|---|---|---|---|---|---|
| **Demographics** | | | | | | | |
| Cohort | | | Mean ± SD | Patients | % of Cohort | P-Value | Std diff. |
| 1<br>2 | AI | Age at Index | 37.0 +/- 7.1<br>36.9 +/- 7.2 | 622<br>622 | 100%<br>100% | 0.874 | 0.009 |
| 1<br>2 | F | Female | | 0<br>0 | 0%<br>0% | -- | -- |
| 1<br>2 | 2054-5 | Black or African American | | 60<br>59 | 9.6%<br>9.5% | 0.923 | 0.005 |
| 1<br>2 | M | Male | | 622<br>622 | 100%<br>100% | -- | -- |
| 1<br>2 | 2106-3 | White | | 453<br>452 | 72.8%<br>72.7% | 0.949 | 0.004 |
| 1<br>2 | 1002-5 | American Indian or Alaska Native | | 10<br>10 | 1.6%<br>1.6% | 1 | <0.001 |
| 1<br>2 | UNK | Unknown Race | | 41<br>45 | 6.6%<br>7.2% | 0.655 | 0.025 |
| 1<br>2 | 2076-8 | Native Hawaiian or Other Pacific Islander | | 10<br>10 | 1.6%<br>1.6% | 1 | <0.001 |
| 1<br>2 | UN | Unknown Ethnicity | | 101<br>107 | 16.2%<br>17.2% | 0.648 | 0.026 |
| 1<br>2 | 2186-5 | Not Hispanic or Latino | | 433<br>429 | 69.6%<br>69.0% | 0.806 | 0.014 |
| 1<br>2 | 2135-2 | Hispanic or Latino | | 88<br>86 | 14.1%<br>13.8% | 0.870 | 0.009 |
| 1<br>2 | 2131-1 | Other Race | | 50<br>47 | 8.0%<br>7.6% | 0.751 | 0.018 |
| 1<br>2 | 2028-9 | Asian | | 13<br>12 | 2.1%<br>1.9% | 0.840 | 0.011 |
| **Diagnosis** | | | | | | | |
| Cohort | | | Mean ± SD | Patients | % of Cohort | P-Value | Std diff. |
| 1<br>2 | Z55 | Problems related to education and literacy | | 0<br>0 | 0%<br>0% | -- | -- |
| 1<br>2 | Z56 | Problems related to employment and unemployment | | 10<br>10 | 1.6%<br>1.6% | 1 | <0.001 |
| 1<br>2 | Z81 | Family history of mental and behavioral disorders | | 10<br>10 | 1.6%<br>1.6% | 1 | <0.001 |
| 1<br>2 | E70-E88 | Metabolic disorders | | 146<br>153 | 23.5%<br>24.6% | 0.642 | 0.026 |
| 1<br>2 | F31 | Bipolar disorder | | 10<br>10 | 1.6%<br>1.6% | 1 | <0.001 |
| 1<br>2 | F32 | Depressive episode | | 53<br>68 | 8.5%<br>10.9% | 0.151 | 0.081 |
| 1<br>2 | F33 | Major depressive disorder, recurrent | | 10<br>18 | 1.6%<br>2.9% | 0.126 | 0.087 |
| 1<br>2 | X71 | Intentional self-harm by drowning and submersion | | 0<br>0 | 0%<br>0% | -- | -- |

| | | | | | | | |
|---|---|---|---|---|---|---|---|
| 1<br>2 | R45.851 | Suicidal ideations | | 10<br>10 | 1.6%<br>1.6% | 1 | <0.001 |
| 1<br>2 | T14.91 | Suicide attempt | | 0<br>0 | 0%<br>0% | -- | -- |
| 1<br>2 | X71-X83 | Intentional self-harm | | 0<br>0 | 0%<br>0% | -- | -- |
| 1<br>2 | W54.0 | Bitten by dog | | 0<br>0 | 0%<br>0% | -- | -- |
| 1<br>2 | L60.0 | Ingrowing nail | | 10<br>10 | 1.6%<br>1.6% | 1 | <0.001 |
| 1<br>2 | B07 | Viral warts | | 12<br>10 | 1.9%<br>1.6% | 0.667 | 0.024 |
| 1<br>2 | I00-I99 | Diseases of the circulatory system | | 227<br>226 | 36.5%<br>36.3% | 0.953 | 0.003 |
| 1<br>2 | F41 | Other anxiety disorders | | 74<br>86 | 11.9%<br>13.8% | 0.309 | 0.058 |
| 1<br>2 | F41.1 | Generalized anxiety disorder | | 14<br>21 | 2.3%<br>3.4% | 0.230 | 0.068 |
| 1<br>2 | F40 | Phobic anxiety disorders | | 10<br>10 | 1.6%<br>1.6% | 1 | <0.001 |
| 1<br>2 | F41.8 | Other specified anxiety disorders | | 10<br>10 | 1.6%<br>1.6% | 1 | <0.001 |
| 1<br>2 | F41.0 | Panic disorder [episodic paroxysmal anxiety] | | 10<br>10 | 1.6%<br>1.6% | 1 | <0.001 |
| 1<br>2 | F41.3 | Other mixed anxiety disorders | | 0<br>0 | 0%<br>0% | -- | -- |
| 1<br>2 | F41.9 | Anxiety disorder, unspecified | | 58<br>65 | 9.3%<br>10.5% | 0.506 | 0.038 |
| 1<br>2 | F42 | Obsessive-compulsive disorder | | 10<br>10 | 1.6%<br>1.6% | 1 | <0.001 |
| 1<br>2 | F31.2 | Bipolar disorder, current episode manic severe with psychotic features | | 0<br>0 | 0%<br>0% | -- | -- |
| 1<br>2 | F31.5 | Bipolar disorder, current episode depressed, severe, with psychotic features | | 0<br>0 | 0%<br>0% | -- | -- |
| 1<br>2 | F32.3 | Major depressive disorder, single episode, severe with psychotic features | | 0<br>0 | 0%<br>0% | -- | -- |
| 1<br>2 | F33.3 | Major depressive disorder, recurrent, severe with psychotic symptoms | | 0<br>0 | 0%<br>0% | -- | -- |
| 1<br>2 | F30.2 | Manic episode, severe with psychotic symptoms | | 0<br>0 | 0%<br>0% | -- | -- |
| **Medication** | | | | | | | |
| Cohort | | | Mean ± SD | Patients | % of Cohort | P-Value | Std diff. |
| 1<br>2 | R01AD | Corticosteroids | | 188<br>185 | 30.2%<br>29.7% | 0.853 | 0.011 |

## Supplementary Table S42. Baseline characteristics after propensity score matching low-dose methotrexate *vs* Sulfasalazine (males)

| Cohort 1. Low-dose methotrexate (N = 1,268) and cohort 2. Sulfasalazine (N = 1,268) | | | | | | | |
|---|---|---|---|---|---|---|---|
| **Demographics** | | | | | | | |
| Cohort | | | Mean ± SD | Patients | % of Cohort | P-Value | Std diff. |
| 1<br>2 | AI | Age at Index | 35.7 +/- 8.2<br>35.5 +/- 7.9 | 1,268<br>1,268 | 100%<br>100% | 0.664 | 0.017 |
| 1<br>2 | F | Female | | 0<br>0 | 0%<br>0% | -- | -- |
| 1<br>2 | 2054-5 | Black or African American | | 91<br>94 | 7.2%<br>7.4% | 0.819 | 0.009 |
| 1<br>2 | M | Male | | 1,268<br>1,268 | 100%<br>100% | -- | -- |
| 1<br>2 | 2106-3 | White | | 963<br>950 | 75.9%<br>74.9% | 0.549 | 0.024 |
| 1<br>2 | 1002-5 | American Indian or Alaska Native | | 14<br>12 | 1.1%<br>0.9% | 0.693 | 0.016 |
| 1<br>2 | UNK | Unknown Race | | 94<br>105 | 7.4%<br>8.3% | 0.417 | 0.032 |
| 1<br>2 | 2076-8 | Native Hawaiian or Other Pacific Islander | | 10<br>10 | 0.8%<br>0.8% | 1 | <0.001 |
| 1<br>2 | UN | Unknown Ethnicity | | 303<br>283 | 23.9%<br>22.3% | 0.346 | 0.037 |
| 1<br>2 | 2186-5 | Not Hispanic or Latino | | 816<br>827 | 64.4%<br>65.2% | 0.647 | 0.018 |
| 1<br>2 | 2135-2 | Hispanic or Latino | | 149<br>158 | 11.8%<br>12.5% | 0.584 | 0.022 |
| 1<br>2 | 2131-1 | Other Race | | 57<br>54 | 4.5%<br>4.3% | 0.771 | 0.012 |
| 1<br>2 | 2028-9 | Asian | | 43<br>47 | 3.4%<br>3.7% | 0.668 | 0.017 |
| **Diagnosis** | | | | | | | |
| Cohort | | | Mean ± SD | Patients | % of Cohort | P-Value | Std diff. |
| 1<br>2 | Z55 | Problems related to education and literacy | | 0<br>10 | 0%<br>0.8% | 0.002 | 0.126 |
| 1<br>2 | Z56 | Problems related to employment and unemployment | | 10<br>10 | 0.8%<br>0.8% | 1 | <0.001 |
| 1<br>2 | Z81 | Family history of mental and behavioral disorders | | 10<br>10 | 0.8%<br>0.8% | 1 | <0.001 |
| 1<br>2 | E70-E88 | Metabolic disorders | | 296<br>299 | 23.3%<br>23.6% | 0.888 | 0.006 |
| 1<br>2 | F31 | Bipolar disorder | | 19<br>25 | 1.5%<br>2.0% | 0.362 | 0.036 |
| 1<br>2 | F32 | Depressive episode | | 126<br>145 | 9.9%<br>11.4% | 0.222 | 0.049 |
| 1<br>2 | F33 | Major depressive disorder, recurrent | | 24<br>32 | 1.9%<br>2.5% | 0.280 | 0.043 |
| 1<br>2 | X71 | Intentional self-harm by drowning and submersion | | 0<br>0 | 0%<br>0% | -- | -- |

| | | | | | | | |
|---|---|---|---|---|---|---|---|
| 1<br>2 | R45.851 | Suicidal ideations | | 10<br>13 | 0.8%<br>1.0% | 0.530 | 0.025 |
| 1<br>2 | T14.91 | Suicide attempt | | 0<br>0 | 0%<br>0% | -- | -- |
| 1<br>2 | X71-X83 | Intentional self-harm | | 10<br>10 | 0.8%<br>0.8% | 1 | <0.001 |
| 1<br>2 | W54.0 | Bitten by dog | | 10<br>10 | 0.8%<br>0.8% | 1 | <0.001 |
| 1<br>2 | L60.0 | Ingrowing nail | | 10<br>12 | 0.8%<br>0.9% | 0.668 | 0.017 |
| 1<br>2 | B07 | Viral warts | | 23<br>27 | 1.8%<br>2.1% | 0.568 | 0.023 |
| 1<br>2 | I00-I99 | Diseases of the circulatory system | | 359<br>372 | 28.3%<br>29.3% | 0.569 | 0.023 |
| 1<br>2 | F41 | Other anxiety disorders | | 171<br>199 | 13.5%<br>15.7% | 0.115 | 0.063 |
| 1<br>2 | F41.1 | Generalized anxiety disorder | | 60<br>61 | 4.7%<br>4.8% | 0.926 | 0.004 |
| 1<br>2 | F40 | Phobic anxiety disorders | | 16<br>11 | 1.3%<br>0.9% | 0.333 | 0.038 |
| 1<br>2 | F41.8 | Other specified anxiety disorders | | 26<br>24 | 2.1%<br>1.9% | 0.775 | 0.011 |
| 1<br>2 | F41.0 | Panic disorder [episodic paroxysmal anxiety] | | 14<br>17 | 1.1%<br>1.3% | 0.588 | 0.022 |
| 1<br>2 | F41.3 | Other mixed anxiety disorders | | 0<br>0 | 0%<br>0% | -- | -- |
| 1<br>2 | F41.9 | Anxiety disorder, unspecified | | 140<br>161 | 11.0%<br>12.7% | 0.197 | 0.051 |
| 1<br>2 | F42 | Obsessive-compulsive disorder | | 10<br>10 | 0.8%<br>0.8% | 1 | <0.001 |
| 1<br>2 | F31.2 | Bipolar disorder, current episode manic severe with psychotic features | | 0<br>0 | 0%<br>0% | -- | -- |
| 1<br>2 | F31.5 | Bipolar disorder, current episode depressed, severe, with psychotic features | | 0<br>0 | 0%<br>0% | -- | -- |
| 1<br>2 | F32.3 | Major depressive disorder, single episode, severe with psychotic features | | 0<br>10 | 0%<br>0.8% | 0.002 | 0.126 |
| 1<br>2 | F33.3 | Major depressive disorder, recurrent, severe with psychotic symptoms | | 0<br>10 | 0%<br>0.8% | 0.002 | 0.126 |
| 1<br>2 | F30.2 | Manic episode, severe with psychotic symptoms | | 0<br>0 | 0%<br>0% | -- | -- |
| **Medication** | | | | | | | |
| Cohort | | | Mean ± SD | Patients | % of Cohort | P-Value | Std diff. |
| 1<br>2 | R01AD | Corticosteroids | | 379<br>413 | 29.9%<br>32.6% | 0.145 | 0.058 |

## Supplementary Table S43. Baseline characteristics after propensity score matching low-dose methotrexate *vs* Minocycline (males)

| Cohort 1. Low-dose methotrexate (N = 207) and cohort 2. Minocycline (N = 207) | | | | | | | |
|---|---|---|---|---|---|---|---|
| **Demographics** | | | | | | | |
| Cohort | | | Mean ± SD | Patients | % of Cohort | P-Value | Std diff. |
| 1<br>2 | AI | Age at Index | 32.5 +/- 10.0<br>33.3 +/- 9.3 | 207<br>207 | 100%<br>100% | 0.388 | 0.085 |
| 1<br>2 | F | Female | | 0<br>0 | 0%<br>0% | -- | -- |
| 1<br>2 | 2054-5 | Black or African American | | 32<br>32 | 15.5%<br>15.5% | 1 | <0.001 |
| 1<br>2 | M | Male | | 207<br>207 | 100%<br>100% | -- | -- |
| 1<br>2 | 2106-3 | White | | 136<br>136 | 65.7%<br>65.7% | 1 | <0.001 |
| 1<br>2 | 1002-5 | American Indian or Alaska Native | | 10<br>10 | 4.8%<br>4.8% | 1 | <0.001 |
| 1<br>2 | UNK | Unknown Race | | 10<br>12 | 4.8%<br>5.8% | 0.661 | 0.043 |
| 1<br>2 | 2076-8 | Native Hawaiian or Other Pacific Islander | | 10<br>10 | 4.8%<br>4.8% | 1 | <0.001 |
| 1<br>2 | UN | Unknown Ethnicity | | 38<br>31 | 18.4%<br>15.0% | 0.356 | 0.091 |
| 1<br>2 | 2186-5 | Not Hispanic or Latino | | 158<br>153 | 76.3%<br>73.9% | 0.570 | 0.056 |
| 1<br>2 | 2135-2 | Hispanic or Latino | | 11<br>23 | 5.3%<br>11.1% | 0.032 | 0.212 |
| 1<br>2 | 2131-1 | Other Race | | 10<br>12 | 4.8%<br>5.8% | 0.661 | 0.043 |
| 1<br>2 | 2028-9 | Asian | | 13<br>10 | 6.3%<br>4.8% | 0.520 | 0.063 |
| **Diagnosis** | | | | | | | |
| Cohort | | | Mean ± SD | Patients | % of Cohort | P-Value | Std diff. |
| 1<br>2 | Z55 | Problems related to education and literacy | | 10<br>10 | 4.8%<br>4.8% | 1 | <0.001 |
| 1<br>2 | Z56 | Problems related to employment and unemployment | | 10<br>10 | 4.8%<br>4.8% | 1 | <0.001 |
| 1<br>2 | Z81 | Family history of mental and behavioral disorders | | 10<br>10 | 4.8%<br>4.8% | 1 | <0.001 |
| 1<br>2 | E70-E88 | Metabolic disorders | | 64<br>76 | 30.9%<br>36.7% | 0.213 | 0.123 |
| 1<br>2 | F31 | Bipolar disorder | | 10<br>10 | 4.8%<br>4.8% | 1 | <0.001 |
| 1<br>2 | F32 | Depressive episode | | 35<br>35 | 16.9%<br>16.9% | 1 | <0.001 |
| 1<br>2 | F33 | Major depressive disorder, recurrent | | 10<br>10 | 4.8%<br>4.8% | 1 | <0.001 |
| 1<br>2 | X71 | Intentional self-harm by drowning and submersion | | 0<br>0 | 0%<br>0% | -- | -- |

| Cohort | Code | Description | Patients | % of Cohort | P-Value | Std diff. |
|---|---|---|---|---|---|---|
| 1<br>2 | R45.851 | Suicidal ideations | 10<br>10 | 4.8%<br>4.8% | 1 | <0.001 |
| 1<br>2 | T14.91 | Suicide attempt | 0<br>0 | 0%<br>0% | -- | -- |
| 1<br>2 | X71-X83 | Intentional self-harm | 0<br>0 | 0%<br>0% | -- | -- |
| 1<br>2 | W54.0 | Bitten by dog | 10<br>0 | 4.8%<br>0% | 0.001 | 0.319 |
| 1<br>2 | L60.0 | Ingrowing nail | 10<br>10 | 4.8%<br>4.8% | 1 | <0.001 |
| 1<br>2 | B07 | Viral warts | 10<br>10 | 4.8%<br>4.8% | 1 | <0.001 |
| 1<br>2 | I00-I99 | Diseases of the circulatory system | 102<br>104 | 49.3%<br>50.2% | 0.844 | 0.019 |
| 1<br>2 | F41 | Other anxiety disorders | 31<br>38 | 15.0%<br>18.4% | 0.356 | 0.091 |
| 1<br>2 | F41.1 | Generalized anxiety disorder | 10<br>12 | 4.8%<br>5.8% | 0.661 | 0.043 |
| 1<br>2 | F40 | Phobic anxiety disorders | 10<br>10 | 4.8%<br>4.8% | 1 | <0.001 |
| 1<br>2 | F41.8 | Other specified anxiety disorders | 10<br>10 | 4.8%<br>4.8% | 1 | <0.001 |
| 1<br>2 | F41.0 | Panic disorder [episodic paroxysmal anxiety] | 10<br>10 | 4.8%<br>4.8% | 1 | <0.001 |
| 1<br>2 | F41.3 | Other mixed anxiety disorders | 0<br>0 | 0%<br>0% | -- | -- |
| 1<br>2 | F41.9 | Anxiety disorder, unspecified | 23<br>29 | 11.1%<br>14.0% | 0.374 | 0.088 |
| 1<br>2 | F42 | Obsessive-compulsive disorder | 10<br>10 | 4.8%<br>4.8% | 1 | <0.001 |
| 1<br>2 | F31.2 | Bipolar disorder, current episode manic severe with psychotic features | 0<br>0 | 0%<br>0% | -- | -- |
| 1<br>2 | F31.5 | Bipolar disorder, current episode depressed, severe, with psychotic features | 0<br>0 | 0%<br>0% | -- | -- |
| 1<br>2 | F32.3 | Major depressive disorder, single episode, severe with psychotic features | 0<br>0 | 0%<br>0% | -- | -- |
| 1<br>2 | F33.3 | Major depressive disorder, recurrent, severe with psychotic symptoms | 0<br>0 | 0%<br>0% | -- | -- |
| 1<br>2 | F30.2 | Manic episode, severe with psychotic symptoms | 0<br>0 | 0%<br>0% | -- | -- |
| **Medication** | | | | | | |
| Cohort | | Mean ± SD | Patients | % of Cohort | P-Value | Std diff. |
| 1<br>2 | R01AD | Corticosteroids | 86<br>94 | 41.5%<br>45.4% | 0.428 | 0.078 |

## Supplementary Table S44. Baseline characteristics after propensity score matching low-dose methotrexate *vs* Abatacept (males)

| Cohort 1. Low-dose methotrexate (N = 243) and cohort 2. Abatacept (N = 243) | | | | | | | |
|---|---|---|---|---|---|---|---|
| **Demographics** | | | | | | | |
| Cohort | | | Mean ± SD | Patients | % of Cohort | P-Value | Std diff. |
| 1<br>2 | AI | Age at Index | 36.5 +/- 7.7<br>36.2 +/- 8.1 | 243<br>243 | 100%<br>100% | 0.693 | 0.036 |
| 1<br>2 | F | Female | | 0<br>0 | 0%<br>0% | -- | -- |
| 1<br>2 | 2054-5 | Black or African American | | 23<br>22 | 9.5%<br>9.1% | 0.876 | 0.014 |
| 1<br>2 | M | Male | | 243<br>243 | 100%<br>100% | -- | -- |
| 1<br>2 | 2106-3 | White | | 196<br>196 | 80.7%<br>80.7% | 1 | <0.001 |
| 1<br>2 | 1002-5 | American Indian or Alaska Native | | 10<br>10 | 4.1%<br>4.1% | 1 | <0.001 |
| 1<br>2 | UNK | Unknown Race | | 14<br>14 | 5.8%<br>5.8% | 1 | <0.001 |
| 1<br>2 | 2076-8 | Native Hawaiian or Other Pacific Islander | | 0<br>0 | 0%<br>0% | -- | -- |
| 1<br>2 | UN | Unknown Ethnicity | | 37<br>44 | 15.2%<br>18.1% | 0.394 | 0.077 |
| 1<br>2 | 2186-5 | Not Hispanic or Latino | | 183<br>170 | 75.3%<br>70.0% | 0.186 | 0.120 |
| 1<br>2 | 2135-2 | Hispanic or Latino | | 23<br>29 | 9.5%<br>11.9% | 0.379 | 0.080 |
| 1<br>2 | 2131-1 | Other Race | | 10<br>10 | 4.1%<br>4.1% | 1 | <0.001 |
| 1<br>2 | 2028-9 | Asian | | 10<br>10 | 4.1%<br>4.1% | 1 | <0.001 |
| **Diagnosis** | | | | | | | |
| Cohort | | | Mean ± SD | Patients | % of Cohort | P-Value | Std diff. |
| 1<br>2 | Z55 | Problems related to education and literacy | | 0<br>0 | 0%<br>0% | -- | -- |
| 1<br>2 | Z56 | Problems related to employment and unemployment | | 10<br>10 | 4.1%<br>4.1% | 1 | <0.001 |
| 1<br>2 | Z81 | Family history of mental and behavioral disorders | | 0<br>0 | 0%<br>0% | -- | -- |
| 1<br>2 | E70-E88 | Metabolic disorders | | 71<br>70 | 29.2%<br>28.8% | 0.920 | 0.009 |
| 1<br>2 | F31 | Bipolar disorder | | 13<br>11 | 5.3%<br>4.5% | 0.675 | 0.038 |
| 1<br>2 | F32 | Depressive episode | | 24<br>25 | 9.9%<br>10.3% | 0.880 | 0.014 |
| 1<br>2 | F33 | Major depressive disorder, recurrent | | 13<br>10 | 5.3%<br>4.1% | 0.522 | 0.058 |
| 1<br>2 | X71 | Intentional self-harm by drowning and submersion | | 0<br>0 | 0%<br>0% | -- | -- |

| | | | | | | | |
|---|---|---|---|---|---|---|---|
| 1<br>2 | R45.851 | Suicidal ideations | | 10<br>10 | 4.1%<br>4.1% | 1 | <0.001 |
| 1<br>2 | T14.91 | Suicide attempt | | 0<br>0 | 0%<br>0% | -- | -- |
| 1<br>2 | X71-X83 | Intentional self-harm | | 0<br>0 | 0%<br>0% | -- | -- |
| 1<br>2 | W54.0 | Bitten by dog | | 0<br>0 | 0%<br>0% | -- | -- |
| 1<br>2 | L60.0 | Ingrowing nail | | 10<br>0 | 4.1%<br>0% | 0.001 | 0.293 |
| 1<br>2 | B07 | Viral warts | | 10<br>10 | 4.1%<br>4.1% | 1 | <0.001 |
| 1<br>2 | I00-I99 | Diseases of the circulatory system | | 97<br>96 | 39.9%<br>39.5% | 0.926 | 0.008 |
| 1<br>2 | F41 | Other anxiety disorders | | 43<br>45 | 17.7%<br>18.5% | 0.814 | 0.021 |
| 1<br>2 | F41.1 | Generalized anxiety disorder | | 12<br>14 | 4.9%<br>5.8% | 0.687 | 0.037 |
| 1<br>2 | F40 | Phobic anxiety disorders | | 10<br>10 | 4.1%<br>4.1% | 1 | <0.001 |
| 1<br>2 | F41.8 | Other specified anxiety disorders | | 10<br>10 | 4.1%<br>4.1% | 1 | <0.001 |
| 1<br>2 | F41.0 | Panic disorder [episodic paroxysmal anxiety] | | 10<br>10 | 4.1%<br>4.1% | 1 | <0.001 |
| 1<br>2 | F41.3 | Other mixed anxiety disorders | | 0<br>0 | 0%<br>0% | -- | -- |
| 1<br>2 | F41.9 | Anxiety disorder, unspecified | | 34<br>35 | 14.0%<br>14.4% | 0.897 | 0.012 |
| 1<br>2 | F42 | Obsessive-compulsive disorder | | 10<br>10 | 4.1%<br>4.1% | 1 | <0.001 |
| 1<br>2 | F31.2 | Bipolar disorder, current episode manic severe with psychotic features | | 0<br>0 | 0%<br>0% | -- | -- |
| 1<br>2 | F31.5 | Bipolar disorder, current episode depressed, severe, with psychotic features | | 0<br>0 | 0%<br>0% | -- | -- |
| 1<br>2 | F32.3 | Major depressive disorder, single episode, severe with psychotic features | | 0<br>0 | 0%<br>0% | -- | -- |
| 1<br>2 | F33.3 | Major depressive disorder, recurrent, severe with psychotic symptoms | | 0<br>0 | 0%<br>0% | -- | -- |
| 1<br>2 | F30.2 | Manic episode, severe with psychotic symptoms | | 0<br>0 | 0%<br>0% | -- | -- |
| **Medication** | | | | | | | |
| Cohort | | | Mean ± SD | Patients | % of Cohort | P-Value | Std diff. |
| 1<br>2 | R01AD | Corticosteroids | | 85<br>75 | 35.0%<br>30.9% | 0.334 | 0.088 |

**Supplementary Table S45. Baseline characteristics after propensity score matching low-dose methotrexate *vs* Tofacitinib (males)**

| Cohort 1. Low-dose methotrexate (N = 343) and cohort 2. Tofacitinib (N = 343) | | | | | | | |
|---|---|---|---|---|---|---|---|
| **Demographics** | | | | | | | |
| Cohort | | | Mean ± SD | Patients | % of Cohort | P-Value | Std diff. |
| 1<br>2 | AI | Age at Index | 35.6 +/- 8.1<br>35.2 +/- 8.4 | 343<br>343 | 100%<br>100% | 0.618 | 0.038 |
| 1<br>2 | F | Female | | 0<br>0 | 0%<br>0% | -- | -- |
| 1<br>2 | 2054-5 | Black or African American | | 28<br>28 | 8.2%<br>8.2% | 1 | <0.001 |
| 1<br>2 | M | Male | | 343<br>343 | 100%<br>100% | -- | -- |
| 1<br>2 | 2106-3 | White | | 272<br>268 | 79.3%<br>78.1% | 0.709 | 0.028 |
| 1<br>2 | 1002-5 | American Indian or Alaska Native | | 10<br>10 | 2.9%<br>2.9% | 1 | <0.001 |
| 1<br>2 | UNK | Unknown Race | | 19<br>20 | 5.5%<br>5.8% | 0.869 | 0.013 |
| 1<br>2 | 2076-8 | Native Hawaiian or Other Pacific Islander | | 0<br>0 | 0%<br>0% | -- | -- |
| 1<br>2 | UN | Unknown Ethnicity | | 55<br>53 | 16.0%<br>15.5% | 0.834 | 0.016 |
| 1<br>2 | 2186-5 | Not Hispanic or Latino | | 267<br>262 | 77.8%<br>76.4% | 0.650 | 0.035 |
| 1<br>2 | 2135-2 | Hispanic or Latino | | 21<br>28 | 6.1%<br>8.2% | 0.299 | 0.079 |
| 1<br>2 | 2131-1 | Other Race | | 13<br>16 | 3.8%<br>4.7% | 0.569 | 0.043 |
| 1<br>2 | 2028-9 | Asian | | 10<br>10 | 2.9%<br>2.9% | 1 | <0.001 |
| **Diagnosis** | | | | | | | |
| Cohort | | | Mean ± SD | Patients | % of Cohort | P-Value | Std diff. |
| 1<br>2 | Z55 | Problems related to education and literacy | | 0<br>0 | 0%<br>0% | -- | -- |
| 1<br>2 | Z56 | Problems related to employment and unemployment | | 0<br>10 | 0%<br>2.9% | 0.001 | 0.245 |
| 1<br>2 | Z81 | Family history of mental and behavioral disorders | | 0<br>10 | 0%<br>2.9% | 0.001 | 0.245 |
| 1<br>2 | E70-E88 | Metabolic disorders | | 77<br>82 | 22.4%<br>23.9% | 0.651 | 0.035 |
| 1<br>2 | F31 | Bipolar disorder | | 10<br>12 | 2.9%<br>3.5% | 0.665 | 0.033 |
| 1<br>2 | F32 | Depressive episode | | 43<br>49 | 12.5%<br>14.3% | 0.501 | 0.051 |
| 1<br>2 | F33 | Major depressive disorder, recurrent | | 10<br>10 | 2.9%<br>2.9% | 1 | <0.001 |
| 1<br>2 | X71 | Intentional self-harm by drowning and submersion | | 0<br>0 | 0%<br>0% | -- | -- |

| | | | | | | | |
|---|---|---|---|---|---|---|---|
| 1<br>2 | R45.851 | Suicidal ideations | | 10<br>10 | 2.9%<br>2.9% | 1 | <0.001 |
| 1<br>2 | T14.91 | Suicide attempt | | 0<br>0 | 0%<br>0% | -- | -- |
| 1<br>2 | X71-X83 | Intentional self-harm | | 0<br>0 | 0%<br>0% | -- | -- |
| 1<br>2 | W54.0 | Bitten by dog | | 0<br>10 | 0%<br>2.9% | 0.001 | 0.245 |
| 1<br>2 | L60.0 | Ingrowing nail | | 10<br>10 | 2.9%<br>2.9% | 1 | <0.001 |
| 1<br>2 | B07 | Viral warts | | 10<br>10 | 2.9%<br>2.9% | 1 | <0.001 |
| 1<br>2 | I00-I99 | Diseases of the circulatory system | | 119<br>124 | 34.7%<br>36.2% | 0.690 | 0.030 |
| 1<br>2 | F41 | Other anxiety disorders | | 56<br>63 | 16.3%<br>18.4% | 0.480 | 0.054 |
| 1<br>2 | F41.1 | Generalized anxiety disorder | | 10<br>16 | 2.9%<br>4.7% | 0.230 | 0.092 |
| 1<br>2 | F40 | Phobic anxiety disorders | | 10<br>10 | 2.9%<br>2.9% | 1 | <0.001 |
| 1<br>2 | F41.8 | Other specified anxiety disorders | | 10<br>10 | 2.9%<br>2.9% | 1 | <0.001 |
| 1<br>2 | F41.0 | Panic disorder [episodic paroxysmal anxiety] | | 10<br>10 | 2.9%<br>2.9% | 1 | <0.001 |
| 1<br>2 | F41.3 | Other mixed anxiety disorders | | 0<br>0 | 0%<br>0% | -- | -- |
| 1<br>2 | F41.9 | Anxiety disorder, unspecified | | 52<br>56 | 15.2%<br>16.3% | 0.675 | 0.032 |
| 1<br>2 | F42 | Obsessive-compulsive disorder | | 0<br>10 | 0%<br>2.9% | 0.001 | 0.245 |
| 1<br>2 | F31.2 | Bipolar disorder, current episode manic severe with psychotic features | | 0<br>0 | 0%<br>0% | -- | -- |
| 1<br>2 | F31.5 | Bipolar disorder, current episode depressed, severe, with psychotic features | | 0<br>0 | 0%<br>0% | -- | -- |
| 1<br>2 | F32.3 | Major depressive disorder, single episode, severe with psychotic features | | 0<br>0 | 0%<br>0% | -- | -- |
| 1<br>2 | F33.3 | Major depressive disorder, recurrent, severe with psychotic symptoms | | 0<br>10 | 0%<br>2.9% | 0.001 | 0.245 |
| 1<br>2 | F30.2 | Manic episode, severe with psychotic symptoms | | 0<br>0 | 0%<br>0% | -- | -- |
| **Medication** | | | | | | | |
| Cohort | | | Mean ± SD | Patients | % of Cohort | P-Value | Std diff. |
| 1<br>2 | R01AD | Corticosteroids | | 127<br>130 | 37.0%<br>37.9% | 0.813 | 0.018 |

**Supplementary Table S46. Baseline characteristics after propensity score matching low-dose methotrexate *vs* Upadacitinib (males)**

| Cohort 1. Low-dose methotrexate (N = 233) and cohort 2. Upadacitinib (N = 233) | | | | | | | |
|---|---|---|---|---|---|---|---|
| **Demographics** | | | | | | | |
| Cohort | | | Mean ± SD | Patients | % of Cohort | P-Value | Std diff. |
| 1<br>2 | AI | Age at Index | 36.4 +/- 7.2<br>36.1 +/- 7.3 | 233<br>233 | 100%<br>100% | 0.641 | 0.043 |
| 1<br>2 | F | Female | | 0<br>0 | 0%<br>0% | -- | -- |
| 1<br>2 | 2054-5 | Black or African American | | 13<br>12 | 5.6%<br>5.2% | 0.837 | 0.019 |
| 1<br>2 | M | Male | | 233<br>233 | 100%<br>100% | -- | -- |
| 1<br>2 | 2106-3 | White | | 185<br>187 | 79.4%<br>80.3% | 0.817 | 0.021 |
| 1<br>2 | 1002-5 | American Indian or Alaska Native | | 10<br>10 | 4.3%<br>4.3% | 1 | <0.001 |
| 1<br>2 | UNK | Unknown Race | | 16<br>14 | 6.9%<br>6.0% | 0.706 | 0.035 |
| 1<br>2 | 2076-8 | Native Hawaiian or Other Pacific Islander | | 10<br>10 | 4.3%<br>4.3% | 1 | <0.001 |
| 1<br>2 | UN | Unknown Ethnicity | | 44<br>43 | 18.9%<br>18.5% | 0.905 | 0.011 |
| 1<br>2 | 2186-5 | Not Hispanic or Latino | | 161<br>166 | 69.1%<br>71.2% | 0.613 | 0.047 |
| 1<br>2 | 2135-2 | Hispanic or Latino | | 28<br>24 | 12.0%<br>10.3% | 0.556 | 0.055 |
| 1<br>2 | 2131-1 | Other Race | | 10<br>10 | 4.3%<br>4.3% | 1 | <0.001 |
| 1<br>2 | 2028-9 | Asian | | 10<br>10 | 4.3%<br>4.3% | 1 | <0.001 |
| **Diagnosis** | | | | | | | |
| Cohort | | | Mean ± SD | Patients | % of Cohort | P-Value | Std diff. |
| 1<br>2 | Z55 | Problems related to education and literacy | | 0<br>0 | 0%<br>0% | -- | -- |
| 1<br>2 | Z56 | Problems related to employment and unemployment | | 10<br>10 | 4.3%<br>4.3% | 1 | <0.001 |
| 1<br>2 | Z81 | Family history of mental and behavioral disorders | | 10<br>10 | 4.3%<br>4.3% | 1 | <0.001 |
| 1<br>2 | E70-E88 | Metabolic disorders | | 78<br>79 | 33.5%<br>33.9% | 0.922 | 0.009 |
| 1<br>2 | F31 | Bipolar disorder | | 10<br>10 | 4.3%<br>4.3% | 1 | <0.001 |
| 1<br>2 | F32 | Depressive episode | | 49<br>46 | 21.0%<br>19.7% | 0.730 | 0.032 |
| 1<br>2 | F33 | Major depressive disorder, recurrent | | 18<br>17 | 7.7%<br>7.3% | 0.860 | 0.016 |
| 1<br>2 | X71 | Intentional self-harm by drowning and submersion | | 0<br>0 | 0%<br>0% | -- | -- |

| | | | | | | | |
|---|---|---|---|---|---|---|---|
| 1<br>2 | R45.851 | Suicidal ideations | | 10<br>10 | 4.3%<br>4.3% | 1 | <0.001 |
| 1<br>2 | T14.91 | Suicide attempt | | 0<br>0 | 0%<br>0% | -- | -- |
| 1<br>2 | X71-X83 | Intentional self-harm | | 10<br>0 | 4.3%<br>0% | 0.001 | 0.299 |
| 1<br>2 | W54.0 | Bitten by dog | | 10<br>10 | 4.3%<br>4.3% | 1 | <0.001 |
| 1<br>2 | L60.0 | Ingrowing nail | | 10<br>10 | 4.3%<br>4.3% | 1 | <0.001 |
| 1<br>2 | B07 | Viral warts | | 10<br>10 | 4.3%<br>4.3% | 1 | <0.001 |
| 1<br>2 | I00-I99 | Diseases of the circulatory system | | 94<br>84 | 40.3%<br>36.1% | 0.340 | 0.088 |
| 1<br>2 | F41 | Other anxiety disorders | | 48<br>52 | 20.6%<br>22.3% | 0.652 | 0.042 |
| 1<br>2 | F41.1 | Generalized anxiety disorder | | 15<br>19 | 6.4%<br>8.2% | 0.476 | 0.066 |
| 1<br>2 | F40 | Phobic anxiety disorders | | 10<br>10 | 4.3%<br>4.3% | 1 | <0.001 |
| 1<br>2 | F41.8 | Other specified anxiety disorders | | 10<br>10 | 4.3%<br>4.3% | 1 | <0.001 |
| 1<br>2 | F41.0 | Panic disorder [episodic paroxysmal anxiety] | | 10<br>10 | 4.3%<br>4.3% | 1 | <0.001 |
| 1<br>2 | F41.3 | Other mixed anxiety disorders | | 0<br>0 | 0%<br>0% | -- | -- |
| 1<br>2 | F41.9 | Anxiety disorder, unspecified | | 41<br>42 | 17.6%<br>18.0% | 0.904 | 0.011 |
| 1<br>2 | F42 | Obsessive-compulsive disorder | | 10<br>10 | 4.3%<br>4.3% | 1 | <0.001 |
| **Medication** | | | | | | | |
| Cohort | | | Mean ± SD | Patients | % of Cohort | P-Value | Std diff. |
| 1<br>2 | R01AD | Corticosteroids | | 103<br>102 | 44.2%<br>43.8% | 0.926 | 0.009 |

# Supplementary Figure 3

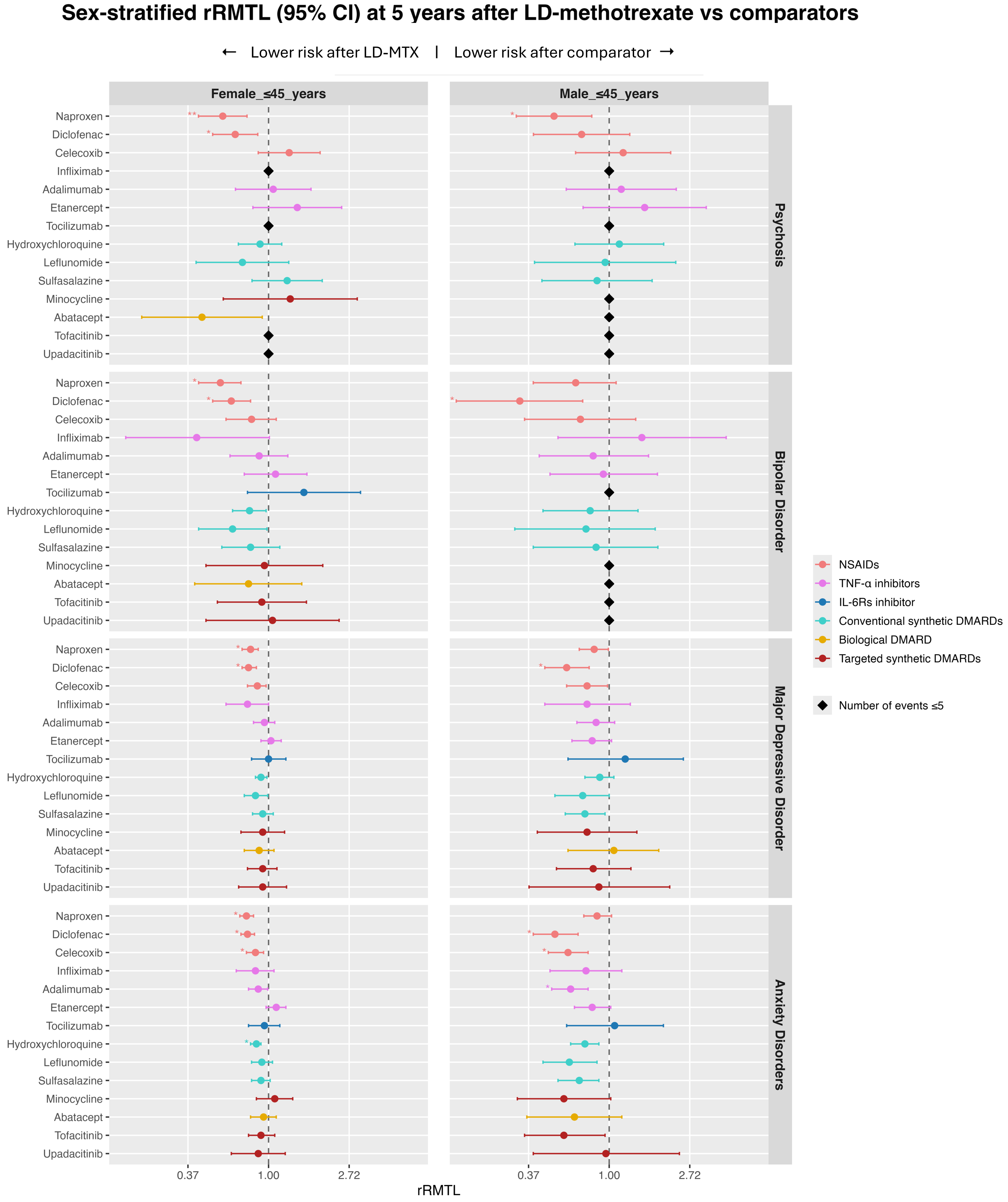


**Supplementary Figure 3. Sex-stratified restricted mean time lost ratios (rRMTL) at 5 years after initiation of low-dose methotrexate (LD-methotrexate) *vs* comparator drugs.** Values are shown on the log scale with 95% confidence intervals (CIs); axis labels are back-transformed to rRMTL for interpretability. An rRMTL >1 indicates that people were diagnosed with psychosis more frequently or sooner after the comparator drug than after LD-MTX, and conversely for rRMTL <1. The vertical dashed line represents no difference (rRMTL = 1). Colours indicate drug classes. Black diamonds denote drug–subgroup combinations with ≤5 events in either treatment group; * = P less than Bonferroni corrected critical value for multiple comparison: α = 0.05/8 = 0.0083 for the primary analyses (3 non-steroidal anti-inflammatory drugs), and α = 0.05/44 = 0.00056 for secondary analyses (4 outcomes × 11 drugs). Cohort size after propensity score matching: Naproxen (female N=9,719; male N=2,604 ), Diclofenac (female N=10,596; male N=1,013), Celecoxib (female N=7,013; male N=1,601), Infliximab (female N=829; male N=301), Adalimumab (female N=5,230; male N=1,606), Etanercept (female N=4,931; male N=1,188), Tocilizumab (female N=1,316; male N=227), Hydroxychloroquine (female N=17,318; male N=2,437), Leflunomide (female N=3,298; male N=622), Sulfasalazine (female N=5,439; male N=1,268), Minocycline (female N=880; male N=207), Abatacept (female N=1,960; male N=243), Tofacitinib (female N=1,797; male N=343), Upadacitinib (female N=1,169; male N=233). LD-MTX, low-dose methotrexate; rRMTL, restricted mean time lost ratio; NSAIDs, non-steroidal anti-inflammatory drugs; DMARDs, disease modifying antirheumatic drugs.

**Supplementary Table S47A. Summary of restricted mean time lost ratio (rRMTL, 95% CI) for the risk of psychosis, bipolar disorder, major depressive disorder, and anxiety disorders at five years follow-up (low-dose methotrexate vs comparators)**

| Comparator | rRMTL | rRMTL (CI low) | rRMTL (CI high) | p_value | Events low-dose methotrexate | Events Comparator |
|---|---|---|---|---|---|---|
| **Psychosis** | | | | | | |
| **Naproxen** | **0.69** | **0.55** | **0.87** | **0.00185** | **130** | **191** |
| **Diclofenac** | **0.71** | **0.56** | **0.90** | **0.00429** | **131** | **178** |
| Celecoxib | 1.23 | 0.86 | 1.74 | 0.253 | 80 | 57 |
| Infliximab | 0.54 | 0.27 | 1.09 | 0.0845 | 12 | 23 |
| Adalimumab | 1.01 | 0.710 | 1.45 | 0.942 | 69 | 63 |
| Etanercept | 1.18 | 0.73 | 1.90 | 0.496 | 40 | 35 |
| Tocilizumab | 2.14 | 1.02 | 4.50 | 0.0452 | 24 | 10 |
| Hydroxychloroquine | 0.79 | 0.53 | 1.19 | 0.262 | 48 | 56 |
| Leflunomide | 0.89 | 0.56 | 1.43 | 0.636 | 36 | 42 |
| Sulfasalazine | 0.80 | 0.53 | 1.22 | 0.305 | 44 | 52 |
| Minocycline | 1.56 | 0.73 | 3.30 | 0.248 | 17 | 11 |
| Abatacept | 0.47 | 0.23 | 0.95 | 0.0349 | 14 | 25 |
| Tofacitinib | 1.90 | 0.92 | 3.91 | 0.0835 | 22 | 12 |
| Upadacitinib | - | - | - | - | - | - |
| **Bipolar disorder** | | | | | | |
| **Naproxen** | **0.52** | **0.41** | **0.66** | **$3.19\times10^{-8}$** | **120** | **213** |
| **Diclofenac** | **0.58** | **0.47** | **0.71** | **$3.33\times10^{-7}$** | **151** | **243** |
| Celecoxib | 0.72 | 0.54 | 0.96 | 0.0255 | 88 | 108 |
| Infliximab | 0.57 | 0.29 | 1.14 | 0.113 | 14 | 21 |
| Adalimumab | 0.81 | 0.61 | 1.07 | 0.145 | 100 | 111 |
| Etanercept | 1 | 0.71 | 1.42 | 0.982 | 76 | 68 |
| Tocilizumab | 1.16 | 0.64 | 2.12 | 0.621 | 26 | 18 |
| Hydroxychloroquine | 0.79 | 0.66 | 0.96 | 0.0172 | 218 | 248 |
| Leflunomide | 0.64 | 0.43 | 0.94 | 0.0218 | 49 | 72 |
| Sulfasalazine | 0.73 | 0.34 | 1.56 | 0.412 | 11 | 16 |
| Minocycline | 0.89 | 0.46 | 1.71 | 0.727 | 17 | 20 |
| Abatacept | 1.15 | 0.64 | 2.07 | 0.636 | 26 | 21 |
| Tofacitinib | 1.08 | 0.66 | 1.76 | 0.772 | 38 | 29 |
| Upadacitinib | 1.32 | 0.6 | 2.92 | 0.494 | 22 | 9 |
| **Major Depressive Disorder** | | | | | | |
| **Naproxen** | **0.78** | **0.72** | **0.85** | **$2.12\times10^{-8}$** | **900** | **1110** |
| **Diclofenac** | **0.77** | **0.71** | **0.83** | **$7.29\times10^{-11}$** | **1056** | **1317** |
| **Celecoxib** | **0.84** | **0.76** | **0.94** | **0.00135** | **642** | **645** |

| | | | | | | |
|---|---|---|---|---|---|---|
| Infliximab | 0.57 | 0.44 | 0.74 | 0.0022 | 82 | 133 |
| Adalimumab | 0.98 | 0.88 | 1.08 | 0.631 | 763 | 732 |
| Etanercept | 1.01 | 0.9 | 1.13 | 0.892 | 564 | 527 |
| Tocilizumab | 1.02 | 0.83 | 1.26 | 0.84 | 168 | 138 |
| Hydroxychloroquine | 0.94 | 0.88 | 1 | 0.0576 | 1692 | 1681 |
| Leflunomide | 0.8 | 0.7 | 0.93 | 0.00241 | 333 | 393 |
| Sulfasalazine | 0.68 | 0.53 | 0.87 | 0.00262 | 92 | 130 |
| Minocycline | 0.71 | 0.55 | 0.91 | 0.00779 | 95 | 125 |
| Abatacept | 0.97 | 0.82 | 1.14 | 0.692 | 243 | 219 |
| Tofacitinib | 0.97 | 0.82 | 1.14 | 0.697 | 259 | 221 |
| Upadacitinib | 0.86 | 0.64 | 1.15 | 0.309 | 131 | 77 |
| **Anxiety Disorders** | | | | | | |
| **Naproxen** | **0.76** | **0.7** | **0.82** | **$6.31\times10^{-12}$** | **1033** | **1302** |
| **Diclofenac** | **0.76** | **0.71** | **0.82** | **$2.14\times10^{-13}$** | **1173** | **1472** |
| **Celecoxib** | **0.76** | **0.69** | **0.84** | **$2.07\times10^{-8}$** | **708** | **775** |
| Infliximab | 0.69 | 0.56 | 0.86 | 0.0052 | 126 | 166 |
| **Adalimumab** | **0.85** | **0.77** | **0.93** | **0.00046** | **834** | **895** |
| Etanercept | 1.06 | 0.95 | 1.18 | 0.314 | 654 | 586 |
| Tocilizumab | 0.88 | 0.72 | 1.06 | 0.177 | 177 | 170 |
| **Hydroxychloroquine** | **0.84** | **0.79** | **0.89** | **$1.36\times10^{-8}$** | **1901** | **2093** |
| Leflunomide | 0.83 | 0.73 | 0.95 | 0.00517 | 409 | 453 |
| **Sulfasalazine** | **0.62** | **0.48** | **0.8** | **0.000246** | **84** | **136** |
| Minocycline | 0.85 | 0.68 | 1.05 | 0.121 | 133 | 155 |
| Abatacept | 0.86 | 0.74 | 1.01 | 0.0583 | 267 | 265 |
| Tofacitinib | 0.89 | 0.77 | 1.03 | 0.13 | 287 | 259 |
| Upadacitinib | 0.7 | 0.5 | 0.98 | 0.0374 | 141 | 88 |

Cohort of individuals with rheumatoid arthritis who initiated pharmacological treatment at age ≤45 years. rRMTL <1 indicate that the risk for psychosis is lower after low-dose methotrexate than after the comparator drug. P adjusted using Bonferroni for multiple comparison: NSAIDs (naproxen, diclofenac, celecoxib): $\alpha = 0.05/3 = 0.0167$; DMARDs $\alpha = 0.05/44 = 0.0011$. Cohort size after propensity score matching: Naproxen (N=12,447), Diclofenac (N=12,916), Celecoxib (N=8,647), Infliximab (N=1,226), Adalimumab (N=8,915), Etanercept (N=6,240), Tocilizumab (N=1,617), Hydroxychloroquine (N=21,445), Leflunomide (N=3,966), Sulfasalazine (N=6,358), Minocycline (N=1,161), Abatacept (N=2,219), Tofacitinib (N=2,269), Upadacitinib (N=1,403). rRMTL, restricted mean time lost ratio; NSAIDs, non-steroidal anti-inflammatory drugs; DMARDs, disease modifying antirheumatic drugs.

## Supplementary Table S47B. Equal-treatment sensitivity analysis under unified Bonferroni denominators

| Comparator | Drug class | Outcome | rRMTL (95% CI) | p-value | α/3 (NSAID) or α/44 (DMARD) | α/14 (per-outcome unified) | α/55 (global unified) |
|---|---|---|---|---|---|---|---|
| **Naproxen** | **NSAID** | **Psychosis** | **0.69 (0.55;0.87)** | **0.002** | **0.0167** | **0.00357** | 0.000909 |
| **Naproxen** | **NSAID** | **Bipolar disorder** | **0.52 (0.41;0.66)** | **$3.19\times10^{-8}$** | **0.0167** | **0.00357** | **0.000909** |
| **Naproxen** | **NSAID** | **Major Depressive Disorder** | **0.78 (0.72;0.85)** | **$2.12\times10^{-8}$** | **0.0167** | **0.00357** | **0.000909** |
| **Naproxen** | **NSAID** | **Anxiety** | **0.76 (0.70;0.82)** | **$6.31\times10^{-12}$** | **0.0167** | **0.00357** | **0.000909** |
| **Diclofenac** | **NSAID** | **Psychosis** | **0.71 (0.56;0.89)** | **0.004** | **0.0167** | 0.00357 | 0.000909 |
| **Diclofenac** | **NSAID** | **Bipolar disorder** | **0.58 (0.47;0.71)** | **$3.33\times10^{-07}$** | **0.0167** | **0.00357** | **0.000909** |
| **Diclofenac** | **NSAID** | **Major Depressive Disorder** | **0.77 (0.71;0.83)** | **$7.29\times10^{-11}$** | **0.0167** | **0.00357** | **0.000909** |
| **Diclofenac** | **NSAID** | **Anxiety** | **0.76 (0.71;0.82)** | **$2.14\times10^{-13}$** | **0.0167** | **0.00357** | **0.000909** |
| Celecoxib | NSAID | Psychosis | 1.23 (0.86;1.74) | 0.253 | 0.0167 | 0.00357 | 0.000909 |
| Celecoxib | NSAID | Bipolar disorder | 0.72 (0.54;0.96) | 0.025 | 0.0167 | 0.00357 | 0.000909 |
| **Celecoxib** | **NSAID** | **Major Depressive Disorder** | **0.84 (0.76;0.94)** | **0.0013** | **0.0167** | **0.00357** | 0.000909 |
| **Celecoxib** | **NSAID** | **Anxiety** | **0.76 (0.69;0.84)** | **$2.07\times10^{-8}$** | **0.0167** | **0.00357** | **0.000909** |
| Infliximab | bDMARD | Psychosis | 0.54 (0.27;1.09) | 0.084 | 0.00114 | 0.00357 | 0.000909 |
| Infliximab | bDMARD | Bipolar disorder | 0.57 (0.29;1.14) | 0.110 | 0.00114 | 0.00357 | 0.000909 |
| **Infliximab** | **bDMARD** | **Major Depressive Disorder** | **0.57 (0.44;0.74)** | **0.0022** | 0.00114 | **0.00357** | 0.000909 |
| Infliximab | bDMARD | Anxiety | 0.69 (0.56;0.86) | 0.0052 | 0.00114 | 0.00357 | 0.000909 |
| Adalimumab | bDMARD | Psychosis | 1.01 (0.71;1.45) | 0.942 | 0.00114 | 0.00357 | 0.000909 |
| Adalimumab | bDMARD | Bipolar disorder | 0.81 (0.61;1.07) | 0.150 | 0.00114 | 0.00357 | 0.000909 |
| Adalimumab | bDMARD | Major Depressive Disorder | 0.98 (0.88;1.08) | 0.630 | 0.00114 | 0.00357 | 0.000909 |
| **Adalimumab** | **bDMARD** | **Anxiety** | **0.85 (0.77;0.93)** | **0.0005** | **0.00114** | **0.00357** | **0.000909** |
| Etanercept | bDMARD | Psychosis | 1.18 (0.73;1.89) | 0.496 | 0.00114 | 0.00357 | 0.000909 |
| Etanercept | bDMARD | Bipolar disorder | 1.00 (0.71;1.42) | 0.980 | 0.00114 | 0.00357 | 0.000909 |
| Etanercept | bDMARD | Major Depressive Disorder | 1.01 (0.90;1.13) | 0.890 | 0.00114 | 0.00357 | 0.000909 |
| Etanercept | bDMARD | Anxiety | 1.06 (0.95;1.18) | 0.310 | 0.00114 | 0.00357 | 0.000909 |
| Tocilizumab | bDMARD | Psychosis | 2.14 (1.01;4.50) | 0.045 | 0.00114 | 0.00357 | 0.000909 |
| Tocilizumab | bDMARD | Bipolar disorder | 1.16 (0.64;2.12) | 0.620 | 0.00114 | 0.00357 | 0.000909 |
| Tocilizumab | bDMARD | Major Depressive Disorder | 1.02 (0.83;1.26) | 0.840 | 0.00114 | 0.00357 | 0.000909 |
| Tocilizumab | bDMARD | Anxiety | 0.88 (0.72;1.06) | 0.180 | 0.00114 | 0.00357 | 0.000909 |
| Hydroxychloroquine | csDMARD | Psychosis | 0.79 (0.53;1.19) | 0.262 | 0.00114 | 0.00357 | 0.000909 |
| Hydroxychloroquine | csDMARD | Bipolar disorder | 0.79 (0.66;0.96) | 0.017 | 0.00114 | 0.00357 | 0.000909 |
| Hydroxychloroquine | csDMARD | Major Depressive Disorder | 0.94 (0.88;1.00) | 0.058 | 0.00114 | 0.00357 | 0.000909 |
| **Hydroxychloroquine** | **csDMARD** | **Anxiety** | **0.84 (0.79;0.89)** | **$1.36\times10^{-8}$** | **0.00114** | **0.00357** | **0.000909** |
| Leflunomide | csDMARD | Psychosis | 0.89 (0.56–1.43) | 0.636 | 0.00114 | 0.00357 | 0.000909 |
| Leflunomide | csDMARD | Bipolar disorder | 0.64 (0.43–0.94) | 0.022 | 0.00114 | 0.00357 | 0.000909 |

| | | | | | | | | |
|---|---|---|---|---|---|---|---|---|
| **Leflunomide** | **csDMARD** | **Major Depressive Disorder** | **0.80 (0.70–0.93)** | **0.0024** | 0.00114 | **0.00357** | 0.000909 |
| Leflunomide | csDMARD | Anxiety | 0.83 (0.73–0.95) | 0.0052 | 0.00114 | 0.00357 | 0.000909 |
| Sulfasalazine | csDMARD | Psychosis | 0.80 (0.40–1.60) | 0.528 | 0.00114 | 0.00357 | 0.000909 |
| Sulfasalazine | csDMARD | Bipolar disorder | 0.73 (0.34–1.56) | 0.410 | 0.00114 | 0.00357 | 0.000909 |
| **Sulfasalazine** | **csDMARD** | **Major Depressive Disorder** | **0.68 (0.53–0.87)** | **0.0026** | 0.00114 | **0.00357** | 0.000909 |
| **Sulfasalazine** | **csDMARD** | **Anxiety** | **0.62 (0.48–0.80)** | **0.0003** | **0.00114** | **0.00357** | **0.000909** |
| Minocycline | csDMARD | Psychosis | 1.56 (0.73–3.31) | 0.248 | 0.00114 | 0.00357 | 0.000909 |
| Minocycline | csDMARD | Bipolar disorder | 0.89 (0.46–1.71) | 0.730 | 0.00114 | 0.00357 | 0.000909 |
| Minocycline | csDMARD | Major Depressive Disorder | 0.71 (0.55–0.91) | 0.0078 | 0.00114 | 0.00357 | 0.000909 |
| Minocycline | csDMARD | Anxiety | 0.85 (0.68–1.05) | 0.120 | 0.00114 | 0.00357 | 0.000909 |
| Abatacept | bDMARD | Psychosis | 0.47 (0.23–0.95) | 0.035 | 0.00114 | 0.00357 | 0.000909 |
| Abatacept | bDMARD | Bipolar disorder | 1.15 (0.64–2.07) | 0.640 | 0.00114 | 0.00357 | 0.000909 |
| Abatacept | bDMARD | Major Depressive Disorder | 0.97 (0.82–1.14) | 0.690 | 0.00114 | 0.00357 | 0.000909 |
| Abatacept | bDMARD | Anxiety | 0.86 (0.74–1.01) | 0.058 | 0.00114 | 0.00357 | 0.000909 |
| Tofacitinib | tsDMARD | Psychosis | 1.89 (0.92–3.91) | 0.083 | 0.00114 | 0.00357 | 0.000909 |
| Tofacitinib | tsDMARD | Bipolar disorder | 1.08 (0.66–1.76) | 0.770 | 0.00114 | 0.00357 | 0.000909 |
| Tofacitinib | tsDMARD | Major Depressive Disorder | 0.97 (0.82–1.14) | 0.70 | 0.00114 | 0.00357 | 0.000909 |
| Tofacitinib | tsDMARD | Anxiety | 0.89 (0.77–1.03) | 0.130 | 0.00114 | 0.00357 | 0.000909 |
| Upadacitinib | tsDMARD | Psychosis* | - | - | - | - | - |
| Upadacitinib | tsDMARD | Bipolar disorder | 1.32 (0.60–2.92) | 0.490 | 0.00114 | 0.00357 | 0.000909 |
| Upadacitinib | tsDMARD | Major Depressive Disorder | 0.86 (0.64–1.15) | 0.310 | 0.00114 | 0.00357 | 0.000909 |
| Upadacitinib | tsDMARD | Anxiety | 0.70 (0.50–0.98) | 0.037 | 0.00114 | 0.00357 | 0.000909 |

Sensitivity analysis under unified Bonferroni denominators. α/3 is the original primary correction (3 NSAIDs per outcome); α/44 is the original secondary correction (11 DMARDs × 4 outcomes); α/14 corrects across all 14 comparators x 1 outcome family at a time; α/55 corrects across the full 55 informative low-dose methotrexate-vs-comparator outcome tests in the analysis. *One cell, upadacitinib × psychosis, is suppressed for insufficient events (printed as "-"), hence 55 rather than 56. NSAID: non-steroidal anti-inflammatory drug; DMARD: disease-modifying anti-rheumatic drugs; bDMARD: biological DMARD; csDMARD: conventional synthetic DMARD; ts-DMARD: targeted synthetic DMARD.

**Supplementary Table S48. Summary of restricted mean time lost ratio (rRMTL, 95% CI) for the risk of psychosis, bipolar disorder, major depressive disorder, and anxiety disorders at five years follow-up (low-dose methotrexate vs comparators) in females**

| Comparator | rRMTL | rRMTL (CI low) | rRMTL (CI high) | p_value | Events low-dose methotrexate | Events Comparator |
|---|---|---|---|---|---|---|
| **Psychosis** | | | | | | |
| **Naproxen** | **0.57** | **0.42** | **0.77** | **$2.17{\times}10^{-4}$** | **73** | **127** |
| **Diclofenac** | **0.66** | **0.50** | **0.88** | **0.004** | **90** | **133** |
| Celecoxib | 1.29 | 0.88 | 1.89 | 0.191 | 73 | 45 |
| Infliximab | - | - | - | - | - | - |
| Adalimumab | 1.06 | 0.66 | 1.69 | 0.808 | 41 | 35 |
| Etanercept | 1.43 | 0.82 | 2.48 | 0.205 | 34 | 27 |
| Tocilizumab | - | - | - | - | - | - |
| Hydroxychloroquine | 0.90 | 0.69 | 1.18 | 0.443 | 114 | 120 |
| Leflunomide | 0.72 | 0.41 | 1.29 | 0.269 | 22 | 29 |
| Sulfasalazine | 1.26 | 0.82 | 1.95 | 0.300 | 50 | 39 |
| Minocycline | 1.31 | 0.57 | 3.01 | 0.526 | 13 | 10 |
| Abatacept | 0.44 | 0.21 | 0.93 | 0.030 | 13 | 23 |
| Tofacitinib | - | - | - | - | - | - |
| Upadacitinib | - | - | - | - | - | - |
| **Bipolar disorder** | | | | | | |
| **Naproxen** | **0.55** | **0.42** | **0.71** | **$4.94{\times}10^{-6}$** | **99** | **166** |
| **Diclofenac** | **0.63** | **0.5** | **0.8** | **0.00013** | **127** | **185** |
| Celecoxib | 0.81 | 0.59 | 1.1 | 0.180 | 80 | 91 |
| Infliximab | 0.41 | 0.17 | 1.01 | 0.052 | 6 | 17 |
| Adalimumab | 0.89 | 0.62 | 1.27 | 0.530 | 66 | 64 |
| Etanercept | 1.09 | 0.74 | 1.61 | 0.670 | 58 | 52 |
| Tocilizumab | 1.55 | 0.77 | 3.13 | 0.220 | 23 | 12 |
| Hydroxychloroquine | 0.79 | 0.64 | 0.97 | 0.025 | 183 | 212 |
| Leflunomide | 0.64 | 0.42 | 0.98 | 0.042 | 39 | 57 |
| Sulfasalazine | 0.8 | 0.56 | 1.15 | 0.230 | 59 | 63 |
| Minocycline | 0.95 | 0.46 | 1.96 | 0.880 | 14 | 16 |
| Abatacept | 0.78 | 0.4 | 1.51 | 0.460 | 17 | 20 |
| Tofacitinib | 0.92 | 0.53 | 1.6 | 0.770 | 27 | 24 |
| Upadacitinib | 1.05 | 0.46 | 2.4 | 0.900 | 17 | 9 |
| **Major Depressive Disorder** | | | | | | |
| **Naproxen** | **0.80** | **0.72** | **0.88** | **$2.71{\times}10^{-6}$** | **722** | **875** |
| **Diclofenac** | **0.78** | **0.72** | **0.86** | **$4.01{\times}10^{-8}$** | **846** | **1042** |
| Celecoxib | 0.87 | 0.77 | 0.97 | 0.014 | 501 | 506 |

| | | | | | | |
|---|---|---|---|---|---|---|
| Infliximab | 0.77 | 0.59 | 1 | 0.052 | 81 | 100 |
| Adalimumab | 0.95 | 0.83 | 1.08 | 0.420 | 453 | 431 |
| Etanercept | 1.03 | 0.91 | 1.17 | 0.640 | 468 | 425 |
| Tocilizumab | 1 | 0.81 | 1.24 | 1 | 154 | 133 |
| Hydroxychloroquine | 0.91 | 0.85 | 0.98 | 0.011 | 1446 | 1461 |
| Leflunomide | 0.85 | 0.74 | 0.99 | 0.032 | 306 | 334 |
| Sulfasalazine | 0.93 | 0.82 | 1.06 | 0.270 | 426 | 429 |
| Minocycline | 0.93 | 0.71 | 1.22 | 0.620 | 89 | 93 |
| Abatacept | 0.89 | 0.74 | 1.07 | 0.220 | 193 | 192 |
| Tofacitinib | 0.93 | 0.77 | 1.11 | 0.410 | 202 | 183 |
| Upadacitinib | 0.93 | 0.69 | 1.25 | 0.620 | 121 | 70 |
| **Anxiety Disorders** | | | | | | |
| **Naproxen** | **0.76** | **0.70** | **0.83** | **$1.01{\times}10^{-9}$** | **833** | **1058** |
| **Diclofenac** | **0.77** | **0.71** | **0.84** | **$2.42{\times}10^{-10}$** | **974** | **1203** |
| **Celecoxib** | **0.85** | **0.76** | **0.94** | **$1.66{\times}10^{-3}$** | **593** | **600** |
| Infliximab | 0.85 | 0.67 | 1.07 | 0.170 | 107 | 121 |
| Adalimumab | 0.88 | 0.78 | 0.99 | 0.027 | 528 | 524 |
| Etanercept | 1.10 | 0.97 | 1.24 | 0.130 | 537 | 480 |
| Tocilizumab | 0.95 | 0.78 | 1.15 | 0.580 | 186 | 162 |
| **Hydroxychloroquine** | **0.86** | **0.80** | **0.91** | **$1.78{\times}10^{-6}$** | **1679** | **1799** |
| Leflunomide | 0.92 | 0.81 | 1.05 | 0.200 | 396 | 385 |
| Sulfasalazine | 0.91 | 0.81 | 1.02 | 0.092 | 520 | 532 |
| Minocycline | 1.08 | 0.86 | 1.35 | 0.510 | 128 | 117 |
| Abatacept | 0.94 | 0.80 | 1.1 | 0.450 | 262 | 238 |
| Tofacitinib | 0.91 | 0.78 | 1.08 | 0.280 | 246 | 218 |
| Upadacitinib | 0.88 | 0.63 | 1.23 | 0.450 | 149 | 76 |

Cohort of female individuals with rheumatoid arthritis who initiated pharmacological treatment at age ≤45 years. rRMTL <1 indicate that the risk for psychosis is lower after low-dose methotrexate than after the comparator drug. P adjusted using Bonferroni for multiple comparison: NSAIDs (naproxen, diclofenac, celecoxib): $\alpha = 0.05/6 = 0.0083$; DMARDs $\alpha = 0.05/88 = 0.00056$. Cohort size after propensity score matching: Naproxen (N=9,719), Diclofenac (N=10,596), Celecoxib (N=7,013), Infliximab (N=829), Adalimumab (N=5,230), Etanercept (N=4,931), Tocilizumab (N=1,316), Hydroxychloroquine (N=17,318), Leflunomide (N=3,298), Sulfasalazine (N=5,439), Minocycline (N=880), Abatacept (N=1,960), Tofacitinib (N=1,797), Upadacitinib (N=1,169). rRMTL, restricted mean time lost ratio; NSAIDs, non-steroidal anti-inflammatory drugs; DMARDs, disease modifying antirheumatic drugs.

**Supplementary Table S49. Summary of restricted mean time lost ratio (rRMTL, 95% CI) for the risk of psychosis, bipolar disorder, major depressive disorder, and anxiety disorders at five years follow-up (low-dose methotrexate vs comparators) in males**

| Comparator | rRMTL | rRMTL (CI low) | rRMTL (CI high) | p_value | Events low-dose methotrexate | Events Comparator |
|---|---|---|---|---|---|---|
| **Psychosis** | | | | | | |
| **Naproxen** | **0.50** | **0.32** | **0.81** | **0.004** | **29** | **53** |
| Diclofenac | 0.71 | 0.39 | 1.29 | 0.262 | 18 | 26 |
| Celecoxib | 1.19 | 0.66 | 2.15 | 0.565 | 28 | 19 |
| Infliximab | - | - | - | - | - | - |
| Adalimumab | 1.16 | 0.59 | 2.30 | 0.667 | 19 | 15 |
| Etanercept | 1.55 | 0.72 | 3.34 | 0.259 | 16 | 12 |
| Tocilizumab | - | - | - | - | - | - |
| Hydroxychloroquine | 1.13 | 0.65 | 1.97 | 0.654 | 30 | 24 |
| Leflunomide | 0.95 | 0.40 | 2.28 | 0.911 | 10 | 10 |
| Sulfasalazine | 0.86 | 0.43 | 1.70 | 0.666 | 15 | 18 |
| Minocycline | - | - | - | - | - | - |
| Abatacept | - | - | - | - | - | - |
| Tofacitinib | - | - | - | - | - | - |
| Upadacitinib | - | - | - | - | - | - |
| **Bipolar disorder** | | | | | | |
| Naproxen | 0.66 | 0.39 | 1.09 | 0.11 | 25 | 39 |
| **Diclofenac** | **0.33** | **0.15** | **0.72** | **0.0051** | **9** | **24** |
| Celecoxib | 0.70 | 0.35 | 1.39 | 0.31 | 17 | 17 |
| Infliximab | 1.50 | 0.53 | 4.27 | 0.45 | 9 | 7 |
| Adalimumab | 0.82 | 0.42 | 1.63 | 0.58 | 17 | 17 |
| Etanercept | 0.93 | 0.48 | 1.82 | 0.84 | 16 | 19 |
| Tocilizumab | - | - | - | - | - | - |
| Hydroxychloroquine | 0.79 | 0.44 | 1.43 | 0.44 | 21 | 25 |
| Leflunomide | 0.75 | 0.31 | 1.77 | 0.51 | 10 | 11 |
| Sulfasalazine | 0.85 | 0.39 | 1.83 | 0.68 | 13 | 14 |
| Minocycline | - | - | - | - | - | - |
| Abatacept | - | - | - | - | - | - |
| Tofacitinib | - | - | - | - | - | - |
| Upadacitinib | - | - | - | - | - | - |
| **Major Depressive Disorder** | | | | | | |
| Naproxen | 0.83 | 0.69 | 0.99 | 0.044 | 191 | 226 |
| **Diclofenac** | **0.59** | **0.45** | **0.78** | **0.00016** | **74** | **123** |
| Celecoxib | 0.76 | 0.59 | 0.98 | 0.036 | 102 | 107 |

| | | | | | | |
|---|---|---|---|---|---|---|
| Infliximab | 0.76 | 0.45 | 1.30 | 0.320 | 21 | 29 |
| Adalimumab | 0.85 | 0.67 | 1.07 | 0.160 | 122 | 128 |
| Etanercept | 0.81 | 0.63 | 1.03 | 0.085 | 104 | 131 |
| Tocilizumab | 1.22 | 0.60 | 2.51 | 0.580 | 19 | 11 |
| Hydroxychloroquine | 0.89 | 0.74 | 1.06 | 0.190 | 193 | 216 |
| Leflunomide | 0.72 | 0.51 | 1 | 0.052 | 53 | 70 |
| Sulfasalazine | 0.74 | 0.58 | 0.95 | 0.017 | 97 | 129 |
| Minocycline | 0.76 | 0.41 | 1.41 | 0.390 | 16 | 21 |
| Abatacept | 1.06 | 0.60 | 1.85 | 0.850 | 23 | 19 |
| Tofacitinib | 0.82 | 0.52 | 1.31 | 0.410 | 30 | 34 |
| Upadacitinib | 0.88 | 0.37 | 2.12 | 0.780 | 19 | 17 |
| **Anxiety Disorders** | | | | | | |
| Naproxen | 0.86 | 0.73 | 1.03 | 0.099 | 220 | 236 |
| **Diclofenac** | **0.51** | **0.39** | **0.68** | **$3.19\times10^{-6}$** | **69** | **122** |
| **Celecoxib** | **0.60** | **0.47** | **0.77** | **$4.24\times10^{-5}$** | **103** | **133** |
| Infliximab | 0.75 | 0.48 | 1.17 | 0.210 | 30 | 38 |
| **Adalimumab** | **0.62** | **0.49** | **0.77** | **$2.23\times10^{-5}$** | **119** | **161** |
| Etanercept | 0.81 | 0.65 | 1.02 | 0.075 | 119 | 144 |
| Tocilizumab | 1.07 | 0.59 | 1.96 | 0.820 | 23 | 16 |
| Hydroxychloroquine | 0.74 | 0.62 | 0.88 | 0.0007 | 189 | 251 |
| Leflunomide | 0.61 | 0.44 | 0.86 | 0.0043 | 51 | 78 |
| Sulfasalazine | 0.69 | 0.53 | 0.88 | 0.0034 | 91 | 131 |
| Minocycline | 0.57 | 0.32 | 1.02 | 0.059 | 15 | 29 |
| Abatacept | 0.65 | 0.36 | 1.17 | 0.150 | 17 | 23 |
| Tofacitinib | 0.57 | 0.35 | 0.95 | 0.031 | 22 | 35 |
| Upadacitinib | 0.96 | 0.39 | 2.39 | 0.930 | 13 | 14 |

Cohort of male individuals with rheumatoid arthritis who initiated pharmacological treatment at age ≤45 years. rRMTL <1 indicate that the risk for psychosis is lower after low-dose methotrexate than after the comparator drug. P adjusted using Bonferroni for multiple comparison: NSAIDs (naproxen, diclofenac, celecoxib): $\alpha = 0.05/6 = 0.0083$; DMARDs $\alpha = 0.05/88 = 0.00056$. Cohort size after propensity score matching: Naproxen (N=2,604), Diclofenac (N=1,013), Celecoxib (N=1,601), Infliximab (N=301), Adalimumab (N=1,606), Etanercept (N=1,188), Tocilizumab (N=227), Hydroxychloroquine (N=2,437), Leflunomide (N=622), Sulfasalazine (N=1,268), Minocycline (N=207), Abatacept (N=243), Tofacitinib (N=343), Upadacitinib (N=233). rRMTL, restricted mean time lost ratio; NSAIDs, non-steroidal anti-inflammatory drugs; DMARDs, disease modifying antirheumatic drugs.

# Supplementary Figure 4

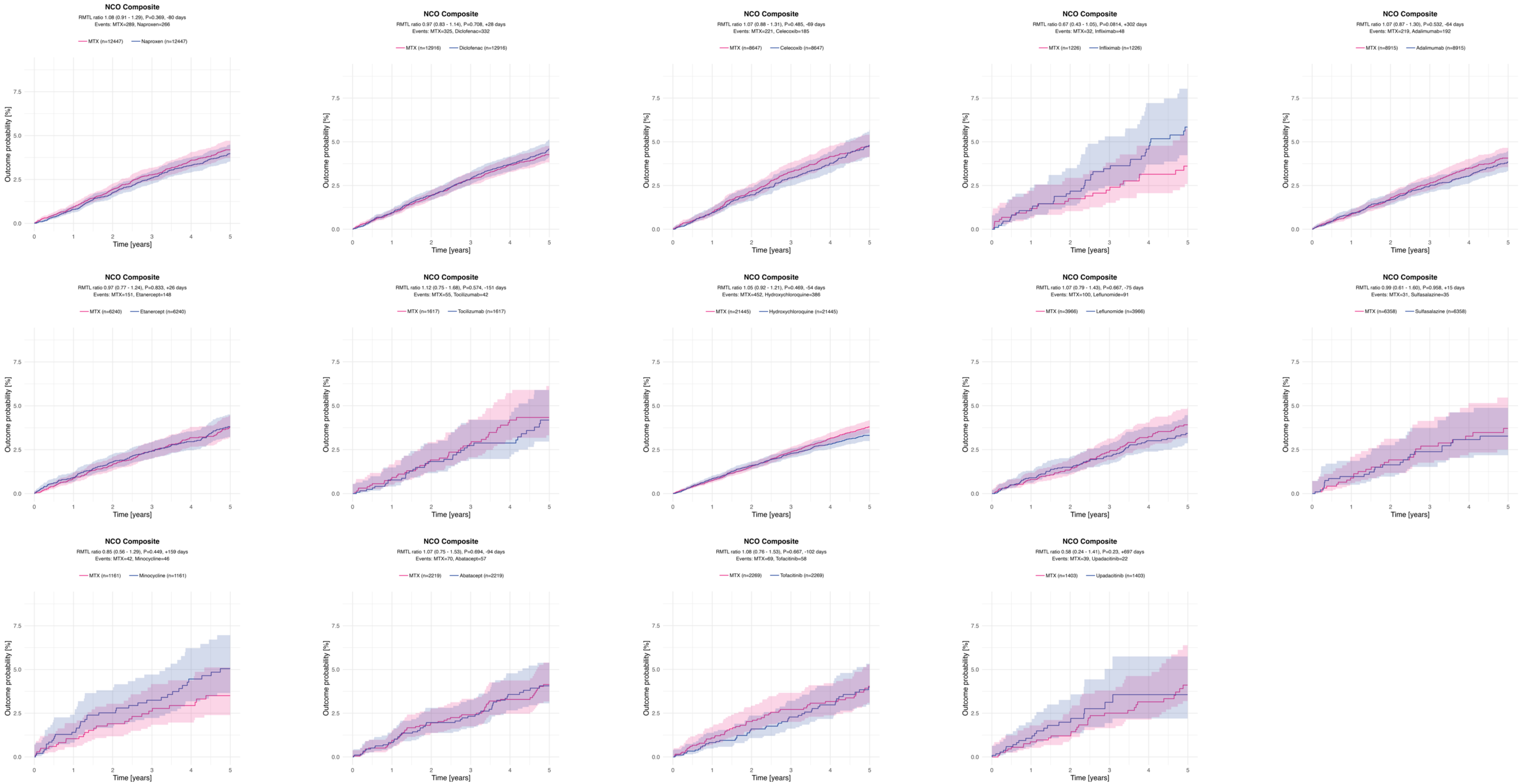


**Supplementary Figure 4.** Curves representing the Kaplan–Meier estimates of the cumulative incidence of negative control outcome. Cohort of individuals with rheumatoid arthritis who initiated pharmacological treatment at age ≤45 years.

## Supplementary Table S50. Proportional hazards diagnostics across outcome·comparator analyses

| Comparator | Outcome | Cox hazard ratio, HR (95% CI) | rRMTL (95% CI) | Schoenfeld $\chi^2$ | Schoenfeld p-value |
|---|---|---|---|---|---|
| Naproxen | Psychosis | 0.69 (0.56; 0.83) | 0.69 (0.55; 0.87) | 0.355 | 0.551 |
| Naproxen | Bipolar disorder | 0.61 (0.50; 0.74) | 0.52 (0.41; 0.66) | 3.446 | 0.063 |
| Naproxen | Major depressive disorder | 0.80 (0.74; 0.86) | 0.78 (0.72; 0.85) | 0.027 | 0.869 |
| Naproxen | Anxiety disorders | 0.79 (0.74; 0.85) | 0.76 (0.70; 0.82) | 1.209 | 0.272 |
| Diclofenac | Psychosis | 0.73 (0.60; 0.89) | 0.71 (0.56; 0.90) | 0.148 | 0.700 |
| Diclofenac | Bipolar disorder | 0.62 (0.52; 0.75) | 0.58 (0.47; 0.71) | 2.158 | 0.142 |
| Diclofenac | Major depressive disorder | 0.76 (0.71; 0.82) | 0.77 (0.71; 0.83) | 0.220 | 0.639 |
| Diclofenac | Anxiety disorders | 0.76 (0.71; 0.82) | 0.76 (0.71; 0.82) | 0.122 | 0.726 |
| Celecoxib | Psychosis | 1.09 (0.82; 1.46) | 1.23 (0.86; 1.74) | 0.157 | 0.692 |
| Celecoxib | Bipolar disorder | 0.74 (0.57; 0.95) | 0.72 (0.54; 0.96) | 0.087 | 0.767 |
| Celecoxib | Major depressive disorder | 0.84 (0.76; 0.93) | 0.84 (0.76; 0.94) | 0.089 | 0.766 |
| Celecoxib | Anxiety disorders | 0.75 (0.68; 0.82) | 0.76 (0.69; 0.84) | 0.882 | 0.348 |
| Infliximab | Psychosis | 0.54 (0.27; 1.08) | 0.54 (0.27; 1.09) | 0.145 | 0.703 |
| Infliximab | Bipolar disorder | 0.66 (0.34; 1.29) | 0.57 (0.29; 1.14) | 2.726 | 0.099 |
| Infliximab | Major depressive disorder | 0.55 (0.42; 0.73) | 0.57 (0.44; 0.74) | 0.746 | 0.388 |
| Infliximab | Anxiety disorders | 0.70 (0.56; 0.88) | 0.69 (0.56; 0.86) | 4.614 | **0.032** |
| Adalimumab | Psychosis | 0.99 (0.74; 1.33) | 1.01 (0.71; 1.45) | 0.023 | 0.879 |
| Adalimumab | Bipolar disorder | 0.96 (0.76; 1.23) | 0.81 (0.61; 1.07) | 0.823 | 0.364 |
| Adalimumab | Major depressive disorder | 0.95 (0.86; 1.04) | 0.98 (0.88; 1.08) | 2.624 | 0.105 |
| Adalimumab | Anxiety disorders | 0.88 (0.80; 0.95) | 0.85 (0.78; 0.93) | 0.237 | 0.627 |
| Etanercept | Psychosis | 1.14 (0.78; 1.67) | 1.18 (0.73; 1.90) | 0.201 | 0.654 |
| Etanercept | Bipolar disorder | 1.14 (0.84; 1.53) | 1.00 (0.71; 1.42) | 1.247 | 0.264 |
| Etanercept | Major depressive disorder | 1.04 (0.94; 1.16) | 1.01 (0.90; 1.13) | 0.679 | 0.410 |
| Etanercept | Anxiety disorders | 1.07 (0.97; 1.19) | 1.06 (0.95; 1.18) | 0.847 | 0.357 |
| Tocilizumab | Psychosis | 1.72 (0.86; 3.42) | 2.14 (1.02; 4.50) | 0.210 | 0.647 |
| Tocilizumab | Bipolar disorder | 1.24 (0.68; 2.26) | 1.16 (0.64; 2.12) | 3.116 | 0.077 |
| Tocilizumab | Major depressive disorder | 1.05 (0.84; 1.32) | 1.02 (0.83; 1.26) | 4.094 | **0.043** |
| Tocilizumab | Anxiety disorders | 0.88 (0.71; 1.08) | 0.88 (0.72; 1.06) | 3.243 | 0.072 |
| Hydroxychloroquine | Psychosis | 0.87 (0.63; 1.19) | 0.79 (0.53; 1.19) | 0.004 | 0.948 |
| Hydroxychloroquine | Bipolar disorder | 0.89 (0.76; 1.04) | 0.80 (0.66; 0.96) | 3.043 | 0.081 |
| Hydroxychloroquine | Major depressive disorder | 0.94 (0.88; 0.99) | 0.94 (0.88; 1.00) | 1.902 | 0.168 |
| Hydroxychloroquine | Anxiety disorders | 0.85 (0.81; 0.90) | 0.84 (0.79; 0.89) | 4.218 | **0.040** |
| Leflunomide | Psychosis | 0.72 (0.49; 1.04) | 0.89 (0.56; 1.43) | 1.987 | 0.159 |
| Leflunomide | Bipolar disorder | 0.70 (0.50; 0.98) | 0.64 (0.43; 0.94) | 0.112 | 0.738 |

| | | | | | |
|---|---|---|---|---|---|
| Leflunomide | Major depressive disorder | 0.78 (0.68; 0.89) | 0.80 (0.70; 0.93) | 1.115 | 0.291 |
| Leflunomide | Anxiety disorders | 0.81 (0.71; 0.91) | 0.83 (0.73; 0.95) | 0.105 | 0.746 |
| Sulfasalazine | Psychosis | 0.92 (0.65; 1.29) | 0.80 (0.53; 1.22) | 0.116 | 0.733 |
| Sulfasalazine | Bipolar disorder | 0.70 (0.33; 1.52) | 0.73 (0.34; 1.56) | 1.009 | 0.315 |
| Sulfasalazine | Major depressive disorder | 0.69 (0.53; 0.91) | 0.68 (0.53; 0.87) | 5.268 | **0.022** |
| Sulfasalazine | NCO composite | 0.91 (0.56; 1.48) | 0.99 (0.61; 1.60) | 1.087 | 0.297 |
| Sulfasalazine | Anxiety disorders | 0.61 (0.46; 0.80) | 0.62 (0.48; 0.80) | 0.136 | 0.712 |
| Minocycline | Psychosis | 1.56 (0.73; 3.34) | 1.56 (0.73; 3.31) | 0.032 | 0.857 |
| Minocycline | Bipolar disorder | 0.86 (0.45; 1.65) | 0.89 (0.46; 1.71) | 1.330 | 0.249 |
| Minocycline | Major depressive disorder | 0.72 (0.56; 0.95) | 0.71 (0.56; 0.91) | 3.732 | 0.053 |
| Minocycline | Anxiety disorders | 0.86 (0.68; 1.09) | 0.85 (0.69; 1.04) | 1.083 | 0.298 |
| Abatacept | Psychosis | 0.62 (0.35; 1.10) | 0.47 (0.23; 0.95) | 0.228 | 0.633 |
| Abatacept | Bipolar disorder | 0.96 (0.55; 1.66) | 1.15 (0.64; 2.07) | 0.241 | 0.624 |
| Abatacept | Major depressive disorder | 0.94 (0.79; 1.13) | 0.97 (0.82; 1.14) | 1.702 | 0.192 |
| Abatacept | Anxiety disorders | 0.84 (0.71; 1.00) | 0.86 (0.74; 1.00) | 0.021 | 0.883 |
| Tofacitinib | Psychosis | 1.91 (0.96; 3.78) | 1.90 (0.92; 3.91) | 0.099 | 0.753 |
| Tofacitinib | Bipolar disorder | 1.11 (0.69; 1.80) | 1.07 (0.66; 1.75) | 2.413 | 0.120 |
| Tofacitinib | Major depressive disorder | 0.95 (0.79; 1.14) | 0.97 (0.82; 1.14) | 0.000 | 0.992 |
| Tofacitinib | NCO composite | 1.04 (0.74; 1.47) | 1.08 (0.76; 1.53) | 1.900 | 0.168 |
| Tofacitinib | Anxiety disorders | 0.88 (0.74; 1.04) | 0.89 (0.77; 1.03) | 1.734 | 0.188 |
| Upadacitinib | Psychosis | - | - | - | - |
| Upadacitinib | Bipolar disorder | 1.10 (0.48; 2.50) | 1.32 (0.60; 2.92) | 5.143 | **0.023** |
| Upadacitinib | Major depressive disorder | 0.79 (0.58; 1.07) | 0.86 (0.64; 1.15) | 3.561 | 0.059 |
| Upadacitinib | NCO composite | 0.78 (0.44; 1.36) | 0.58 (0.24; 1.41) | 2.566 | 0.109 |
| Upadacitinib | Anxiety disorders | 0.74 (0.56; 0.99) | 0.70 (0.50; 0.98) | 3.874 | **0.049** |

Cox hazard ratios (HRs) were estimated for each outcome–comparator analysis. Restricted mean time lost ratios (rRMTL) were calculated over the prespecified 5-year follow-up horizon using the survRM2 package; values below 1 indicate less time lived with the outcome in the low-dose methotrexate group relative to the comparator group. Proportional hazards were assessed using the generalised Schoenfeld residual test implemented in cox.zph from the survival package; $p<0{\cdot}05$ indicates evidence of violation of the proportional hazards assumption. Values below 1 for both Cox HR and rRMTL favour low-dose methotrexate. Abbreviations: SD, standard deviation; CI, confidence interval; HR, hazard ratio; rRMTL, restricted mean time lost ratio; csDMARD, conventional synthetic DMARD; bDMARD, biological DMARD; tsDMARD, targeted synthetic DMARD; DMARD, disease-modifying antirheumatic drug.

**Supplementary Table S51A. Sensitivity cohort definition for symmetric exclusion of prior/concurrent anti-rheumatic drugs from both low-dose methotrexate and NSAID arms**

| Sensitivity cohort | Baseline medications excluded |
|---|---|
| **low-dose methotrexate cohort** | Any prior or concurrent exposure to the three NSAID comparators: **naproxen, diclofenac, or celecoxib**; and to the 11 DMARD comparators: **infliximab, adalimumab, etanercept, tocilizumab, hydroxychloroquine, leflunomide, sulfasalazine, minocycline, abatacept, tofacitinib, or upadacitinib**. |
| **Naproxen cohort** | Any prior or concurrent exposure to **low-dose methotrexate**; the other two NSAID comparators, **diclofenac or celecoxib**; and the same 11 DMARD comparators listed above. |
| **Diclofenac cohort** | Any prior or concurrent exposure to **low-dose methotrexate**; the other two NSAID comparators, **naproxen or celecoxib**; and the same 11 DMARD comparators listed above. |

Sensitivity analyses performed to assess whether baseline co-medication differences could explain the primary findings. These cohorts were constructed using the same eligibility criteria and propensity-score matching strategy as the primary cohorts, with the additional application of symmetric baseline medication exclusions across exposure groups. Specifically, individuals with prior or concurrent exposure to anti-rheumatic drugs other than the index treatment were excluded from both the low-dose methotrexate and NSAID comparator cohorts. We note that this approach isolates the drug effect from the within-arm co-medication, but it is no longer representative of routine rheumatoid arthritis care. The strategy is therefore informative as a sensitivity replication, but the previous cohorts remain the primary designs for inference about clinical practice. NSAID, non-steroidal anti-inflammatory drug; DMARD, disease-modifying anti-rheumatic drug.

**Supplementary Table S51B. Sensitivity cohort definition for symmetric exclusion of prior/concurrent anti-rheumatic drugs from both low-dose methotrexate and NSAID arms**

| Outcome | Comparator | N per arm | Events low-dose methotrexate | Events Comparator | rRMTL (95% CI) | p-value |
|---|---|---|---|---|---|---|
| Psychosis | Naproxen | 5,340 | 44 | 77 | 0.583 (0.396–0.860) | 0.006 |
| | Diclofenac | 4,228 | 33 | 51 | 0.634 (0.402–1.000) | 0.050 |
| Bipolar disorder | Naproxen | 5,340 | 65 | 115 | 0.532 (0.389–0.727) | $7.7\times10^{-5}$ |
| | Diclofenac | 4,228 | 64 | 92 | 0.647 (0.466–0.899) | 0.009 |
| Major depressive disorder | Naproxen | 5,340 | 526 | 619 | 0.807 (0.720–0.904) | $2.1\times10^{-4}$ |
| | Diclofenac | 4,228 | 439 | 552 | 0.737 (0.654–0.831) | $6.0\times10^{-7}$ |
| Anxiety disorders | Naproxen | 5,340 | 574 | 725 | 0.739 (0.665–0.822) | $2.6\times10^{-8}$ |
| | Diclofenac | 4,228 | 476 | 638 | 0.687 (0.614–0.769) | $6.9\times10^{-11}$ |

Sensitivity analyses performed to assess whether baseline co-medication differences could explain the primary findings. See Supplementary Table S51A for information on how the cohorts were constructed. rRMTL, restricted mean time lost ratio; CI, confidence interval; N, number per arm.

# Supplementary Figure 5A

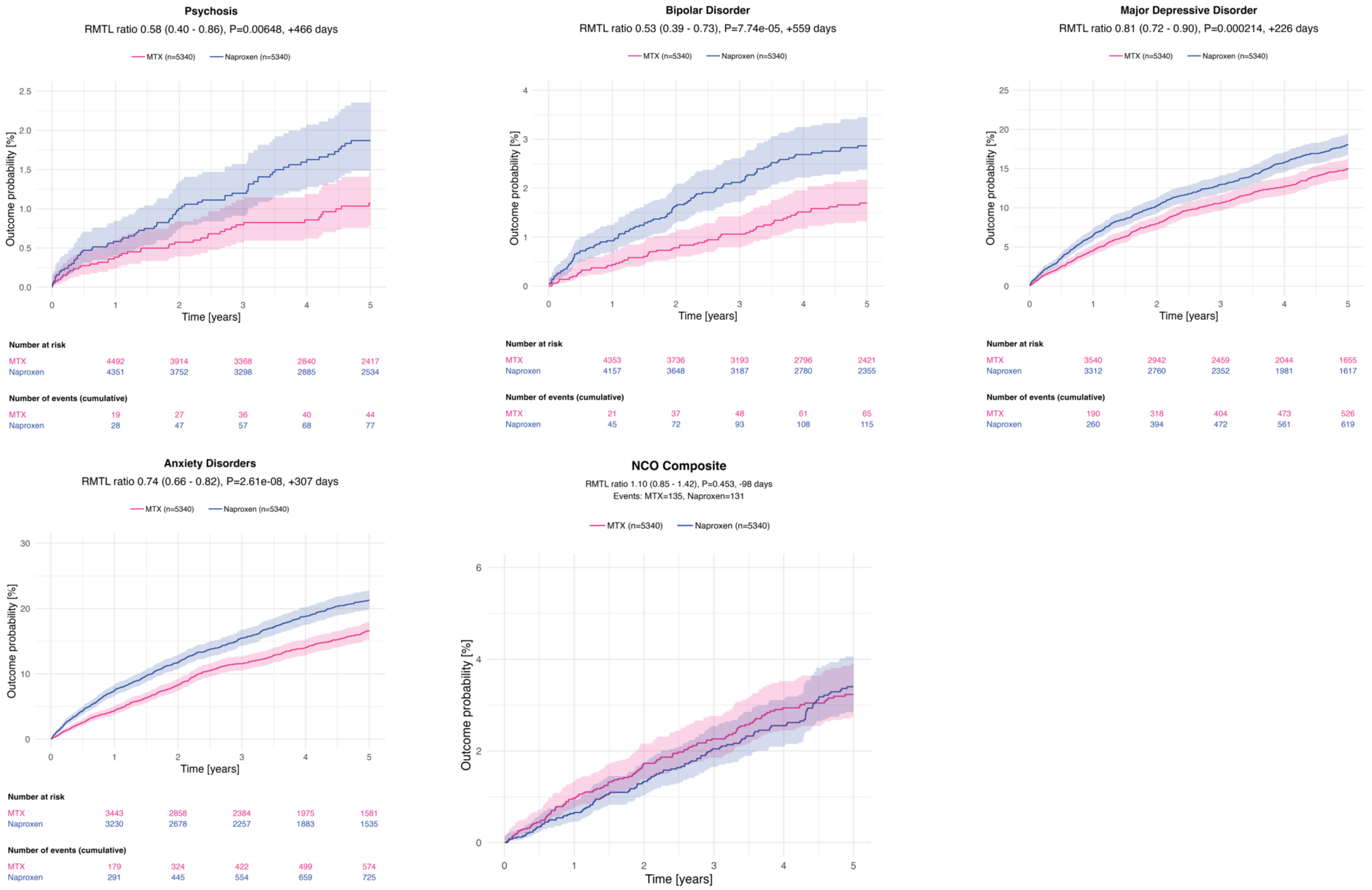


**Supplementary Figure 5A**. Sensitivity analyses performed to assess whether baseline co-medication differences could explain the primary findings. These cohorts were constructed using the same eligibility criteria and propensity-score matching strategy as the primary cohorts, with the additional application of symmetric baseline medication exclusions across exposure groups. Specifically, individuals with prior or concurrent exposure to anti-rheumatic drugs other than the index treatment were excluded from both the low-dose methotrexate and NSAID comparator cohorts. Curves represent the Kaplan–Meier estimates of the cumulative incidence for psychosis and secondary psychiatric outcomes after initiation of low-dose methotrexate (MTX) *vs* comparator drugs. Cohort of individuals with rheumatoid arthritis who initiated pharmacological treatment at age ≤45 year. rRMTL <1 indicate that the risk for bipolar disorder is lower after low-dose methotrexate than after the comparator drug, and conversely for rRMTL >1. Cohort size after propensity score matching: Naproxen (N=5,340). NCO: Negative control outcome. NSAID, non-steroidal anti-inflammatory drug; rRMTL, restricted mean time lost ratio.

# Supplementary Figure 5B

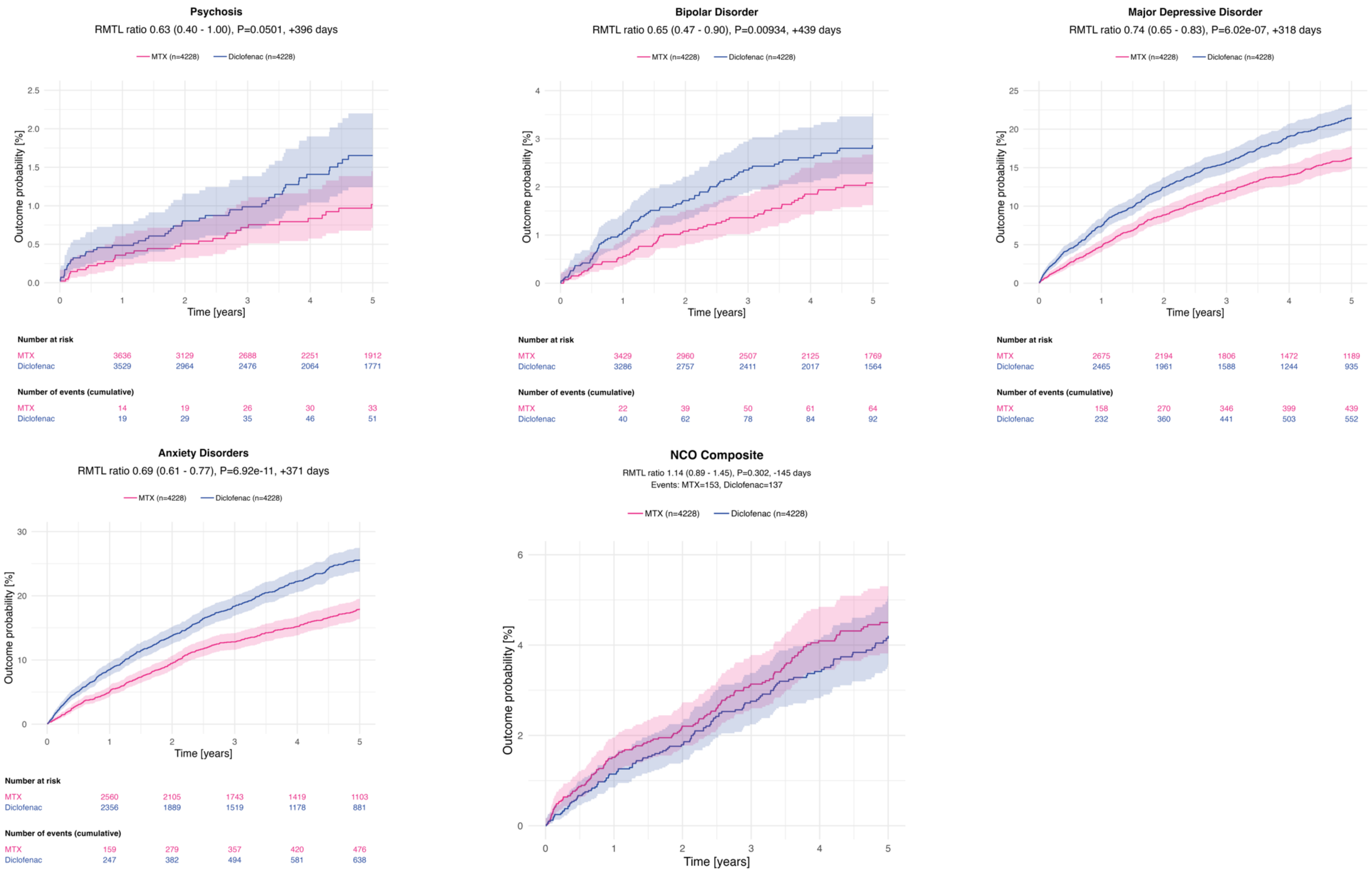


**Supplementary Figure 5B** Sensitivity analyses performed to assess whether baseline co-medication differences could explain the primary findings. These cohorts were constructed using the same eligibility criteria and propensity-score matching strategy as the primary cohorts, with the additional application of symmetric baseline medication exclusions across exposure groups. Specifically, individuals with prior or concurrent exposure to anti-rheumatic drugs other than the index treatment were excluded from both the low-dose methotrexate and NSAID comparator cohorts. Curves represent the Kaplan–Meier estimates of the cumulative incidence for psychosis and secondary psychiatric outcomes after initiation of low-dose methotrexate (MTX) *vs* comparator drugs. Cohort of individuals with rheumatoid arthritis who initiated pharmacological treatment at age ≤45 year. rRMTL <1 indicate that the risk for bipolar disorder is lower after low-dose methotrexate than after the comparator drug, and conversely for rRMTL >1. Cohort size after propensity score matching: Diclofenac (N=4,228). NCO: Negative control outcome. NSAID, non-steroidal anti-inflammatory drug; rRMTL, restricted mean time lost ratio.
